# ProbOnto: ontology and knowledge base of probability distributions

**DOI:** 10.1093/bioinformatics/btw170

**Published:** 2016-04-03

**Authors:** Maciej J. Swat, Pierre Grenon, Sarala Wimalaratne

**Affiliations:** ^1^EMBL-European Bioinformatics Institute, Wellcome Trust Genome Campus, Hinxton, Cambridgeshire CB10 1SD, UK; ^2^CHIME, University College London, London, UK

## Abstract

**Motivation:** Probability distributions play a central role in mathematical and statistical modelling. The encoding, annotation and exchange of such models could be greatly simplified by a resource providing a common reference for the definition of probability distributions. Although some resources exist, no suitably detailed and complex ontology exists nor any database allowing programmatic access.

**Results:** ProbOnto, is an ontology-based knowledge base of probability distributions, featuring more than 80 uni- and multivariate distributions with their defining functions, characteristics, relationships and re-parameterization formulas. It can be used for model annotation and facilitates the encoding of distribution-based models, related functions and quantities.

**Availability and Implementation:** http://probonto.org

**Contact:** mjswat@ebi.ac.uk

**Supplementary information:** Supplementary data are available at *Bioinformatics* online.

## 1 Introduction

When encoding probabilistic uncertainties using a given parametric distribution, its name and parameters are usually sufficient to specify the intended distribution unambiguously, as in many cases such parameter set is unique. However, in multiple cases, two or more parameterizations exist and it becomes essential to specify correctly the parameters for the distribution in order to obtain the correct model. A well-structured, independent, standard reference would greatly facilitate the specification of a distribution and its declaration in a programming language or exchange format as is shown by interoperability issues between tools with distributions differing in their parameterizations (LeBauer *et al.*, 2013).

Many resources are available online (Dinov *et al.*, 2015; Williams *et al.*, 2008), as tools (Marichev and Trott, 2013) and in printed format (Forbes *et al.*, 2011; Johnson *et al.*, 2005; Leemis and Mcqueston, 2008) but a comprehensive ontology which formalizes the theory of kinds and relations of probability distributions does not yet exist.

Some Bioportal (Noy *et al.*, 2009) ontologies provide a simple classification and little information; in most cases parameters, defining functions, or quantities are not defined and information about the relationships between distributions is not available. Such ontologies, because of their simplicity, are insufficient for our purposes and it is the same with existing databases of distributions.

## 2 Methods

We compiled an initial ‘common denominator’ collection of parametric distributions from UncertML (Williams *et al.*, 2008) and Matlab Statistical Toolbox (MathWorks, 2015) and then extended this set to cover models used in various statistical modelling areas, in particular, pharmacometrics. A number of important and more exotic discrete data models were treated with alternative versions of the Negative Binomial and the Generalized Poisson, or the Conway–Maxwell–Poisson distribution.

We further included all distributions and relevant parameterizations used in the following tools: Monolix (Lixoft Team, 2014), NONMEM (Beal *et al.*, 2009) and winBUGS (Lunn *et al.*, 2009), and a few found in MCSim (Bois, 2010). We completed this collection with many relationships/re-parameterizations between distributions (Leemis and Mcqueston, 2008).

We used the following sources to populate the database with probability functions, their relationships and quantities: Forbes *et al.* (2011), Johnson *et al.* (2005) and the probability distribution pages of the Wikipedia.

The following list summarizes the features of ProbOnto 1.0

- Over 80 uni- and multivariate distributions and alternative parameterizations.
- Probability density or mass functions and available cumulative distribution, hazard and survival functions.
- Supports encoding of univariate mixture distributions.
- Over 130 relationships and re-parameterization formulas.
- Related quantities such as mean, median, mode and variance.
- Parameter and support/range definitions and distribution type.
- Latex and R code for mathematical functions.

LeBauer *et al.* (2013) showed that re-parameterizations between related distributions are essential for the interoperability between existing tools using only two tools and five distributions. ProbOnto extended coverage in this respect beyond proof of concept with a wider scope and number of tools and distributions. ProbOnto contains, for example, six alternative parameterizations of the log-normal or five of the negative binomial distribution, all in use in different contexts. Scientists and tool developers can look all these up in ProbOnto (Supplementary Material).

## 3 Ontological model

ProbOnto is a knowledge base built from a simple ontological model. At its core, a probability distribution is an instance of the class thereof, a specialization of the class of mathematical objects. A distribution relates to a number of other individuals, which are instances of various categories in the ontology. For example, these are parameters and related functions associated with a given probability distribution. This strategy allows for the rich representation of attributes and relationships between domain objects. The ontology can be seen as a conceptual schema in the domain of mathematics and has been implemented as a PowerLoom (MacGregor *et al.*, 1997) knowledge base. An OWL version is generated programmatically using the Jena API (McBride, 2001). Output for ProbOnto are provided as supplementary materials and published on or linked from the ProbOnto website. The OWL version of ProbOnto is available via Ontology Lookup Service (OLS) to facilitate simple searching and visualization of the content (Jupp *et al.*, 2015). In addition the OLS API provides methods to programmatically access ProbOnto and to integrate it into applications.

## 4 Use case

ProbOnto was first designed to facilitate the encoding of nonlinear mixed effect models and their annotation in PharmML, Pharmacometrics Markup Language, (Swat *et al.*, 2015) developed by DDMoRe (Harnisch *et al.*, 2013). The scope and features of the language made ProbOnto invaluable in encoding of diverse models applicable to discrete (e.g. count, categorical and time-to-event) and continuous data (Supplementary Material).

Despite its PharmML original context, ProbOnto is purpose independent and does not put implementation constraints on tool designers, thus allowing an open ended number of usage scenarios.

When using ProbOnto with PharmML, a small generic XML schema was enough to allow for flexible encoding of distributions relevant in pharmacometric modelling, their parameters and functions (see Supplementary Material, Appendix). The following example shows how the negative binomial distribution is encoded by using its codename and declaring that of its parameters (‘rate’ and ‘overdispersion’).

<Distribution>

 <po:ProbOnto name=“NegativeBinomial2”>

  <po:Parameter name=“rate”>

   <ct:Assign>

    <ct:SymbRef symbIdRef=“lambda”/>

   </ct:Assign>

  </po:Parameter>

  <po:Parameter name=“overdispersion”>

   <ct:Assign>

    <ct:SymbRef symbIdRef=“tau”/>

   </ct:Assign>

  </po:Parameter>

 </po:ProbOnto>

</Distriution>

To specify any given distribution unambiguously using ProbOnto, it is sufficient to declare its code name and the code names of its parameters.

## 5 Future plans and conclusions

ProbOnto provides a means for the encoding and annotation of statistical models, thus facilitating their exchange between software tools. Due to its generic construction which does not enforce a specific implementation in target software, ProbOnto can be applied across various modelling platforms and databases.

Although it already incorporates a high number of distributions, this collection is still growing with new distributions added on regular basis; ProbOnto will ultimately include all distributions supported by tools such as STAN (STAN Development Team, 2015) and R (R Core Team, 2015). In the future, additional features will extend the knowledge base such as applications of a probability distribution, its mathematical properties, such as linear combination, convolution and scaling (Leemis and Mcqueston, 2008) and related data type.

## Supplementary Material

Supplementary Data
