# Supplementary material for: ProbOnto: ontology and knowledge base of probability distributions
Source: Bioinformatics. 2016 Apr 3;32(17):2719–21. doi: 10.1093/bioinformatics/btw170 (PMC5013898; doi:10.1093/bioinformatics/btw170)
Supplement: Supplementary Data [file supp_32_17_2719__index.html]

ProbOnto: ontology and knowledge base of probability distributions — ProbOnto: ontology and knowledge base of probability distributions — Supplementary Data 

# ProbOnto: ontology and knowledge base of probability distributions

## Supplementary Data

files

- Supplementary Data - pdf file
- Supplementary Data - pdf file
