# Supplementary material for: ProbOnto: ontology and knowledge base of probability distributions
Source: Bioinformatics. 2016 Apr 3;32(17):2719–21. doi: 10.1093/bioinformatics/btw170 (PMC5013898; doi:10.1093/bioinformatics/btw170)
Supplement: Supplementary Data [file supp_btw170_Swat_et_al_Supplementary1_ProbOnto_KBprintout.pdf]

Supplementary materials part 1/2

M.J. Swat et al.

ProbOnto – Ontology and Knowledge Base of Probability Distributions

ProbOnto Knowledge Base

Version 1.0 (March 2016)

For updates visit http://probonto.org

Contents

List of Figures **1**

1 Knowledge base overview **4**
1.1 Symbols 4
1.2 Distributions – properties and relationships 6

References **118**

List of Figures

1.1 Bernoulli1 distribution plotted using the provided R code. . . . 6
1.2 Beta1 distribution plotted using the provided R code. . . . 7
1.3 BetaBinomial1 distribution plotted using the provided R code. . . . 9
1.4 Binomial1 distribution plotted using the provided R code. . . . 10
1.5 BirnbaumSaunders1 distribution plotted using the provided R code. . . . 12
1.6 CategoricalNonordered1 distribution plotted using the provided R code. . . . 13
1.7 CategoricalOrdered1 distribution plotted using the provided R code. . . . 14
1.8 Cauchy1 distribution plotted using the provided R code. . . . 15
1.9 ChiSquared1 distribution plotted using the provided R code. . . . 16
1.10 ConwayMaxwellPoisson1 distribution plotted using the provided R code. . . . 18
1.11 DoublePoisson1 distribution plotted using the provided R code. . . . 20
1.12 Erlang1 distribution plotted using the provided R code. . . . 22
1.13 Exponential1 distribution plotted using the provided R code. . . . 23
1.14 Exponential2 distribution plotted using the provided R code. . . . 25
1.15 F1 distribution plotted using the provided R code. . . . 26
1.16 Gamma1 distribution plotted using the provided R code. . . . 28
1.17 Gamma2 distribution plotted using the provided R code. . . . 29
1.18 GeneralizedGamma1 distribution plotted using the provided R code. . . . 31
1.19 GeneralizedGamma2 distribution plotted using the provided R code. . . . 32
1.20 GeneralizedGamma3 distribution plotted using the provided R code. . . . 34
1.21 GeneralizedNegativeBinomial1 distribution plotted using the provided R code. . . . 35
1.22 GeneralizedPoisson1 distribution plotted using the provided R code. . . . 37
1.23 GeneralizedPoisson3 distribution plotted using the provided R code. . . . 38
1.24 GeneralizedPoisson2 distribution plotted using the provided R code. . . . 39
1.25 Geometric1 distribution plotted using the provided R code. . . . 40
1.26 Gompertz1 distribution plotted using the provided R code. . . . 42
1.27 Gumbel1 distribution plotted using the provided R code. . . . 43
1.28 HalfNormal1 distribution plotted using the provided R code. . . . 44
1.29 Hypergeometric1 distribution plotted using the provided R code. . . . 45
1.30 InverseBinomial1 distribution plotted using the provided R code. . . . 47
1.31 InverseGamma1 distribution plotted using the provided R code. . . . 48
1.32 InverseGaussian1 distribution plotted using the provided R code. . . . 49
1.33 Laplace1 distribution plotted using the provided R code. . . . 51
1.34 Laplace2 distribution plotted using the provided R code. . . . 52
1.35 LogLogistic1 distribution plotted using the provided R code. . . . 54
1.36 LogLogistic2 distribution plotted using the provided R code. . . . 55
1.37 LogNormal1 distribution plotted using the provided R code. . . . 57
1.38 LogNormal2 distribution plotted using the provided R code. . . . 59
1.39 LogNormal3 distribution plotted using the provided R code. . . . 60
1.40 LogNormal4 distribution plotted using the provided R code. . . . 62
1.41 LogNormal5 distribution plotted using the provided R code. . . . 64
1.42 LogNormal6 distribution plotted using the provided R code. . . . 66
1.43 LogUniform1 distribution plotted using the provided R code. . . . 68
1.44 Logistic1 distribution plotted using the provided R code. . . . 69
1.45 Logistic2 distribution plotted using the provided R code. . . . 70
1.46 Example 1: PMF and CDF of the Mixture Poisson distribution 72
1.47 Example 2: PDF and CDF of the Mixture Normal distribution 72
1.48 Nakagami1 distribution plotted using the provided R code. . . . 77
1.49 NegativeBinomial1 distribution plotted using the provided R code. . . . 78

1.50 NegativeBinomial2 distribution plotted using the provided R code. 80
1.51 NegativeBinomial3 distribution plotted using the provided R code. 82
1.52 NegativeBinomial4 distribution plotted using the provided R code. 83
1.53 NegativeBinomial5 distribution plotted using the provided R code. 85
1.54 Normal1 distribution plotted using the provided R code. 87
1.55 Normal2 distribution plotted using the provided R code. 89
1.56 Normal3 distribution plotted using the provided R code. 91
1.57 NormalInverseGamma1 distribution plotted using the provided R code. 92
1.58 Pareto1 distribution plotted using the provided R code. 93
1.59 Poisson1 distribution plotted using the provided R code. 95
1.60 Rayleigh1 distribution plotted using the provided R code. 97
1.61 StandardNormal1 distribution plotted using the provided R code. 98
1.62 StandardUniform1 distribution plotted using the provided R code. 100
1.63 StudentT1 distribution plotted using the provided R code. 101
1.64 StudentT2 distribution plotted using the provided R code. 103
1.65 Triangular1 distribution plotted using the provided R code. 104
1.66 TruncatedNormal1 distribution plotted using the provided R code. 105
1.67 Uniform1 distribution plotted using the provided R code. 107
1.68 UniformDiscrete1 distribution plotted using the provided R code. 108
1.69 UniformDiscrete2 distribution plotted using the provided R code. 109
1.70 Weibull1 distribution plotted using the provided R code. 111
1.71 Weibull2 distribution plotted using the provided R code. 112
1.72 ZeroInflatedGeneralizedPoisson1 distribution plotted using the provided R code. 115
1.73 ZeroInflatedNegativeBinomial1 distribution plotted using the provided R code. 116
1.74 ZeroInflatedPoisson1 distribution plotted using the provided R code. 117

Chapter 1

Knowledge base overview

This document provides detailed summary of the most important features for supported univariate and multivariate distributions. After the symbol definitions we list for each distribution its

- type
- support definition
- parameters
- defining functions (with R code)
- quantities such as mean, median, mode and variance
- relationships and re-parameterisations
- (for univariate distributions only) PDF/PMF and CDF plots based on the R code stored in the KB.

1.1 Symbols

Some of the symbols used in definitions of the functions and quantities listed in the subsequent sections are collected here with references

- Beta function, $B(x,y)$

  $$B(x,y) = \frac{\Gamma(x)\Gamma(y)}{\Gamma(x+y)}$$

  `mathworld.wolfram.com/BetaFunction.html`
  `en.wikipedia.org/wiki/Beta_function`

- Regularized incomplete Beta function, $I_p(a,b)$, $I_{1-p}(a,b)$

  $$I_x(a,b) = \frac{B(x;a,b)}{B(a,b)}$$

  with

  $$B(x;a,b) = \int_0^x t^{a-1}(1-t)^{b-1}dt$$

  then *incomplete beta function*.
  `mathworld.wolfram.com/RegularizedBetaFunction.html`
  `en.wikipedia.org/wiki/Beta_function#Incomplete_beta_function`

- Error function, erf

  $$\mathrm{erf}(z) = \frac{2}{\pi}\int_0^z e^{-t^2}dt$$

  `mathworld.wolfram.com/Erf.html`
  `en.wikipedia.org/wiki/Error_function`

- Euler constant, γ_E

$$\gamma_E = \lim_{n\to\infty}\Big(-\log(n)\sum_{k=1}^{n}\frac{1}{k}\Big) = \int_1^\infty \Big(\frac{1}{\lfloor x\rfloor} - \frac{1}{x}\Big)dx$$

mathworld.wolfram.com/Euler-MascheroniConstant.html
en.wikipedia.org/wiki/Euler%E2%80%93Mascheroni_constant

- Floor function, $\lfloor x\rfloor$

$$\text{floor}(x) = \lfloor x\rfloor \text{ is the largest integer not greater than } x$$

mathworld.wolfram.com/FloorFunction.html
en.wikipedia.org/wiki/Floor_and_ceiling_functions

- Gamma function, Γ

$$\Gamma(n) = (n-1)!, \text{ for } n - \text{ positive integer}$$

mathworld.wolfram.com/GammaFunction.html
en.wikipedia.org/wiki/Gamma_function

- Lower incomplete gamma function, $\gamma(s,x)$

$$\gamma(s,x) = \int_0^x t^{s-1}\,e^{-t}\,\mathrm{d}t$$

mathworld.wolfram.com/IncompleteGammaFunction.html
https://en.wikipedia.org/wiki/Incomplete_gamma_function#Lower_incomplete_Gamma_function
https://cran.r-project.org/web/packages/zipfR/zipfR.pdf

- Multivariate Gamma function, Γ_p

$$\Gamma_p(a) = \pi^{p(p-1)/4}\prod_{j=1}^{p}\Gamma\left[a + (1-j)/2\right]$$

en.wikipedia.org/wiki/Multivariate_gamma_function

- Iverson bracket, $[x = i]$

$$[P] = \begin{cases} 1 & \text{if } P \text{ is true;} \\ 0 & \text{otherwise.} \end{cases}$$

mathworld.wolfram.com/IversonBracket.html
en.wikipedia.org/wiki/Iverson_bracket

- Pochhammer symbol, $(x)_n$

$$(x)_n = x(x-1)(x-2)\cdots(x-n+1).$$

http://mathworld.wolfram.com/PochhammerSymbol.html
https://en.wikipedia.org/wiki/Pochhammer_symbol

- Generalized Hypergeometric function, ${}_pF_q$

$${}_pF_q(a_1,\ldots,a_p;b_1,\ldots,b_q;z) = \sum_{n=0}^{\infty}\frac{(a_1)_n\ldots(a_p)_n}{(b_1)_n\ldots(b_q)_n}\frac{z^n}{n!}$$

en.wikipedia.org/wiki/Generalized_hypergeometric_function

- Hypergeometric function, ${}_2F_1$

$${}_2F_1(a,b;c;z) = \sum_{n=0}^{\infty}\frac{(a)_n(b)_n}{(c)_n}\frac{z^n}{n!}.$$

mathworld.wolfram.com/HypergeometricFunction.html
en.wikipedia.org/wiki/Hypergeometric_function

1.2 Distributions – properties and relationships

Bernoulli1

**name**	Bernoulli 1 (ID: 0000000)
**type**	discrete
**variate**	k, scalar
**support**	$k \in \{0, 1\}$

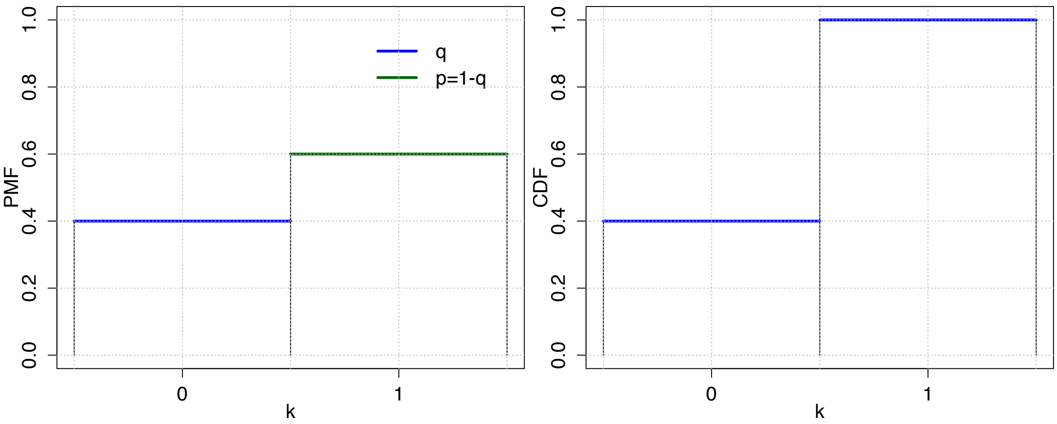

Figure 1.1: Bernoulli1 distribution plotted using the provided R code.

Model

A Bernoulli trial is a probabilistic experiment that can have one of two outcomes, success (x = 1) or failure (x = 0), and in which the probability of success is p.

Parameter: probability

**name**	probability of success
**type**	scalar
**symbol**	p
**definition**	$0 < p < 1, p \in R$

Functions

PMF

$$\begin{cases} q = (1-p) & \text{for } k = 0 \\ p & \text{for } k = 1 \end{cases}$$

PMF in R

```
q=(1-p) for k=0 \\
p for k=1
```

CDF

$$\begin{cases} 0 & \text{for } k < 0 \\ q & \text{for } 0 \leq k < 1 \\ 1 & \text{for } k \geq 1 \end{cases}$$

Characteristics

Mean

$$p$$

Median

$$\begin{cases} 0 & \text{if } q > p \\ 0.5 & \text{if } q = p \\ 1 & \text{if } q < p \end{cases}$$

Mode

$$\begin{cases} 0 & \text{if } q > p \\ 0,1 & \text{if } q = p \\ 1 & \text{if } q < p \end{cases}$$

Variance

$$p(1-p)$$

Relationships

- Relationship pair: $Bernoulli1(p) \rightarrow Binomial1(n, p)$
- Relationship type: Transformation
- Relationship definition: $\Sigma X(iid)$

- Relationship pair: $Binomial1(n, p) \rightarrow Bernoulli1(p)$
- Relationship type: Special case
- Relationship definition: $n = 1$

References

[Leemis and Mcqueston, 2008]
`https://en.wikipedia.org/wiki/Bernoulli_distribution`
`http://www.uncertml.org/distributions/bernoulli`
`http://www.math.wm.edu/~leemis/chart/UDR/PDFs/BinomialBernoulli.pdf`
`http://www.math.wm.edu/~leemis/chart/UDR/PDFs/BernoulliBinomial.pdf`

Beta1

**name**	Beta 1 (ID: 0000057)
**type**	continuous
**variate**	x, scalar
**support**	$x \in (0, 1)$

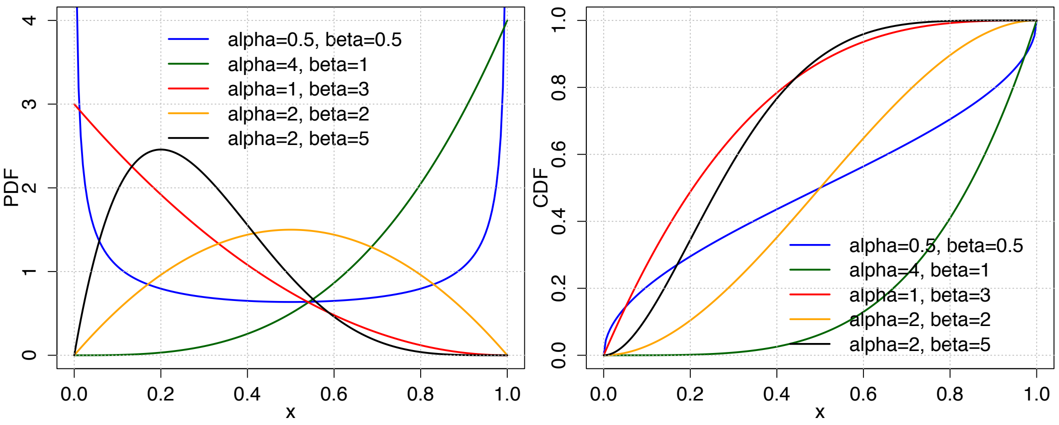

Figure 1.2: Beta1 distribution plotted using the provided R code.

Parameter: alpha

**name**	shape
**type**	scalar
**symbol**	α
**definition**	$\alpha > 0$

Parameter: beta

**name**	shape
**type**	scalar
**symbol**	β
**definition**	$\beta > 0$

Functions

PDF

$$\frac{x^{\alpha-1}(1-x)^{\beta-1}}{B(\alpha,\beta)}$$

PDF in R

```
(x^(alpha-1)*(1-x)^(beta-1))/beta(alpha,beta)
```

CDF

$$I_x(\alpha,\beta)$$

CDF in R

```
Rbeta(x, alpha, beta)
```

Characteristics

Mean

$$\frac{\alpha}{\alpha+\beta}$$

Median

$$I_{\frac{1}{2}}^{[-1]}(\alpha,\beta)$$

Mode

$$\frac{\alpha-1}{\alpha+\beta-2}$$

Variance

$$\frac{\alpha\beta}{(\alpha+\beta)^2(\alpha+\beta+1)}$$

Relationships

- Relationship pair: $Beta1(alpha, beta) \rightarrow Normal1(\mu, \sigma)$
- Relationship type: Special case & Limiting
- Relationship definition: $\alpha = \beta, \beta \rightarrow \infty$

- Relationship pair: $Gamma1(k, theta) \rightarrow Beta1(alpha, beta)$
- Relationship type: Transformation
- Relationship definition: $X1, X2 \sim Gamma1(k, theta)$ and $Y = X1/(X1 + X2) \Rightarrow Y \sim Beta1(alpha, beta)$

- Relationship pair: $StandardUniform1(0, 1) \rightarrow Beta1(alpha, beta)$
- Relationship type: Transformation
- Relationship definition: $aX_1, X_2, ..., X_n(iid) \sim StandardNormal1 \Rightarrow$ with $\alpha = r$ and $\beta = n-r+1, X_{(r)} \sim Beta(\alpha, \beta)$

References

[Leemis and Mcqueston, 2008]
http://en.wikipedia.org/wiki/Beta_distribution
http://www.uncertml.org/distributions/beta
http://www.math.wm.edu/~leemis/chart/UDR/PDFs/GammaBeta.pdf
http://www.math.wm.edu/~leemis/chart/UDR/PDFs/BetaNormal.pdf
http://www.math.wm.edu/~leemis/chart/UDR/PDFs/StandarduniformBeta.pdf

BetaBinomial1

**name**	Beta-binomial 1 (ID: 0000090)
**type**	discrete
**variate**	k, scalar
**support**	$k \in \{0, \ldots, n\}$

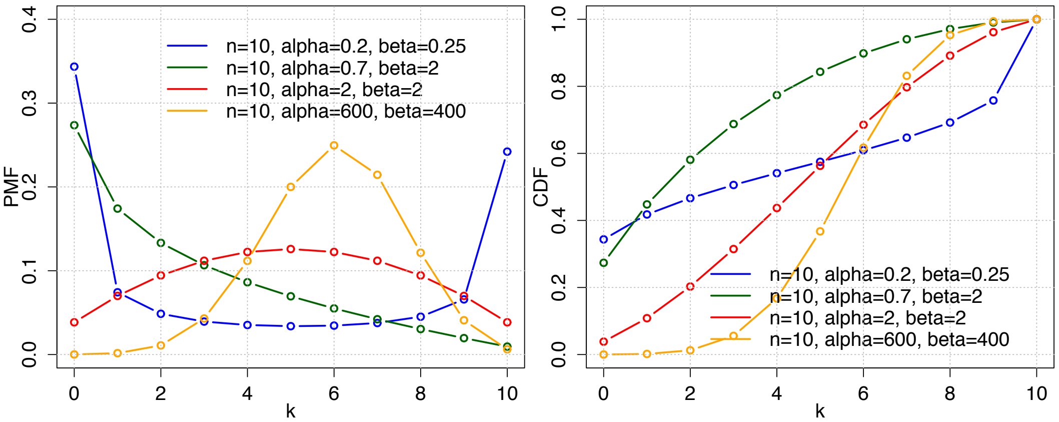

Figure 1.3: BetaBinomial1 distribution plotted using the provided R code.

Model

Beta-binomial distribution is a family of discrete probability distributions on a finite support of non-negative integers arising when the probability of success in each of a fixed or known number of Bernoulli trials is either unknown or random. The beta-binomial distribution is the binomial distribution in which the probability of success at each trial is not fixed but random and follows the beta distribution.

Parameter: numberOfTrials

**name**	number of trials
**type**	scalar
**symbol**	n
**definition**	$n \in N, n > 0$

Parameter: alpha

**name**	left beta parameter
**type**	scalar
**symbol**	α
**definition**	$\alpha > 0$

Parameter: beta

**name**	right beta parameter
**type**	scalar
**symbol**	β
**definition**	$\beta > 0$

Functions

PMF

$$\binom{n}{k} \frac{B(k+\alpha, n-k+\beta)}{B(\alpha,\beta)}$$

PMF in R

```
choose(n,k) * beta(k+alpha,n-k+beta) / beta(alpha,beta)
```

CDF

$$\Sigma_{i=1}^{x} f(i), x \in \{0, 1, 2, ...\} \text{ with } f \text{ the PMF}$$

CDF in R

```
cumsum(PMF)
```

Characteristics

Mean

$$\frac{n\alpha}{\alpha + \beta}$$

Variance

$$\frac{n\alpha\beta(\alpha + \beta + n)}{(\alpha + \beta)^2(\alpha + \beta + 1)}$$

Relationships

- Relationship pair: $BetaBinomial1(n, \alpha, \beta) \rightarrow UniformDiscrete2(n)$
- Relationship type: Special case
- Relationship definition: $\alpha = 1, \beta = 1$

References

[Leemis and Mcqueston, 2008]
`https://en.wikipedia.org/wiki/Beta-binomial_distribution`
`http://www.math.wm.edu/~leemis/chart/UDR/PDFs/BetabinomialRectangular.pdf`

Binomial1

**name**	Binomial 1 (ID: 0000117)
**type**	discrete
**variate**	k, scalar
**support**	$k \in \{0, \dots, n\}$

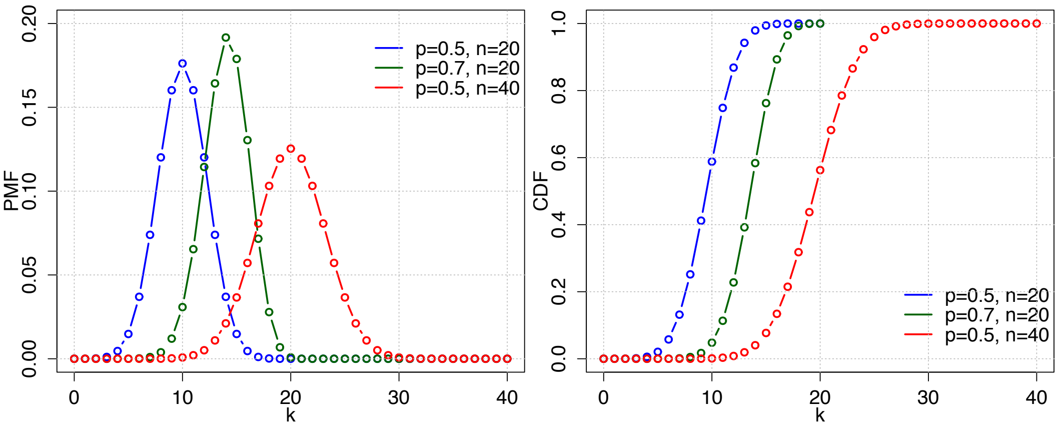

Figure 1.4: Binomial1 distribution plotted using the provided R code.

Model

Binomial distribution with parameters n and p is the probability distribution of the number of successes in a sequence of n independent yes/no experiments (Bernoulli trial) each of which yields success with probability p.

Parameter: numberOfTrials

**name**	number of trials
**type**	scalar
**symbol**	n
**definition**	$n \in N, n \geq 0$

Parameter: probability

**name**	success probability in each trial
**type**	scalar
**symbol**	p
**definition**	$p \in [0, 1]$

Functions

PMF

$$\binom{n}{k} p^k (1-p)^{n-k}$$

PMF in R

```
choose(n,k) * p^k*(1-p)^(n-k)
```

CDF

$$I_{1-p}(n-k, 1+k)$$

CDF in R

```
Rbeta(1-p, n-k, 1+k)
```

Characteristics

Mean

$$np$$

Median

$$\lfloor np \rfloor \text{ or } \lceil np \rceil$$

Mode

$$\lfloor (n+1)p \rfloor \; or \; \lfloor (n+1)p \rfloor - 1$$

Variance

$$np(1-p)$$

Relationships

- Relationship pair: $Binomial1(n, p) \rightarrow Bernoulli1(p)$
- Relationship type: Special case
- Relationship definition: $n = 1$

- Relationship pair: $Binomial1(n, p) \rightarrow Poisson1(\lambda)$
- Relationship type: Reparameterisation & Limiting
- Relationship definition: $\lambda = np, n \rightarrow \infty$

- Relationship pair: $Binomial1(n, p) \rightarrow Normal1(\mu, \sigma)$
- Relationship type: Limiting
- Relationship definition: For $X \sim Binomial1(n, p)$ as $n \rightarrow \infty, X$ is approximately normally distributed $Normal1(\mu, \sigma)$ with $\mu = np, \sigma = np(1-p)$.

- Relationship pair: $ConwayMaxwellPoisson1(\lambda, \nu) \rightarrow Binomial1(p)$
- Relationship type: Transformation
- Relationship definition: For $\nu = \infty$ the distribution of the sum is binomial with parameters n and $\lambda/(1+\lambda)$

- Relationship pair: $Hypergeometric1(N, K, n) \rightarrow Binomial1(n, p)$
- Relationship type: Reparameterisation & Limiting

- Relationship definition: $p = K/N, n = n, N \to \infty$
- Relationship pair: $Bernoulli1(p) \to Binomial1(n, p)$
- Relationship type: Transformation
- Relationship definition: $\Sigma X(iid)$
- Relationship pair: $GeneralizedNegativeBinomial1(\theta, \beta, m) \to Binomial1(n, p)$
- Relationship type: Special case & Reparameterisation
- Relationship definition: $\beta = 0$ and set $m = n, \theta = p$

References

[Leemis and Mcqueston, 2008], [Consul and Famoye, 2006]
https://en.wikipedia.org/wiki/Binomial_distribution
http://www.uncertml.org/distributions/binomial
http://www.math.wm.edu/~leemis/chart/UDR/PDFs/BinomialBernoulli.pdf
http://www.math.wm.edu/~leemis/chart/UDR/PDFs/BinomialPoisson.pdf
http://www.math.wm.edu/~leemis/chart/UDR/PDFs/HypergeometricBinomial.pdf
http://www.math.wm.edu/~leemis/chart/UDR/PDFs/BernoulliBinomial.pdf
http://www.math.wm.edu/~leemis/chart/UDR/PDFs/BinomialNormal.pdf

BirnbaumSaunders1

**name**	Birnbaum-Saunders 1 (ID: 0000177)
**type**	continuous
**variate**	x, scalar
**support**	$x \in (0, +\infty)$

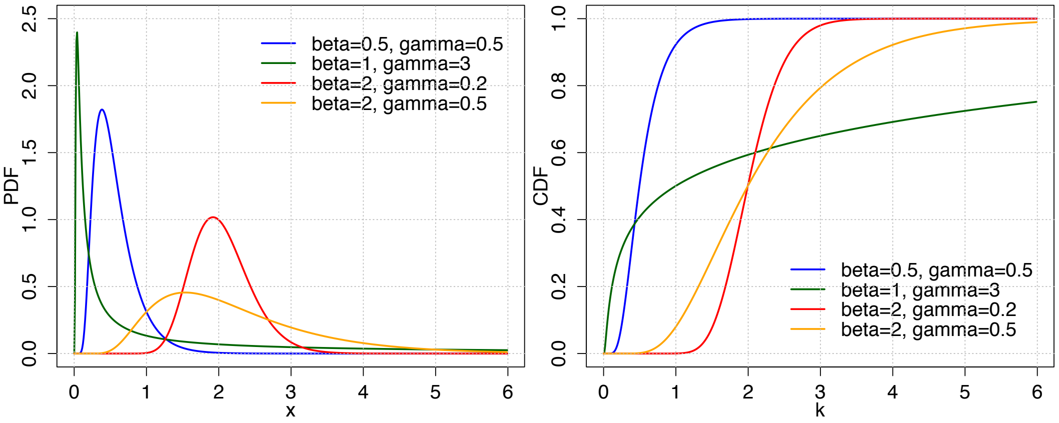

Figure 1.5: BirnbaumSaunders1 distribution plotted using the provided R code.

Parameter: scale

**name**	scale
**type**	scalar
**symbol**	β
**definition**	$\beta > 0$

Parameter: shape

**name**	shape
**type**	scalar
**symbol**	γ
**definition**	$\gamma > 0$

Functions

PDF

$$\frac{1}{\sqrt{2\pi}} \exp\Big[-\frac{(\sqrt{x/\beta}-\sqrt{\beta/x})^2}{2\gamma^2}\Big]\Big[\frac{\sqrt{x/\beta}+\sqrt{\beta/x}}{2\gamma x}\Big]$$

PDF in R

```
1/(sqrt(2*pi))* exp( -(sqrt(x/beta) - sqrt(beta/x))^2 / (2*gamma^2) ) *
( sqrt(x/beta) + sqrt(beta/x) ) / (2*gamma*x)
```

CDF

$$\int_0^x f(x), \text{ with f the PDF}$$

CDF in R

```
cumsum(PDF*rep(stepSize,length(PDF)))
```

References

[MathWorks, 2015]
https://en.wikipedia.org/wiki/Birnbaum%E2%80%93Saunders_distribution

CategoricalNonordered1

**name**	Categorical Nonordered 1 (ID: 0000248)
**type**	discrete
**variate**	x, scalar
**support**	$x \in \{1, \ldots, k\}$

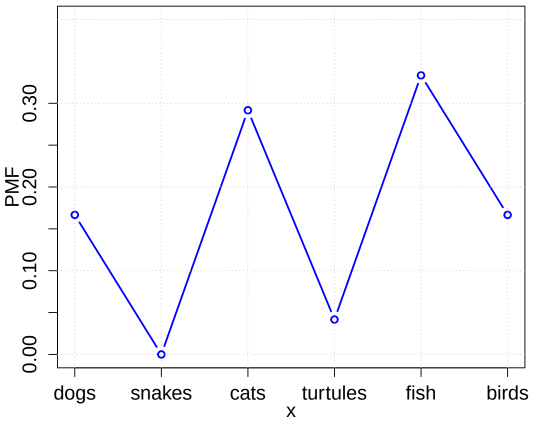

Figure 1.6: CategoricalNonordered1 distribution plotted using the provided R code.

Parameter: categoryProb

**name**	category probabilities
**type**	vector
**symbol**	$p_1, \ldots, p_k$
**definition**	$0 \leq p_i \leq 1, \Sigma p_i = 1$

Functions

PMF

$$p(x = i) = p_i$$

CDF

$$undefined$$

Characteristics

Mean

$$E([x=i]) = p_i, \text{this is the mean of the Iverson bracket } [x=i] \text{ and not the mean of } x$$

Median

$$i \text{ such that } \sum_{j=1}^{i-1} p_j \leq 0.5 \text{ and } \sum_{j=1}^{i} p_j \geq 0.5$$

Mode

$$i \text{ such that } p_i = \max(p_1, \ldots, p_k)$$

Variance

$$Var([x=i]) = p_i(1-p_i)Cov([x=i],[x=j]) = -p_i p_j \quad (i \neq j)$$

References

http://en.wikipedia.org/wiki/Categorical_distribution

CategoricalOrdered1

**name**	Categorical Ordered 1 (ID: 0000224)
**type**	discrete
**variate**	x, scalar
**support**	$x \in \{1, \ldots, k\}$

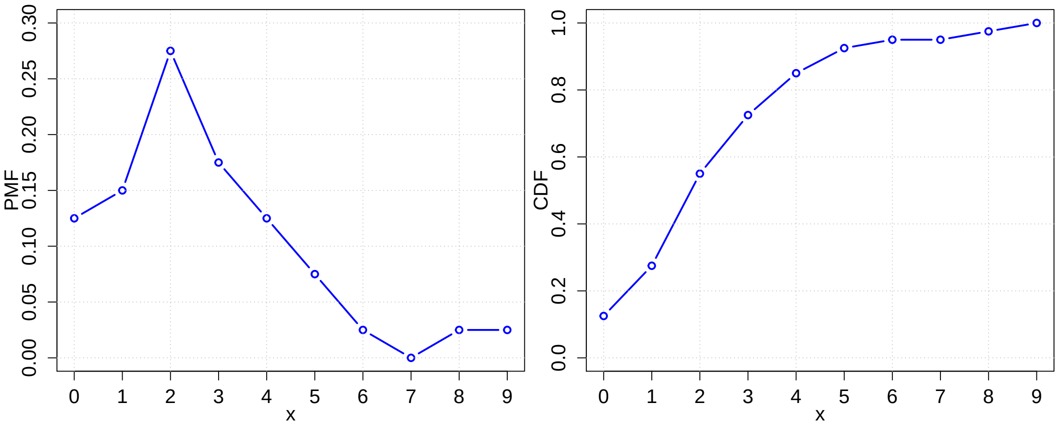

Figure 1.7: CategoricalOrdered1 distribution plotted using the provided R code.

Parameter: categoryProb

**name**	category probabilities
**type**	vector
**symbol**	$p_1, \ldots, p_k$
**definition**	$0 \leq p_i \leq 1, \Sigma p_i = 1$

Functions

PMF

$$p(x=i) = p_i$$

CDF

$$\begin{cases} 0 & \text{for } x < 1 \\ \sum_{j=1}^{i} p_j & \text{for } x \in [i, i+1) \\ 1 & \text{for } x \geq k \end{cases}$$

Characteristics

Mean

$$E([x = i]) = p_i, \text{this is the mean of the Iverson bracket } [x = i] \text{ and not the mean of } x$$

Median

$$i \text{ such that } \sum_{j=1}^{i-1} p_j \leq 0.5 \text{ and } \sum_{j=1}^{i} p_j \geq 0.5$$

Mode

$$i \text{ such that } p_i = \max(p_1, \ldots, p_k)$$

Variance

$$Var([x = i]) = p_i(1 - p_i)Cov([x = i], [x = j]) = -p_i p_j \quad (i \neq j)$$

References

http://en.wikipedia.org/wiki/Categorical_distribution

Cauchy1

**name**	Cauchy 1 (ID: 0000274)
**type**	continuous
**variate**	x, scalar
**support**	$x \in (-\infty, +\infty)$

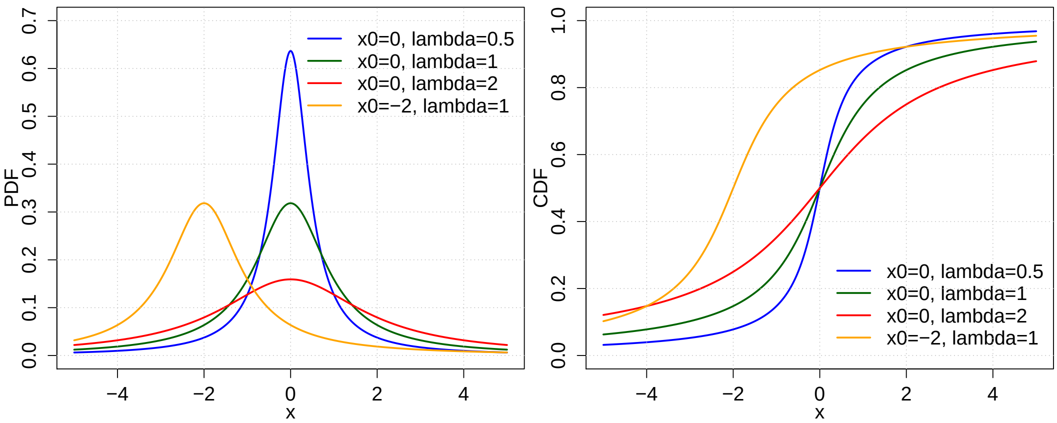

Figure 1.8: Cauchy1 distribution plotted using the provided R code.

Parameter: location

**name**	location
**type**	scalar
**symbol**	x_0
**definition**	$x_0 \in R$

Parameter: scale

**name**	scale
**type**	scalar
**symbol**	γ
**definition**	$\gamma \in R$

Functions

PDF

$$\frac{1}{\pi\gamma\left[1+\left(\frac{x-x_0}{\gamma}\right)^2\right]}$$

PDF in R

```
1 / (pi*gamma*(1 + ((x-x0)^2/gamma^2)))
```

CDF

$$\frac{1}{\pi}\arctan\left(\frac{x-x_0}{\gamma}\right)+\frac{1}{2}$$

CDF in R

```
1/pi * atan((x-x0)/gamma)+1/2
```

Characteristics

Mean

$$undefined$$

Median

$$x_0$$

Mode

$$x_0$$

Variance

$$undefined$$

Refereneces

http://en.wikipedia.org/wiki/Cauchy_distribution
http://www.uncertml.org/distributions/cauchy

ChiSquared1

**name**	Chi-squared 1 (ID: 0000299)
**type**	continuous
**variate**	x, scalar
**support**	$x \in [0, +\infty)$

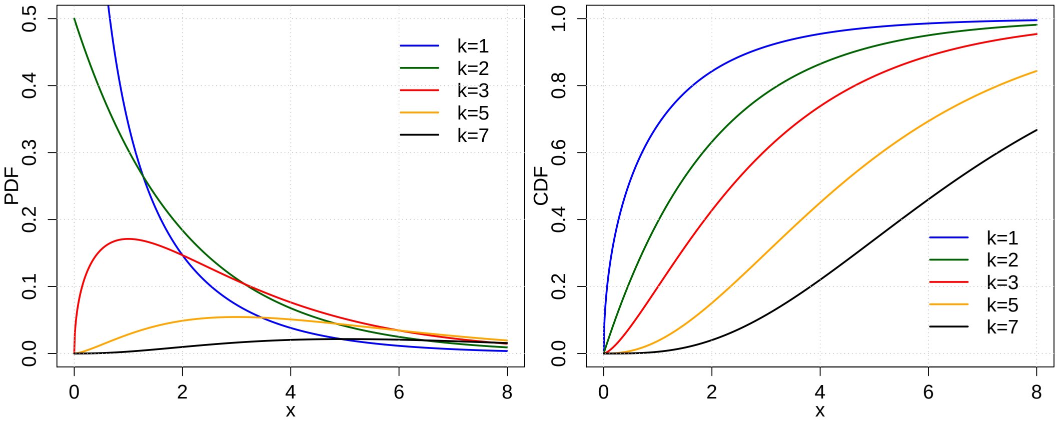

Figure 1.9: ChiSquared1 distribution plotted using the provided R code.

Parameter: degreesOfFreedom

**name**	degrees of freedom
**type**	scalar
**symbol**	k
**definition**	$k \in N$

Functions

PDF

$$\frac{1}{2^{\frac{k}{2}}\Gamma\left(\frac{k}{2}\right)}\, x^{\frac{k}{2}-1}e^{-\frac{x}{2}}$$

PDF in R

```
1/( 2^k/2 * gamma(k/2) ) *  x^(k/2-1) * exp(-x/2)
```

CDF

$$\frac{1}{\Gamma\left(\frac{k}{2}\right)}\,\gamma\left(\frac{k}{2},\frac{x}{2}\right)$$

CDF in R

```
1/gamma(k/2) * Igamma(k/2,x/2)
```

Characteristics

Mean

$$k$$

Median

$$\approx k\left(1-\frac{2}{9k}\right)^3$$

Mode

$$max\{k-2,0\}$$

Variance

$$2k$$

Relationships

- Relationship pair: $ChiSquared1(k) \rightarrow Exponential1(\lambda)$
- Relationship type: Special case
- Relationship definition: $k = 2$ and $\lambda = 1/2$

- Relationship pair: $ChiSquared1(n) \rightarrow F1(n_1, n_2)$
- Relationship type: Transformation
- Relationship definition: If $X_1 \sim ChiSquared1(n_1), X_2 \sim ChiSquared1(n_2)$ are independent random variables $\Rightarrow \frac{X_1/n_1}{X_2/n_2} \sim F1(n_1, n_2)$

- Relationship pair: $GeneralizedGamma2(a, b, c, k) \rightarrow ChiSquared1(k)$
- Relationship type: Special case & Reparameterisation
- Relationship definition: $a = 0, b = 2, c = k_{ChiSquare1}/2, k = 1$

- Relationship pair: $StandardNormal1(0, 1) \rightarrow ChiSquared1(k)$
- Relationship type: Transformation
- Relationship definition: ΣX^2

- Relationship pair: $InverseGaussian1(\lambda, \mu) \rightarrow ChiSquared1(k)$
- Relationship type: Transformation
- Relationship definition: $X \sim InverseGaussian1(\lambda, \mu)$ and $Y = \lambda(X-\mu)^2/(\mu^2 X) \Rightarrow Y \sim ChiSquared1(k)$

- Relationship pair: $Normal1(\mu, \sigma) \rightarrow ChiSquared1(n)$
- Relationship type: Transformation
- Relationship definition: If $X_i \sim N(\mu, \sigma), i = 1, 2, ..., n$ are mutually independent and identically distributed random variables and $Y = \sum_{i=1}^{n}((X_i-\mu)/\sigma)^2 \Rightarrow Y \sim ChiSquared1(n)$

- Relationship pair: $Gamma1(k, \theta) \to ChiSquared1(n)$
- Relationship type: Special case
- Relationship definition: $k_{ChiSquared1} = 2k, \theta = 2$
- Relationship pair: $F1(n_1, n_2) \to ChiSquared1(n)$
- Relationship type: Limiting
- Relationship definition: If $X \sim F1(n_1, n_2)$, the limiting distribution of $n_1 X$ as $n_2 \to \infty$ is the chi-square distribution with n_1 degrees of freedom

References

[Leemis and Mcqueston, 2008]
http://en.wikipedia.org/wiki/Chi-squared_distribution
http://www.uncertml.org/distributions/chi-square
http://www.math.wm.edu/~leemis/chart/UDR/PDFs/StandardnormalChisquare.pdf
http://www.math.wm.edu/~leemis/chart/UDR/PDFs/ChisquareExponential.pdf
http://www.math.wm.edu/~leemis/chart/UDR/PDFs/InversegaussianChisquare.pdf
http://www.math.wm.edu/~leemis/chart/UDR/PDFs/NormalChisquare.pdf
http://www.math.wm.edu/~leemis/chart/UDR/PDFs/GammaChisquareT.pdf
http://www.math.wm.edu/~leemis/chart/UDR/PDFs/ChisquareF.pdf
http://www.math.wm.edu/~leemis/chart/UDR/PDFs/FChisquare.pdf

ConwayMaxwellPoisson1

**name**	Conway-Maxwell-Poisson 1 (ID: 0000323)
**type**	discrete
**variate**	x, scalar
**support**	$x \in \{0, 1, 2, 3, \dots\}$

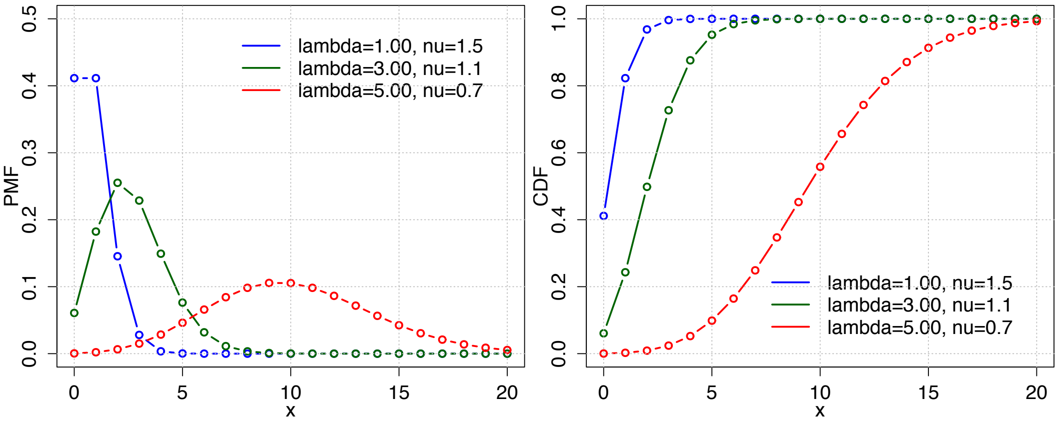

Figure 1.10: ConwayMaxwellPoisson1 distribution plotted using the provided R code.

Parameter: rate

**name**	Poisson intensity
**type**	scalar
**symbol**	λ
**definition**	$\lambda \in R, \lambda > 0$

Parameter: rateOfDecay

**name**	rate of decay
**type**	scalar
**symbol**	ν
**definition**	$\nu \geq 0$

Functions

PMF

$$\frac{\lambda^x}{(x!)^\nu}\frac{1}{Z(\lambda,\nu)} \text{ with } Z(\lambda,\nu)=\sum_{j=0}^{\infty}\frac{\lambda^j}{(j!)^\nu}$$

PMF in R

```
lambda^x/(factorial(x))^nu*1/Z(lambda,nu,n);
Z(lambda,nu,n): for(i in 0:n) { Z=Z+lambda^i/(factorial(i))^nu }}
```

CDF

$$\Sigma_{i=1}^{x} f(i), x \in \{0,1,2,...\} \text{ with } f \text{ the PMF}$$

CDF in R

```
cumsum(PMF)
```

Characteristics

Mean

$$\sum_{j=0}^{\infty}\frac{j\lambda^j}{(j!)^\nu Z(\lambda,\nu)}$$

Median

No closed form

Variance

$$\sum_{j=0}^{\infty}\frac{j^2\lambda^j}{(j!)^\nu Z(\lambda,\nu)} - \text{Mean}^2$$

Relationships

- Relationship pair: $ConwayMaxwellPoisson1(\lambda,\nu) \rightarrow Poisson1(\lambda)$
- Relationship type: Transformation
- Relationship definition: For $\nu = 1$ the sum has a Poisson distribution with parameter $n\lambda$

- Relationship pair: $ConwayMaxwellPoisson1(\lambda,\nu) \rightarrow Binomial1(p)$
- Relationship type: Transformation
- Relationship definition: For $\nu = \infty$ the distribution of the sum is binomial with parameters n and $\lambda/(1+\lambda)$

- Relationship pair: $ConwayMaxwellPoisson1(\lambda,\nu) \rightarrow NegativeBinomial1(r,p)$
- Relationship type: Transformation
- Relationship definition: For $\nu = 0$ and $\lambda < 1$ the sum of Conway-Maxwell-Poisson distributed variables reduces to the sum of geometric variables, which follows a Negative Binomial distribution with parameters n and $1-\lambda$

References

[Shmueli et al., 2005]
https://en.wikipedia.org/wiki/Conway%E2%80%93Maxwell%E2%80%93Poisson_distribution

Dirichlet1

**name**	Dirichlet 1 (ID: 0000345)
**type**	continuous
**variate**	x, vector
**support**	$x_1, \cdots, x_K$ where $x_i \in [0,1]$ *and* $\sum_{i=1}^{K} x_i = 1$

Parameter: concentration

**name**	concentration
**type**	vector
**symbol**	$\alpha_1, \cdots, \alpha_K$
**definition**	$\alpha_1, \cdots, \alpha_K, \alpha_i > 0$

Functions

PDF

$$\frac{1}{B(\alpha)} \prod_{i=1}^{K} x_i^{\alpha_i - 1} \quad \text{where} \quad B(\alpha) = \frac{\prod_{i=1}^{K} \Gamma(\alpha_i)}{\Gamma(\sum_{i=1}^{K} \alpha_i)} \quad \text{where} \quad \alpha = (\alpha_1, \ldots, \alpha_K)$$

Characteristics

Mean

$$E[X_i] = \frac{\alpha_i}{\sum_k \alpha_k}$$

Mode

$$x_i = \frac{\alpha_i - 1}{\sum_{i=1}^{K} \alpha_i - K}, \quad \alpha_i > 1$$

Variance

$$Var[X_i] = \frac{\alpha_i(\alpha_0 - \alpha_i)}{\alpha_0^2(\alpha_0 + 1)} \quad \text{where} \quad \alpha_0 = \sum_{i=1}^{K} \alpha_i Cov[X_i, X_j] = \frac{-\alpha_i \alpha_j}{\alpha_0^2(\alpha_0 + 1)} \quad (i \neq j)$$

References

http://en.wikipedia.org/wiki/Dirichlet_distribution
http://www.uncertml.org/distributions/dirichlet

DoublePoisson1

**name**	Double Poisson 1 (ID: 0000370)
**type**	discrete
**variate**	x, scalar
**support**	$x \in \{1, 2, 3, \ldots, n\}$

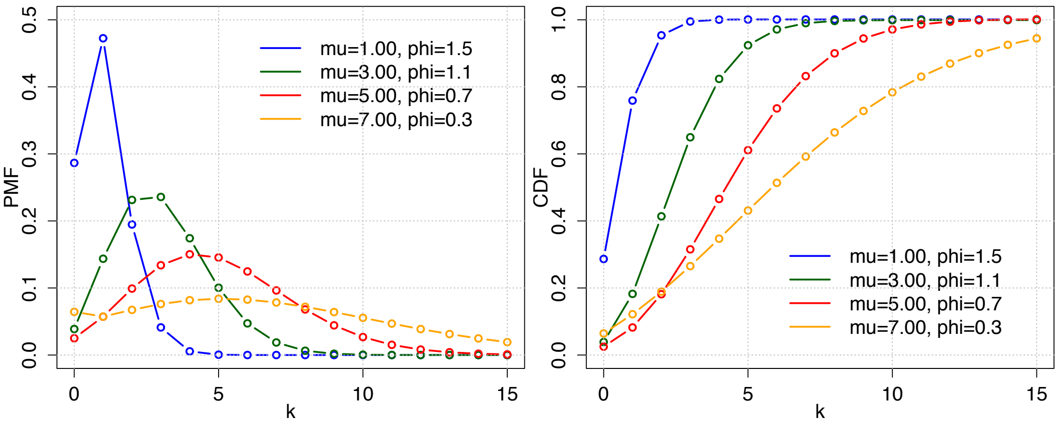

Figure 1.11: DoublePoisson1 distribution plotted using the provided R code.

Parameter: rate

**name**	Poisson intensity
**type**	scalar
**symbol**	μ
**definition**	$\mu \in R, \mu > 0$

Parameter: dispersion

**name**	dispersion
**type**	scalar
**symbol**	ϕ
**definition**	$\phi \in R$

Functions

PMF

$$K(\mu,\phi)\phi^{1/2}\exp(-\phi\mu)\frac{\exp(-y)y^y}{y!}\left(\frac{e\mu}{y}\right)^{\phi y} \text{ with } \frac{1}{K(\mu,\phi)} \approx 1+\frac{1-\phi}{12\phi\mu}\left(1+\frac{1}{\phi\mu}\right)$$

PMF in R

```
K(mu,phi) *phi^(1/2) * exp(-phi*mu) * (exp(-y)*y^y)/factorial(y) * (exp(1)*mu/y)^(phi*y) \\
\text{ with } K(mu,phi) = 1 + (1-phi)/(12*mu*phi)*(1 + 1/(mu*phi))
```

CDF

$$\Sigma_{i=1}^{x} f(i), x \in \{0,1,2,...\} \text{ with } f \text{ the PMF}$$

CDF in R

```
cumsum(PMF)
```

Characteristics

Mean

$$\mu$$

Variance

$$\mu/\phi$$

Relationships

- Relationship pair: $DoublePoisson1(\mu,\phi) \to Poisson1(\lambda)$
- Relationship type: Special case
- Relationship definition: $\phi = 1$

References

[Cameron and Trivedi, 2013]
http://support.sas.com/resources/papers/proceedings09/250-2009.pdf

Erlang1

**name**	Erlang 1 (ID: 0000392)
**type**	continuous
**variate**	x, scalar
**support**	$x \in [0,+\infty)$ (Forbes), $x \in (0,+\infty)$ (Leemis)

Parameter: scale

**name**	scale
**type**	scalar
**symbol**	b
**definition**	$b > 0$

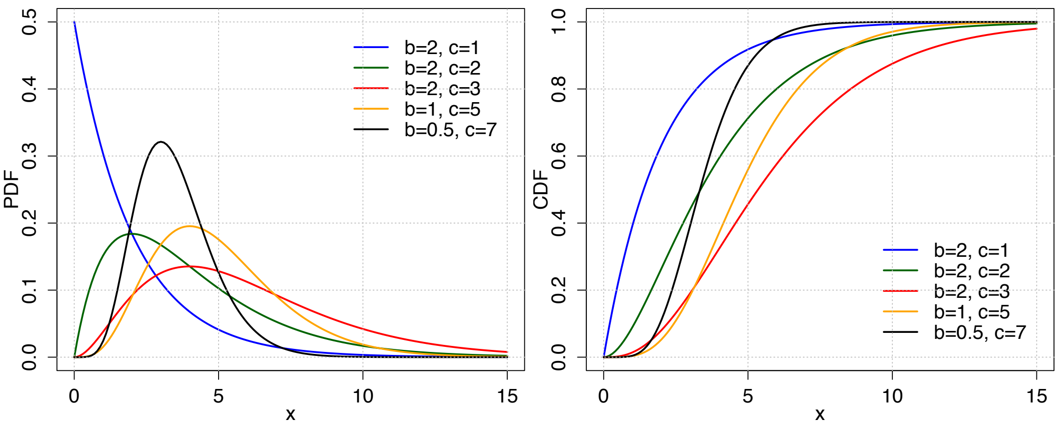

Figure 1.12: Erlang1 distribution plotted using the provided R code.

Parameter: shape

**name**	shape
**type**	scalar
**symbol**	c
**definition**	$c > 0$

Functions

PDF

$$\frac{(x/b)^{c-1}\exp(-x/b)}{b(c-1)!}$$

PDF in R

```
((x/b)^(c-1) *exp(-x/b)) / (b * factorial(c-1))
```

CDF

$$1 - \left[\exp\left(-\frac{x}{b}\right)\right]\left(\sum_{i=0}^{c-1}\frac{(x/b)^i}{i!}\right)$$

CDF in R

```
R function
```

Characteristics

Mean

$$bc$$

Mode

$$b(c-1), c \geq 1$$

Variance

$$b^2c$$

Relationships

- Relationship pair: $Erlang1(b,c) \rightarrow Exponential2(\beta)$
- Relationship type: Special case
- Relationship definition: $c = 1, b = \beta$

- Relationship pair: $Gamma1(k,\theta) \rightarrow Erlang1(b,c)$
- Relationship type: Special case
- Relationship definition: $k \in N, k = c, \theta = b$

References

[Forbes et al., 2011], [Leemis and Mcqueston, 2008]
`http://www.math.wm.edu/~leemis/chart/UDR/PDFs/GammaErlang.pdf`
`http://www.math.wm.edu/~leemis/chart/UDR/PDFs/Erlang.pdf`

Exponential1

**name**	Exponential 1 (ID: 0000418)
**type**	continuous
**variate**	x, scalar
**support**	$x \in [0, +\infty)$

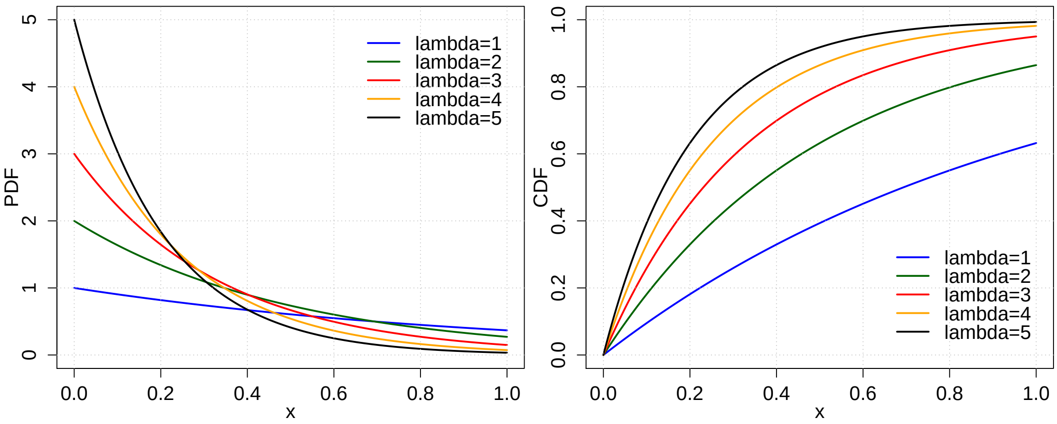

Figure 1.13: Exponential1 distribution plotted using the provided R code.

Parameter: rate

**name**	rate or inverse scale
**type**	scalar
**symbol**	λ
**definition**	$\lambda > 0$

Functions

PDF

$$\lambda e^{-\lambda x}$$

PDF in R

```
lambda*exp(-lambda*x)
```

CDF

$$1 - \exp(-\lambda x)$$

CDF in R

```
1 - exp(-lambda*x)
```

Characteristics

Mean

$$\lambda^{-1}$$

Median

$$\lambda^{-1} \ln(2)$$

Mode

$$0$$

Variance

$$\lambda^{-2}$$

Relationships

- Relationship pair: $Exponential1(\lambda) \rightarrow Exponential2(\beta)$
- Relationship type: Reparameterisation
- Relationship definition: $\beta = 1/\lambda$

- Relationship pair: $Pareto1(x_m, \alpha) \rightarrow Exponential1(\lambda)$
- Relationship type: Transformation
- Relationship definition: $X \sim Pareto1, Y = \log(X/\lambda) \Rightarrow Y \sim Exponential1$

- Relationship pair: $GeneralizedGamma2(a, b, c, k) \rightarrow Exponential1(\lambda)$
- Relationship type: Special case & Reparameterisation
- Relationship definition: $k = c = 1, a = 0, b = 1/\lambda$

- Relationship pair: $Gamma1(k, \theta) \rightarrow Exponential1(\lambda)$
- Relationship type: Special case & Reparameterisation
- Relationship definition: $k = 1, \theta = 1/\lambda$

- Relationship pair: $Weibull1(\lambda, k) \rightarrow Exponential1(\lambda_{Exponetial})$
- Relationship type: Special case & Reparameterisation
- Relationship definition: $k = 1, \lambda_{Exponetial} = 1/\lambda$

- Relationship pair: $ChiSquared1(k) \rightarrow Exponential1(\lambda)$
- Relationship type: Special case
- Relationship definition: $k = 2$ and $\lambda = 1/2$

- Relationship pair: $StandardUniform1(0, 1) \rightarrow Exponential1(\lambda)$
- Relationship type: Transformation
- Relationship definition: $-\frac{1}{\lambda}\log(X)$

- Relationship pair: $Exponential2(\beta) \rightarrow Exponential1(\lambda)$
- Relationship type: Reparameterisation
- Relationship definition: $\lambda = 1/\beta$

References

[Leemis and Mcqueston, 2008], [Forbes et al., 2011]
`http://en.wikipedia.org/wiki/Exponential_distribution`
`http://www.uncertml.org/distributions/exponential`
`http://www.math.wm.edu/~leemis/chart/UDR/PDFs/ChisquareExponential.pdf`
`http://www.math.wm.edu/~leemis/chart/UDR/PDFs/StandarduniformExponentialB.pdf`
`http://www.math.wm.edu/~leemis/chart/UDR/PDFs/ParetoExponential.pdf`

Exponential2

**name**	Exponential 2 (ID: 0000443)
**type**	continuous
**variate**	x, scalar
**support**	$x \in [0, +\infty)$

Parameter: mean

**name**	scale
**type**	scalar
**symbol**	β
**definition**	$\beta > 0$

Functions

PDF

$$1/\beta e^{-x/\beta}$$

PDF in R

```
1/beta*exp(-1/beta*x)
```

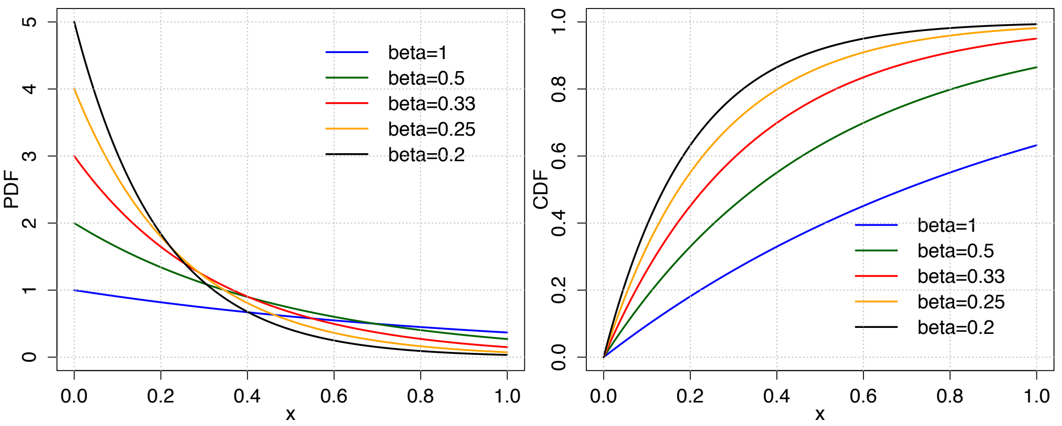

Figure 1.14: Exponential2 distribution plotted using the provided R code.

CDF

$$1 - \exp(-x/\beta)$$

CDF in R

```
1 - exp(-x/beta)
```

Characteristics

Mean

$$\beta$$

Median

$$\lambda^{-1}\ln(2)$$

Mode

$$0$$

Variance

$$\beta^2$$

Relationships

- Relationship pair: $Exponential2(\beta) \rightarrow Exponential1(\lambda)$
- Relationship type: Reparameterisation
- Relationship definition: $\lambda = 1/\beta$

- Relationship pair: $Exponential2(1) \rightarrow F1(n_1, n_2)$
- Relationship type: Transformation
- Relationship definition: If $X_1, X_2 \sim Exponential2(1)$ mutually independent and identically distributed random variables $\Rightarrow X_1/X_2$ has the $F1$ distribution

- Relationship pair: $Exponential1(\lambda) \rightarrow Exponential2(\beta)$
- Relationship type: Reparameterisation
- Relationship definition: $\beta = 1/\lambda$

- Relationship pair: $Erlang1(b, c) \rightarrow Exponential2(\beta)$
- Relationship type: Special case
- Relationship definition: $c = 1, b = \beta$

References

[Forbes et al., 2011], [Leemis and Mcqueston, 2008]
`http://www.itl.nist.gov/div898/handbook/eda/section3/eda3667.htm`
`http://www.math.wm.edu/~leemis/chart/UDR/PDFs/Exponential.pdf`
`http://www.math.wm.edu/~leemis/chart/UDR/PDFs/ExponentialF.pdf`

F1

**name**	F 1 (ID: 0000492)
**type**	continuous
**variate**	x, scalar
**support**	$x \in [0, +\infty)$

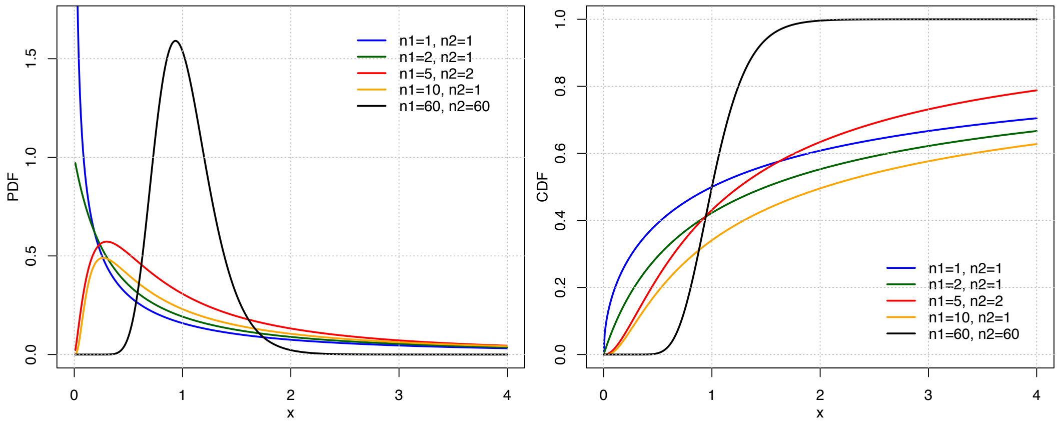

Figure 1.15: F1 distribution plotted using the provided R code.

Parameter: numerator

**name**	degree of freedom
**type**	scalar
**symbol**	n_1
**definition**	$n_1 > 0$

Parameter: denominator

**name**	degree of freedom
**type**	scalar
**symbol**	n_2
**definition**	$n_2 > 0$

Functions

PDF

$$\frac{\Gamma(\frac{n_1+n_2}{2})(\frac{n_1}{n_2})^{n_1/2}x^{n_1/2-1}}{\Gamma(\frac{n_1}{2})\Gamma(\frac{n_2}{2})\left[\frac{n_1}{n_2}x+1\right]^{(n_1+n_2)/2}}$$

PDF in R

```
gamma((n1 + n2)/2)*(n1/n2)^(n1/2)*x^(n1/2-1)/(gamma(n1/2)*gamma(n2/2)*(n1/n2*x+1)^((n1+n2)/2))
```

CDF

$$I_{\frac{n_1 x}{n_1 x+n_2}}\left(\frac{n_1}{2}, \frac{n_2}{2}\right)$$

CDF in R

```
Rbeta(n1*x / (n1*x + n2), n1/2, n2/2)
```

Characteristics

Mean

$$n_2/(n_2-2), n_2>2$$

Mode

$$\frac{n_2(n_1-2)}{n_1(n_2+2)}, n_1>2$$

Variance

$$\frac{2n_2^2(n_1+n_2-2)}{n_1(n_2-2)^2(n_2-4)}, n_2>4$$

Relationships

- Relationship pair: $F1(n_1,n_2) \to ChiSquared1(n)$
- Relationship type: Limiting
- Relationship definition: If $X \sim F1(n_1,n_2)$, the limiting distribution of n_1X as $n_2 \to \infty$ is the chi-square distribution with n_1 degrees of freedom

- Relationship pair: $ChiSquared1(n) \to F1(n_1,n_2)$
- Relationship type: Transformation
- Relationship definition: If $X_1 \sim ChiSquared1(n_1), X_2 \sim ChiSquared1(n_2)$ are independent random variables $\Rightarrow \frac{X_1/n_1}{X_2/n_2} \sim F1(n_1,n_2)$

- Relationship pair: $Exponential2(1) \to F1(n_1,n_2)$
- Relationship type: Transformation
- Relationship definition: If $X_1, X_2 \sim Exponential2(1)$ mutually independent and identically distributed random variables $\Rightarrow X_1/X_2$ has the $F1$ distribution

- Relationship pair: $StudentT1(\nu) \to F1(n_1,n_2)$
- Relationship type: Transformation
- Relationship definition: If $X \sim StudentT1(\nu) \Rightarrow Y = X^2 \sim F(1,\nu)$

References

[Leemis and Mcqueston, 2008], [Forbes et al., 2011]
http://www.math.wm.edu/~leemis/chart/UDR/PDFs/F.pdf
http://www.uncertml.org/distributions/f
http://www.math.wm.edu/~leemis/chart/UDR/PDFs/ChisquareF.pdf
http://www.math.wm.edu/~leemis/chart/UDR/PDFs/FChisquare.pdf
http://www.math.wm.edu/~leemis/chart/UDR/PDFs/ExponentialF.pdf
http://www.math.wm.edu/~leemis/chart/UDR/PDFs/TF.pdf

Gamma1

**name**	Gamma 1 (ID: 0000571)
**type**	continuous
**variate**	x, scalar
**support**	$x \in (0, +\infty)$

Parameter: shape

**name**	shape
**type**	scalar
**symbol**	k
**definition**	$k > 0$

Parameter: scale

**name**	scale
**type**	scalar
**symbol**	θ
**definition**	$\theta > 0$

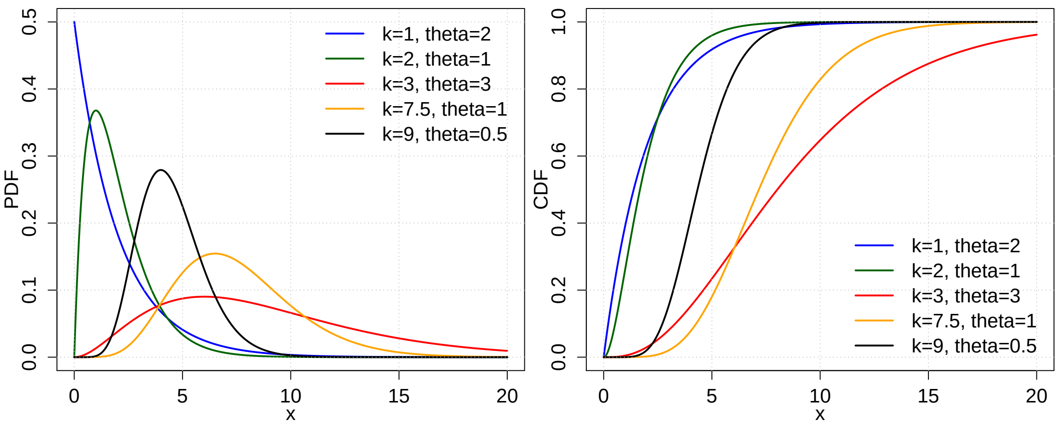

Figure 1.16: Gamma1 distribution plotted using the provided R code.

Functions

PDF

$$\frac{1}{\Gamma(k)\theta^k}x^{k-1}e^{-\frac{x}{\theta}}$$

PDF in R

```
1 / (gamma(k) * theta^k) * x^(k-1) * exp(-x/theta)
```

CDF

$$\frac{1}{\Gamma(k)}\gamma\left(k,\ \frac{x}{\theta}\right)$$

CDF in R

```
1/gamma(k) * Igamma(k,x/theta)
```

Characteristics

Mean

$$k\theta$$

Median

No simple closed form

Mode

$$(k - 1)\theta \text{ for } k \geq 1$$

Variance

$$k\theta^2$$

Relationships

- Relationship pair: $Gamma1(k, \theta) \rightarrow Exponential1(\lambda)$
- Relationship type:
- Relationship definition: $k = 1, \theta = 1/\lambda$

- Relationship pair: $Gamma1(\alpha, \beta) \rightarrow InverseGamma1(\alpha, \beta)$
- Relationship type: Transformation
- Relationship definition: If $X \sim Gamma1(\alpha, \beta)$ then $X^{-1} \sim InverseGamma1(\alpha, \beta^{-1})$

- Relationship pair: $Gamma1(k, theta) \rightarrow Gamma2(r, mu)$
- Relationship type: Reparameterisation
- Relationship definition: $r = k, \mu = 1/\theta$

- Relationship pair: $Gamma1(k, theta) \rightarrow Beta1(alpha, beta)$
- Relationship type: Transformation
- Relationship definition: $X1, X2 \sim Gamma1(k, theta)$ and $Y = X1/(X1 + X2) \Rightarrow Y \sim Beta1(alpha, beta)$

- Relationship pair: $Gamma1(k, theta) \to Normal1(\mu, \sigma)$
- Relationship type: Special case & Limiting
- Relationship definition: $\mu = k\theta, \sigma^2 = k^2\theta, \theta \to \infty$

- Relationship pair: $Gamma1(k, \theta) \to Erlang1(b, c)$
- Relationship type: Special case
- Relationship definition: $k \in N, k = c, \theta = b$

- Relationship pair: $Gamma1(k, \theta) \to ChiSquared1(n)$
- Relationship type: Special case
- Relationship definition: $k_{ChiSquared1} = 2k, \theta = 2$

- Relationship pair: $GeneralizedGamma2(a, b, c, k) \to Gamma1(k, \theta)$
- Relationship type: Transformation
- Relationship definition: $k = 1, a = 0$ and renaming parameters: $c = k, b = \theta$

- Relationship pair: $GeneralizedGamma1(a, d, p) \to Gamma1(k, \theta)$
- Relationship type: Special case
- Relationship definition: $p = 1, k = d, \theta = a$

- Relationship pair: $Gamma2(r, mu) \to Gamma1(k, theta)$
- Relationship type: Reparameterisation
- Relationship definition: $k = r, \theta = 1/\mu$

References

[Leemis and Mcqueston, 2008], [Forbes et al., 2011]
`http://en.wikipedia.org/wiki/Gamma_distribution`
`http://www.uncertml.org/distributions/gamma`
`http://www.math.wm.edu/~leemis/chart/UDR/PDFs/GammaBeta.pdf`
`http://www.math.wm.edu/~leemis/chart/UDR/PDFs/GammaNormal1.pdf`
`http://www.math.wm.edu/~leemis/chart/UDR/PDFs/GammaErlang.pdf`
`http://www.math.wm.edu/~leemis/chart/UDR/PDFs/GammaChisquareT.pdf`

Gamma2

**name**	Gamma 2 (ID: 0000597)
**type**	continuous
**variate**	x, scalar
**support**	$x \in (0, +\infty)$

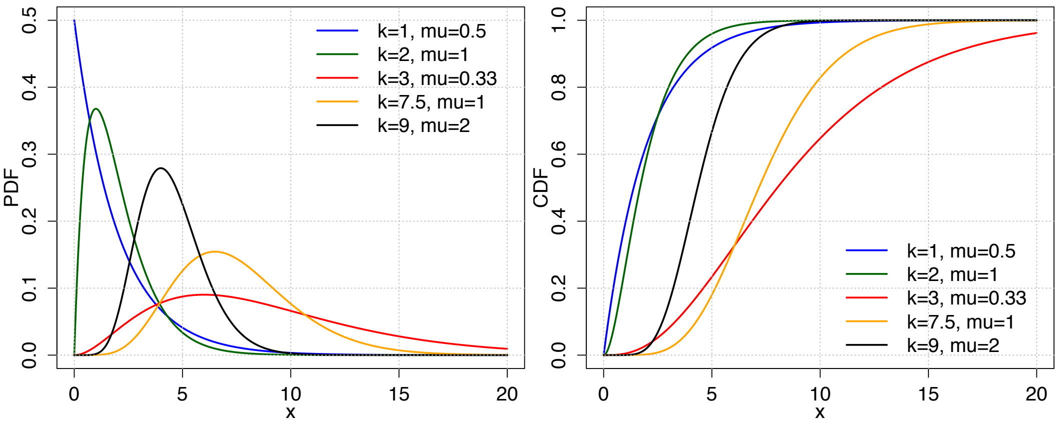

Figure 1.17: Gamma2 distribution plotted using the provided R code.

Parameter: shape

**name**	shape
**type**	scalar
**symbol**	r
**definition**	$r > 0$

Parameter: rate

**name**	rate
**type**	scalar
**symbol**	μ
**definition**	$\mu > 0$

Functions

PDF

$$\frac{\mu^r x^{r-1} e^{-\mu x}}{\Gamma(r)}$$

PDF in R

```
(mu^r * x^(r-1) * exp(-mu*x)) / gamma(r)
```

CDF

$$\frac{1}{\Gamma(r)} \gamma\left(r, \mu x\right)$$

CDF in R

```
1/gamma(r) * Igamma(r,mu*x,lower=T)
```

Characteristics

Mean

$$r/\mu$$

Variance

$$r/\mu^2$$

Relationships

- Relationship pair: $Gamma2(r, mu) \rightarrow Gamma1(k, theta)$
- Relationship type: Reparameterisation
- Relationship definition: $k = r, \theta = 1/\mu$

- Relationship pair: $Gamma1(k, theta) \rightarrow Gamma2(r, mu)$
- Relationship type: Reparameterisation
- Relationship definition: $r = k, \mu = 1/\theta$

References

[Spiegelhalter et al., 2003]

GeneralizedGamma1

**name**	Generalized Gamma 1 (ID: 0000621)
**type**	continuous
**variate**	x, scalar
**support**	$x \in (0, +\infty)$

Parameter: scale

**name**	scale
**type**	scalar
**symbol**	a
**definition**	$a > 0$

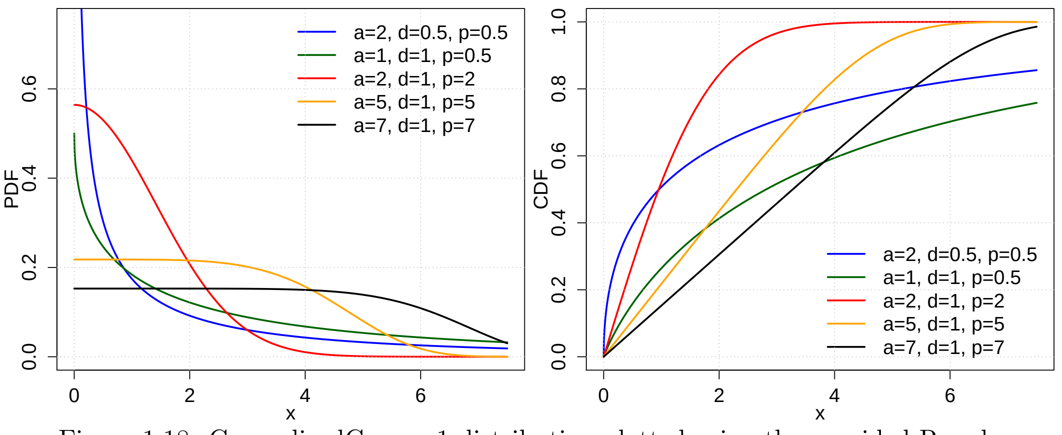

Figure 1.18: GeneralizedGamma1 distribution plotted using the provided R code.

Parameter: shape1

**name**	shape
**type**	scalar
**symbol**	d
**definition**	$d > 0$

Parameter: shape2

**name**	shape
**type**	scalar
**symbol**	p
**definition**	$p > 0$

Functions

PDF

$$\frac{p/a^d}{\Gamma(d/p)} x^{d-1} e^{-(x/a)^p}$$

PDF in R

```
p/a^d/gamma(d/p) * x^(d-1) * exp(-(x/a)^p)
```

CDF

$$\frac{\gamma(d/p,(x/a)^p)}{\Gamma(d/p)}$$

CDF in R

```
Igamma(d/p, (x/a)^p, lower=T) / gamma(d/p)
```

Characteristics

Mean

$$a \frac{\Gamma((d+1)/p)}{\Gamma(d/p)}$$

Mode

$$a \left(\frac{d-1}{p}\right)^{\frac{1}{p}}, \text{ for } d > 1$$

Variance

$$a^2 \left(\frac{\Gamma((d+2)/p)}{\Gamma(d/p)} - \left(\frac{\Gamma((d+1)/p)}{\Gamma(d/p)}\right)^2\right)$$

Relationships

- Relationship pair: $GeneralizedGamma1(a, d, p) \to Gamma1(k, \theta)$
- Relationship type: Special case
- Relationship definition: $p = 1, k = d, \theta = a$

- Relationship pair: $GeneralizedGamma1(a, d, p) \to GeneralizedGamma3(r, \mu, \beta)$
- Relationship type: Reparameterisation
- Relationship definition: $r = d/p, \beta = p, \mu = 1/a$

- Relationship pair: $GeneralizedGamma3(r, \mu, \beta) \to GeneralizedGamma1(a, d, p)$
- Relationship type: Reparameterisation
- Relationship definition: $a = 1/\mu, d = \beta r, p = \beta$

- Relationship pair: $GeneralizedGamma2(a, b, c) \to GeneralizedGamma1(a, d, p)$
- Relationship type: Reparameterisation
- Relationship definition: $a = 0, kc = d,$ and rename $k = p, b = a$

References

[Stacy, 1962]
`http://en.wikipedia.org/wiki/Generalized_gamma_distribution`

GeneralizedGamma2

**name**	Generalized Gamma 2 (ID: 0000644)
**type**	continuous
**variate**	x, scalar
**support**	$0 < a < x$

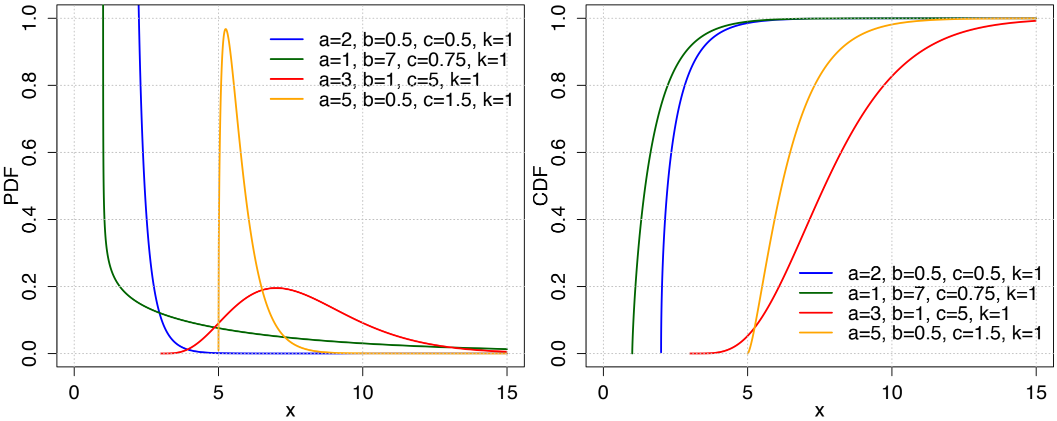

Figure 1.19: GeneralizedGamma2 distribution plotted using the provided R code.

Parameter: location

**name**	location
**type**	scalar
**symbol**	a
**definition**	$a > 0$

Parameter: scale

**name**	scale
**type**	scalar
**symbol**	b
**definition**	$b > 0$

Parameter: shape1

**name**	shape1
**type**	scalar
**symbol**	c
**definition**	$c > 0$

Parameter: shape2

**name**	shape2
**type**	scalar
**symbol**	k
**definition**	$k > 0$

Functions

PDF

$$\frac{k(x-a)^{kc-1}}{b^{kc}\Gamma(c)} \exp\Big[-\Big(\frac{x-a}{b}\Big)^k\Big]$$

PDF in R

```
(k*(x-a)^(k*c-1)) / (b^(k*c)*gamma(c)) * exp( -((x-a)/b)^k )
```

CDF

$$\frac{\gamma(c, (\frac{x-a}{b})^k)}{\Gamma(c)}$$

CDF in R

```
Igamma(c, ((x-a)/b)^k, lower=T) / gamma(c)
```

Characteristics

Mean

$$a + b\Gamma(c + 1/k)/\Gamma(c)$$

Mode

$$a + b(c - 1/k)^{1/k}, c > 1/k$$

Variance

$$b^2\{\Gamma(c + 2/k)/\Gamma(c) - [\Gamma(c + 1/k)/\Gamma(c)]^2\}$$

Relationships

- Relationship pair: $GeneralizedGamma2(a, b, c, k) \rightarrow Gamma1(k, \theta)$
- Relationship type: Transformation
- Relationship definition: $k = 1, a = 0$ and renaming parameters: $c = k, b = \theta$

- Relationship pair: $GeneralizedGamma2(a, b, c, k) \rightarrow Exponential1(\lambda)$
- Relationship type: Special case & Reparameterisation
- Relationship definition: $k = c = 1, a = 0, b = 1/\lambda$

- Relationship pair: $GeneralizedGamma2(a, b, c, k) \rightarrow Weibull1(\lambda, k)$
- Relationship type: Special case & Reparameterisation
- Relationship definition: $c = 1, a = 0, b = \lambda$

- Relationship pair: $GeneralizedGamma2(a, b, c, k) \rightarrow ChiSquared1(k)$
- Relationship type: Special case & Reparameterisation
- Relationship definition: $a = 0, b = 2, c = k_{ChiSquare1}/2, k = 1$

- Relationship pair: $GeneralizedGamma2(a, b, c) \rightarrow GeneralizedGamma1(a, d, p)$
- Relationship type: Reparameterisation
- Relationship definition: $a = 0, kc = d,$ and rename $k = p, b = a$

References

[Forbes et al., 2011]
`http://www.mathwave.com/help/easyfit/html/analyses/distributions/gen_gamma.html`

GeneralizedGamma3

**name**	Generalized Gamma 3 (ID: 0000670)
**type**	continuous
**variate**	x, scalar
**support**	$x \in (0, +\infty)$

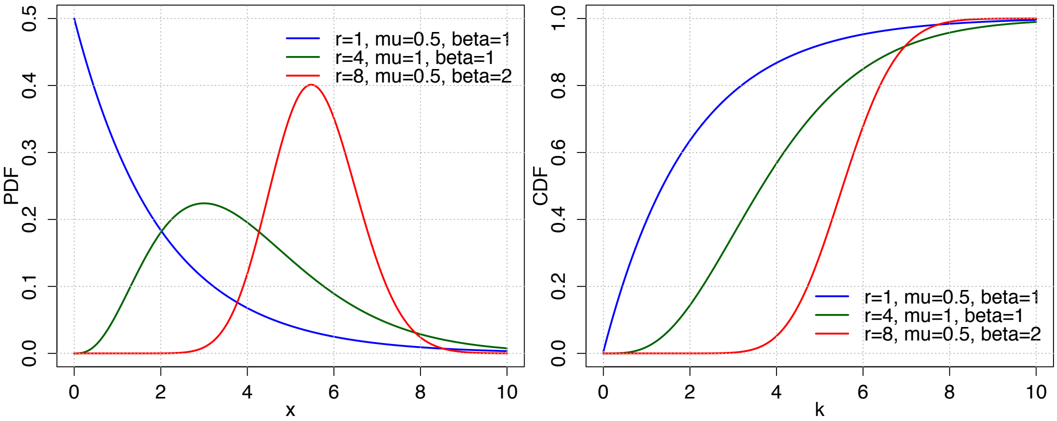

Figure 1.20: GeneralizedGamma3 distribution plotted using the provided R code.

Parameter: scale

**name**	scale
**type**	scalar
**symbol**	r
**definition**	$r > 0$

Parameter: shape1

**name**	shape
**type**	scalar
**symbol**	μ
**definition**	$\mu > 0$

Parameter: shape2

**name**	shape
**type**	scalar
**symbol**	β
**definition**	$\beta > 0$

Functions

PDF

$$\frac{\beta}{\Gamma(r)} \mu^{\beta r} x^{\beta r - 1} \exp[-(\mu x)^{\beta}]$$

PDF in R

```
beta / gamma(r) * mu^(beta*r) * x^(beta*r -1) * exp(-(mu*x)^beta)
```

Relationships

- Relationship pair: $GeneralizedGamma3(r, \mu, \beta) \rightarrow GeneralizedGamma1(a, d, p)$
- Relationship type: Reparameterisation
- Relationship definition: $a = 1/\mu, d = \beta r, p = \beta$

- Relationship pair: $GeneralizedGamma1(a, d, p) \rightarrow GeneralizedGamma3(r, \mu, \beta)$
- Relationship type: Reparameterisation
- Relationship definition: $r = d/p, \beta = p, \mu = 1/a$

References

[Spiegelhalter et al., 2003]

GeneralizedNegativeBinomial1

**name**	Generalized Negative Binomial 1 (ID: 0000690)
**type**	discrete
**variate**	x, scalar
**support**	$x \in \{0, 1, 2, 3, \dots\}$

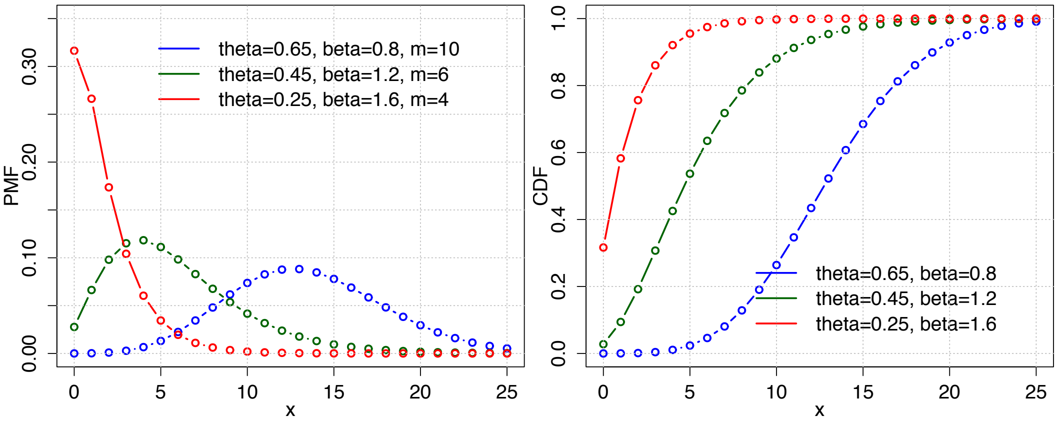

Figure 1.21: GeneralizedNegativeBinomial1 distribution plotted using the provided R code.

Parameter: theta

**name**	scale
**type**	scalar
**symbol**	θ
**definition**	$0 < \theta < 1$

Parameter: beta

**name**	shape1
**type**	scalar
**symbol**	β
**definition**	$\beta = 0$ or $1 \leq \beta \leq \theta^{-1}$

Parameter: m

**name**	shape2
**type**	scalar
**symbol**	m
**definition**	$m > 0$

Functions

PMF

$$\frac{m}{m+\beta x}\binom{m+\beta x}{x}\theta^x(1-\theta)^{m+\beta x-x}$$

PMF in R

```
m/(m+beta*x) * choose(m+beta*x,x)*theta^x * (1-theta)^(m+beta*x-x)
```

CDF

$$\Sigma_{i=1}^{x} f(i), x \in \{0, 1, 2, ...\} \text{ with } f \text{ the PMF}$$

CDF in R

```
cumsum(PMF)
```

Characteristics

Mean

$$m\theta(1-\theta\beta)^{-1}$$

Variance

$$m\theta(1-\theta)(1-\theta\beta)^{-3}$$

Relationships

- Relationship pair: $GeneralizedNegativeBinomial1(\theta, \beta, m) \rightarrow Binomial1(n, p)$
- Relationship type: Special case & Reparameterisation
- Relationship definition: $\beta = 0$ and set $m = n, \theta = p$

- Relationship pair: $GeneralizedNegativeBinomial1(\theta, \beta, m) \rightarrow NegativeBinomial4(r, p)$
- Relationship type: Special case & Reparameterisation
- Relationship definition: $\beta = 1$ and set $m = r, \theta = p$

- Relationship pair: $GeneralizedNegativeBinomial1(\theta, \beta, m) \rightarrow InverseBinomial1(k, p)$
- Relationship type: Special case & Reparameterisation
- Relationship definition: $\beta = 2, \theta = 1 - p$

References

[Consul and Famoye, 2006], [Yanagimoto, 1989]

GeneralizedPoisson1

**name**	Generalized Poisson 1 (ID: 0000712)
**type**	discrete
**variate**	k, scalar
**support**	$k \in \{0, 1, 2, 3, \ldots\}$

Parameter: rate

**name**	Poisson intensity
**type**	scalar
**symbol**	θ
**definition**	$\theta > 0$

Parameter: dispersion

**name**	dispersion
**type**	scalar
**symbol**	δ
**definition**	$max(-1, -\theta/m) < \delta < 1$ with $m(\geq 4)$ the largest positive integer for which $\theta + m\delta > 0$ when $\delta < 0$

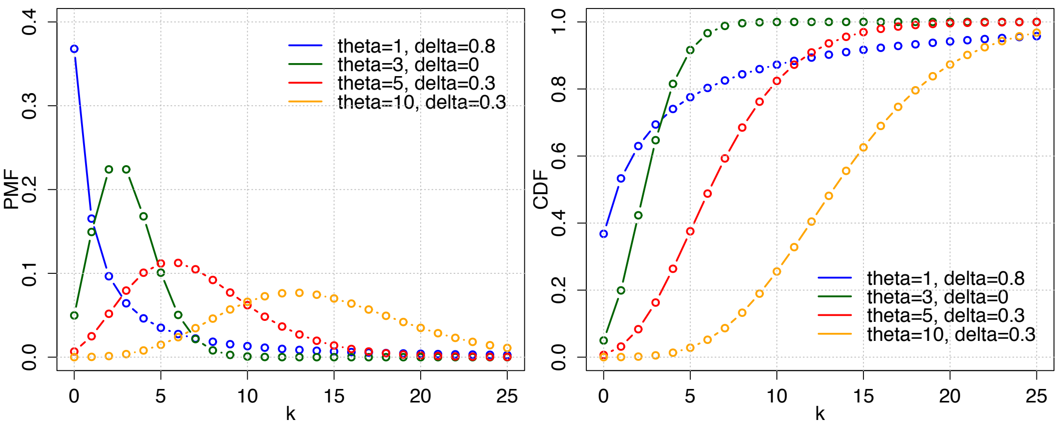

Figure 1.22: GeneralizedPoisson1 distribution plotted using the provided R code.

Functions

PMF

$$\frac{\theta(\theta+\delta k)^{k-1}\times e^{-\theta-\delta k}}{k!}$$

PMF in R

```
(theta*(theta+k*delta)^(k-1) * exp(-theta-k*delta)) / factorial(k)
```

CDF

$$\Sigma_{i=1}^{x} f(i), x \in \{0,1,2,...\} \text{ with } f \text{ the PMF}$$

CDF in R

```
cumsum(PMF)
```

Characteristics

Mean

$$\frac{\theta}{1-\delta}$$

Variance

$$\frac{\theta}{(1-\delta)^3}$$

Relationships

- Relationship pair: $GeneralizedPoisson1(\theta,\delta) \rightarrow Poisson1(\lambda)$
- Relationship type: Special case
- Relationship definition: $\delta = 0, \theta = \mu$

- Relationship pair: $GeneralizedPoisson1(\theta,\delta) \rightarrow GeneralizedPoisson2(\mu,\delta)$
- Relationship type: Reparameterisation
- Relationship definition: $\mu = \theta/(1-\delta)$

- Relationship pair: $GeneralizedPoisson2(\mu,\delta) \rightarrow GeneralizedPoisson1(\theta,\delta)$
- Relationship type: Reparameterisation
- Relationship definition: $\theta = \mu(1-\delta)$

References

[Yang et al., 2007], [Consul and Famoye, 2006]
http://finzi.psych.upenn.edu/library/VGAM/html/genpoisson.html

GeneralizedPoisson3

**name**	Generalized Poisson 3 (ID: 0000758)
**type**	discrete
**variate**	y, scalar
**support**	$y \in \{0, 1, 2, 3, \dots\}$

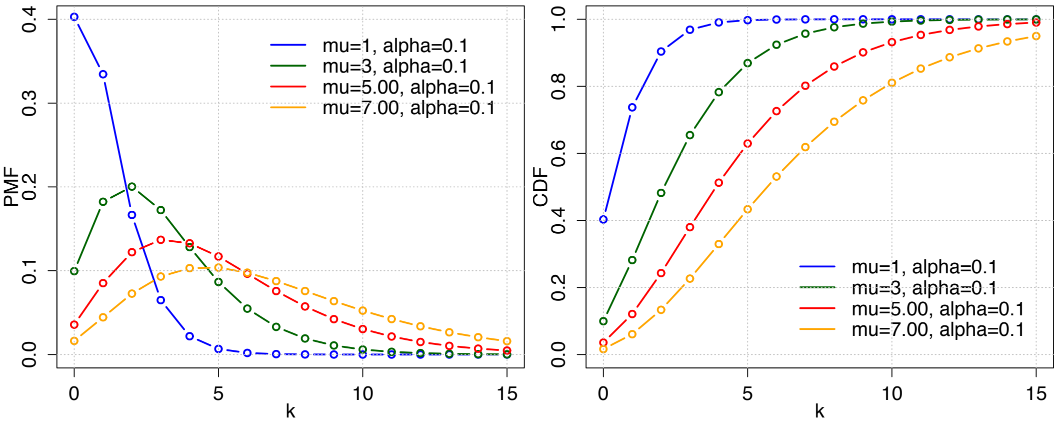

Figure 1.23: GeneralizedPoisson3 distribution plotted using the provided R code.

Parameter: mean

**name**	mean
**type**	scalar
**symbol**	μ
**definition**	$\mu > 0$

Parameter: dispersion

**name**	dispersion
**type**	scalar
**symbol**	α
**definition**	$\alpha > -1, \alpha \in R$

Functions

PMF

$$\left(\frac{\mu}{1+\alpha\mu}\right)^y \frac{(1+\alpha y)^{y-1}}{y!} \exp\left[\frac{-\mu(1+\alpha y)}{1+\alpha\mu}\right]$$

PMF in R

```
(mu/(1+alpha*mu))^y *(1+alpha*y)^(y-1)/factorial(y)*exp(-mu*(1+alpha*y)/(1+alpha*mu))
```

CDF

$$\Sigma_{i=1}^{x} f(i), x \in \{0, 1, 2, ...\} \text{ with } f \text{ the PMF}$$

CDF in R

```
cumsum(PMF)
```

Characteristics

Mean

$$\mu$$

Variance

$$\mu(1+\alpha\mu)^2$$

Relationships

- Relationship pair: $GeneralizedPoisson3(\mu, \alpha) \rightarrow Poisson1(\lambda)$
- Relationship type: Special case
- Relationship definition: $\alpha = 0, \lambda = \mu$
- Relationship pair: $ZeroInflatedGeneralizedPoisson1(\mu, \alpha, p0) \rightarrow GeneralizedPoisson3(\mu, \alpha)$
- Relationship type: Special case
- Relationship definition: $p0 = 0$

References

[Hilbe, 2011], [Famoye and Singh, 2006], [Ismail and Zamani, 2013]

GeneralizedPoisson2

**name**	GeneralizedPoisson2 (ID: 0000735)
**type**	discrete
**variate**	k, scalar
**support**	$k \in \{0, 1, 2, 3, \dots\}$

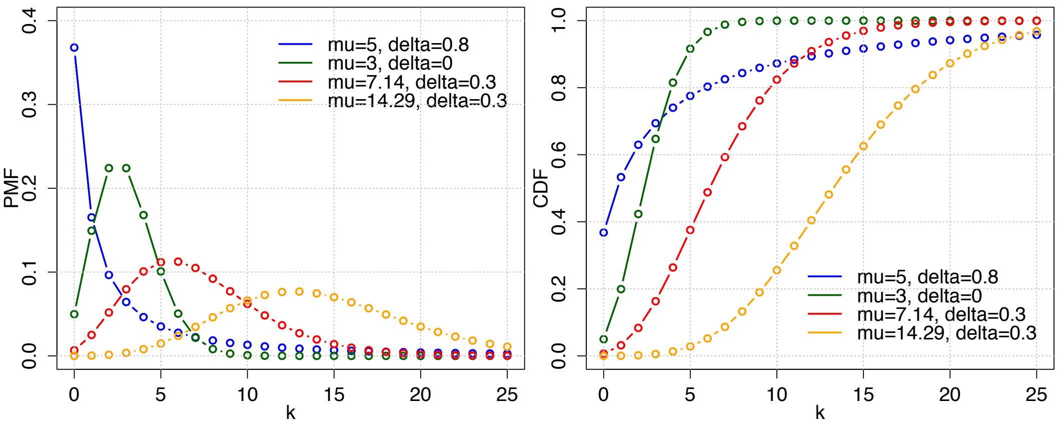

Figure 1.24: GeneralizedPoisson2 distribution plotted using the provided R code.

Parameter: mean

**name**	mean
**type**	scalar
**symbol**	μ
**definition**	$\mu > 0$

Parameter: dispersion

**name**	dispersion
**type**	scalar
**symbol**	δ
**definition**	$max(-1, -\mu/m) < \delta < 1$ with $m(\geq 4)$ the largest positive integer for which $\mu + m\delta > 0$.

Functions

PMF

$$\frac{\mu(1-\delta)[\mu(1-\delta)+\delta k]^{k-1}}{k!} e^{-[\mu(1-\delta)+\delta k]}$$

PMF in R

```
-(mu*(1-delta)*(mu*(1-delta)+delta*k)^(k-1)) / factorial(k) * exp(-(mu*(1-delta)+delta*k))
```

CDF

$$\Sigma_{i=1}^{x} f(i), x \in \{0, 1, 2, ...\} \text{ with } f \text{ the PMF}$$

CDF in R

```
cumsum(PMF)
```

Characteristics

Mean

$$\mu$$

Variance

$$\frac{\mu}{(1-\delta)^2}$$

Relationships

- Relationship pair: $GeneralizedPoisson2(\mu, \delta) \rightarrow Poisson1(\lambda)$
- Relationship type: Special case
- Relationship definition: $\delta = 0, \lambda = \mu$

- Relationship pair: $GeneralizedPoisson2(\mu, \delta) \rightarrow GeneralizedPoisson1(\theta, \delta)$
- Relationship type: Reparameterisation
- Relationship definition: $\theta = \mu(1 - \delta)$

- Relationship pair: $GeneralizedPoisson1(\theta, \delta) \rightarrow GeneralizedPoisson2(\mu, \delta)$
- Relationship type: Reparameterisation
- Relationship definition: $\mu = \theta/(1 - \delta)$

References

[Plan, 2014], [Yang et al., 2007]

Geometric1

**name**	Geometric 1 (ID: 0000782)
**type**	discrete
**variate**	k, scalar
**support**	$k \in \{0, 1, 2, 3, \dots\}$, number of failures

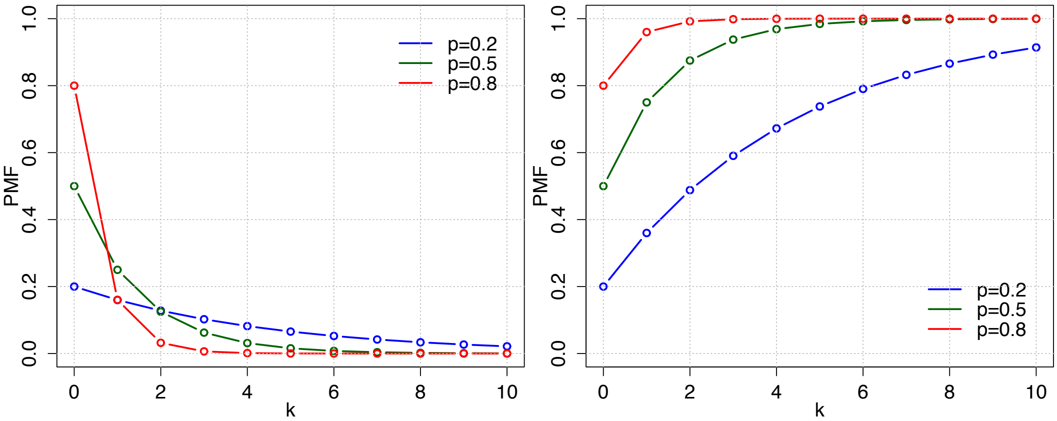

Figure 1.25: Geometric1 distribution plotted using the provided R code.

Model

The geometric distribution is the probability distribution of the number $Y = X - 1$ of failures before the first success, supported on the set $\{0, 1, 2, 3, ...\}$.

Parameter: probability

**name**	success probability
**type**	scalar
**symbol**	p
**definition**	$0 < p < 1$

Functions

PMF

$$(1-p)^k\, p$$

PMF in R

```
p*(1-p)^k
```

CDF

$$1-(1-p)^{k+1}$$

CDF in R

```
1-(1 - p)^(k+1)
```

Characteristics

Mean

$$\frac{1-p}{p}$$

Median

$$\left\lceil \frac{-1}{\log_2(1-p)} - 1 \right\rceil \text{ (not unique if } -1/\log_2(1-p)-1 \text{ is an integer)}$$

Mode

$$0$$

Variance

$$\frac{1-p}{p^2}$$

Relationships

- Relationship pair: $Geometric1(p) \rightarrow NegativeBinomial1(r,p)$
- Relationship type: Transformation
- Relationship definition: $\Sigma X(iid)$

- Relationship pair: $NegativeBinomial1(r,p) \rightarrow Geometric1(p)$
- Relationship type: Special case
- Relationship definition: $n = 1$

References

[Leemis and Mcqueston, 2008]
http://en.wikipedia.org/wiki/Geometric_distribution
http://www.uncertml.org/distributions/geometric
http://www.math.wm.edu/~leemis/chart/UDR/PDFs/PascalGeometric.pdf
http://www.math.wm.edu/~leemis/chart/UDR/PDFs/GeometricPascal.pdf

Gompertz1

**name**	Gompertz 1 (ID: 0000008)
**type**	continuous
**variate**	x, scalar
**support**	$x \in (-\infty, +\infty)$

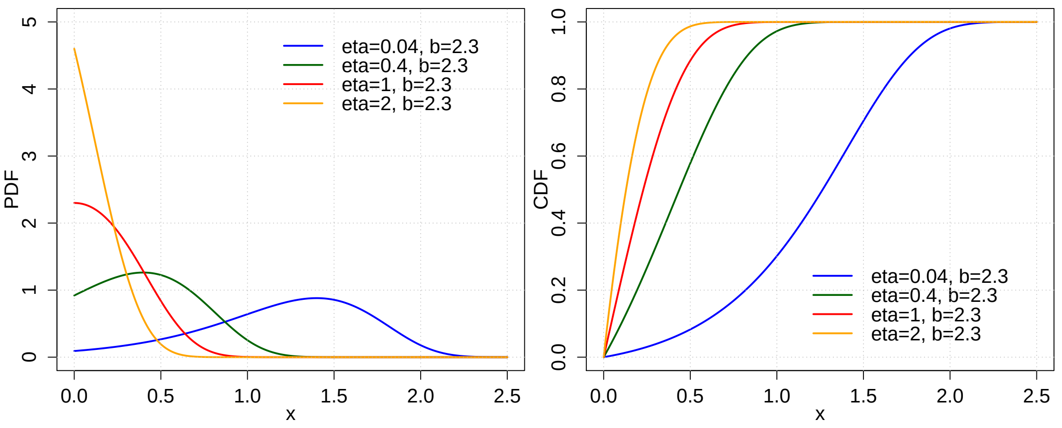

Figure 1.26: Gompertz1 distribution plotted using the provided R code.

Parameter: shape

**name**	shape
**type**	scalar
**symbol**	η
**definition**	$\eta > 0$

Parameter: scale

**name**	scale
**type**	scalar
**symbol**	b
**definition**	$b > 0$

Functions

PDF

$$b\eta e^{bx} e^{\eta} \exp\left(-\eta e^{bx}\right)$$

PDF in R

```
b*eta*exp(b*x)*exp(eta)*exp(-eta*exp(b*x))
```

CDF

$$1 - \exp\left(-\eta\left(e^{bx} - 1\right)\right)$$

CDF in R

```
1-exp(-eta*(exp(b*x)-1))
```

Characteristics

Median

$$(1/b)\log\left[(-1/\eta)\log(1/2) + 1\right]$$

Rerefences

https://en.wikipedia.org/wiki/Gompertz_distribution

Gumbel1

**name**	Gumbel 1 (ID: 0000032)
**type**	continuous
**variate**	x, scalar
**support**	$x \in (-\infty, +\infty)$

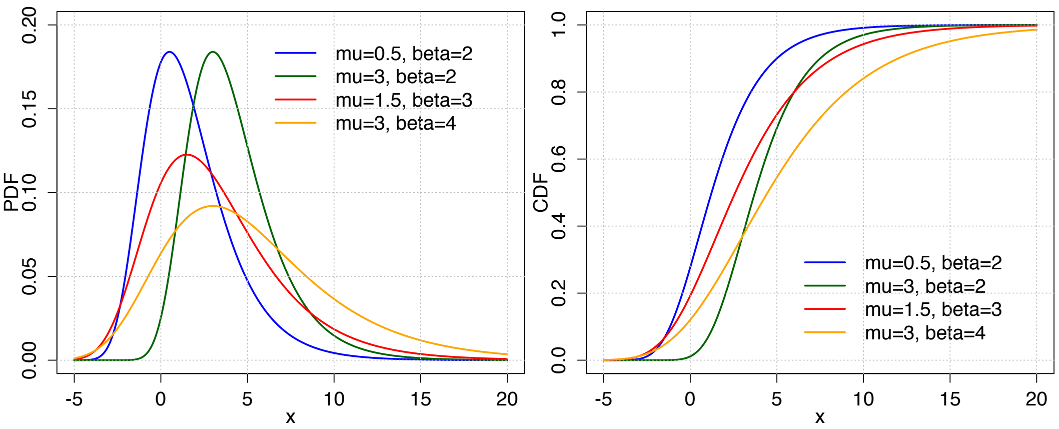

Figure 1.27: Gumbel1 distribution plotted using the provided R code.

Parameter: location

**name**	location
**type**	scalar
**symbol**	μ
**definition**	$\mu \in R$

Parameter: scale

**name**	scale
**type**	scalar
**symbol**	β
**definition**	$\beta > 0, \beta \in R$

Functions

PDF

$$\frac{e^{-e^{-\frac{x-\mu}{\beta}}} e^{-\frac{x-\mu}{\beta}}}{\beta}$$

PDF in R

```
(exp(-exp(-(x-mu)/beta)) * exp(-(x-mu)/beta))/beta
```

CDF

$$e^{-e^{-(x-\mu)/\beta}}$$

CDF in R

```
exp(-exp(-(x-mu)/beta))
```

Characteristics

Mean

$$\mu + \beta \gamma_E; \text{ with is Euler constant } \gamma_E$$

Median

$$\mu - \beta \ln(\ln(2))$$

Mode

$$\mu$$

Variance

$$\frac{\pi^2}{6}\beta^2$$

References

https://en.wikipedia.org/wiki/Gumbel_distribution

HalfNormal1

**name**	Half-normal 1 (ID: 0000068)
**type**	continuous
**variate**	x, scalar
**support**	$x \in [0, +\infty)$

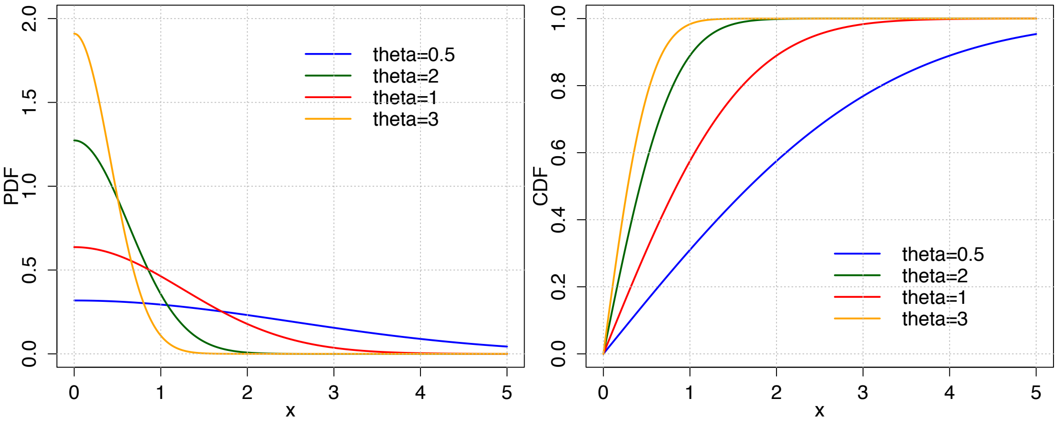

Figure 1.28: HalfNormal1 distribution plotted using the provided R code.

Parameter: inverseScale

**name**	inverse scale
**type**	scalar
**symbol**	θ
**definition**	$\theta > 0$

Functions

PDF

$$\frac{2\theta}{\pi} e^{-\theta^2 x^2/\pi}$$

PDF in R

```
2*theta/pi * exp(-theta^2 * x^2 / pi)
```

CDF

$$\mathrm{erf}(\theta x/\sqrt{\pi})$$

CDF in R

```
erf(theta * x / sqrt(pi))
```

Characteristics

Mean

$$1/\theta$$

Variance

$$\frac{\pi - 2}{2\theta^2}$$

Relationships

- Relationship pair: $TruncatedNormal1(\mu, \sigma, a, b) \rightarrow HalfNormal1(\theta)$
- Relationship type: Special case
- Relationship definition: $\mu = 0, a = 0, b = \infty$

References

[Forbes et al., 2011]
`http://reference.wolfram.com/language/ref/HalfNormalDistribution.html`
`http://mathworld.wolfram.com/Half-NormalDistribution.html`

Hypergeometric1

**name**	Hypergeometric 1 (ID: 0000126)
**type**	discrete
**variate**	k, scalar
**support**	$k \in \{\max(0,\, n + K - N),\, \ldots,\, \min(n,\, K)\}$

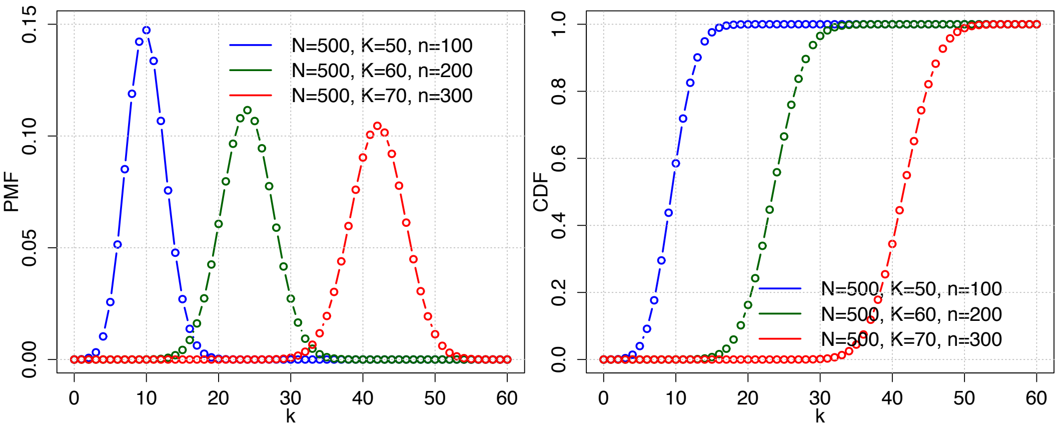

Figure 1.29: Hypergeometric1 distribution plotted using the provided R code.

Model

Hypergeometric distribution is a discrete probability distribution that describes the probability of k successes in n draws, without replacement, from a finite population of size N that contains exactly K successes, wherein each draw is either a success or a failure. In contrast, the binomial distribution describes the probability of k successes in n draws with replacement.

Parameter: populationSize

**name**	population size
**type**	scalar
**symbol**	N
**definition**	$N \in \{0, 1, 2, \ldots\}$

Parameter: numberOfSuccesses

**name**	number of successes
**type**	scalar
**symbol**	K
**definition**	$K \in \{0, 1, 2, \ldots, N\}$

Parameter: numberOfTrials

**name**	number of trials
**type**	scalar
**symbol**	n
**definition**	$n \in \{0, 1, 2, \dots, N\}$

Functions

PMF

$$\frac{\binom{K}{k}\binom{N-K}{n-k}}{\binom{N}{n}}$$

PMF in R

```
choose(K,k)*choose(M-K,n-k)/choose(M,n)
```

CDF

$$1 - \frac{\binom{n}{k+1}\binom{N-n}{K-k-1}}{\binom{N}{K}} {}_3F_2\left[\begin{array}{c} 1,\ k+1-K,\ k+1-n \\ k+2,\ N+k+2-K-n \end{array};1\right]$$

CDF in R

```
cumsum(PMF)
```

Characteristics

Mean

$$n\frac{K}{N}$$

Mode

$$\left\lfloor \frac{(n+1)(K+1)}{N+2} \right\rfloor$$

Variance

$$n\frac{K}{N}\frac{(N-K)}{N}\frac{N-n}{N-1}$$

Relationships

- Relationship pair: $Hypergeometric1(N, K, n) \rightarrow Binomial1(n, p)$
- Relationship type:
- Relationship definition: $p = K/N, n = n, N \rightarrow \infty$

- Relationship pair: $Hypergeometric1(N, K, n) \rightarrow Poisson1(\lambda)$
- Relationship type: Limiting
- Relationship definition: $X \sim Hypergeometric1(N, K, n) \Rightarrow Y \sim Poisson1(\lambda)$ as K, N and n tend to infinity for K/N small and $nK/N \rightarrow \lambda$

- Relationship pair: $Hypergeometric1(N, K, n) \rightarrow Normal1(\mu, \sigma)$
- Relationship type: Limiting
- Relationship definition: $X \sim Hypergeometric1(N, K, n) \Rightarrow Y \sim Normal1(\mu, \sigma)$ for large n, but K/N not too small

References

[Forbes et al., 2011], [Leemis and Mcqueston, 2008]
http://en.wikipedia.org/wiki/Hypergeometric_distribution
http://www.uncertml.org/distributions/hypergeometric
http://www.math.wm.edu/~leemis/chart/UDR/PDFs/HypergeometricBinomial.pdf

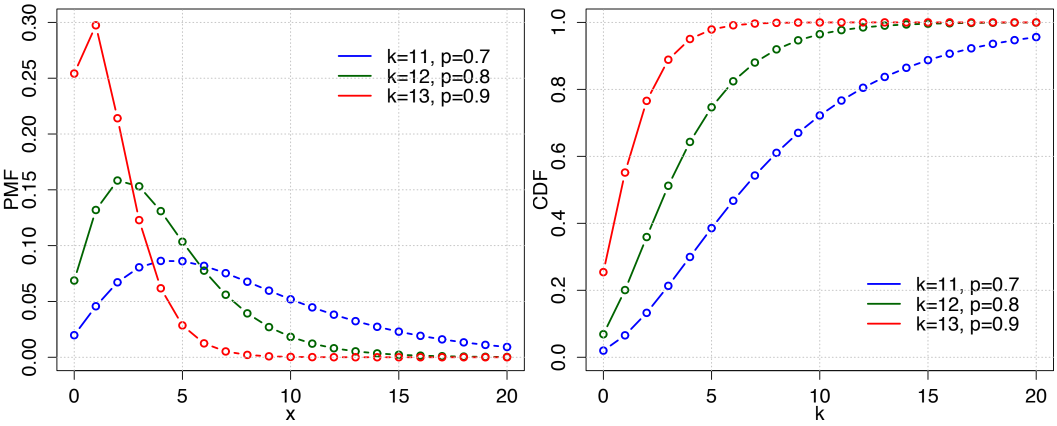

Figure 1.30: InverseBinomial1 distribution plotted using the provided R code.

InverseBinomial1

**name**	Inverse Binomial 1 (ID: 0000154)
**type**	discrete
**variate**	x, scalar
**support**	$x \in \{0, 1, 2, 3, \dots\}$

Parameter: index

**name**	index parameter
**type**	scalar
**symbol**	k
**definition**	$k \in \{0, 1, 2, \dots\}$

Parameter: probability

**name**	probability
**type**	scalar
**symbol**	p
**definition**	$1/2 < p < 1$

Functions

PMF

$$\frac{k\,\Gamma(2x+k)}{\Gamma(x+1)\,\Gamma(x+k+1)}\,p^{k+x}(1-p)^x$$

PMF in R

```
(k * gamma(2*x+k)) / (gamma(x+1) * gamma(x+k+1)) * p^(x+k) * (1-p)^x
```

Characteristics

Mean

$$k(1-p)/(2p-1)$$

Variance

$$kp(1-p)/(2p-1)^3$$

Relationships

- Relationship pair: $GeneralizedNegativeBinomial1(\theta, \beta, m) \rightarrow InverseBinomial1(k, p)$
- Relationship type:
- Relationship definition: $\beta = 2, \theta = 1 - p$

References

[Yanagimoto, 1989]
`https://cran.r-project.org/web/packages/VGAM/VGAM.pdf`

InverseGamma1

**name**	Inverse-Gamma 1 (ID: 0000182)
**type**	continuous
**variate**	x, scalar
**support**	$x \in (0, +\infty)$

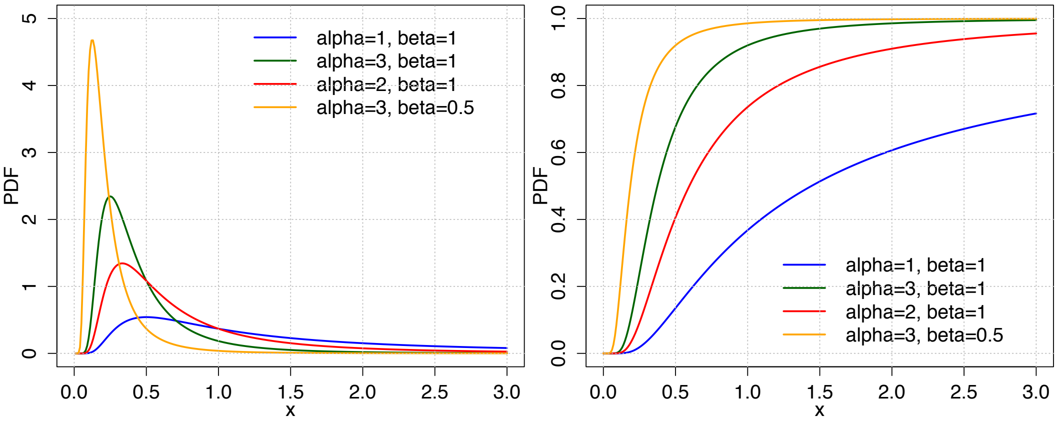

Figure 1.31: InverseGamma1 distribution plotted using the provided R code.

Parameter: shape

**name**	shape
**type**	scalar
**symbol**	α
**definition**	$\alpha > 0, \alpha \in R$

Parameter: scale

**name**	scale
**type**	scalar
**symbol**	β
**definition**	$\beta > 0, \beta \in R$

Functions

PDF

$$\frac{\beta^\alpha}{\Gamma(\alpha)} x^{-\alpha-1} \exp\left(\frac{-\beta}{x}\right)$$

PDF in R

```
beta^alpha/gamma(alpha) * x^(-alpha-1) * exp(-beta/x)
```

CDF

$$\frac{\Gamma(\alpha, \beta/x)}{\Gamma(\alpha)}$$

CDF in R

```
Igamma(alpha, beta/x, lower=F) / gamma(alpha)
```

Characteristics

Mean

$$\frac{\beta}{\alpha - 1} \text{ for } \alpha > 1$$

Mode

$$\frac{\beta}{\alpha + 1}$$

Variance

$$\frac{\beta^2}{(\alpha - 1)^2(\alpha - 2)} \text{ for } \alpha > 2$$

Relationships

- Relationship pair: $Gamma1(\alpha, \beta) \rightarrow InverseGamma1(\alpha, \beta)$
- Relationship type: Transformation
- Relationship definition: If $X \sim Gamma1(\alpha, \beta)$ then $X^{-1} \sim InverseGamma1(\alpha, \beta^{-1})$

References

http://en.wikipedia.org/wiki/Inverse-gamma_distribution
http://www.uncertml.org/distributions/inverse-gamma

InverseGaussian1

**name**	Inverse Gaussian 1 (ID: 0000212)
**type**	continuous
**variate**	x, scalar
**support**	$x \in (0, +\infty)$

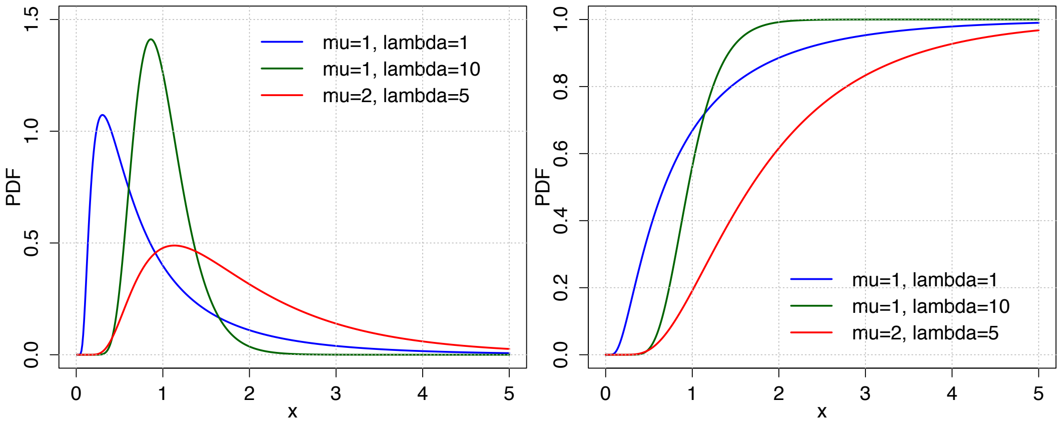

Figure 1.32: InverseGaussian1 distribution plotted using the provided R code.

Parameter: shape

**name**	shape
**type**	scalar
**symbol**	λ
**definition**	$\lambda > 0$

Parameter: mean

**name**	mean
**type**	scalar
**symbol**	μ
**definition**	$\mu > 0$

Functions

PDF

$$\sqrt{\frac{\lambda}{2\pi x^3}} \exp\Big(-\frac{\lambda}{2\mu^2 x}(x-\mu)^2\Big)$$

PDF in R

```
sqrt(lambda/(2*pi*x^3) ) * exp(-lambda/(2*mu^2 x) * (x-mu)^2)
```

CDF

$$\Phi\left(\sqrt{\frac{\lambda}{x}}\left(\frac{x}{\mu}-1\right)\right) + \exp\left(\frac{2\lambda}{\mu}\right)\Phi\left(-\sqrt{\frac{\lambda}{x}}\left(\frac{x}{\mu}+1\right)\right)$$

CDF in R

```
pnorm(sqrt(lambda/x) * (x/mu-1)) + exp(2*lambda/mu) * pnorm(-sqrt(lambda/x) * (x/mu+1))
```

Relationships

- Relationship pair: $InverseGaussian1(\lambda, \mu) \rightarrow StandardNormal1(0, 1)$
- Relationship type: Limiting
- Relationship definition: $\lambda \rightarrow \infty$

- Relationship pair: $InverseGaussian1(\lambda, \mu) \rightarrow ChiSquared1(k)$
- Relationship type: Transformation
- Relationship definition: $X \sim InverseGaussian1(\lambda, \mu)$ and $Y = \lambda(X-\mu)^2/(\mu^2 X) \Rightarrow Y \sim ChiSquared1(k)$

References

[Leemis and Mcqueston, 2008]
https://en.wikipedia.org/wiki/Inverse_Gaussian_distribution
http://www.math.wm.edu/~leemis/chart/UDR/PDFs/InversegaussianStandardnormal.pdf
http://www.math.wm.edu/~leemis/chart/UDR/PDFs/InversegaussianChisquare.pdf

InverseWishart1

**name**	Inverse-Wishart 1 (ID: 0000232)
**type**	continuous
**variate**	X, matrix
**support**	$X(p \times p)$ – positive-definite matrix

Parameter: scaleMatrix

**name**	scale matrix
**type**	matrix
**symbol**	Ψ
**definition**	$\Psi > 0$, positive-definite matrix

Parameter: degreesOfFreedom

**name**	degrees of freedom
**type**	scalar
**symbol**	ν
**definition**	$\nu > p - 1, \nu \in R$

Functions

PDF

$$\frac{|\Psi|^{\frac{\nu}{2}}}{2^{\frac{\nu p}{2}}\Gamma_p(\frac{\nu}{2})}|X|^{-\frac{\nu+p+1}{2}} e^{-\frac{1}{2}\mathrm{tr}(\Psi X^{-1})}$$

Characteristics

Mean

$$\frac{\Psi}{\nu - p - 1} \text{ for } \nu > p + 1$$

Mode

$$\frac{\Psi}{\nu + p + 1}$$

References

https://en.wikipedia.org/wiki/Inverse-Wishart_distribution

Laplace1

**name**	Laplace 1 (ID: 0000256)
**type**	continuous
**variate**	x, scalar
**support**	$x \in (-\infty, +\infty)$

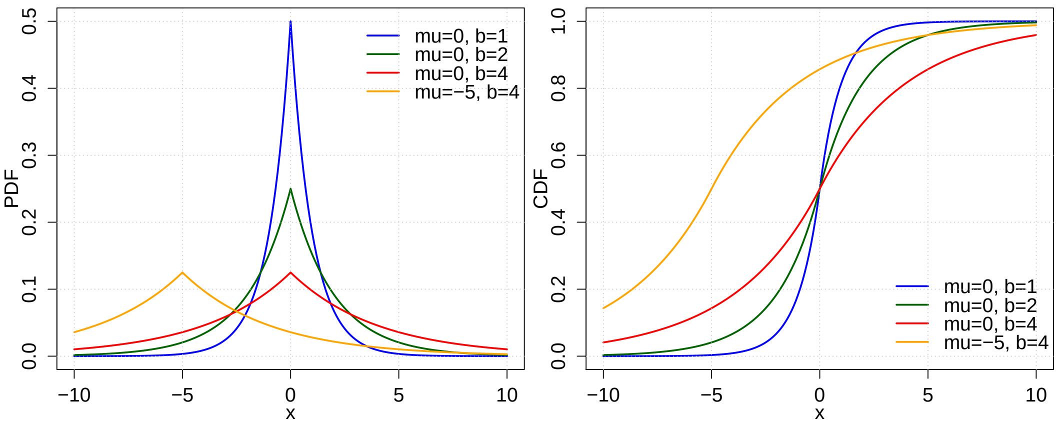

Figure 1.33: Laplace1 distribution plotted using the provided R code.

Parameter: location

**name**	location
**type**	scalar
**symbol**	μ
**definition**	$\mu \in R$

Parameter: scale

**name**	scale
**type**	scalar
**symbol**	b
**definition**	$b > 0, b \in R$

Functions

PDF

$$\frac{1}{2\,b} \exp\left(-\frac{|x-\mu|}{b}\right)$$

PDF in R

```
1/(2*b) * exp(- abs(x-mu)/b )
```

CDF

$$\begin{cases} \frac{1}{2}\exp\left(\frac{x-\mu}{b}\right) & \text{if } x < \mu \\ 1 - \frac{1}{2}\exp\left(-\frac{x-\mu}{b}\right) & \text{if } x \geq \mu \end{cases}$$

CDF in R

```
1/2 * exp( (x-mu)/b ) # for x < mu
1- 1/2 * exp( -(x-mu)/b ) # x >= mu
```

Characteristics

Mean

$$\mu$$

Median

$$\mu$$

Mode

$$\mu$$

Variance

$$2b^2$$

Relationships

- Relationship pair: $Laplace1(\mu, b) \to Laplace2(\mu, \tau)$
- Relationship type: Reparameterisation
- Relationship definition: $\tau = 1/b$

- Relationship pair: $Laplace2(\mu, \tau) \to Laplace1(\mu, b)$
- Relationship type: Reparameterisation
- Relationship definition: $b = 1/\tau$

References

https://en.wikipedia.org/wiki/Laplace_distribution
http://www.uncertml.org/distributions/laplace

Laplace2

**name**	Laplace 2 (ID: 0000283)
**type**	continuous
**variate**	x, scalar
**support**	$x \in (-\infty, +\infty)$

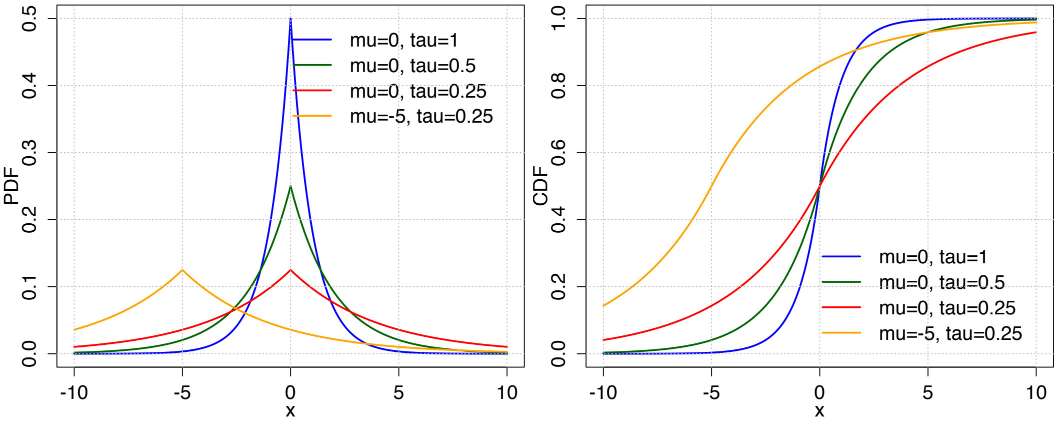

Figure 1.34: Laplace2 distribution plotted using the provided R code.

Parameter: location

**name**	location
**type**	scalar
**symbol**	μ
**definition**	$\mu \in R$

Parameter: tau

**name**	precision
**type**	scalar
**symbol**	τ
**definition**	$\tau > 0, \tau \in R$

Functions

PDF

$$\frac{\tau}{2} \exp\left(-\tau|x-\mu|\right)$$

PDF in R

```
tau/2 * exp(-tau * abs(x-mu))
```

CDF

$$\begin{cases} \frac{1}{2}\exp\left(\tau(x-\mu)\right) & \text{if } x < \mu \\ 1-\frac{1}{2}\exp\left(-\tau(x-\mu)\right) & \text{if } x \geq \mu \end{cases}$$

CDF in R

```
1/2 * exp( tau*(x-mu) ) # for x < mu
1- 1/2 * exp( -tau*(x-mu) ) # x >= mu
```

Characteristics

Mean

$$\mu$$

Variance

$$2/\tau^2$$

Relationships

- Relationship pair: $Laplace2(\mu, \tau) \rightarrow Laplace1(\mu, b)$
- Relationship type: Reparameterisation
- Relationship definition: $b = 1/\tau$

- Relationship pair: $Laplace1(\mu, b) \rightarrow Laplace2(\mu, \tau)$
- Relationship type: Reparameterisation
- Relationship definition: $\tau = 1/b$

References

[Spiegelhalter et al., 2003], [Lunn, 2012]

LogLogistic1

**name**	Log-Logistic 1 (ID: 0000377)
**type**	continuous
**variate**	x, scalar
**support**	$x \in [0, +\infty)$

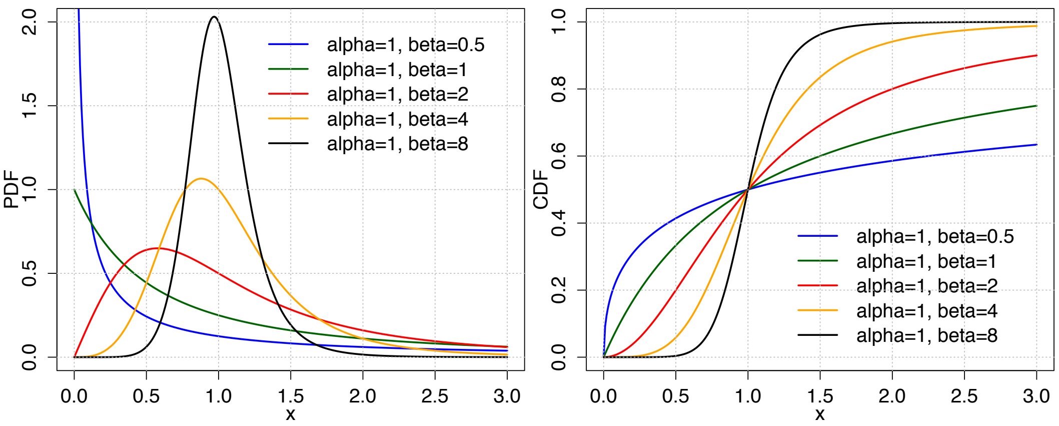

Figure 1.35: LogLogistic1 distribution plotted using the provided R code.

Parameter: scale

**name**	scale
**type**	scalar
**symbol**	α
**definition**	$\alpha > 0$

Parameter: shape

**name**	shape
**type**	scalar
**symbol**	β
**definition**	$\beta > 0$

Functions

PDF

$$\frac{(\beta/\alpha)(x/\alpha)^{\beta-1}}{(1+(x/\alpha)^{\beta})^2}$$

PDF in R

```
(beta/alpha)*(x/alpha)^(beta-1) / (1+(x/alpha)^beta)^2
```

CDF

$$\frac{1}{1+(x/\alpha)^{-\beta}}$$

CDF in R

```
1 / (1+(x/alpha)^(-beta))
```

Characteristics

Mean

$$\frac{\alpha\pi/\beta}{\sin(\pi/\beta)} \text{ if } \beta > 1, \text{ else undefined}$$

Median

$$\alpha$$

Mode

$$\alpha\Big(\frac{\beta-1}{\beta+1}\Big)^{1/\beta} \text{ if } \beta > 1, 0 \text{ otherwise}$$

Variance

$$\alpha^2\Big(\frac{2\pi/\beta}{\sin(2\pi/\beta)} - \frac{(\pi/\beta)^2}{\sin^2(\pi/\beta)}\Big), \text{ for } \beta > 2$$

Relationships

- Relationship pair: $LogLogistic1(\alpha, \beta) \to Logistic1(\mu, s)$
- Relationship type: Transformation
- Relationship definition: If $X \sim LogLogistic1(\alpha, \beta) \Rightarrow Y = log(X) \sim Logistic1(\mu, s)$ with $\mu = \log(\alpha), s = 1/\beta)$

- Relationship pair: $LogLogistic1(\alpha, \beta) \to LogLogistic2(\lambda, \kappa)$
- Relationship type: Reparameterisation
- Relationship definition: $\lambda = 1/\alpha$

- Relationship pair: $LogLogistic2(\lambda, \kappa) \to LogLogistic1(\alpha, \beta)$
- Relationship type: Reparameterisation
- Relationship definition: $\alpha = 1/\lambda$

References

http://en.wikipedia.org/wiki/Logistic_distribution
http://www.uncertml.org/distributions/logistic

LogLogistic2

**name**	Log-Logistic 2 (ID: 0000402)
**type**	continuous
**variate**	x, scalar
**support**	$x \in (0, +\infty)$

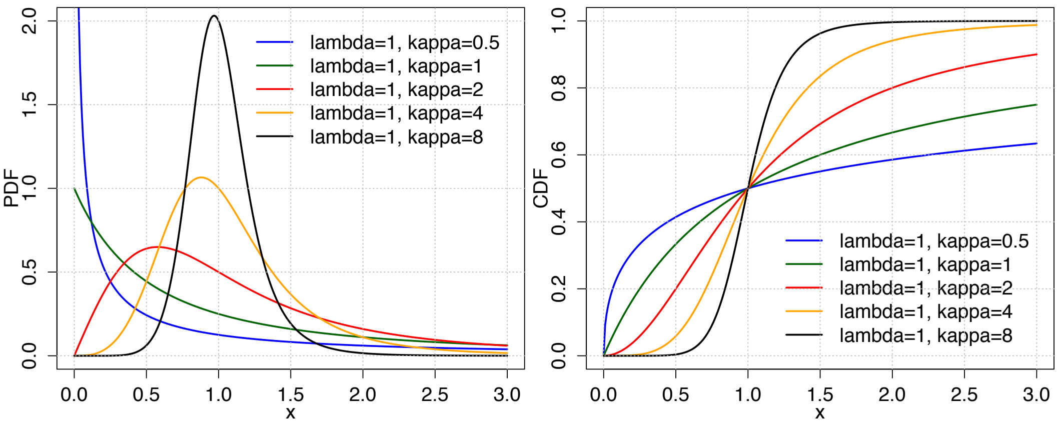

Figure 1.36: LogLogistic2 distribution plotted using the provided R code.

Parameter: scale

**name**	scale
**type**	scalar
**symbol**	λ
**definition**	$\lambda > 0$

Parameter: shape

**name**	shape
**type**	scalar
**symbol**	κ
**definition**	$\kappa > 0$

Functions

PDF

$$\frac{\lambda\kappa(\lambda x)^{\kappa-1}}{(1+(\lambda x)^{\kappa})^2}$$

PDF in R

```
(lambda*kappa*(lambda*x)^(kappa-1)) / (1+(lambda*x)^kappa)^2
```

CDF

$$\frac{(\lambda x)^{\kappa}}{1+(\lambda x)^{\kappa}}$$

CDF in R

```
(lambda*x)^kappa / (1+(lambda*x)^kappa)
```

Characteristics

Mean

$$\frac{\pi}{\kappa\lambda\sin(\pi/\kappa)}$$

Median

$$1/\lambda$$

Variance

$$\frac{\pi\big(2\kappa[1-\cos(\frac{\pi}{\kappa})^2]+\pi\sin\big(\frac{\pi(\kappa+2)}{\kappa}\big)\big)}{\sin\big(\frac{\pi(\kappa+2)}{\kappa}\big)\big(\cos^2(\frac{\pi}{\kappa})-1\big)(\lambda\kappa)^2}$$

Relationships

- Relationship pair: $LogLogistic2(\lambda,\kappa) \to LogLogistic1(\alpha,\beta)$
- Relationship type: Reparameterisation
- Relationship definition: $\alpha = 1/\lambda$

- Relationship pair: $StandardUniform1 \to LogLogistic2(\lambda,\kappa)$
- Relationship type: Transformation
- Relationship definition: If $X \sim StandardUniform1$ and $Y = \frac{1}{\lambda}\left(\frac{1-X}{X}\right)^{1/\kappa} \Rightarrow Y \sim LogLogistic2(\lambda,\kappa)$

- Relationship pair: $LogLogistic1(\alpha,\beta) \to LogLogistic2(\lambda,\kappa)$
- Relationship type: Reparameterisation
- Relationship definition: $\lambda = 1/\alpha$

References

[Leemis and Mcqueston, 2008]
http://www.math.wm.edu/~leemis/chart/UDR/PDFs/Loglogistic.pdf

LogNormal1

**name**	Log-Normal 1 (ID: 0000428)
**type**	continuous
**variate**	x, scalar
**support**	$x \in (0,+\infty)$

Parameter: meanLog

**name**	mean of log(x)
**type**	scalar
**symbol**	μ
**definition**	$\mu \in R$

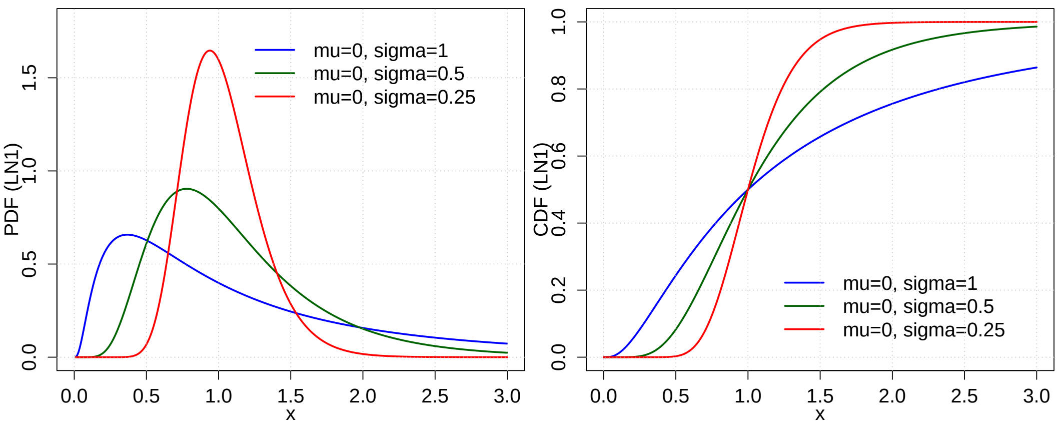

Figure 1.37: LogNormal1 distribution plotted using the provided R code.

Parameter: stdevLog

**name**	shape
**type**	scalar
**symbol**	σ
**definition**	$\sigma > 0$

Functions

PDF

$$\frac{1}{x\sigma\sqrt{2\pi}}\; e^{-\frac{(\log x-\mu)^2}{2\sigma^2}}$$

PDF in R

```
1/(x*sigma*sqrt(2*pi)) * exp((-(log(x)-mu)^2)/(2*sigma^2))
```

CDF

$$\frac{1}{2}+\frac{1}{2}\,\mathrm{erf}\Big[\frac{\log x-\mu}{\sqrt{2}\sigma}\Big]$$

CDF in R

```
1/2 + 1/2 *erf( (log(x)-mu)/(sqrt(2)*sigma) )
```

Characteristics

Mean

$$e^{\mu+\sigma^2/2}$$

Median

$$e^{\mu}$$

Mode

$$e^{\mu-\sigma^2}$$

Variance

$$(e^{\sigma^2}-1)e^{2\mu+\sigma^2}$$

Relationships

- Relationship pair: $LogNormal1(\mu,\sigma) \rightarrow Normal1(\mu,\sigma)$
- Relationship type: Transformation
- Relationship definition: $\log(X)$

- Relationship pair: $LogNormal1(\mu,\sigma) \rightarrow LogNormal2(\mu,v)$
- Relationship type: Reparameterisation
- Relationship definition: $\mu = \mu, v = \sigma^2$

- Relationship pair: $LogNormal1(\mu, \sigma) \rightarrow LogNormal6(m, \sigma_g)$
- Relationship type: Reparameterisation
- Relationship definition: $m = \exp(\mu), \sigma_g = \exp(\sigma)$

- Relationship pair: $LogNormal1(\mu, \sigma) \rightarrow LogNormal3(m, \sigma)$
- Relationship type: Reparameterisation
- Relationship definition: $m = \exp(\mu), \sigma = \sigma$

- Relationship pair: $LogNormal1(\mu, \sigma) \rightarrow LogNormal4(m, cv)$
- Relationship type: Reparameterisation
- Relationship definition: $m = \exp(\mu), cv = \sqrt{\exp(\sigma^2) - 1}$

- Relationship pair: $LogNormal1(\mu, \sigma) \rightarrow LogNormal5(\mu, \tau)$
- Relationship type: Reparameterisation
- Relationship definition: $\mu = \mu, \tau = 1/\sigma^2$

- Relationship pair: $Normal1(\mu, \sigma) \rightarrow LogNormal1(\mu, \sigma)$
- Relationship type: Transformation
- Relationship definition: $\exp(X)$

- Relationship pair: $LogNormal2(\mu, v) \rightarrow LogNormal1(\mu, \sigma)$
- Relationship type: Reparameterisation
- Relationship definition: $\mu_{LogNormal1} = \mu, \sigma_{LogNormal1} = \sqrt{v}$

- Relationship pair: $LogNormal6(m, \sigma_g) \rightarrow LogNormal1(\mu, \sigma)$
- Relationship type: Reparameterisation
- Relationship definition: $\mu = \log(m), \sigma = \log(\sigma_g)$

- Relationship pair: $LogNormal3(m, \sigma) \rightarrow LogNormal1(\mu, \sigma)$
- Relationship type: Reparameterisation
- Relationship definition: $\mu = \log(m), \sigma = \sigma$

- Relationship pair: $LogNormal4(m, cv) \rightarrow LogNormal1(\mu, \sigma)$
- Relationship type: Reparameterisation
- Relationship definition: $\mu = \log(m), \sigma = \sqrt{\log(cv^2 + 1)}$

- Relationship pair: $LogNormal5(\mu, \tau) \rightarrow LogNormal1(\mu, \sigma)$
- Relationship type: Reparameterisation
- Relationship definition: $\mu = \mu, \sigma = 1/\sqrt{\tau}$

References

[Leemis and Mcqueston, 2008]
http://en.wikipedia.org/wiki/Log-normal_distribution
http://www.uncertml.org/distributions/log-normal
http://www.math.wm.edu/~leemis/chart/UDR/PDFs/NormalLognormal.pdf
http://www.math.wm.edu/~leemis/chart/UDR/PDFs/NormalLognormal.pdf

LogNormal2

**name**	Log-Normal 2 (ID: 0000453)
**type**	continuous
**variate**	x, scalar
**support**	$x \in (0, +\infty)$

Parameter: meanLog

**name**	mean of log(x)
**type**	scalar
**symbol**	μ
**definition**	$\mu \in R$

Parameter: varLog

**name**	shape
**type**	scalar
**symbol**	v
**definition**	$v > 0$

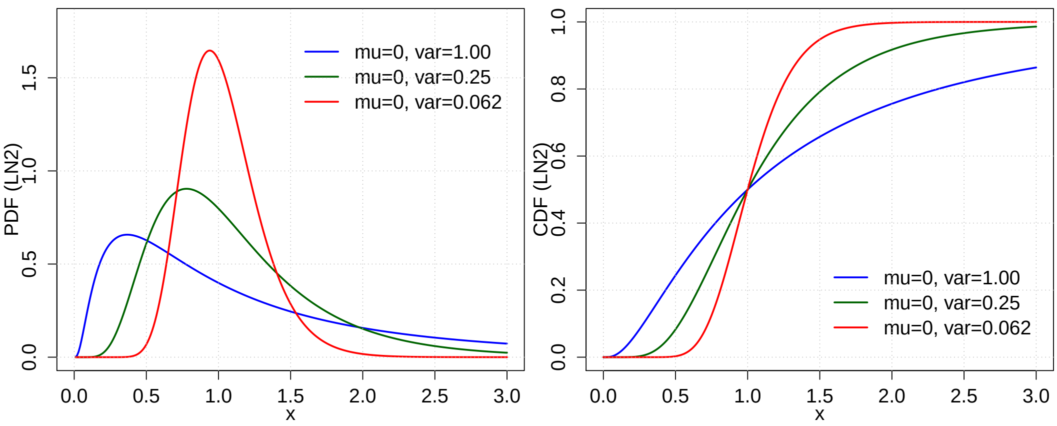

Figure 1.38: LogNormal2 distribution plotted using the provided R code.

Functions

PDF

$$\frac{1}{x\sqrt{v}\sqrt{2\pi}}\; e^{-\frac{(\log x-\mu)^2}{2v}}$$

PDF in R

```
1/(x*sqrt(v)*sqrt(2*pi)) * exp(-(ln(x)-mu)^2/(2*v))
```

CDF

$$\frac{1}{2}+\frac{1}{2}\,\mathrm{erf}\Big[\frac{\log x-\mu}{\sqrt{2}\sqrt{var}}\Big]$$

CDF in R

```
1/2 + 1/2 * erf( (log(x)-mu) / (sqrt(2)*sqrt(var)) )
```

Characteristics

Mean

$$e^{\mu+v/2}$$

Median

$$e^{\mu}$$

Mode

$$e^{\mu-v}$$

Variance

$$(e^v-1)e^{2\mu+v}$$

Relationships

- Relationship pair: $LogNormal2(\mu, v) \rightarrow LogNormal1(\mu, \sigma)$
- Relationship type: Reparameterisation
- Relationship definition: $\mu_{LogNormal1} = \mu, \sigma_{LogNormal1} = \sqrt{v}$
- Relationship pair: $LogNormal2(\mu, v) \rightarrow LogNormal6(m, \sigma_g)$
- Relationship type: Reparameterisation
- Relationship definition: $m = \exp(\mu), \sigma_g = \exp(\sqrt{v})$
- Relationship pair: $LogNormal2(\mu, v) \rightarrow LogNormal3(m, \sigma)$
- Relationship type: Reparameterisation
- Relationship definition: $m = \exp(\mu), \sigma = \sqrt{v}$
- Relationship pair: $LogNormal2(\mu, v) \rightarrow LogNormal4(m, cv)$
- Relationship type: Reparameterisation
- Relationship definition: $m = \exp(\mu), cv = \sqrt{\exp(v) - 1}$

- Relationship pair: $LogNormal2(\mu, v) \to LogNormal5(\mu, \tau)$
- Relationship type: Reparameterisation
- Relationship definition: $\mu = \mu, \tau = 1/v$

- Relationship pair: $LogNormal5(\mu, \tau) \to LogNormal2(\mu, v)$
- Relationship type: Reparameterisation
- Relationship definition: $\mu = \mu, v = 1/\tau$

- Relationship pair: $LogNormal1(\mu, \sigma) \to LogNormal2(\mu, v)$
- Relationship type: Reparameterisation
- Relationship definition: $\mu = \mu, v = \sigma^2$

- Relationship pair: $LogNormal6(m, \sigma_g) \to LogNormal2(\mu, v)$
- Relationship type: Reparameterisation
- Relationship definition: $\mu = \log(m), v = \log(\sigma_g^2)$

- Relationship pair: $LogNormal3(m, \sigma) \to LogNormal2(\mu, v)$
- Relationship type: Reparameterisation
- Relationship definition: $\mu = \log(m), v = \sigma^2$

- Relationship pair: $LogNormal4(m, cv) \to LogNormal2(\mu, v)$
- Relationship type: Reparameterisation
- Relationship definition: $\mu = \log(m), v = \log(cv^2 + 1)$

References

http://en.wikipedia.org/wiki/Log-normal_distribution
http://www.uncertml.org/distributions/log-normal

LogNormal3

**name**	Log-Normal 3 (ID: 0000478)
**type**	continuous
**variate**	x, scalar
**support**	$x \in (0, +\infty)$

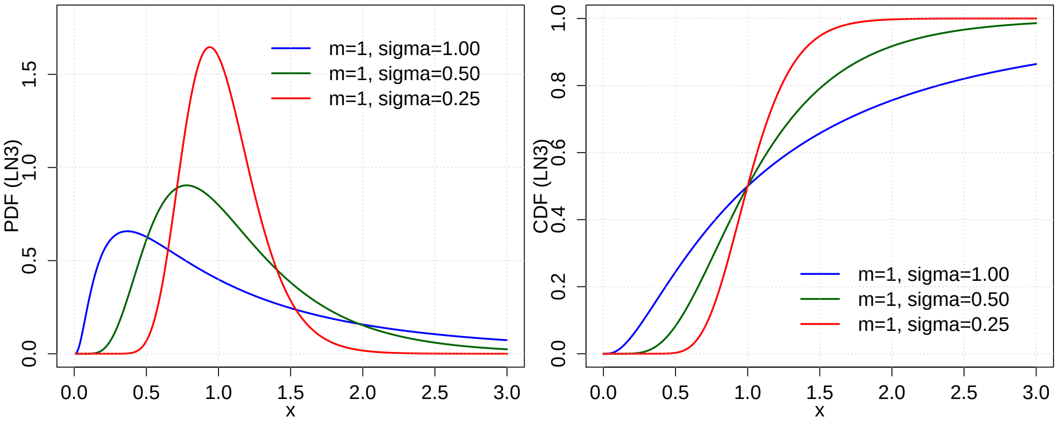

Figure 1.39: LogNormal3 distribution plotted using the provided R code.

Parameter: median

**name**	median / geometric mean
**type**	scalar
**symbol**	m
**definition**	$m > 0$

Parameter: stdevLog

**name**	shape
**type**	scalar
**symbol**	σ
**definition**	$\sigma > 0$

Functions

PDF

$$\frac{1}{x\sigma\sqrt{2\pi}}\, e^{-\frac{[\log(x/m)]^2}{2\sigma^2}}$$

PDF in R

```
1/(x*sigma*sqrt(2*pi)) * exp(-(log(x/m))^2 / (2*sigma^2))
```

CDF

$$\frac{1}{2}+\frac{1}{2}\,\mathrm{erf}\Big[\frac{\log x-\log m}{\sqrt{2}\sigma}\Big]$$

CDF in R

```
1/2 + 1/2 * erf( (log(x)-log(m)) / (sqrt(2)*sigma) )
```

Characteristics

Mean

$$me^{\frac{1}{2}\sigma^2}$$

Median

$$m$$

Mode

$$m/e^{\sigma^2}$$

Variance

$$m^2e^{\sigma^2}[e^{\sigma^2}-1]$$

Relationships

- Relationship pair: $LogNormal3(m,\sigma) \rightarrow LogNormal4(m,cv)$
- Relationship type: Reparameterisation
- Relationship definition: $m=m, cv=\sqrt{\exp(\sigma^2)-1}$

- Relationship pair: $LogNormal3(m,\sigma) \rightarrow LogNormal5(\mu,\tau)$
- Relationship type: Reparameterisation
- Relationship definition: $\mu=\log(m), \tau=1/\sigma^2$

- Relationship pair: $LogNormal3(m,\sigma) \rightarrow LogNormal1(\mu,\sigma)$
- Relationship type: Reparameterisation
- Relationship definition: $\mu=\log(m), \sigma=\sigma$

- Relationship pair: $LogNormal3(m,\sigma) \rightarrow LogNormal6(m,\sigma_g)$
- Relationship type: Reparameterisation
- Relationship definition: $m=m, \sigma_g=\exp(\sigma)$

- Relationship pair: $LogNormal3(m,\sigma) \rightarrow LogNormal2(\mu,v)$
- Relationship type: Reparameterisation
- Relationship definition: $\mu=\log(m), v=\sigma^2$

- Relationship pair: $LogNormal4(m,cv) \rightarrow LogNormal3(m,\sigma)$
- Relationship type: Reparameterisation
- Relationship definition: $m=m, \sigma=\sqrt{\log(cv^2+1)}$

- Relationship pair: $LogNormal5(\mu,\tau) \rightarrow LogNormal3(m,\sigma)$
- Relationship type: Reparameterisation
- Relationship definition: $m=\exp(\mu), \sigma=1/\sqrt{\tau}$

- Relationship pair: $LogNormal1(\mu, \sigma) \rightarrow LogNormal3(m, \sigma)$
- Relationship type: Reparameterisation
- Relationship definition: $m = \exp(\mu), \sigma = \sigma$

- Relationship pair: $LogNormal6(m, \sigma_g) \rightarrow LogNormal3(m, \sigma)$
- Relationship type: Reparameterisation
- Relationship definition: $m = m, \sigma = \log(\sigma_g)$

- Relationship pair: $LogNormal2(\mu, v) \rightarrow LogNormal3(m, \sigma)$
- Relationship type: Reparameterisation
- Relationship definition: $m = \exp(\mu), \sigma = \sqrt{v}$

References

[Leemis and Mcqueston, 2008]
`http://www.math.wm.edu/~leemis/chart/UDR/PDFs/Lognormal.pdf`

LogNormal4

**name**	Log-Normal 4 (ID: 0000500)
**type**	continuous
**variate**	x, scalar
**support**	$x \in (0, +\infty)$

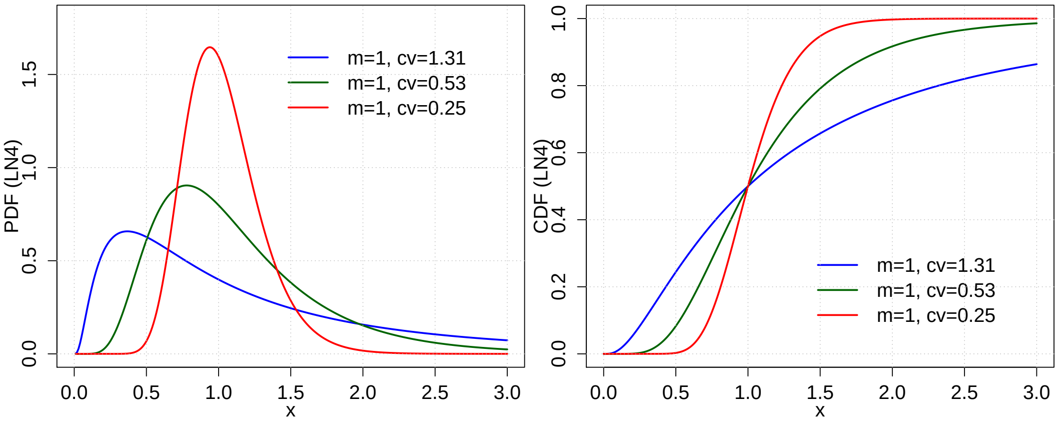

Figure 1.40: LogNormal4 distribution plotted using the provided R code.

Parameter: median

**name**	median / geometric mean
**type**	scalar
**symbol**	m
**definition**	$m > 0$

Parameter: coefVar

**name**	coefficient of variation
**type**	scalar
**symbol**	cv
**definition**	$cv > 0$

Functions

PDF

$$\frac{1}{x\sqrt{\log(cv^2+1)}\sqrt{2\pi}}\, e^{-\frac{[\log(x/m)]^2}{2\ln(cv^2+1)}}$$

PDF in R

```
1/(x*sqrt(log(cv^2+1))*sqrt(2*pi)) * exp( -(log(x/m))^2 / (2*log(cv^2+1)) )
```

CDF

$$\frac{1}{2} + \frac{1}{2}\,\mathrm{erf}\Big[\frac{\log x - \log m}{\sqrt{2}\sqrt{\log(cv^2+1)}}\Big]$$

CDF in R

```
1/2 + 1/2 * erf( (log(x)-log(m)) / (sqrt(2*log(cv^2+1))) )
```

Characteristics

Mean

$$m\sqrt{cv^2+1}$$

Median

$$m$$

Mode

$$m/(cv^2+1)$$

Variance

$$m^2(cv^2+1)cv^2$$

Relationships

- Relationship pair: $LogNormal4(m, cv) \rightarrow LogNormal3(m, \sigma)$
- Relationship type: Reparameterisation
- Relationship definition: $m = m, \sigma = \sqrt{\log(cv^2+1)}$

- Relationship pair: $LogNormal4(m, cv) \rightarrow LogNormal5(\mu, \tau)$
- Relationship type: Reparameterisation
- Relationship definition: $\mu = \log(m), \tau = 1/\log(cv^2+1)$

- Relationship pair: $LogNormal4(m, cv) \rightarrow LogNormal1(\mu, \sigma)$
- Relationship type: Reparameterisation
- Relationship definition: $\mu = \log(m), \sigma = \sqrt{\log(cv^2+1)}$

- Relationship pair: $LogNormal4(m, cv) \rightarrow LogNormal6(m, \sigma_g)$
- Relationship type: Reparameterisation
- Relationship definition: $m = m, \sigma_g = \exp\big(\sqrt{\log(cv^2+1)}\big)$

- Relationship pair: $LogNormal4(m, cv) \rightarrow LogNormal2(\mu, v)$
- Relationship type: Reparameterisation
- Relationship definition: $\mu = \log(m), v = \log(cv^2+1)$

- Relationship pair: $LogNormal3(m, \sigma) \rightarrow LogNormal4(m, cv)$
- Relationship type: Reparameterisation
- Relationship definition: $m = m, cv = \sqrt{\exp(\sigma^2) - 1}$

- Relationship pair: $LogNormal5(\mu, \tau) \rightarrow LogNormal4(m, cv)$
- Relationship type: Reparameterisation
- Relationship definition: $m = \exp(\mu), cv = \sqrt{\exp(1/\tau) - 1}$

- Relationship pair: $LogNormal1(\mu, \sigma) \rightarrow LogNormal4(m, cv)$
- Relationship type: Reparameterisation
- Relationship definition: $m = \exp(\mu), cv = \sqrt{\exp(\sigma^2) - 1}$

- Relationship pair: $LogNormal6(m, \sigma_g) \rightarrow LogNormal4(m, cv)$
- Relationship type: Reparameterisation
- Relationship definition: $m = m, cv = \sqrt{\exp\big(\log^2(\sigma_g)\big) - 1}$

- Relationship pair: $LogNormal2(\mu, v) \rightarrow LogNormal4(m, cv)$
- Relationship type: Reparameterisation
- Relationship definition: $m = \exp(\mu), cv = \sqrt{\exp(v) - 1}$

LogNormal5

**name**	Log-Normal 5 (ID: 0000526)
**type**	continuous
**variate**	x, scalar
**support**	$x \in (0, +\infty)$

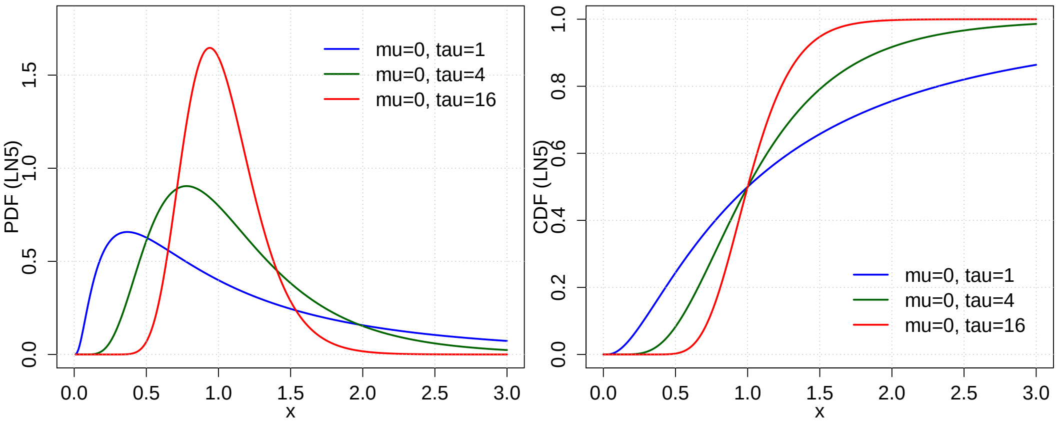

Figure 1.41: LogNormal5 distribution plotted using the provided R code.

Parameter: meanLog

**name**	mean of log(x)
**type**	scalar
**symbol**	μ
**definition**	$\mu \in R$

Parameter: precision

**name**	precision
**type**	scalar
**symbol**	τ
**definition**	$\tau > 0$

Functions

PDF

$$\sqrt{\frac{\tau}{2\pi}}\frac{1}{x}e^{-\frac{\tau}{2}(\log x-\mu)^2}$$

PDF in R

```
sqrt(tau / (2*pi)) * (1/x) * exp(- (tau/2)*(log(x)-mu)^2 )
```

CDF

$$\frac{1}{2}+\frac{1}{2}\,\mathrm{erf}\left[\frac{\log x-\mu}{\sqrt{2/\tau}}\right]$$

CDF in R

```
1/2 + 1/2 * erf( (log(x)-mu) / sqrt(2/tau) )
```

Characteristics

Mean

$$e^{\mu+\frac{1}{2\tau}}$$

Median

$$e^{\mu}$$

Mode

$$e^{\mu-\frac{1}{\tau}}$$

Variance

$$e^{2\mu+\frac{1}{\tau}}[e^{\frac{1}{\tau}}-1]$$

Relationships

- Relationship pair: $LogNormal5(\mu,\tau) \to LogNormal2(\mu,v)$
- Relationship type: Reparameterisation
- Relationship definition: $\mu=\mu, v=1/\tau$

- Relationship pair: $LogNormal5(\mu,\tau) \to LogNormal3(m,\sigma)$
- Relationship type: Reparameterisation
- Relationship definition: $m=\exp(\mu), \sigma=1/\sqrt{\tau}$

- Relationship pair: $LogNormal5(\mu,\tau) \to LogNormal4(m,cv)$
- Relationship type: Reparameterisation
- Relationship definition: $m=\exp(\mu), cv=\sqrt{\exp(1/\tau)-1}$

- Relationship pair: $LogNormal5(\mu,\tau) \to LogNormal1(\mu,\sigma)$
- Relationship type: Reparameterisation
- Relationship definition: $\mu=\mu, \sigma=1/\sqrt{\tau}$

- Relationship pair: $LogNormal5(\mu,\tau) \to LogNormal6(m,\sigma_g)$
- Relationship type: Reparameterisation
- Relationship definition: $m=\exp(\mu), \sigma_g=\exp(1/\sqrt{\tau})$

- Relationship pair: $LogNormal3(m,\sigma) \to LogNormal5(\mu,\tau)$
- Relationship type: Reparameterisation
- Relationship definition: $\mu=\log(m), \tau=1/\sigma^2$

- Relationship pair: $LogNormal4(m,cv) \to LogNormal5(\mu,\tau)$
- Relationship type: Reparameterisation
- Relationship definition: $\mu=\log(m), \tau=1/\log(cv^2+1)$

- Relationship pair: $LogNormal1(\mu,\sigma) \to LogNormal5(\mu,\tau)$
- Relationship type: Reparameterisation
- Relationship definition: $\mu=\mu, \tau=1/\sigma^2$

- Relationship pair: $LogNormal6(m,\sigma_g) \to LogNormal5(\mu,\tau)$
- Relationship type: Reparameterisation
- Relationship definition: $\mu=\log(m), \tau=1/\log^2(\sigma_g)$

- Relationship pair: $LogNormal2(\mu,v) \to LogNormal5(\mu,\tau)$
- Relationship type: Reparameterisation
- Relationship definition: $\mu=\mu, \tau=1/v$

References

[Spiegelhalter et al., 2003], [Lunn, 2012], [Plummer, 2003]

LogNormal6

**name**	Log-Normal 6 (ID: 0000553)
**type**	continuous
**variate**	x, scalar
**support**	$x \in (0, +\infty)$

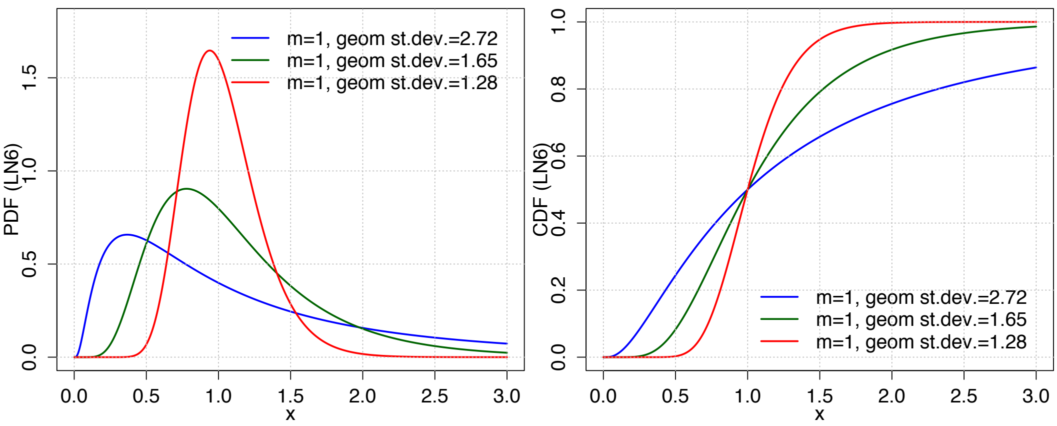

Figure 1.42: LogNormal6 distribution plotted using the provided R code.

Parameter: median

**name**	median / geometric mean
**type**	scalar
**symbol**	m
**definition**	$m > 0$

Parameter: geomStdev

**name**	shape
**type**	scalar
**symbol**	σ_g
**definition**	$\sigma_g > 0$

Functions

PDF

$$\frac{1}{x\log(\sigma_g)\sqrt{2\pi}}\exp\left[\frac{-[\log(x/m)]^2}{2\log^2(\sigma_g)}\right]$$

PDF in R

```
1/(x*log(sigma_g)*sqrt(2*pi))*exp(-(log(x/m))^2/(2*log(sigma_g)^2))
```

CDF

$$\frac{1}{2}+\frac{1}{2}\,\mathrm{erf}\left[\frac{\log x-\log m}{\sqrt{2}\log(\sigma_g)}\right]$$

CDF in R

```
1/2 + 1/2 * erf( (log(x)-log(m)) / (sqrt(2)*log(sigma_g)) )
```

Characteristics

Mean

$$m\,e^{\frac{1}{2}\log^2(\sigma_g)}$$

Median

$$m$$

Mode

$$m/e^{\log^2(\sigma_g)}$$

Variance

$$m^2e^{\log^2(\sigma_g)}[e^{\log^2(\sigma_g)}-1]$$

Relationships

- Relationship pair: $LogNormal6(m, \sigma_g) \rightarrow LogNormal1(\mu, \sigma)$
- Relationship type: Reparameterisation
- Relationship definition: $\mu = \log(m), \sigma = \log(\sigma_g)$

- Relationship pair: $LogNormal6(m, \sigma_g) \rightarrow LogNormal2(\mu, v)$
- Relationship type: Reparameterisation
- Relationship definition: $\mu = \log(m), v = \log(\sigma_g^2)$

- Relationship pair: $LogNormal6(m, \sigma_g) \rightarrow LogNormal3(m, \sigma)$
- Relationship type: Reparameterisation
- Relationship definition: $m = m, \sigma = \log(\sigma_g)$

- Relationship pair: $LogNormal6(m, \sigma_g) \rightarrow LogNormal4(m, cv)$
- Relationship type: Reparameterisation
- Relationship definition: $m = m, cv = \sqrt{\exp\big(\log^2(\sigma_g)\big) - 1}$

- Relationship pair: $LogNormal6(m, \sigma_g) \rightarrow LogNormal5(\mu, \tau)$
- Relationship type: Reparameterisation
- Relationship definition: $\mu = \log(m), \tau = 1/\log^2(\sigma_g)$

- Relationship pair: $LogNormal1(\mu, \sigma) \rightarrow LogNormal6(m, \sigma_g)$
- Relationship type: Reparameterisation
- Relationship definition: $m = \exp(\mu), \sigma_g = \exp(\sigma)$

- Relationship pair: $LogNormal2(\mu, v) \rightarrow LogNormal6(m, \sigma_g)$
- Relationship type: Reparameterisation
- Relationship definition: $m = \exp(\mu), \sigma_g = \exp(\sqrt{v})$

- Relationship pair: $LogNormal3(m, \sigma) \rightarrow LogNormal6(m, \sigma_g)$
- Relationship type: Reparameterisation
- Relationship definition: $m = m, \sigma_g = \exp(\sigma)$

- Relationship pair: $LogNormal4(m, cv) \rightarrow LogNormal6(m, \sigma_g)$
- Relationship type: Reparameterisation
- Relationship definition: $m = m, \sigma_g = \exp\big(\sqrt{\log(cv^2 + 1)}\big)$

- Relationship pair: $LogNormal5(\mu, \tau) \rightarrow LogNormal6(m, \sigma_g)$
- Relationship type: Reparameterisation
- Relationship definition: $m = \exp(\mu), \sigma_g = \exp(1/\sqrt{\tau})$

References

[Limpert et al., 2001]

LogUniform1

**name**	Log-Uniform 1 (ID: 0000580)
**type**	continuous
**variate**	x, scalar
**support**	$x \in (min, max)$

Parameter: minimum

**name**	minimum
**type**	scalar
**symbol**	min
**definition**	$min > 0$

Parameter: maximum

**name**	maximum
**type**	scalar
**symbol**	max
**definition**	$max \geq min$

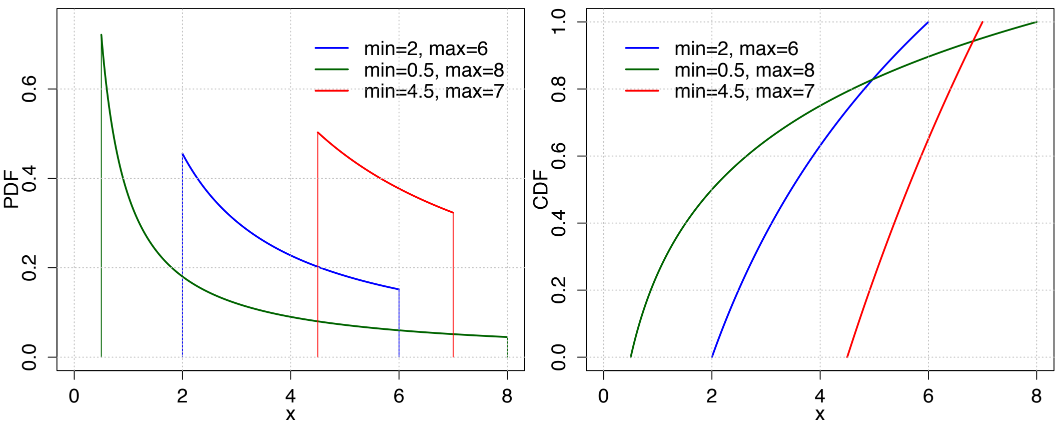

Figure 1.43: LogUniform1 distribution plotted using the provided R code.

Functions

PDF

$$\frac{1}{x(\log(max) - \log(min))}$$

PDF in R

```
1/(x*(log(max) - log(min)))
```

CDF

$$\frac{\log(x) - \log(min)}{\log(max) - \log(min)}$$

CDF in R

```
(log(x) - log(min)) / (log(max) - log(min))
```

Characteristics

Mean

$$\frac{max - min}{\log(max) - \log(min)}$$

Variance

$$\frac{max^2 - min^2}{2[\log(max) - \log(min)]} - \left(\frac{max - min}{\log(max) - \log(min)}\right)^2$$

References

http://www.vosesoftware.com/ModelRiskHelp/index.htm#Distributions/Continuous_distributions/LogUniform_distribution.htm

Logistic1

**name**	Logistic 1 (ID: 0000307)
**type**	continuous
**variate**	x, scalar
**support**	$x \in (-\infty, +\infty)$

Parameter: location

**name**	location
**type**	scalar
**symbol**	μ
**definition**	$\mu \in R$

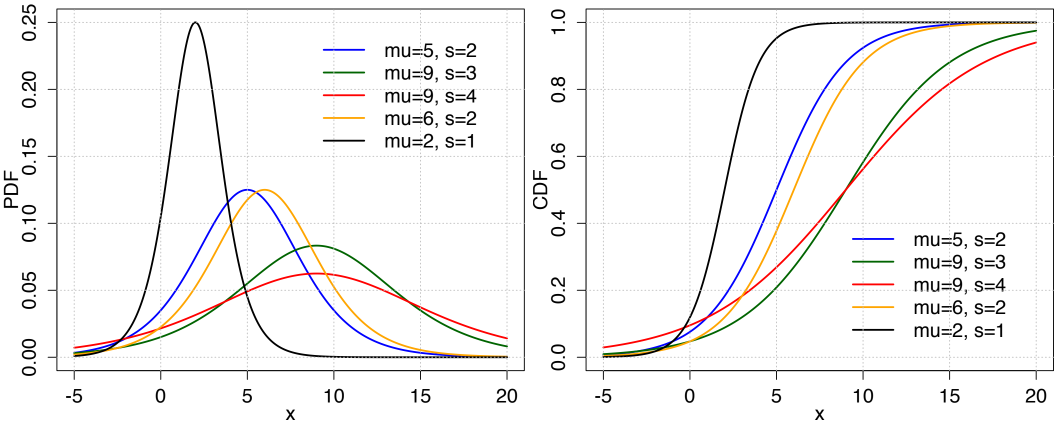

Figure 1.44: Logistic1 distribution plotted using the provided R code.

Parameter: scale

**name**	scale
**type**	scalar
**symbol**	s
**definition**	$s > 0, s \in R$

Functions

PDF

$$\frac{e^{-\frac{x-\mu}{s}}}{s\left(1+e^{-\frac{x-\mu}{s}}\right)^2}$$

PDF in R

```
exp(-(x-mu)/s) / (s*(1+exp(-(x-mu)/s))^2)
```

CDF

$$\frac{1}{1+e^{-\frac{x-\mu}{s}}}$$

CDF in R

```
1/(1+exp(-(x-mu)/s))
```

Characteristics

Mean

$$\mu$$

Median

$$\mu$$

Mode

$$\mu$$

Variance

$$\frac{s^2\pi^2}{3}$$

Relationships

- Relationship pair: $Logistic1(\mu, s) \to Logistic2(\mu, \tau)$
- Relationship type: Reparameterisation
- Relationship definition: $\tau = 1/s$

- Relationship pair: $LogLogistic1(\alpha, \beta) \to Logistic1(\mu, s)$
- Relationship type: Transformation
- Relationship definition: If $X \sim LogLogistic1(\alpha, \beta) \Rightarrow Y = log(X) \sim Logistic1(\mu, s)$ with $\mu = \log(\alpha), s = 1/\beta)$

- Relationship pair: $Logistic2(\mu, \tau) \to Logistic1(\mu, s)$
- Relationship type: Reparameterisation
- Relationship definition: $s = 1/\tau$

References

http://en.wikipedia.org/wiki/Logistic_distribution
http://www.uncertml.org/distributions/logistic

Logistic2

**name**	Logistic 2 (ID: 0000331)
**type**	continuous
**variate**	x, scalar
**support**	$x \in (-\infty, +\infty)$

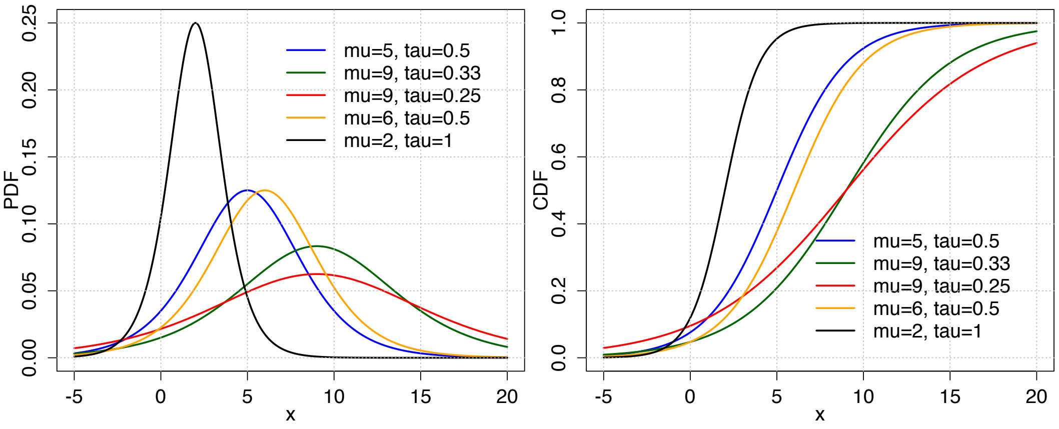

Figure 1.45: Logistic2 distribution plotted using the provided R code.

Parameter: location

**name**	location
**type**	scalar
**symbol**	μ
**definition**	$\mu \in R$

Parameter: inverseScale

**name**	inverse scale
**type**	scalar
**symbol**	τ
**definition**	$\tau > 0, \tau \in R$

Functions

PDF

$$\frac{\tau e^{-\tau(x-\mu)}}{\left(1+e^{-\tau(x-\mu)}\right)^2}$$

PDF in R

```
(tau * exp(-tau*(x-mu))) / (1+exp(-tau*(x-mu)))^2
```

CDF

$$\frac{1}{1+e^{-\tau(x-\mu)}}$$

CDF in R

```
1/(1+exp(-tau*(x-mu)))
```

Characteristics

Mean

$$\mu$$

Median

$$\mu$$

Mode

$$\mu$$

Variance

$$\frac{\pi^2}{3\tau^2}$$

Relationships

- Relationship pair: $Logistic2(\mu,\tau) \rightarrow Logistic1(\mu,s)$
- Relationship type: Reparameterisation
- Relationship definition: $s = 1/\tau$

- Relationship pair: $Logistic1(\mu,s) \rightarrow Logistic2(\mu,\tau)$
- Relationship type: Reparameterisation
- Relationship definition: $\tau = 1/s$

References

[Spiegelhalter et al., 2003]

MixtureDistribution1

**name**	Mixture Distribution 1 (ID: 0000630)
**type**	continuous
**variate**	$-$, -
**support**	$-$

Parameter: weight

**name**	mixing coefficients
**type**	vector
**symbol**	$\pi_1,\ldots,\pi_k$
**definition**	$\Sigma_{i=1}^{K}\pi_i = 1; 0 \le \pi_i \le 1$

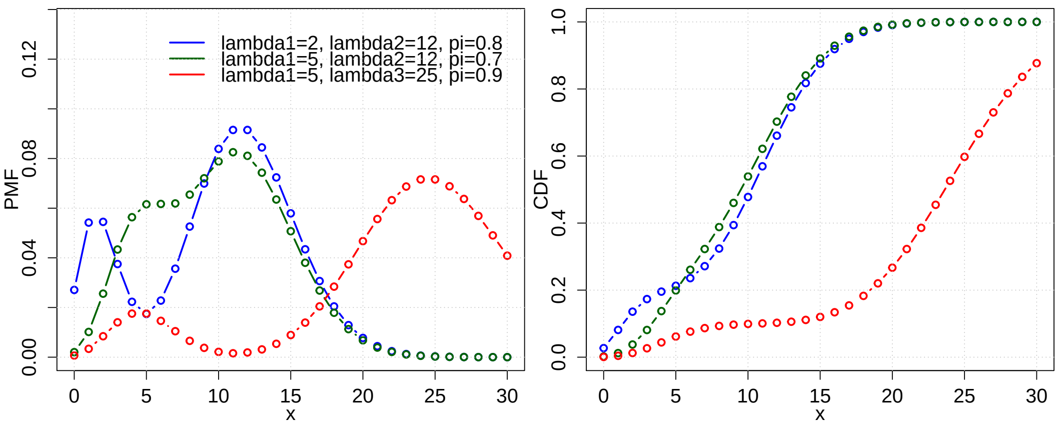

Figure 1.46: Example 1: PMF and CDF of the Mixture Poisson distribution plotted using the formula for for various values as shown in the legend of the left plot. The PMF reads: $(1-\pi_1)\,\lambda_1^k/k!\,\exp(-\lambda_1) + \pi_1\,\lambda_2^k/k!\,\exp(-\lambda_2)$. The CDF reads: $(1-\pi_1)\,\Gamma(\lfloor k+1,\lambda_1\rfloor)/\lfloor k\rfloor! + \pi_1\,\Gamma(\lfloor k+1,\lambda_2\rfloor)/\lfloor k\rfloor!$.

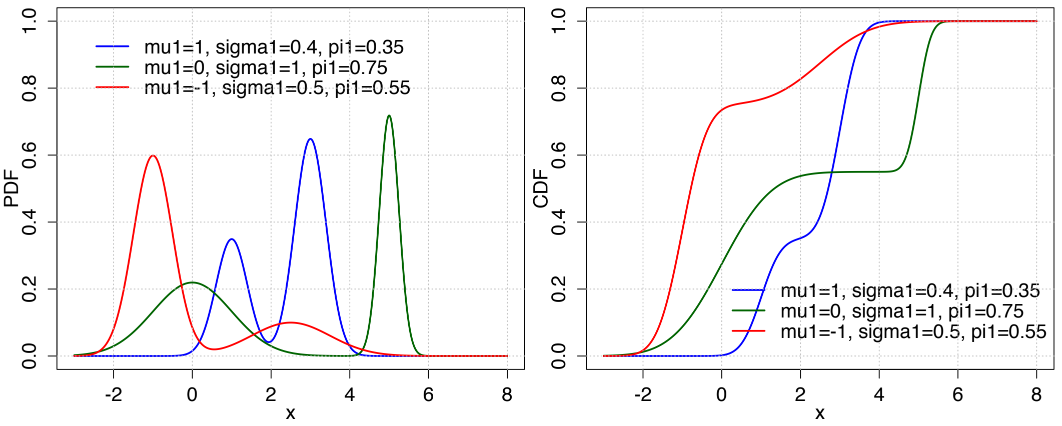

Figure 1.47: Example 2: PDF and CDF of the Mixture Normal distribution plotted using the formula for for various values as shown in the legend of the left plot. The PDF reads: $(1-\pi_1)\times 1/(\sigma_1\sqrt{2\pi})\exp(-(x-\mu_1)^2/(2\sigma_1^2)) + \pi_1 \times 1/(\sigma_2\sqrt{2\pi})\exp(-(x-\mu_2)^2/(2\sigma_2^2))$. The CDF reads: $(1-\pi_1)\times 1/2(1+erf((x-\mu_1)/(\sigma_1\sqrt{2}))) + \pi_1 \times 1/2(1+erf((x-\mu_2)/(\sigma_2\sqrt{2})))$.

Functions

PDF

$$f(x;\pi,\theta) = \sum_{i=1}^{K} \pi_i\, p_i(x;\theta_i) \text{ where } p_i(x;\theta_i) \text{ the PDF of the } i^{th} \text{ component with parameters } \theta_i$$

References

[Forbes et al., 2011]
https://en.wikipedia.org/wiki/Mixture_distribution
http://www.uncertml.org/distributions/mixture-model

Multinomial1

**name**	Multinomial 1 (ID: 0000654)
**type**	discrete
**variate**	X, vector
**support**	$X_i \in \{0,\dots,n\}, \Sigma X_i = n$

Model

For n independent trials each of which leads to a success for exactly one of k categories, with each category having a given fixed success probability, the multinomial distribution gives the probability of any particular combination of numbers of successes for the various categories.

Parameter: numberOfTrials

**name**	number of trials
**type**	scalar
**symbol**	n
**definition**	$n > 0, n \in N$

Parameter: probabilityOfSuccess

**name**	event probabilities
**type**	vector
**symbol**	$p_1, \ldots, p_k$
**definition**	$p_1, \ldots, p_k, \Sigma p_i = 1$

Functions

PMF

$$\frac{n!}{x_1! \cdots x_k!} p_1^{x_1} \cdots p_k^{x_k}$$

Characteristics

Mean

$$E\{X_i\} = np_i$$

Variance

$$Var(X_i) = np_i(1 - p_i); \quad Cov(X_i, X_j) = -np_ip_j \quad (i \neq j)$$

References

http://en.wikipedia.org/wiki/Multinomial_distribution
http://www.uncertml.org/distributions/multinomial

MultivariateNormal1

**name**	Multivariate Normal 1 (ID: 0000719)
**type**	continuous
**variate**	x, vector
**support**	$-\infty < x_i < \infty, i = 1, ..., k$

Parameter: mean

**name**	location
**type**	vector
**symbol**	μ
**definition**	$\mu \in R^k$

Parameter: covarianceMatrix

**name**	covariance matrix
**type**	matrix
**symbol**	Σ
**definition**	$\Sigma \in R^{k \times k}$

Functions

PDF

$$(2\pi)^{-\frac{k}{2}} |\Sigma|^{-\frac{1}{2}}\, e^{-\frac{1}{2}(x-\mu)'\Sigma^{-1}(x-\mu)}$$

CDF

no analytic expression

Characteristics

Mean

$$\mu$$

Mode

$$\mu$$

Variance

$$\Sigma$$

References

[Forbes et al., 2011]
`http://en.wikipedia.org/wiki/Multivariate_normal_distribution`
`http://www.uncertml.org/distributions/multivariate-normal`

MultivariateNormal2

**name**	Multivariate Normal 2 (ID: 0000742)
**type**	continuous
**variate**	x, vector
**support**	$x \in R^k$

Parameter: mean

**name**	location
**type**	vector
**symbol**	μ
**definition**	$\mu \in R^k$

Parameter: precisionMatrix

**name**	precision matrix
**type**	matrix
**symbol**	T
**definition**	inverse of the covariance matrix

Functions

PDF

$$(2\pi)^{-d/2}|T|^{\frac{1}{2}}\ \exp\big(-\frac{1}{2}(x-\mu)'T(x-\mu)\big)$$

CDF

no analytic expression

Characteristics

Mean

$$\mu$$

Mode

$$\mu$$

Variance

$$T^{-1}$$

References

[Spiegelhalter et al., 2003]

MultivariateStudentT1

**name**	Multivariate (Student) T 1 (ID: 0000790)
**type**	continuous
**variate**	x, vector
**support**	$x \in R^p$

Parameter: mean

**name**	location
**type**	vector
**symbol**	μ
**definition**	$\mu = [\mu_1, \ldots, \mu_p]^T, \mu_i \in R$

Parameter: scaleMatrix

**name**	scale matrix
**type**	matrix
**symbol**	Σ
**definition**	Σ, positive-definite real $p \times p$ matrix

Parameter: degreesOfFreedom

**name**	degrees of freedom
**type**	scalar
**symbol**	ν
**definition**	$\nu \geq 2$

Functions

PDF

$$\frac{\Gamma\left[(\nu+p)/2\right]}{\Gamma(\nu/2)\nu^{p/2}\pi^{p/2}\left|\Sigma\right|^{1/2}\left[1+\frac{1}{\nu}(x-\mu)^{\mathrm{T}}\Sigma^{-1}(x-\mu)\right]^{(\nu+p)/2}}$$

CDF

no analytic expression

Characteristics

Mean

$$\begin{cases} \mu & \text{for } \nu > 1 \\ undefined & \text{else} \end{cases}$$

Median

$$\mu$$

Mode

$$\mu$$

Variance

$$\begin{cases} \frac{\nu}{\nu-2}\Sigma & \text{for } \nu > 2 \\ undefined & \text{else} \end{cases}$$

References

http://en.wikipedia.org/wiki/Multivariate_t-distribution
http://www.uncertml.org/distributions/multivariate-student-t

MultivariateStudentT2

**name**	Multivariate (Student) T 2 (ID: 0000016)
**type**	continuous
**variate**	x, vector
**support**	$x \in R^d, k \geq 2$

Parameter: mean

**name**	location
**type**	vector
**symbol**	μ
**definition**	$\mu = [\mu_1, \ldots, \mu_d]^T, \mu_i \in R$

Parameter: precisionMatrix

**name**	precision matrix
**type**	matrix
**symbol**	T
**definition**	Inverse of the covariance matrix

Parameter: degreesOfFreedom

**name**	degrees of freedom
**type**	scalar
**symbol**	k
**definition**	$k \geq 2$

Functions

PDF

$$\frac{\Gamma((k+d)/2)}{\Gamma(k/2)k^{d/2}\pi^{d/2}}|T|^{1/2}\Big[1+\frac{1}{k}(x-\mu)'T(x-\mu)\Big]^{-(k+d)/2}$$

References

[Spiegelhalter et al., 2003]

Nakagami1

**name**	Nakagami 1 (ID: 0000041)
**type**	continuous
**variate**	x, scalar
**support**	$x \in (0, +\infty)$

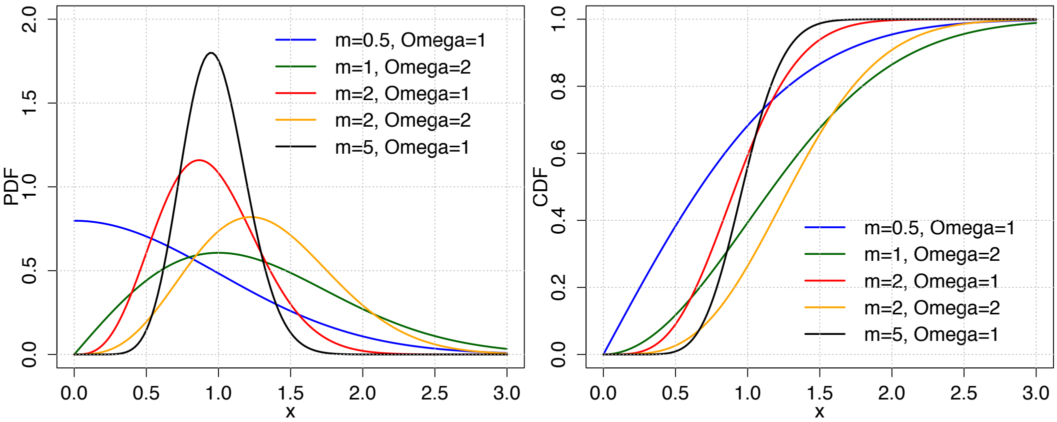

Figure 1.48: Nakagami1 distribution plotted using the provided R code.

Parameter: shape

**name**	shape
**type**	scalar
**symbol**	m
**definition**	$m > 0$

Parameter: spread

**name**	spread
**type**	scalar
**symbol**	Ω
**definition**	$\Omega > 0$

Functions

PDF

$$\frac{2m^m}{\Gamma(m)\Omega^m}x^{2m-1}\exp(-\frac{m}{\omega}x^2)$$

PDF in R

```
2*m^m / (gamma(m)*Omega^m)*x^(2*m-1)*exp(-m/Omega*x^2)
```

CDF

$$\frac{\gamma(m, \frac{m}{\Omega}x^2)}{\Gamma(m)}$$

CDF in R

```
Igamma(m,m/Omega*x^2,lower=T)/gamma(m)
```

Characteristics

Mean

$$\frac{\Gamma(m+\frac{1}{2})}{\Gamma(m)}\left(\frac{\Omega}{m}\right)^{\frac{1}{2}}$$

Median

$$\sqrt{\Omega}$$

Mode

$$\frac{\sqrt{2}}{2}\left(\frac{(2m-1)\Omega}{m}\right)^{1/2}$$

Variance

$$\Omega\left(1-\frac{1}{m}\left(\frac{\Gamma(m+\frac{1}{2})}{\Gamma(m)}\right)^{2}\right)$$

References

`https://en.wikipedia.org/wiki/Nakagami_distribution`

NegativeBinomial1

**name**	Negative Binomial 1 (ID: 0000074)
**type**	discrete
**variate**	k, scalar
**support**	$k \in \{0, 1, 2, 3, \dots\}$ – number of failures

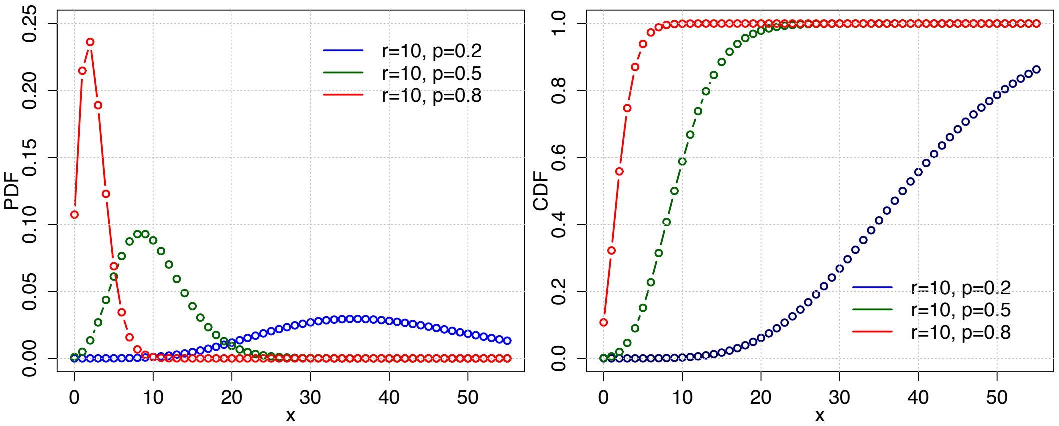

Figure 1.49: NegativeBinomial1 distribution plotted using the provided R code.

Model

Negative Binomial 1 distribution is a discrete probability distribution of the number of failures before the rth success in repeated mutually independent Bernoulli trials, each with probability of success p. (Compare Negative Binomial 4.)

Parameter: numberOfSuccesses

**name**	number of successes
**type**	scalar
**symbol**	r
**definition**	$r > 0, r \in N$

Parameter: probability

**name**	success probability
**type**	scalar
**symbol**	p
**definition**	$p \in (0, 1)$

Functions

PMF

$$\binom{k+r-1}{k} p^r (1-p)^k$$

PMF in R

```
choose(k+r-1,k) * p^r * (1-p)^k
```

CDF

$$1 - I_{1-p}(k+1, r)$$

CDF in R

```
1 - Rbeta(1-p, k+1, r, lower = T)
```

Characteristics

Mean

$$\frac{r(1-p)}{p}$$

Mode

$$\lfloor \frac{(1-p)(r-1)}{p} \rfloor$$

Variance

$$\frac{r(1-p)}{p^2}$$

Relationships

- Relationship pair: $NegativeBinomial1(r, p) \rightarrow NegativeBinomial5(\alpha, \beta)$
- Relationship type: Reparameterisation
- Relationship definition: $\alpha = r, \beta = p/(1-p)$

- Relationship pair: $NegativeBinomial1(r, p) \rightarrow NegativeBinomial4(r, p)$
- Relationship type: Reparameterisation
- Relationship definition: $p = 1 - p$

- Relationship pair: $NegativeBinomial1(r, p) \rightarrow Geometric1(p)$
- Relationship type: Special case
- Relationship definition: $n = 1$

- Relationship pair: $NegativeBinomial1(r, p) \rightarrow Normal1(\mu, \sigma)$
- Relationship type:
- Relationship definition: $\mu = n(1-p), n \rightarrow \infty$

- Relationship pair: $NegativeBinomial1(r, p) \rightarrow Poisson1(\lambda)$
- Relationship type:
- Relationship definition: $\mu = np, n \rightarrow \infty$

- Relationship pair: $NegativeBinomial1(r, p) \rightarrow NegativeBinomial3(\mu, \phi)$
- Relationship type: Reparameterisation
- Relationship definition: $\phi = r, \mu = r(1-p)/p$

- Relationship pair: $NegativeBinomial1(r, p) \rightarrow NegativeBinomial2(\lambda, \tau)$
- Relationship type: Reparameterisation
- Relationship definition: $\tau = 1/r, \lambda = r(1-p)/p$

- Relationship pair: $NegativeBinomial5(\alpha, \beta) \rightarrow NegativeBinomial1(r, p)$
- Relationship type: Reparameterisation
- Relationship definition: $r = \alpha, p = \beta/(1+\beta)$

- Relationship pair: $NegativeBinomial4(r, p) \rightarrow NegativeBinomial1(r, p)$
- Relationship type: Reparameterisation
- Relationship definition: $p = 1 - p$

- Relationship pair: $Geometric1(p) \rightarrow NegativeBinomial1(r, p)$

- Relationship type: Transformation
- Relationship definition: $\Sigma X(iid)$

- Relationship pair: $NegativeBinomial3(\mu, \phi) \rightarrow NegativeBinomial1(r, p)$
- Relationship type: Reparameterisation
- Relationship definition: $r = \phi, p = r/(\mu + r)$

- Relationship pair: $NegativeBinomial2(\lambda, \tau) \rightarrow NegativeBinomial1(r, p)$
- Relationship type: Reparameterisation
- Relationship definition: $r = 1/\tau, p = 1/(1 + \tau\lambda)$

- Relationship pair: $ConwayMaxwellPoisson1(\lambda, \nu) \rightarrow NegativeBinomial1(r, p)$
- Relationship type: Transformation
- Relationship definition: For $\nu = 0$ and $\lambda < 1$ the sum of Conway-Maxwell-Poisson distributed variables reduces to the sum of geometric variables, which follows a Negative Binomial distribution with parameters n and $1 - \lambda$

References

[Shmueli et al., 2005], [Leemis and Mcqueston, 2008]
http://www.math.wm.edu/~leemis/chart/UDR/PDFs/PascalGeometric.pdf
http://www.math.wm.edu/~leemis/chart/UDR/PDFs/GeometricPascal.pdf
http://www.math.wm.edu/~leemis/chart/UDR/PDFs/PascalNormal.pdf
http://www.math.wm.edu/~leemis/chart/UDR/PDFs/PascalPoisson.pdf

NegativeBinomial2

**name**	Negative Binomial 2 (ID: 0000105)
**type**	discrete
**variate**	k, scalar
**support**	$k \in \{0, 1, 2, 3, \dots\}$

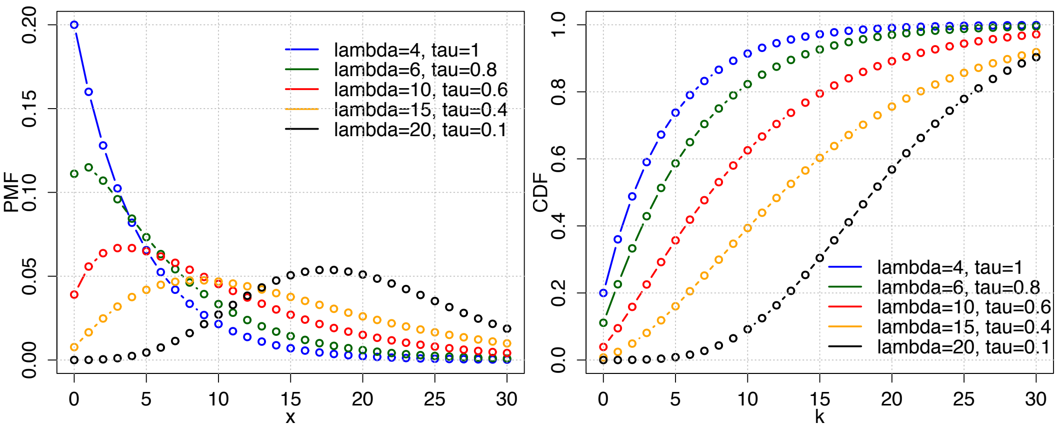

Figure 1.50: NegativeBinomial2 distribution plotted using the provided R code.

Parameter: rate

**name**	Poisson intensity
**type**	scalar
**symbol**	λ
**definition**	$\lambda \in R, \lambda > 0$

Parameter: overdispersion

**name**	overdispersion
**type**	scalar
**symbol**	τ
**definition**	$\tau \in R$

Functions

PMF

$$\frac{\Gamma(k+\frac{1}{\tau})}{k!\,\Gamma(\frac{1}{\tau})}\Big(\frac{1}{1+\tau\lambda}\Big)^{\frac{1}{\tau}}\Big(\frac{\lambda}{\frac{1}{\tau}+\lambda}\Big)^{k}$$

PMF in R

```
gamma(k + 1/tau)/(factorial(k) * gamma(1/tau)) * 1/(1+tau*lambda)^(1/tau) *
(lambda/(1/tau + lambda))^k
```

CDF

$$\Sigma_{i=1}^{x} f(i), x \in \{0,1,2,...\} \text{ with } f \text{ the PMF}$$

CDF in R

```
cumsum(PMF)
```

Characteristics

Mean

$$\lambda$$

Variance

$$\lambda(1+\tau\lambda)$$

Relationships

- Relationship pair: $NegativeBinomial2(\lambda,\tau) \to NegativeBinomial5(\alpha,\beta)$
- Relationship type: Reparameterisation
- Relationship definition: $\alpha = 1/\tau, \beta = 1/(\tau\lambda)$

- Relationship pair: $NegativeBinomial2(\lambda,\tau) \to NegativeBinomial4(r,p)$
- Relationship type: Reparameterisation
- Relationship definition: $r = 1/\tau, p = \frac{\tau\lambda}{1+\tau\lambda}$

- Relationship pair: $NegativeBinomial2(\lambda,\tau) \to NegativeBinomial3(\mu,\phi)$
- Relationship type: Reparameterisation
- Relationship definition: $\mu = \lambda, \phi = 1/\tau$

- Relationship pair: $NegativeBinomial2(\lambda,\tau) \to NegativeBinomial1(r,p)$
- Relationship type: Reparameterisation
- Relationship definition: $r = 1/\tau, p = 1/(1+\tau\lambda)$

- Relationship pair: $NegativeBinomial5(\alpha,\beta) \to NegativeBinomial2(\lambda,\tau)$
- Relationship type: Reparameterisation
- Relationship definition: $\lambda = \alpha/\beta, \tau = 1/\alpha$

- Relationship pair: $ZeroInflatedNegativeBinomial1(\lambda,\tau,p0) \to NegativeBinomial2(\lambda,\tau)$
- Relationship type: Special case
- Relationship definition: $p0 = 0$

- Relationship pair: $NegativeBinomial4(r,p) \to NegativeBinomial2(\lambda,\tau)$
- Relationship type: Reparameterisation
- Relationship definition: $\tau = 1/r, \lambda = \frac{rp}{1-p}$

- Relationship pair: $NegativeBinomial3(\mu,\phi) \to NegativeBinomial2(\lambda,\tau)$
- Relationship type: Reparameterisation
- Relationship definition: $\lambda = \mu, \tau = 1/\phi$

- Relationship pair: $NegativeBinomial1(r,p) \to NegativeBinomial2(\lambda,\tau)$
- Relationship type: Reparameterisation
- Relationship definition: $\tau = 1/r, \lambda = r(1-p)/p$

References

[Trocóniz et al., 2009], [Cameron and Trivedi, 2013], [Hilbe, 2011]

NegativeBinomial3

**name**	Negative Binomial 3 (ID: 0000135)
**type**	discrete
**variate**	k, scalar
**support**	$k \in \{0, 1, 2, 3, \dots\}$

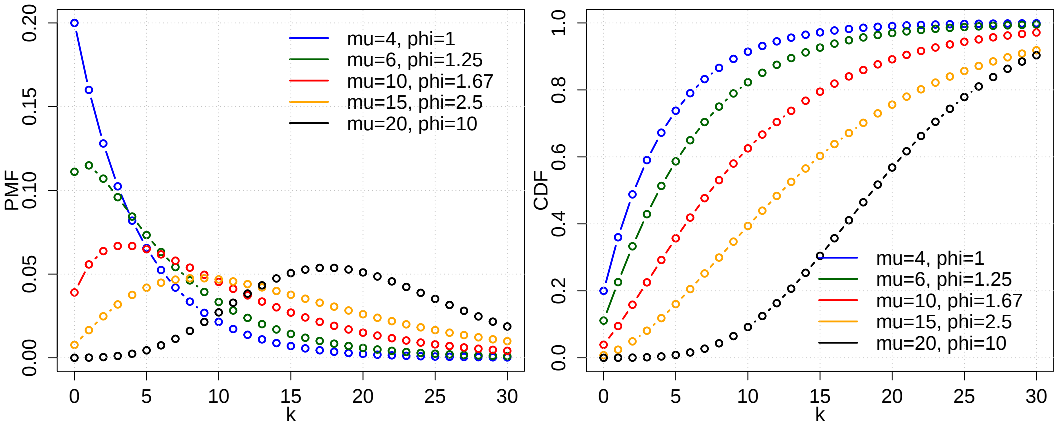

Figure 1.51: NegativeBinomial3 distribution plotted using the provided R code.

Parameter: mean

**name**	mean
**type**	scalar
**symbol**	μ
**definition**	$\mu \in R, \mu > 0$

Parameter: dispersion

**name**	index parameter
**type**	scalar
**symbol**	ϕ
**definition**	$\phi \in R, \phi > 0$

Functions

PMF

$$\binom{k+\phi-1}{k}\left(\frac{\phi}{\mu+\phi}\right)^{\phi}\left(\frac{\mu}{\mu+\phi}\right)^{k}$$

PMF in R

```
choose(k+phi-1,k) * (phi / (mu + phi))^phi * (mu / (mu + phi))^k
```

CDF

$$-$$

Characteristics

Mean

$$\mu$$

Variance

$$\mu + \mu^2/\phi$$

Relationships

- Relationship pair: $NegativeBinomial3(\mu, \phi) \to NegativeBinomial5(\alpha, \beta)$
- Relationship type: Reparameterisation
- Relationship definition: $\alpha = \phi, \beta = \phi/\mu$

- Relationship pair: $NegativeBinomial3(\mu, \phi) \to NegativeBinomial4(r, p)$
- Relationship type: Reparameterisation
- Relationship definition: $r = \phi, p = \mu/(\phi + \mu)$

- Relationship pair: $NegativeBinomial3(\mu, \phi) \to NegativeBinomial1(r, p)$
- Relationship type: Reparameterisation
- Relationship definition: $r = \phi, p = r/(\mu + r)$

- Relationship pair: $NegativeBinomial3(\mu, \phi) \to NegativeBinomial2(\lambda, \tau)$
- Relationship type: Reparameterisation
- Relationship definition: $\lambda = \mu, \tau = 1/\phi$

- Relationship pair: $NegativeBinomial5(\alpha, \beta) \to NegativeBinomial3(\mu, \phi)$
- Relationship type: Reparameterisation
- Relationship definition: $\mu = \alpha/\beta, \phi = \alpha$

- Relationship pair: $NegativeBinomial4(r, p) \to NegativeBinomial3(\mu, \phi)$
- Relationship type: Reparameterisation
- Relationship definition: $\phi = r, \mu = rp/(1 - p)$

- Relationship pair: $NegativeBinomial1(r, p) \to NegativeBinomial3(\mu, \phi)$
- Relationship type: Reparameterisation
- Relationship definition: $\phi = r, \mu = r(1 - p)/p$

- Relationship pair: $NegativeBinomial2(\lambda, \tau) \to NegativeBinomial3(\mu, \phi)$
- Relationship type: Reparameterisation
- Relationship definition: $\mu = \lambda, \phi = 1/\tau$

References

[STAN Development Team, 2015]
`cran.r-project.org/web/packages/VGAM/VGAM.pdf`

NegativeBinomial4

**name**	Negative Binomial 4 (ID: 0000161)
**type**	discrete
**variate**	k, scalar
**support**	$k \in \{0, 1, 2, 3, \dots\}$ – number of successes

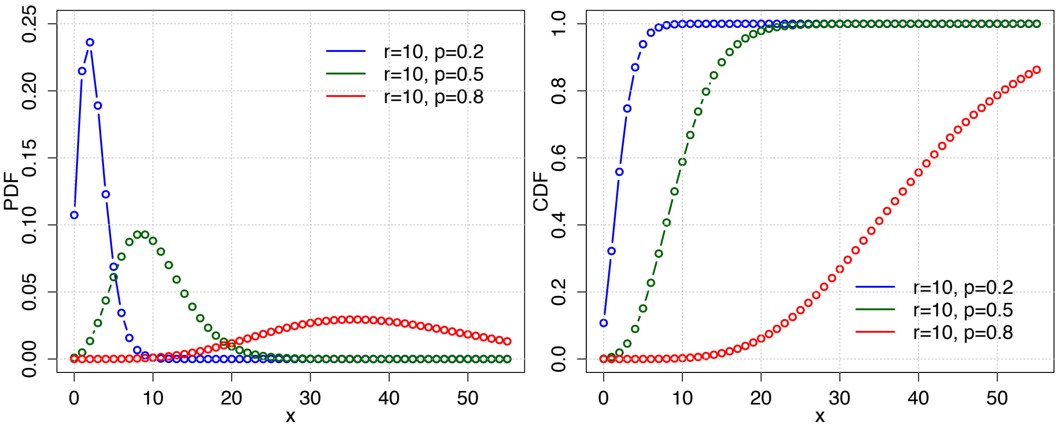

Figure 1.52: NegativeBinomial4 distribution plotted using the provided R code.

Model

Negative Binomial 4 distribution is a discrete probability distribution of the number of successes in a sequence of independent and identically distributed Bernoulli trials before a specified (non-random) number of failures r occurs. (Compare Negative Binomial 1.)

Parameter: numberOfFailures

**name**	number of failures
**type**	scalar
**symbol**	r
**definition**	$r > 0, r \in N$

Parameter: probability

**name**	success probability
**type**	scalar
**symbol**	p
**definition**	$p \in (0, 1)$

Functions

PMF

$$\binom{k+r-1}{k}(1-p)^r p^k$$

PMF in R

```
choose(k+r-1,k) * (1-p)^r * p^k
```

CDF

$$1 - I_p(k+1, r)$$

CDF in R

```
1 - Rbeta(p, k+1, r,lower = T)
```

Characteristics

Mean

$$\frac{pr}{1-p}$$

Mode

$$\begin{cases} \lfloor \frac{p(r-1)}{1-p} \rfloor & \text{for } r > 1 \\ 0 & \text{for } r \leq 1 \end{cases}$$

Variance

$$\frac{pr}{(1-p)^2}$$

Relationships

- Relationship pair: $NegativeBinomial4(r, p) \rightarrow NegativeBinomial2(\lambda, \tau)$
- Relationship type: Reparameterisation
- Relationship definition: $\tau = 1/r, \lambda = \frac{rp}{1-p}$

- Relationship pair: $NegativeBinomial4(r, p) \rightarrow NegativeBinomial1(r, p)$
- Relationship type: Reparameterisation
- Relationship definition: $p = 1 - p$

- Relationship pair: $NegativeBinomial4(r, p) \rightarrow NegativeBinomial5(\alpha, \beta)$
- Relationship type: Reparameterisation
- Relationship definition: $\alpha = r, \beta = (1-p)/p$

- Relationship pair: $NegativeBinomial4(r, p) \rightarrow NegativeBinomial3(\mu, \phi)$
- Relationship type: Reparameterisation

- Relationship definition: $\phi = r, \mu = rp/(1-p)$
- Relationship pair: $NegativeBinomial2(\lambda, \tau) \rightarrow NegativeBinomial4(r, p)$
- Relationship type: Reparameterisation
- Relationship definition: $r = 1/\tau, p = \frac{\tau\lambda}{1+\tau\lambda}$
- Relationship pair: $NegativeBinomial1(r, p) \rightarrow NegativeBinomial4(r, p)$
- Relationship type: Reparameterisation
- Relationship definition: $p = 1 - p$
- Relationship pair: $GeneralizedNegativeBinomial1(\theta, \beta, m) \rightarrow NegativeBinomial4(r, p)$
- Relationship type:
- Relationship definition: $\beta = 1$ and set $m = r, \theta = p$
- Relationship pair: $NegativeBinomial5(\alpha, \beta) \rightarrow NegativeBinomial4(r, p)$
- Relationship type: Reparameterisation
- Relationship definition: $r = \alpha, p = 1/(1+\beta)$
- Relationship pair: $NegativeBinomial3(\mu, \phi) \rightarrow NegativeBinomial4(r, p)$
- Relationship type: Reparameterisation
- Relationship definition: $r = \phi, p = \mu/(\phi + \mu)$

References

[Cameron and Trivedi, 2013], [Consul and Famoye, 2006]
`https://en.wikipedia.org/wiki/Binomial_distribution`
`http://www.uncertml.org/distributions/negative-binomial`

NegativeBinomial5

**name**	Negative Binomial 5 (ID: 0000190)
**type**	discrete
**variate**	k, scalar
**support**	$k \in \{0, 1, 2, 3, \dots\}$

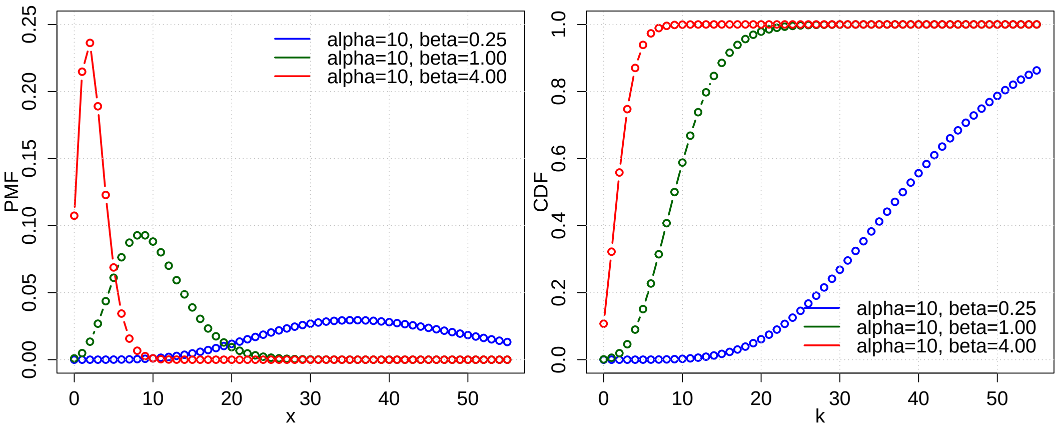

Figure 1.53: NegativeBinomial5 distribution plotted using the provided R code.

Parameter: shape

**name**	shape
**type**	scalar
**symbol**	α
**definition**	$\alpha \in R^+$

Parameter: inverseScale

**name**	inverse scale
**type**	scalar
**symbol**	β
**definition**	$\beta \in R^+$

Functions

PMF

$$\binom{k+\alpha-1}{\alpha-1}\Big(\frac{\beta}{\beta+1}\Big)^{\alpha}\Big(\frac{1}{\beta+1}\Big)^{k}$$

PMF in R

```
choose(k+alpha-1,alpha-1) * (beta / (beta + 1))^alpha * (1 / (beta + 1))^k
```

CDF

$$\Sigma_{i=1}^{x} f(i), x \in \{0,1,2,...\} \text{ with } f \text{ the PMF}$$

CDF in R

```
cumsum(PMF)
```

Characteristics

Mean

$$\alpha/\beta$$

Variance

$$\alpha/\beta^2(\beta+1)$$

Relationships

- Relationship pair: $NegativeBinomial5(\alpha,\beta) \rightarrow NegativeBinomial2(\lambda,\tau)$
- Relationship type: Reparameterisation
- Relationship definition: $\lambda = \alpha/\beta, \tau = 1/\alpha$

- Relationship pair: $NegativeBinomial5(\alpha,\beta) \rightarrow NegativeBinomial3(\mu,\phi)$
- Relationship type: Reparameterisation
- Relationship definition: $\mu = \alpha/\beta, \phi = \alpha$

- Relationship pair: $NegativeBinomial5(\alpha,\beta) \rightarrow NegativeBinomial1(r,p)$
- Relationship type: Reparameterisation
- Relationship definition: $r = \alpha, p = \beta/(1+\beta)$

- Relationship pair: $NegativeBinomial5(\alpha,\beta) \rightarrow NegativeBinomial4(r,p)$
- Relationship type: Reparameterisation
- Relationship definition: $r = \alpha, p = 1/(1+\beta)$

- Relationship pair: $NegativeBinomial2(\lambda,\tau) \rightarrow NegativeBinomial5(\alpha,\beta)$
- Relationship type: Reparameterisation
- Relationship definition: $\alpha = 1/\tau, \beta = 1/(\tau\lambda)$

- Relationship pair: $NegativeBinomial3(\mu,\phi) \rightarrow NegativeBinomial5(\alpha,\beta)$
- Relationship type: Reparameterisation
- Relationship definition: $\alpha = \phi, \beta = \phi/\mu$

- Relationship pair: $NegativeBinomial1(r,p) \rightarrow NegativeBinomial5(\alpha,\beta)$
- Relationship type: Reparameterisation
- Relationship definition: $\alpha = r, \beta = p/(1-p)$

- Relationship pair: $NegativeBinomial4(r,p) \rightarrow NegativeBinomial5(\alpha,\beta)$
- Relationship type: Reparameterisation
- Relationship definition: $\alpha = r, \beta = (1-p)/p$

References

[Gelman et al., 2014]

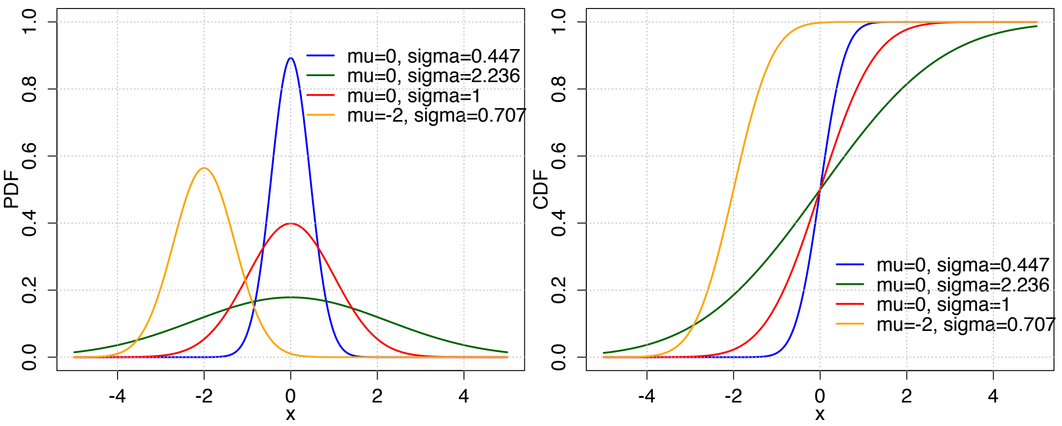

Figure 1.54: Normal1 distribution plotted using the provided R code.

Normal1

**name**	Normal 1 (ID: 0000239)
**type**	continuous
**variate**	x, scalar
**support**	$x \in R$

Parameter: mean

**name**	mean
**type**	scalar
**symbol**	μ
**definition**	$\mu \in R$

Parameter: stdev

**name**	standard deviation
**type**	scalar
**symbol**	σ
**definition**	$\sigma > 0$

Functions

PDF

$$\frac{1}{\sigma\sqrt{2\pi}}e^{-\frac{(x-\mu)^2}{2\sigma^2}}$$

PDF in R

```
1/(sigma*sqrt(2*pi))*exp(-(x-mu)^2/(2*sigma^2))
```

CDF

$$\frac{1}{2}\left[1+\mathrm{erf}\left(\frac{x-\mu}{\sigma\sqrt{2}}\right)\right]$$

CDF in R

```
1/2 * (1 + erf((x-mu)/(sigma*sqrt(2))))
```

Characteristics

Mean

$$\mu$$

Median

$$\mu$$

Mode

$$\mu$$

Variance

$$\sigma^2$$

Relationships

- Relationship pair: $Normal1(\mu, \sigma) \rightarrow LogNormal1(\mu, \sigma)$
- Relationship type: Transformation
- Relationship definition: $\exp(X)$

- Relationship pair: $Normal1(\mu, \sigma) \rightarrow StandardNormal1(0, 1)$
- Relationship type: Special case
- Relationship definition: $\mu = 0, \sigma = 1$

- Relationship pair: $Normal1(\mu, \sigma) \rightarrow Normal2(\mu, v)$
- Relationship type: Reparameterisation
- Relationship definition: $\mu = \mu, v = \sigma^2$

- Relationship pair: $Normal1(\mu, \sigma) \rightarrow Normal3(\mu, \tau)$
- Relationship type: Reparameterisation
- Relationship definition: $\mu = \mu, \tau = 1/\sigma^2$

- Relationship pair: $Normal1(\mu, \sigma) \rightarrow StandardNormal1(0, 1)$
- Relationship type: Transformation
- Relationship definition: $X \sim Normal1(\mu, \sigma); Y = (X - \mu)/\sigma; Y \sim StandardNormal1$

- Relationship pair: $Normal1(\mu, \sigma) \rightarrow ChiSquared1(n)$
- Relationship type: Transformation
- Relationship definition: If $X_i \sim N(\mu, \sigma), i = 1, 2, ..., n$ are mutually independent and identically distributed random variables and $Y = \sum_{i=1}^{n}((X_i - \mu)/\sigma)^2 \Rightarrow Y \sim ChiSquared1(n)$

- Relationship pair: $Poisson1(\lambda) \rightarrow Normal1(\mu, \sigma)$
- Relationship type: Transformation & Limiting
- Relationship definition: $\sigma^2 = \lambda, \mu = \lambda, \lambda \rightarrow \infty$

- Relationship pair: $LogNormal1(\mu, \sigma) \rightarrow Normal1(\mu, \sigma)$
- Relationship type: Transformation
- Relationship definition: $\log(X)$

- Relationship pair: $Normal2(\mu, v) \rightarrow Normal1(\mu, \sigma)$
- Relationship type: Reparameterisation
- Relationship definition: $\mu = \mu, \sigma = \sqrt{v}$

- Relationship pair: $TruncatedNormal1(\mu, \sigma, a, b) \rightarrow Normal1(\mu, \sigma)$
- Relationship type: Special case
- Relationship definition: $a = -\infty, b = \infty$

- Relationship pair: $Normal3(\mu, \tau) \rightarrow Normal1(\mu, \sigma)$
- Relationship type: Reparameterisation
- Relationship definition: $\mu = \mu, \sigma = 1/\sqrt{\tau}$

- Relationship pair: $Binomial1(n, p) \rightarrow Normal1(\mu, \sigma)$
- Relationship type: Limiting
- Relationship definition: For $X \sim Binomial1(n, p)$ as $n \rightarrow \infty, X$ is approximately normally distributed $Normal1(\mu, \sigma)$ with $\mu = np, \sigma = np(1 - p)$.

- Relationship pair: $StandardNormal1(0, 1) \rightarrow Normal1(\mu, \sigma)$
- Relationship type: Transformation
- Relationship definition: $X \sim StandardNormal1$ and $Y = \mu + \sigma X \Rightarrow Y \sim Normal1$

- Relationship pair: $NegativeBinomial1(r, p) \rightarrow Normal1(\mu, \sigma)$
- Relationship type:
- Relationship definition: $\mu = n(1 - p), n \rightarrow \infty$

- Relationship pair: $Gamma1(k, theta) \rightarrow Normal1(\mu, \sigma)$
- Relationship type: Special case & Limiting
- Relationship definition: $\mu = k\theta, \sigma^2 = k^2\theta, \theta \rightarrow \infty$

- Relationship pair: $Beta1(alpha, beta) \rightarrow Normal1(\mu, \sigma)$
- Relationship type: Special case & Limiting

- Relationship definition: $\alpha = \beta, \beta \to \infty$

- Relationship pair: $Hypergeometric1(N, K, n) \to Normal1(\mu, \sigma)$
- Relationship type: Limiting
- Relationship definition: $X \sim Hypergeometric1(N, K, n) \Rightarrow Y \sim Normal1(\mu, \sigma)$ for large n, but K/N not too small

References

[Leemis and Mcqueston, 2008], [Forbes et al., 2011]
http://en.wikipedia.org/wiki/Normal_distribution
http://www.uncertml.org/distributions/normal
http://www.math.wm.edu/~leemis/chart/UDR/PDFs/PoissonNormal.pdf
http://www.math.wm.edu/~leemis/chart/UDR/PDFs/NormalLognormal.pdf
http://www.math.wm.edu/~leemis/chart/UDR/PDFs/NormalChisquare.pdf
http://www.math.wm.edu/~leemis/chart/UDR/PDFs/LognormalNormal.pdf
http://www.math.wm.edu/~leemis/chart/UDR/PDFs/NormalStandardnormalT.pdf
http://www.math.wm.edu/~leemis/chart/UDR/PDFs/StandardnormalNormal.pdf
http://www.math.wm.edu/~leemis/chart/UDR/PDFs/NormalStandardnormalT.pdf
http://www.math.wm.edu/~leemis/chart/UDR/PDFs/GammaNormal1.pdf
http://www.math.wm.edu/~leemis/chart/UDR/PDFs/BetaNormal.pdf
http://www.math.wm.edu/~leemis/chart/UDR/PDFs/PascalNormal.pdf
http://www.math.wm.edu/~leemis/chart/UDR/PDFs/BinomialNormal.pdf
http://www.math.wm.edu/~leemis/chart/UDR/PDFs/NormalChisquare.pdf

Normal2

**name**	Normal 2 (ID: 0000265)
**type**	continuous
**variate**	x, scalar
**support**	$x \in R$

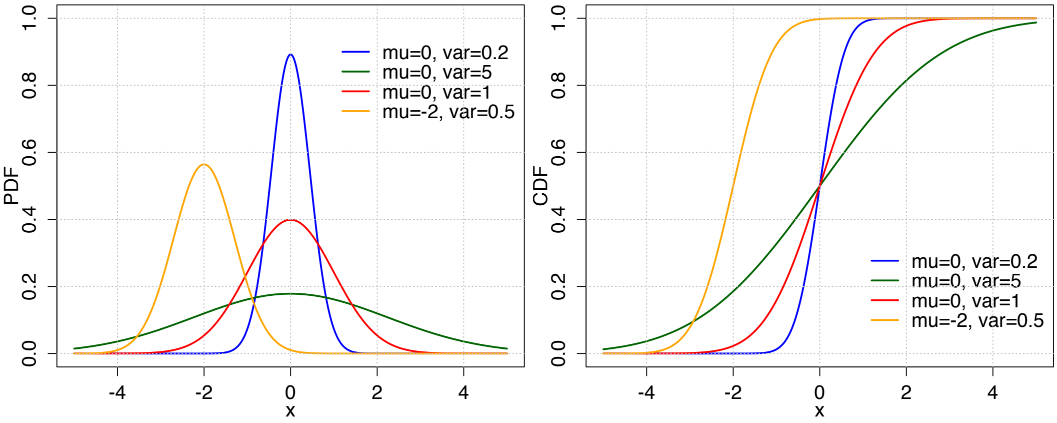

Figure 1.55: Normal2 distribution plotted using the provided R code.

Parameter: mean

**name**	mean
**type**	scalar
**symbol**	μ
**definition**	$\mu \in R$

Parameter: var

**name**	variance
**type**	scalar
**symbol**	v
**definition**	$v > 0$

Functions

PDF

$$\frac{1}{\sqrt{v}\sqrt{2\pi}}e^{-\frac{(x-\mu)^2}{2v}}$$

PDF in R

```
1/(sqrt(var)*sqrt(2*pi))*exp(-(x-mu)^2/(2*var))
```

CDF

$$\frac{1}{2}\left[1+\operatorname{erf}\left(\frac{x-\mu}{\sqrt{v}\sqrt{2}}\right)\right]$$

CDF in R

```
1/2 * (1 + erf((x-mu)/(sqrt(var)*sqrt(2))))
```

Characteristics

Mean

$$\mu$$

Median

$$\mu$$

Mode

$$\mu$$

Variance

$$v$$

Relationships

- Relationship pair: $Normal2(\mu, v) \to Normal1(\mu, \sigma)$
- Relationship type: Reparameterisation
- Relationship definition: $\mu = \mu, \sigma = \sqrt{v}$

- Relationship pair: $Normal2(\mu, v) \to Normal3(\mu, \tau)$
- Relationship type: Reparameterisation
- Relationship definition: $\mu = \mu, \tau = 1/v$

- Relationship pair: $Normal1(\mu, \sigma) \to Normal2(\mu, v)$
- Relationship type: Reparameterisation
- Relationship definition: $\mu = \mu, v = \sigma^2$

- Relationship pair: $Normal3(\mu, \tau) \to Normal2(\mu, v)$
- Relationship type: Reparameterisation
- Relationship definition: $\mu = \mu, v = 1/\tau$

References

`http://en.wikipedia.org/wiki/Normal_distribution`
`http://www.uncertml.org/distributions/normal`

Normal3

**name**	Normal 3 (ID: 0000290)
**type**	continuous
**variate**	x, scalar
**support**	$x \in R$

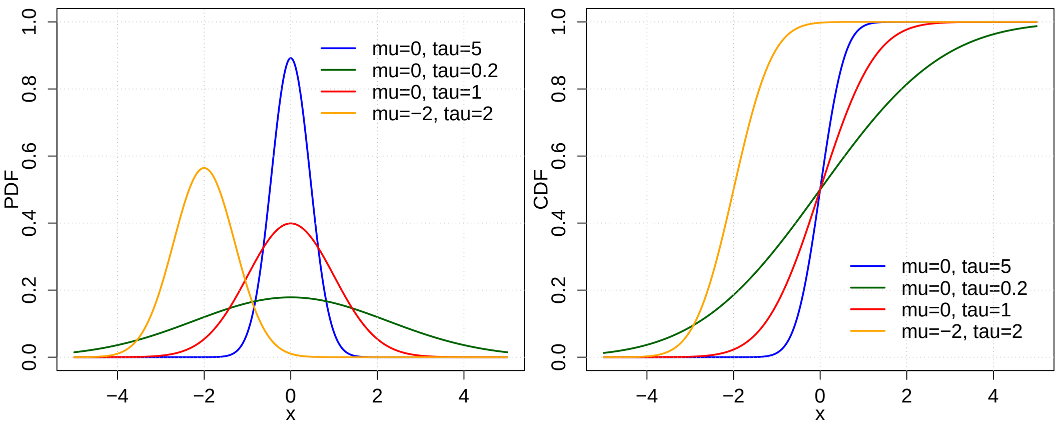

Figure 1.56: Normal3 distribution plotted using the provided R code.

Parameter: mean

**name**	mean
**type**	scalar
**symbol**	μ
**definition**	$\mu \in R$

Parameter: precision

**name**	precision
**type**	scalar
**symbol**	τ
**definition**	$\tau > 0$

Functions

PDF

$$\sqrt{\frac{\tau}{2\pi}} e^{-\frac{\tau}{2}(x-\mu)^2}$$

PDF in R

```
sqrt(tau/(2*pi))*exp(-tau/2*(x-mu)^2)
```

CDF

$$\frac{1}{2}\left[1+\operatorname{erf}\left(\frac{x-\mu}{\sqrt{1/\tau}\sqrt{2}}\right)\right]$$

CDF in R

```
1/2*(1+erf((x-mu)/(sqrt(1/tau)*sqrt(2))))
```

Characteristics

Mean

$$\mu$$

Median

$$\mu$$

Mode

$$\mu$$

Variance

$$1/\tau$$

Relationships

- Relationship pair: $Normal3(\mu, \tau) \rightarrow Normal1(\mu, \sigma)$
- Relationship type: Reparameterisation
- Relationship definition: $\mu = \mu, \sigma = 1/\sqrt{\tau}$

- Relationship pair: $Normal3(\mu, \tau) \rightarrow Normal2(\mu, v)$
- Relationship type: Reparameterisation
- Relationship definition: $\mu = \mu, v = 1/\tau$

- Relationship pair: $Normal1(\mu, \sigma) \rightarrow Normal3(\mu, \tau)$
- Relationship type: Reparameterisation
- Relationship definition: $\mu = \mu, \tau = 1/\sigma^2$

- Relationship pair: $Normal2(\mu, v) \rightarrow Normal3(\mu, \tau)$
- Relationship type: Reparameterisation
- Relationship definition: $\mu = \mu, \tau = 1/v$

References

[Spiegelhalter et al., 2003]

NormalInverseGamma1

**name**	Normal-inverse-gamma 1 (ID: 0000316)
**type**	continuous
**variate**	x, scalar
**support**	$x \in (-\infty, +\infty), \sigma^2 \in (0, +\infty)$

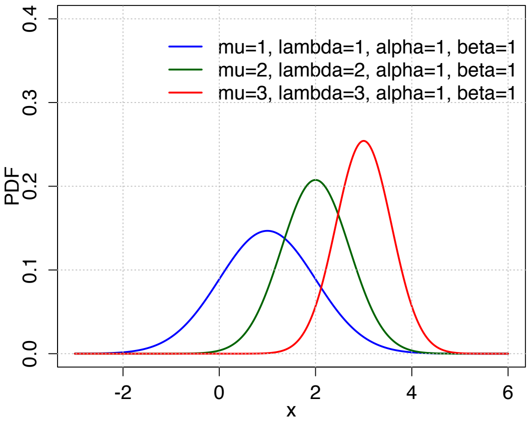

Figure 1.57: NormalInverseGamma1 distribution plotted using the provided R code.

Parameter: mean

**name**	location
**type**	scalar
**symbol**	μ
**definition**	$\mu \in R$

Parameter: lambda

**name**	lambda
**type**	scalar
**symbol**	λ
**definition**	$\lambda > 0, \lambda \in R$

Parameter: alpha

**name**	shape
**type**	scalar
**symbol**	α
**definition**	$\alpha > 0, \alpha \in R$

Parameter: beta

**name**	scale
**type**	scalar
**symbol**	β
**definition**	$\beta > 0, \beta \in R$

Functions

PDF

$$\frac{\sqrt{\lambda}}{\sigma\sqrt{2\pi}}\frac{\beta^{\alpha}}{\Gamma(\alpha)}\left(\frac{1}{\sigma^2}\right)^{\alpha+1}e^{-\frac{2\beta+\lambda(x-\mu)^2}{2\sigma^2}}$$

PDF in R

```
sqrt(lambda)/(sigma*sqrt(2*pi)) * beta^alpha/gamma(alpha) * (1/sigma^2)^(alpha + 1) *
 exp(- (2*beta+lambda*(x-mu)^2)/(2*sigma^2))
```

References

http://en.wikipedia.org/wiki/Normal-inverse-gamma_distribution
http://www.uncertml.org/distributions/normal-inverse-gamma

Pareto1

**name**	Pareto 1 (ID: 0000361)
**type**	continuous
**variate**	x, scalar
**support**	$x \in [x_m, +\infty)$

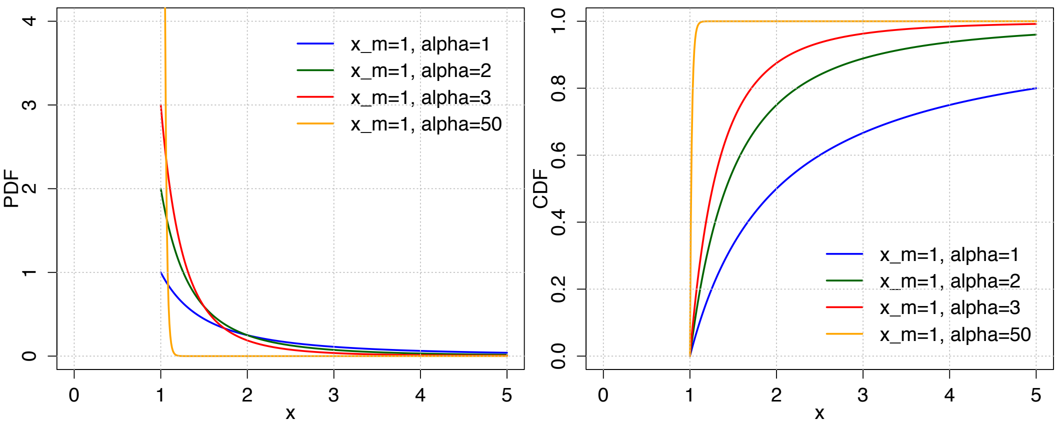

Figure 1.58: Pareto1 distribution plotted using the provided R code.

Parameter: scale

**name**	scale
**type**	scalar
**symbol**	x_m
**definition**	$x_m > 0, x_m \in R$

Parameter: shape

**name**	shape
**type**	scalar
**symbol**	α
**definition**	$\alpha > 0, \alpha \in R$

Functions

PDF

$$\frac{\alpha\, x_m^\alpha}{x^{\alpha+1}} \text{ for } x \geq x_m$$

PDF in R

```
(alpha * x_m^alpha) / x^(alpha+1)
```

CDF

$$1 - \left(\frac{x_m}{x}\right)^\alpha \text{ for } x \geq x_m$$

CDF in R

```
1-(x_m/x)^alpha
```

Characteristics

Mean

$$\begin{cases} \infty & \text{for } \alpha \leq 1 \\ \frac{\alpha\, x_m}{\alpha - 1} & \text{for } \alpha > 1 \end{cases}$$

Median

$$x_m \sqrt[\alpha]{2}$$

Mode

$$x_m$$

Variance

$$\begin{cases} \infty & \text{for } \alpha \in (1, 2] \\ \frac{x_m^2\, \alpha}{(\alpha-1)^2(\alpha-2)} & \text{for } \alpha > 2 \end{cases}$$

Relationships

- Relationship pair: $Pareto1(x_m, \alpha) \rightarrow Exponential1(\lambda)$
- Relationship type: Transformation
- Relationship definition: $X \sim Pareto1, Y = \log(X/\lambda) \Rightarrow Y \sim Exponential1$

- Relationship pair: $StandardUniform1(0, 1) \rightarrow Pareto1(x_m, \alpha)$
- Relationship type: Transformation
- Relationship definition: $x_m X^{-1/\alpha}$

References

http://en.wikipedia.org/wiki/Pareto_distribution
http://www.uncertml.org/distributions/pareto

Poisson1

**name**	Poisson 1 (ID: 0000410)
**type**	discrete
**variate**	k, scalar
**support**	$k \in \{0, 1, 2, 3, \dots\}$

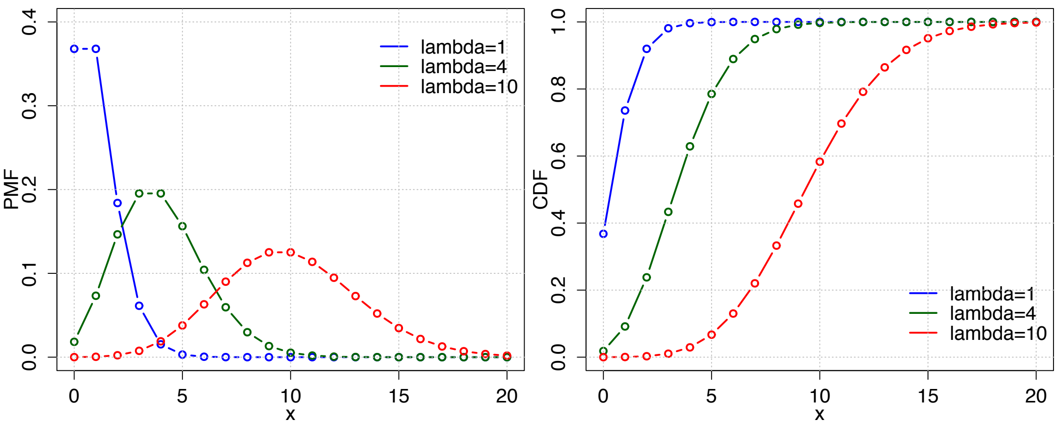

Figure 1.59: Poisson1 distribution plotted using the provided R code.

Parameter: rate

**name**	Poisson intensity
**type**	scalar
**symbol**	λ
**definition**	$\lambda \in Z^{+}$

Functions

PMF

$$\frac{\lambda^k}{k!} e^{-\lambda}$$

PMF in R

```
lambda^k/factorial(k) * exp(-lambda)
```

CDF

$$\frac{\gamma(\lfloor k+1 \rfloor, \lambda)}{\lfloor k \rfloor !}$$

CDF in R

```
Igamma(floor(k+1), lambda, lower=F) / factorial(floor(k))
```

Characteristics

Mean

$$\lambda$$

Median

$$\approx \lfloor \lambda + 1/3 - 0.02/\lambda \rfloor$$

Mode

$$\lceil \lambda \rceil - 1, \lfloor \lambda \rfloor$$

Variance

$$\lambda$$

Relationships

- Relationship pair: $Poisson1(\lambda) \rightarrow Normal1(\mu, \sigma)$
- Relationship type: Transformation & Limiting
- Relationship definition: $\sigma^2 = \lambda, \mu = \lambda, \lambda \rightarrow \infty$

- Relationship pair: $GeneralizedPoisson1(\theta, \delta) \rightarrow Poisson1(\lambda)$
- Relationship type: Special case
- Relationship definition: $\delta = 0, \theta = \mu$

- Relationship pair: $ConwayMaxwellPoisson1(\lambda, \nu) \rightarrow Poisson1(\lambda)$
- Relationship type: Transformation
- Relationship definition: For $\nu = 1$ the sum has a Poisson distribution with parameter $n\lambda$

- Relationship pair: $Binomial1(n, p) \rightarrow Poisson1(\lambda)$
- Relationship type: Transformation & Limiting
- Relationship definition: $\lambda = np, n \rightarrow \infty$

- Relationship pair: $GeneralizedPoisson2(\mu, \delta) \rightarrow Poisson1(\lambda)$
- Relationship type: Special case
- Relationship definition: $\delta = 0, \lambda = \mu$

- Relationship pair: $GeneralizedPoisson3(\mu, \alpha) \rightarrow Poisson1(\lambda)$
- Relationship type: Special case
- Relationship definition: $\alpha = 0, \lambda = \mu$

- Relationship pair: $DoublePoisson1(\mu, \phi) \rightarrow Poisson1(\lambda)$
- Relationship type: Special case
- Relationship definition: $\phi = 1$

- Relationship pair: $ZeroInflatedPoisson1(\lambda, \pi) \rightarrow Poisson1(\lambda)$
- Relationship type: Special case
- Relationship definition: $\pi = 0$

- Relationship pair: $ZeroInflatedNegativeBinomial1(\lambda, \tau, p0) \rightarrow Poisson1(\lambda)$
- Relationship type: Limiting
- Relationship definition: $p0 = 0, \tau \rightarrow 0$

- Relationship pair: $NegativeBinomial1(r, p) \rightarrow Poisson1(\lambda)$
- Relationship type: Reparameterisation & Limiting
- Relationship definition: $\mu = np, n \rightarrow \infty$

- Relationship pair: $ZeroInflatedGeneralizedPoisson1(\mu, \alpha, p0) \rightarrow Poisson1(\lambda)$
- Relationship type: Special case & Reparameterisation
- Relationship definition: $p0 = 0, \alpha = 0, \lambda = \mu$

- Relationship pair: $Hypergeometric1(N, K, n) \rightarrow Poisson1(\lambda)$
- Relationship type: Limiting
- Relationship definition: $X \sim Hypergeometric1(N, K, n) \Rightarrow Y \sim Poisson1(\lambda)$ as K, N and n tend to infinity for K/N small and $nK/N \rightarrow \lambda$

References

[Leemis and Mcqueston, 2008], [Yang et al., 2007], [Plan, 2014], [Hilbe, 2011], [Famoye and Singh, 2006]
[Cameron and Trivedi, 2013], [Trocóniz et al., 2009], [Forbes et al., 2011], [Shmueli et al., 2005]
http://en.wikipedia.org/wiki/Poisson_distribution
http://www.uncertml.org/distributions/poisson
http://www.math.wm.edu/~leemis/chart/UDR/PDFs/BinomialPoisson.pdf
http://www.math.wm.edu/~leemis/chart/UDR/PDFs/PoissonNormal.pdf
http://www.math.wm.edu/~leemis/chart/UDR/PDFs/PascalPoisson.pdf

Rayleigh1

**name**	Rayleigh 1 (ID: 0000462)
**type**	continuous
**variate**	x, scalar
**support**	$x \in [0, +\infty)$

Parameter: scale

**name**	scale
**type**	scalar
**symbol**	σ
**definition**	$\sigma > 0$

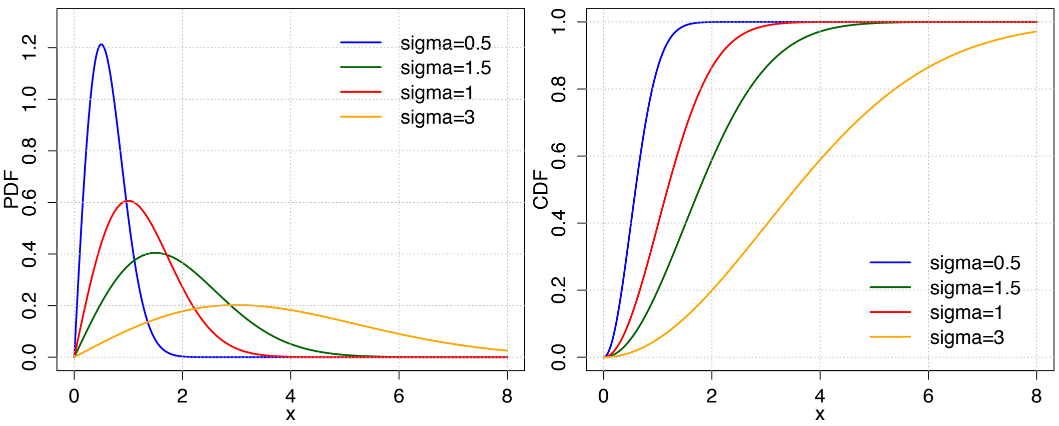

Figure 1.60: Rayleigh1 distribution plotted using the provided R code.

Functions

PDF

$$\frac{x}{\sigma^2}e^{-x^2/(2\sigma^2)}$$

PDF in R

```
x/sigma^2 * exp(-x^2/(2*sigma^2))
```

CDF

$$1-e^{-x^2/(2\sigma^2)}$$

CDF in R

```
1 - exp(-x^2/(2*sigma^2))
```

Characteristics

Mean

$$\sigma\sqrt{\frac{\pi}{2}}$$

Median

$$\sigma\sqrt{\log(4)}$$

Mode

$$\sigma$$

Variance

$$\frac{4-\pi}{2}\sigma^2$$

Relationships

- Relationship pair: $Weibull1(\lambda, k) \to Rayleigh1(\sigma)$
- Relationship type:
- Relationship definition: $k = 2, \lambda = \sqrt{2}\sigma$

References

https://en.wikipedia.org/wiki/Rayleigh_distribution

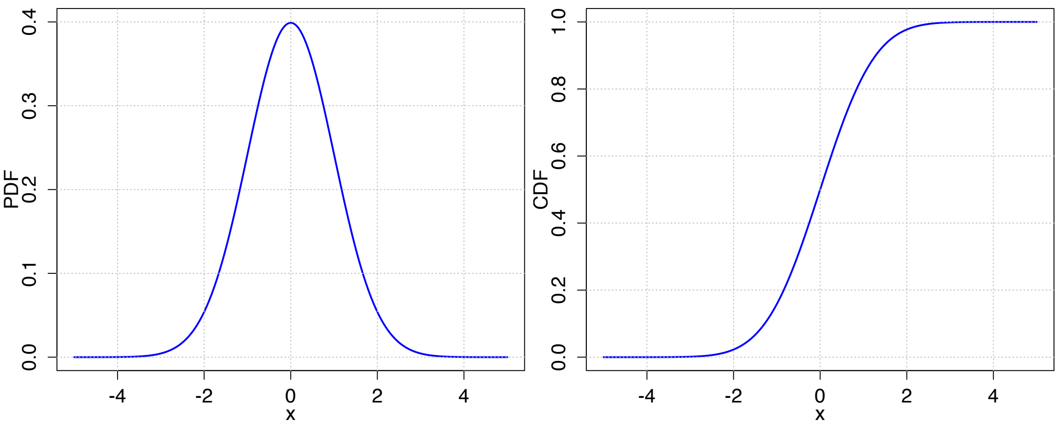

Figure 1.61: StandardNormal1 distribution plotted using the provided R code.

StandardNormal1

**name**	Standard Normal 1 (ID: 0000562)
**type**	continuous
**variate**	x, scalar
**support**	$x \in R$

Parameter: mean

**name**	mean
**type**	scalar
**symbol**	μ
**definition**	$\mu = 0$

Parameter: stdev

**name**	standard deviation
**type**	scalar
**symbol**	σ
**definition**	$\sigma = 1$

Functions

PDF

$$\frac{e^{-\frac{1}{2}x^2}}{\sqrt{2\pi}}$$

PDF in R

```
1/(sqrt(2*pi))*exp(-x^2/2)
```

CDF

$$\frac{1}{2}\left[1 + \operatorname{erf}\left(\frac{x}{\sqrt{2}}\right)\right]$$

CDF in R

```
1/2 * (1 + erf(x/(sqrt(2))))
```

Characteristics

Mean

$$0$$

Median

$$0$$

Mode

$$0$$

Variance

$$1$$

Relationships

- Relationship pair: $StandardNormal1(0,1) \rightarrow ChiSquared1(k)$
- Relationship type: Transformation
- Relationship definition: ΣX^2

- Relationship pair: $StandardNormal1(0,1) \rightarrow Normal1(\mu,\sigma)$
- Relationship type: Transformation
- Relationship definition: $X \sim StandardNormal1$ and $Y = \mu + \sigma X \Rightarrow Y \sim Normal1$

- Relationship pair: $StudentT1(\nu) \rightarrow StandardNormal1(0,1)$
- Relationship type: Limiting
- Relationship definition: $X \sim StudentT1(\nu) \Rightarrow StandardNormal1(0,1)$ as ν tends to infinity. The approximation is reasonable for $\nu \geq 30$

- Relationship pair: $Normal1(\mu,\sigma) \rightarrow StandardNormal1(0,1)$
- Relationship type: Special case
- Relationship definition: $\mu = 0, \sigma = 1$

- Relationship pair: $InverseGaussian1(\lambda,\mu) \rightarrow StandardNormal1(0,1)$
- Relationship type: Limiting
- Relationship definition: $\lambda \rightarrow \infty$

- Relationship pair: $Normal1(\mu,\sigma) \rightarrow StandardNormal1(0,1)$
- Relationship type: Transformation
- Relationship definition: $X \sim Normal1(\mu,\sigma); Y = (X-\mu)/\sigma; Y \sim StandardNormal1$

References

[Forbes et al., 2011], [Leemis and Mcqueston, 2008]
https://en.wikipedia.org/wiki/Normal_distribution#Standard_normal_distribution
http://www.math.wm.edu/~leemis/chart/UDR/PDFs/NormalStandardnormalT.pdf
http://www.math.wm.edu/~leemis/chart/UDR/PDFs/StandardnormalChisquare.pdf
http://www.math.wm.edu/~leemis/chart/UDR/PDFs/StandardnormalNormal.pdf
http://www.math.wm.edu/~leemis/chart/UDR/PDFs/NormalStandardnormalT.pdf
http://www.math.wm.edu/~leemis/chart/UDR/PDFs/InversegaussianStandardnormal.pdf

StandardUniform1

**name**	Standard Uniform 1 (ID: 0000587)
**type**	continuous
**variate**	x, scalar
**support**	$x \in [0,1]$

Parameter: minimum

**name**	minimum
**type**	scalar
**symbol**	a
**definition**	$a = 0$

Parameter: maximum

**name**	maximum
**type**	scalar
**symbol**	b
**definition**	$b = 1$

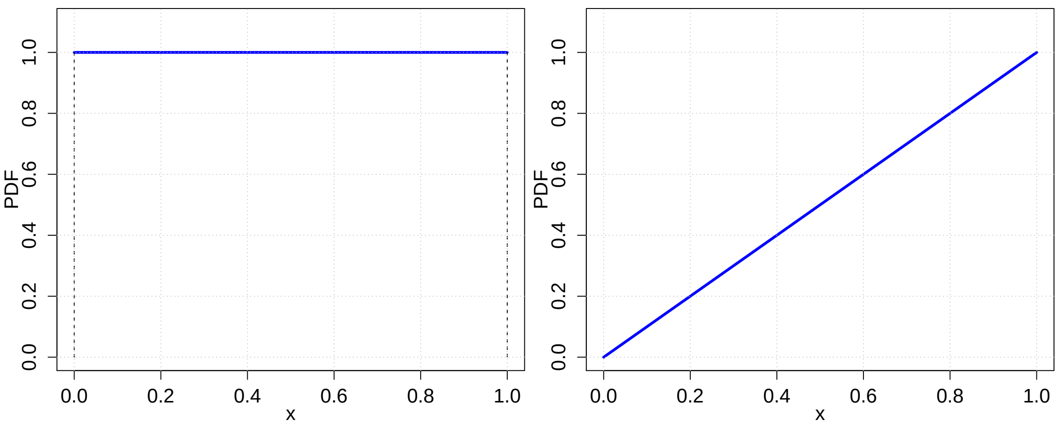

Figure 1.62: StandardUniform1 distribution plotted using the provided R code.

Functions

PDF

$$1$$

PDF in R

```
1
```

CDF

$$x$$

CDF in R

```
x
```

Characteristics

Mean

$$0.5$$

Median

$$0.5$$

Mode

$$\text{any value in } [0, 1]$$

Relationships

- Relationship pair: $StandardUniform1(0, 1) \to Exponential1(\lambda)$
- Relationship type: Transformation
- Relationship definition: $-\frac{1}{\lambda}\log(X)$

- Relationship pair: $StandardUniform1(0, 1) \to Beta1(alpha, beta)$
- Relationship type: Transformation
- Relationship definition: $aX_1, X_2, ..., X_n(iid) \sim StandardNormal1 \Rightarrow$ with $\alpha = r$ and $\beta = n-r+1, X_{(r)} \sim Beta(\alpha, \beta)$

- Relationship pair: $StandardUniform1(0, 1) \to Uniform1(a, b)$
- Relationship type: Transformation
- Relationship definition: $X \sim StandardNormal1$ and $Y = a + (b-a)X \Rightarrow Y \sim Uniform1$

- Relationship pair: $StandardUniform1 \to LogLogistic2(\lambda, \kappa)$
- Relationship type: Transformation
- Relationship definition: If $X \sim StandardUniform1$ and $Y = \frac{1}{\lambda}\left(\frac{1-X}{X}\right)^{1/\kappa} \Rightarrow Y \sim LogLogistic2(\lambda, \kappa)$

- Relationship pair: $Uniform1(a, b) \to StandardUniform1(0, 1)$
- Relationship type: Special case

- Relationship definition: $a = 0, b = 1$
- Relationship pair: $StandardUniform1(0, 1) \rightarrow Pareto1(x_m, \alpha)$
- Relationship type: Transformation
- Relationship definition: $x_m X^{-1/\alpha}$

References

[Leemis and Mcqueston, 2008]
http://www.math.wm.edu/~leemis/chart/UDR/PDFs/Standarduniform.pdf
http://www.math.wm.edu/~leemis/chart/UDR/PDFs/StandarduniformExponentialB.pdf
http://www.math.wm.edu/~leemis/chart/UDR/PDFs/StandarduniformPareto.pdf
http://www.math.wm.edu/~leemis/chart/UDR/PDFs/StandarduniformBeta.pdf
http://www.math.wm.edu/~leemis/chart/UDR/PDFs/StandarduniformUniform.pdf
http://www.math.wm.edu/~leemis/chart/UDR/PDFs/UniformStandarduniform.pdf

StudentT1

**name**	Student's t-distribution 1 (ID: 0000613)
**type**	continuous
**variate**	x, scalar
**support**	$x \in (-\infty, +\infty)$

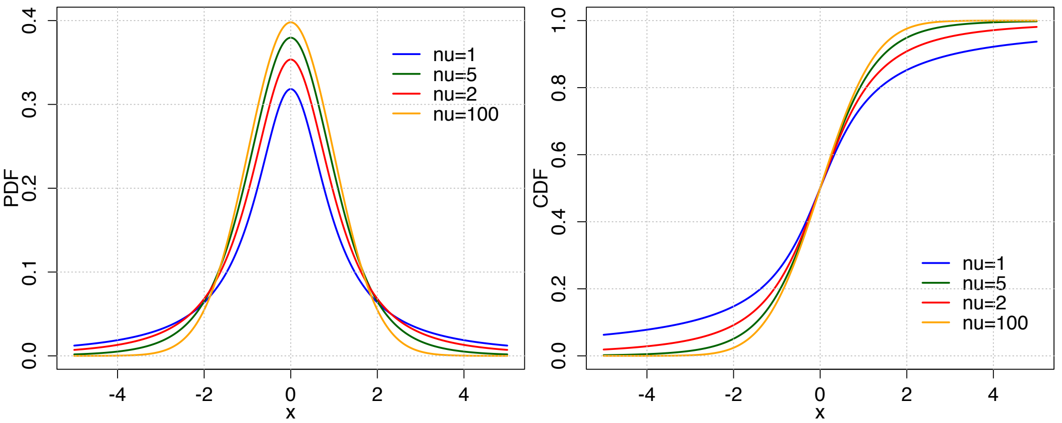

Figure 1.63: StudentT1 distribution plotted using the provided R code.

Parameter: degreesOfFreedom

**name**	degrees of freedom
**type**	scalar
**symbol**	ν
**definition**	$\nu > 0, \nu \in R$

Functions

PDF

$$\frac{\Gamma\left(\frac{\nu+1}{2}\right)}{\sqrt{\nu\pi}\,\Gamma\left(\frac{\nu}{2}\right)}\left(1+\frac{x^2}{\nu}\right)^{-\frac{\nu+1}{2}}$$

PDF in R

```
gamma((nu+1)/2)/(sqrt(nu*pi)*gamma(nu/2))*(1+x^2/nu)^(-(nu+1)/2)
```

CDF

$$\frac{1}{2} + x\Gamma\left(\frac{\nu+1}{2}\right) \times \frac{{}_2F_1\left(\frac{1}{2}, \frac{\nu+1}{2}; \frac{3}{2}; -\frac{x^2}{\nu}\right)}{\sqrt{\pi\nu}\,\Gamma\left(\frac{\nu}{2}\right)}$$

CDF in R

```
1/2+x*gamma((nu+1)/2)*hypergeo(1/2,(nu+1)/2,3/2,-x^2/nu)/( sqrt(pi*nu) *gamma(nu/2))
```

Characteristics

Mean

$$\begin{cases} 0 & \text{for } \nu > 1 \\ undefined & \text{else} \end{cases}$$

Median

$$0$$

Mode

$$0$$

Variance

$$\begin{cases} \frac{\nu}{\nu-2} & \text{for } \nu > 2 \\ \infty & \text{for } 1 < \nu \leq 2 \\ undefined & \text{else} \end{cases}$$

Relationships

- Relationship pair: $StudentT1(\nu) \rightarrow StandardNormal1(0,1)$
- Relationship type: Limiting
- Relationship definition: $X \sim StudentT1(\nu) \Rightarrow StandardNormal1(0,1)$ as ν tends to infinity. The approximation is reasonable for $\nu \geq 30$

- Relationship pair: $StudentT1(\nu) \rightarrow F1(n_1, n_2)$
- Relationship type: Transformation
- Relationship definition: If $X \sim StudentT1(\nu) \Rightarrow Y = X^2 \sim F(1,\nu)$

- Relationship pair: $StudentT2(\mu, \tau, k) \rightarrow StudentT1(\nu)$
- Relationship type: Reparameterisation
- Relationship definition: $\mu = 0, \tau = 1$

References

[Forbes et al., 2011], [Leemis and Mcqueston, 2008]
http://en.wikipedia.org/wiki/Student's_t-distribution
http://www.uncertml.org/distributions/student-t
http://www.math.wm.edu/~leemis/chart/UDR/PDFs/TF.pdf

StudentT2

**name**	Student's t-distribution 2 (ID: 0000635)
**type**	continuous
**variate**	x, scalar
**support**	$x \in (-\infty, +\infty)$

Parameter: mean

**name**	mean
**type**	scalar
**symbol**	μ
**definition**	μinR

Parameter: scale

**name**	scale
**type**	scalar
**symbol**	τ
**definition**	$\tau > 0$

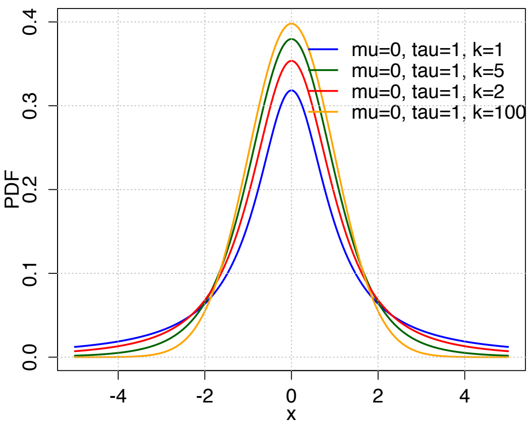

Figure 1.64: StudentT2 distribution plotted using the provided R code.

Parameter: degreesOfFreedom

**name**	degrees of freedom
**type**	scalar
**symbol**	k
**definition**	$k \geq 2$

Functions

PDF

$$\frac{\Gamma\left(\frac{k+1}{2}\right)}{\Gamma\left(\frac{k}{2}\right)}\sqrt{\frac{\tau}{k\pi}}\left[1+\frac{\tau}{k}(x-\mu)^2\right]^{-\frac{k+1}{2}}$$

PDF in R

```
gamma((k+1)/2)/gamma(k/2)*sqrt(tau/(k*pi))*(1+tau/k*(x-mu)^2)^(-(k+1)/2)
```

Characteristics

Mean

$$\mu$$

Mode

$$\mu \text{ for } k > 1$$

Variance

$$\frac{1}{\tau}\frac{k}{k-2} \text{ for } k > 2$$

Relationships

- Relationship pair: $StudentT2(\mu, \tau, k) \rightarrow StudentT1(\nu)$
- Relationship type: Reparameterisation
- Relationship definition: $\mu = 0, \tau = 1$

References

https://en.wikipedia.org/wiki/Student%27s_t-distribution#Non-standardized_Student.27s_t-distribution

Triangular1

**name**	Triangular 1 (ID: 0000661)
**type**	continuous
**variate**	x, scalar
**support**	$a \leq x \leq b$

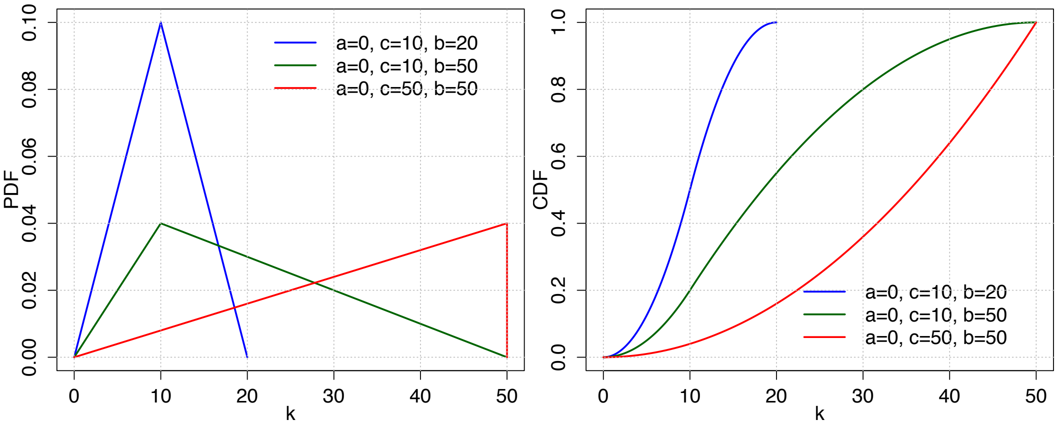

Figure 1.65: Triangular1 distribution plotted using the provided R code.

Parameter: lowerLimit

**name**	lower limit
**type**	scalar
**symbol**	a
**definition**	$a \in R$

Parameter: upperLimit

**name**	upper limit
**type**	scalar
**symbol**	b
**definition**	$b \in R, a < b$

Parameter: shape

**name**	shape (mode)
**type**	scalar
**symbol**	c
**definition**	$c \in R$

Functions

PDF

$$\begin{cases} 2(x-a)/[(b-a)(c-a)] & \text{for } a \leq x \leq c \\ 2(b-x)/[(b-a)(b-c)] & \text{for } c \leq x \leq b \end{cases}$$

PDF in R

```
2*(x-a) / ((b-a)*(c-a)) for a <= x <= c \\
2*(b-x) / ((b-a)*(b-c)) for c <= x <= b
```

CDF

$$\begin{cases} (x-a)^2/[(b-a)(c-a)] & \text{for } a \leq x \leq c \\ 1-(b-x)^2/[(b-a)(b-c)] & \text{for } c \leq x \leq b \end{cases}$$

CDF in R

```
(x-a)^2 / ((b-a)*(c-a)) for a <= x <= c \\
1 - (b-x)^2 / ((b-a)*(b-c)) for c <= x <= b
```

Characteristics

Mean

$$(a+b+c)/3$$

Mode

$$c$$

Variance

$$(a^2+b^2+c^2-ab-ac-bc)/18$$

References

[Forbes et al., 2011]

TruncatedNormal1

**name**	Truncated Normal 1 (ID: 0000681)
**type**	continuous
**variate**	x, scalar
**support**	$x \in [a, b]$

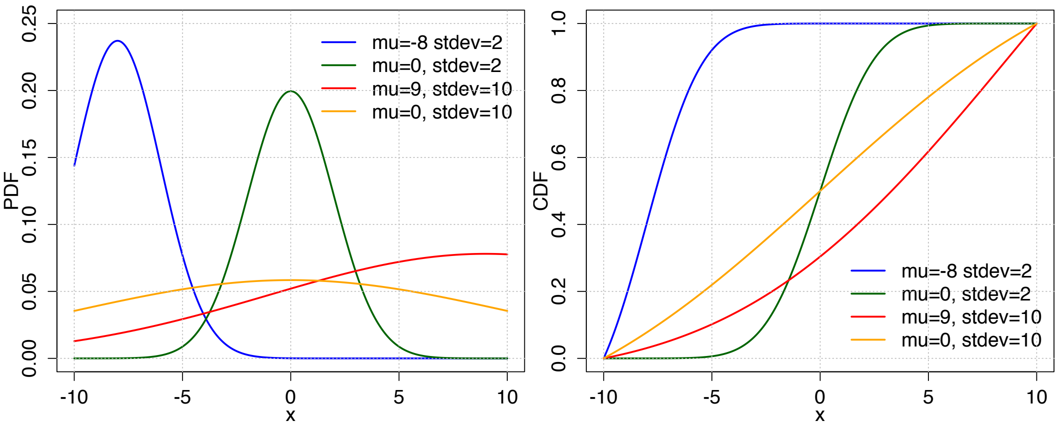

Figure 1.66: TruncatedNormal1 distribution plotted using the provided R code.

Parameter: mean

**name**	mean
**type**	scalar
**symbol**	μ
**definition**	$\mu \in R$

Parameter: stdev

**name**	standard deviation
**type**	scalar
**symbol**	σ
**definition**	$\sigma > 0$

Parameter: lowerBound

**name**	lower bound
**type**	scalar
**symbol**	a
**definition**	$a \in R$

Parameter: upperBound

**name**	upper bound
**type**	scalar
**symbol**	b
**definition**	$b \in R, b > a$

Functions

PDF

$$\frac{\frac{1}{\sigma}\phi(\frac{x-\mu}{\sigma})}{\Phi(\frac{b-\mu}{\sigma}) - \Phi(\frac{a-\mu}{\sigma})}$$

PDF in R

```
( 1/sigma * phi((x-mu)/sigma) ) / ( Phi((b-mu)/sigma)-Phi((a-mu)/sigma) )

phi = function(x) {  1/(sqrt(2*pi))*exp(-x^2/2) }
Phi = function(x) {  1/2 * (1 + erf(x/(sqrt(2)))) }
erf = function(x) {  2 * pnorm(x * sqrt(2)) - 1 }
```

CDF

$$\frac{\Phi(\frac{x-\mu}{\sigma}) - \Phi(\frac{a-\mu}{\sigma})}{\Phi(\frac{b-\mu}{\sigma}) - \Phi(\frac{a-\mu}{\sigma})}$$

CDF in R

```
( Phi((x-mu)/sigma)-Phi((a-mu)/sigma) ) / ( Phi((b-mu)/sigma)-Phi((a-mu)/sigma) )

Phi = function(x) {  1/2 * (1 + erf(x/(sqrt(2)))) }
erf = function(x) {  2 * pnorm(x * sqrt(2)) - 1 }
```

Characteristics

Mean

$$\mu + \frac{\phi(\frac{a-\mu}{\sigma}) - \phi(\frac{b-\mu}{\sigma})}{\Phi(\frac{b-\mu}{\sigma}) - \Phi(\frac{a-\mu}{\sigma})}\sigma$$

Variance

$$\sigma^2\Big[1 + \frac{\frac{a-\mu}{\sigma}\phi(\frac{a-\mu}{\sigma}) - \frac{b-\mu}{\sigma}\phi(\frac{b-\mu}{\sigma})}{\Phi(\frac{b-\mu}{\sigma}) - \Phi(\frac{a-\mu}{\sigma})} - \Big(\frac{\phi(\frac{a-\mu}{\sigma}) - \phi(\frac{b-\mu}{\sigma})}{\Phi(\frac{b-\mu}{\sigma}) - \Phi(\frac{a-\mu}{\sigma})}\Big)^2\Big]$$

Relationships

- Relationship pair: $TruncatedNormal1(\mu, \sigma, a, b) \rightarrow Normal1(\mu, \sigma)$
- Relationship type: Special case
- Relationship definition: $a = -\infty, b = \infty$

- Relationship pair: $TruncatedNormal1(\mu, \sigma, a, b) \rightarrow HalfNormal1(\theta)$
- Relationship type: Special case
- Relationship definition: $\mu = 0, a = 0, b = \infty$

References

[Forbes et al., 2011], [Forbes et al., 2011]
https://en.wikipedia.org/wiki/Truncated_normal_distribution
http://reference.wolfram.com/language/ref/HalfNormalDistribution.html

Uniform1

**name**	Uniform 1 (ID: 0000703)
**type**	continuous
**variate**	x, scalar
**support**	$x \in [a, b]$

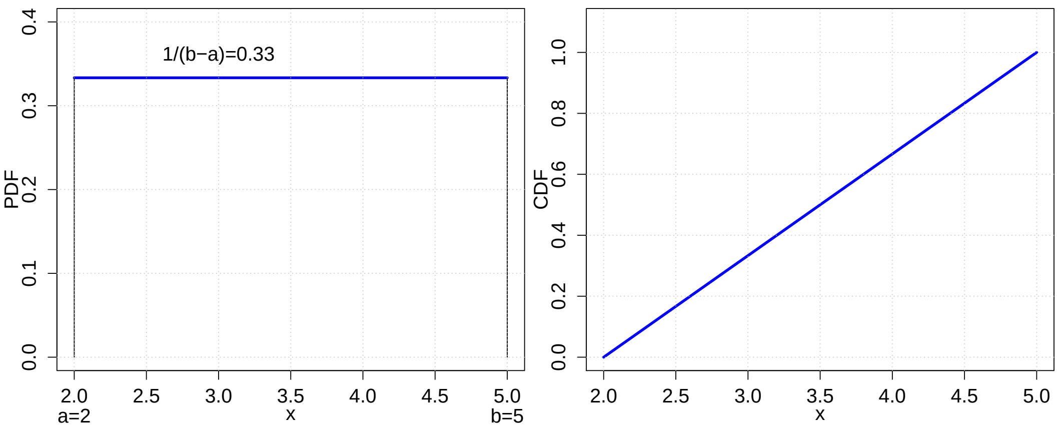

Figure 1.67: Uniform1 distribution plotted using the provided R code.

Parameter: minimum

**name**	minimum
**type**	scalar
**symbol**	a
**definition**	$a \in R$

Parameter: maximum

**name**	maximum
**type**	scalar
**symbol**	b
**definition**	$b \in R, a < b$

Functions

PDF

$$\begin{cases} \frac{1}{b-a} & \text{for } x \in [a,b] \\ 0 & \text{otherwise} \end{cases}$$

PDF in R

```
1/(b-a)
```

CDF

$$\begin{cases} 0 & \text{for } x < a \\ \frac{x-a}{b-a} & \text{for } x \in [a,b) \\ 1 & \text{for } x \geq b \end{cases}$$

CDF in R

```
(x-a)/(b-a)
```

Characteristics

Mean

$$\frac{1}{2}(a+b)$$

Median

$$\frac{1}{2}(a+b)$$

Mode

any value in $[a,b]$

Variance

$$\frac{1}{12}(b-a)^2$$

Relationships

- Relationship pair: $Uniform1(a, b) \rightarrow StandardUniform1(0, 1)$
- Relationship type: Special case
- Relationship definition: $a = 0, b = 1$

- Relationship pair: $StandardUniform1(0, 1) \rightarrow Uniform1(a, b)$
- Relationship type: Transformation
- Relationship definition: $X \sim StandardNormal1$ and $Y = a + (b - a)X \Rightarrow Y \sim Uniform1$

References

[Leemis and Mcqueston, 2008]
http://en.wikipedia.org/wiki/Uniform_distribution_(continuous)
http://www.uncertml.org/distributions/uniform
http://www.math.wm.edu/~leemis/chart/UDR/PDFs/StandarduniformUniform.pdf
http://www.math.wm.edu/~leemis/chart/UDR/PDFs/UniformStandarduniform.pdf

UniformDiscrete1

**name**	Uniform Discrete 1 (ID: 0000727)
**type**	discrete
**variate**	k, scalar
**support**	$k \in \{a, a + 1, ..., b - 1, b\}$

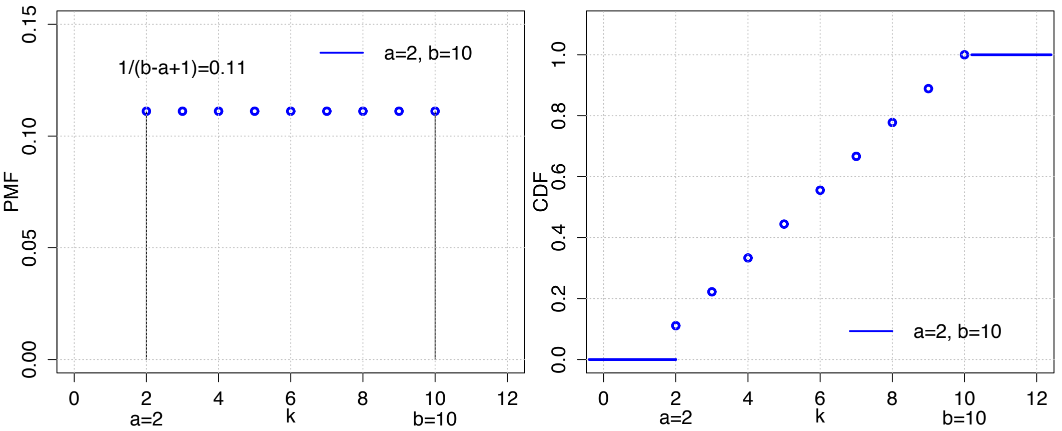

Figure 1.68: UniformDiscrete1 distribution plotted using the provided R code.

Parameter: minimum

**name**	minimum
**type**	scalar
**symbol**	a
**definition**	$a \in \{\ldots, -2, -1, 0, 1, 2, 3, \ldots\}$

Parameter: maximum

**name**	maximum
**type**	scalar
**symbol**	b
**definition**	$b \in \{\ldots, -2, -1, 0, 1, 2, 3, \ldots\}, b \geq a$

Functions

PMF

$$1/(b - a + 1)$$

PMF in R

```
1/(b-a+1)
```

CDF

$$(k-a+1)/(b-a+1)$$

CDF in R

```
(k-a+1)/(b-a+1)
```

Characteristics

Mean

$$(a+b)/2$$

Median

$$(a+b)/2$$

Variance

$$\frac{(b-a+1)^2-1}{12}$$

Relationships

- Relationship pair: $UniformDiscrete1(a,b) \rightarrow UniformDiscrete2(n)$
- Relationship type: Special case
- Relationship definition: $a=0, b=n$

References

[Leemis and Mcqueston, 2008], [Marichev and Trott, 2013]
`http://www.math.wm.edu/~leemis/chart/UDR/PDFs/DiscreteuniformRectangular.pdf`

UniformDiscrete2

**name**	Uniform Discrete 2 (ID: 0000750)
**type**	discrete
**variate**	k, scalar
**support**	$k \in \{0,1,2,\ldots,n\}$

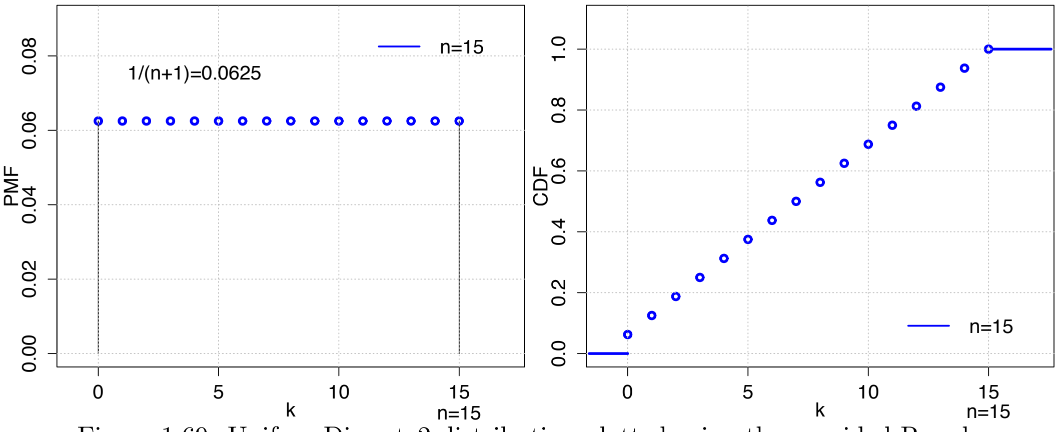

Figure 1.69: UniformDiscrete2 distribution plotted using the provided R code.

Parameter: numberOfValues

**name**	number of values
**type**	scalar
**symbol**	n
**definition**	$n \in N$

Functions

PMF

$$1/(n+1)$$

PMF in R

```
1/(n+1)
```

CDF

$$\frac{k+1}{n+1}$$

CDF in R

```
(k+1)/(n+1)
```

Characteristics

Mean

$$\frac{n}{2}$$

Variance

$$\frac{n(n+2)}{12}$$

Relationships

- Relationship pair: $UniformDiscrete1(a, b) \to UniformDiscrete2(n)$
- Relationship type: Special case
- Relationship definition: $a = 0, b = n$

- Relationship pair: $BetaBinomial1(n, \alpha, \beta) \to UniformDiscrete2(n)$
- Relationship type: Special case
- Relationship definition: $\alpha = 1, \beta = 1$

References

[Leemis and Mcqueston, 2008], [Forbes et al., 2011]
http://www.math.wm.edu/~leemis/chart/UDR/PDFs/Rectangular.pdf
http://www.math.wm.edu/~leemis/chart/UDR/PDFs/DiscreteuniformRectangular.pdf
http://www.math.wm.edu/~leemis/chart/UDR/PDFs/BetabinomialRectangular.pdf

Weibull1

**name**	Weibull 1 (ID: 0000800)
**type**	continuous
**variate**	x, scalar
**support**	$x \in [0, +\infty)$

Parameter: scale

**name**	scale
**type**	scalar
**symbol**	λ
**definition**	$\lambda \in (0, +\infty)$

Parameter: shape

**name**	shape
**type**	scalar
**symbol**	k
**definition**	$k \in (0, +\infty)$

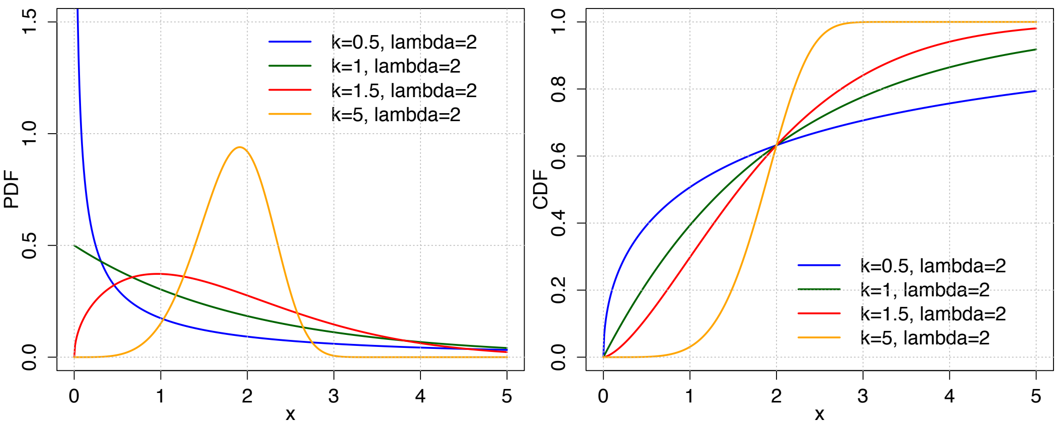

Figure 1.70: Weibull1 distribution plotted using the provided R code.

Functions

PDF

$$\frac{k}{\lambda}\left(\frac{x}{\lambda}\right)^{k-1} e^{-(x/\lambda)^k}$$

PDF in R

```
k/lambda * (x/lambda)^(k-1) * exp(-(x/lambda)^k)
```

CDF

$$1 - \exp(-(x/\lambda)^k)$$

CDF in R

```
1- exp(-(x/lambda)^k)
```

Characteristics

Mean

$$\lambda\,\Gamma(1 + 1/k)$$

Median

$$\lambda(\log(2))^{1/k}$$

Mode

$$\begin{cases} \lambda\left(\frac{k-1}{k}\right)^{\frac{1}{k}} & k > 1 \\ 0 & k = 1 \end{cases}$$

Variance

$$\lambda^2\left[\Gamma\left(1+\frac{2}{k}\right) - \left(\Gamma\left(1+\frac{1}{k}\right)\right)^2\right]$$

Relationships

- Relationship pair: $Weibull1(\lambda, k) \rightarrow Weibull2(\lambda, v)$
- Relationship type: Reparameterisation
- Relationship definition: $k = v, \lambda = 1/\lambda^k$

- Relationship pair: $Weibull1(\lambda, k) \rightarrow Exponential1(\lambda_{Exponetial})$
- Relationship type:
- Relationship definition: $k = 1, \lambda_{Exponetial} = 1/\lambda$

- Relationship pair: $Weibull1(\lambda, k) \rightarrow Rayleigh1(\sigma)$
- Relationship type:
- Relationship definition: $k = 2, \lambda = \sqrt{2}\sigma$

- Relationship pair: $GeneralizedGamma2(a, b, c, k) \rightarrow Weibull1(\lambda, k)$

- Relationship type: Special case & Reparameterisation
- Relationship definition: $c = 1, a = 0, b = \lambda$
- Relationship pair: $Weibull2(\lambda, v) \rightarrow Weibull1(\lambda, k)$
- Relationship type: Reparameterisation
- Relationship definition: $v = k, \lambda = \lambda^{-1/v}$

References

[Forbes et al., 2011]
`http://en.wikipedia.org/wiki/Weibull_distribution`
`http://www.uncertml.org/distributions/weibull`

Weibull2

**name**	Weibull 2 (ID: 0000022)
**type**	continuous
**variate**	x, scalar
**support**	$x > 0$

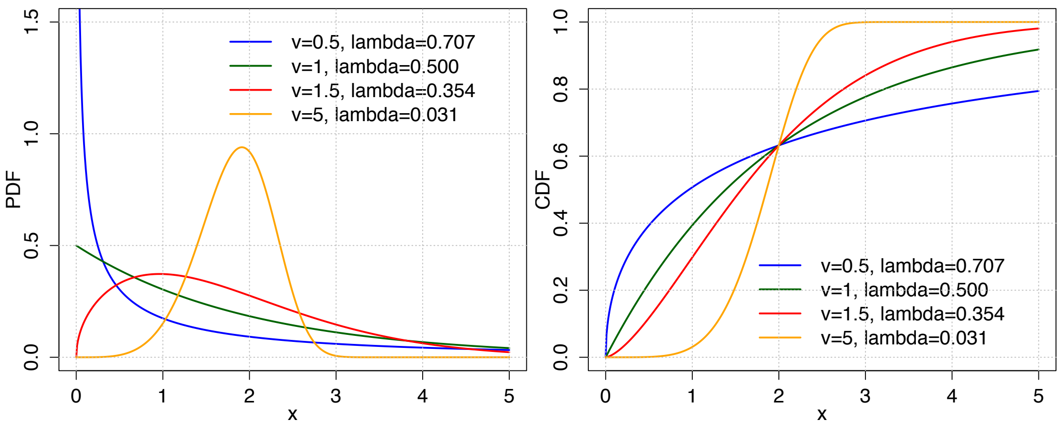

Figure 1.71: Weibull2 distribution plotted using the provided R code.

Parameter: lambda

**name**	lambda
**type**	scalar
**symbol**	λ
**definition**	–

Parameter: shape

**name**	shape
**type**	scalar
**symbol**	v
**definition**	–

Functions

PDF

$$v\lambda x^{v-1} e^{-\lambda x^v}$$

PDF in R

```
v*lambda * x^(v-1) * exp(-lambda * x^v)
```

CDF

$$1 - \exp(-x^v \lambda)$$

CDF in R

```
1- exp(-x^v * lambda)
```

Relationships

- Relationship pair: $Weibull2(\lambda, v) \to Weibull1(\lambda, k)$
- Relationship type: Reparameterisation
- Relationship definition: $v = k, \lambda = \lambda^{-1/v}$

- Relationship pair: $Weibull1(\lambda, k) \to Weibull2(\lambda, v)$
- Relationship type: Reparameterisation
- Relationship definition: $k = v, \lambda = 1/\lambda^k$

References

[Spiegelhalter et al., 2003]

Wishart1

**name**	Wishart 1 (ID: 0000082)
**type**	continuous
**variate**	X, matrix
**support**	$X(p \times p)$ – positive definite matrix

Parameter: scaleMatrix

**name**	scale matrix
**type**	matrix
**symbol**	V
**definition**	$V > 0, p \times p$ – positive definite matrix

Parameter: degreesOfFreedom

**name**	degrees of freedom
**type**	scalar
**symbol**	n
**definition**	$n > p - 1$

Functions

PDF

$$\frac{|X|^{\frac{n-p-1}{2}} e^{-\frac{\mathrm{tr}(V^{-1}X)}{2}}}{2^{\frac{np}{2}} |V|^{\frac{n}{2}} \Gamma_p(\frac{n}{2})}$$

Characteristics

Mean

$$nV$$

Mode

$$(n - p - 1)V \text{ for } n \leq p + 1$$

Variance

$$Var(X_{ij}) = n\left(v_{ij}^2 + v_{ii}v_{jj}\right)$$

References

http://en.wikipedia.org/wiki/Wishart_distribution
http://www.uncertml.org/distributions/wishart

Wishart2

**name**	Wishart 2 (ID: 0000112)
**type**	continuous
**variate**	X, matrix
**support**	$X(p \times p)$ – symmetric, positive definite matrix

Parameter: inverseScaleMatrix

**name**	inverse scale matrix
**type**	matrix
**symbol**	R
**definition**	$p \times p$ – symmetric, positive definite matrix

Parameter: degreesOfFreedom

**name**	degrees of freedom
**type**	scalar
**symbol**	k
**definition**	–

Functions

PDF

$$|R|^{k/2}|x|^{(k-p-1)/2}e^{-\frac{1}{2}\,tr(Rx)}$$

References

[Spiegelhalter et al., 2003]

ZeroInflatedGeneralizedPoisson1

**name**	Zero-Inflated Generalized Poisson 1 (ID: 0000169)
**type**	discrete
**variate**	y, scalar
**support**	$y \in \{0, 1, 2, 3, \dots\}$

Parameter: mean

**name**	mean
**type**	scalar
**symbol**	μ
**definition**	$\mu > 0$

Parameter: dispersion

**name**	dispersion
**type**	scalar
**symbol**	α
**definition**	$\alpha > -1, \alpha \in R$

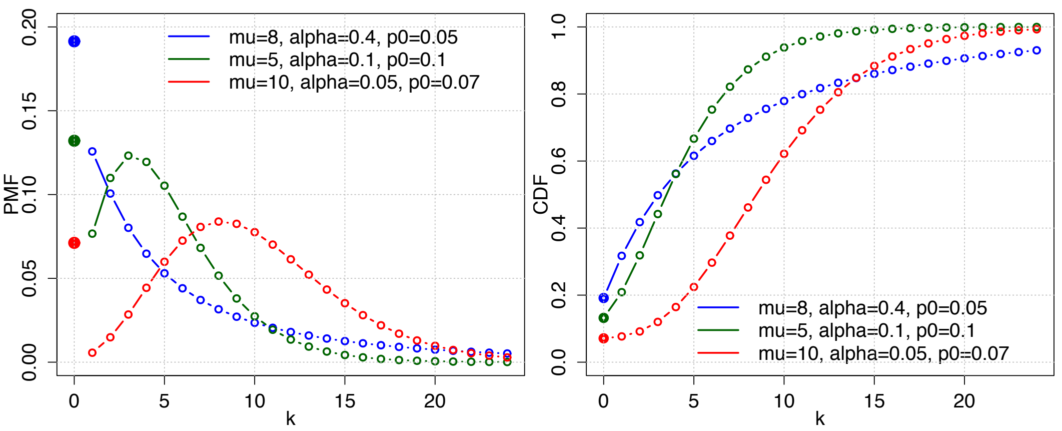

Figure 1.72: ZeroInflatedGeneralizedPoisson1 distribution plotted using the provided R code.

Parameter: probabilityOfZero

**name**	probability of zero
**type**	scalar
**symbol**	$p0$
**definition**	$0 < p0 < 1, p0 \in R$

Functions

PMF

$$\begin{cases} p0 + (1-p0)\exp\left[\frac{-\mu}{1+\alpha\mu}\right] & \text{for } y = 0 \\ (1-p0)\left(\frac{\mu}{1+\alpha\mu}\right)^{y}\frac{(1+\alpha y)^{y-1}}{y!}\exp\left[\frac{-\mu(1+\alpha y)}{1+\alpha\mu}\right] & \text{for } y > 0 \end{cases}$$

PMF in R

```
PMF1=p0 + (1-p0) * exp(-mu/(1+ alpha*mu)) # for y = 0
PMF2=(1-p0)*( mu/(1+alpha*mu))^y*((1+alpha*y)^(y-1))/factorial(y)*
exp((-mu*(1+alpha*y))/(1+alpha*mu)) # for y > 0
```

CDF

$$\Sigma_{i=1}^{x} f(i), x \in \{0, 1, 2, ...\} \text{ with } f \text{ the PMF}$$

CDF in R

```
c(PMF1,cumsum(PMF2)+PMF1)
```

Characteristics

Mean

$$(1-p0)\mu$$

Variance

$$(1-p0)[\mu^2 + \mu(1+\alpha\mu)^2] - (1-p0)^2\mu^2$$

Relationships

- Relationship pair: $ZeroInflatedGeneralizedPoisson1(\mu, \alpha, p0) \rightarrow GeneralizedPoisson3(\mu, \alpha)$
- Relationship type: Special case
- Relationship definition: $p0 = 0$

- Relationship pair: $ZeroInflatedGeneralizedPoisson1(\mu, \alpha, p0) \rightarrow Poisson1(\lambda)$
- Relationship type: Special case & Reparameterisation
- Relationship definition: $p0 = 0, \alpha = 0, \lambda = \mu$

- Relationship pair: $ZeroInflatedGeneralizedPoisson1(\mu, \alpha, p0) \rightarrow ZeroInflatedPoisson1(\lambda, \pi)$
- Relationship type: Special case & Reparameterisation
- Relationship definition: $\alpha = 0, \lambda = \mu$

References

[Famoye and Singh, 2006], [Trocóniz et al., 2009]

ZeroInflatedNegativeBinomial1

**name**	Zero-Inflated Negative Binomial 1 (ID: 0000142)
**type**	discrete
**variate**	k, scalar
**support**	$k \in \{0, 1, 2, 3, \dots\}$

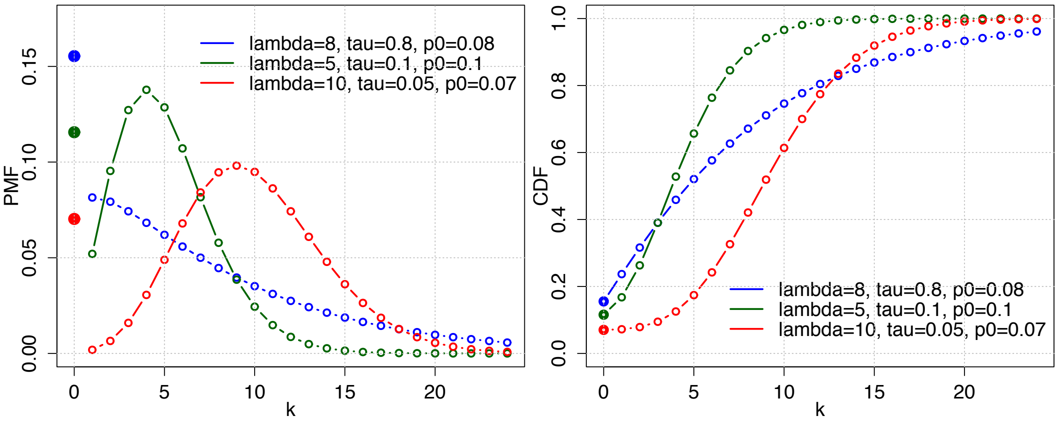

Figure 1.73: ZeroInflatedNegativeBinomial1 distribution plotted using the provided R code.

Parameter: rate

**name**	mean
**type**	scalar
**symbol**	λ
**definition**	$\lambda > 0$

Parameter: overdispersion

**name**	overdispersion
**type**	scalar
**symbol**	τ
**definition**	–

Parameter: probabilityOfZero

**name**	probability of zero
**type**	scalar
**symbol**	$p0$
**definition**	$0 < p0 < 1, p0 \in R$

Functions

PMF

$$\begin{cases} p0 + (1-p0)\left(\frac{1}{1+\tau\lambda}\right)^{1/\tau} & \text{for } y = 0 \\ (1-p0)\frac{\Gamma(y+1/\tau)}{y!\Gamma(1/\tau)}\left(\frac{1}{1+\tau\lambda}\right)^{1/\tau}\left(\frac{\lambda}{1/\tau+\lambda}\right)^{y} & \text{for } y > 0 \end{cases}$$

PMF in R

```
PMF1=p0 + (1-p0) * (1/ (1 + tau * lambda))^(1/tau) # for y=0
PMF2=(1-p0) * gamma(y+1/tau) / (factorial(y) *gamma(1/tau)) * (1/(1 + tau*lambda))^(1/tau) *
 (lambda/(1/tau + lambda))^y # for y>0
```

CDF

$$\Sigma_{i=1}^{x} f(i), x \in \{0, 1, 2, ...\} \text{ with } f \text{ the PMF}$$

CDF in R

```
c(PMF1,cumsum(PMF2)+PMF1)
```

Characteristics

Mean

$$\mu$$

Relationships

- Relationship pair: $ZeroInflatedNegativeBinomial1(\lambda, \tau, p0) \rightarrow NegativeBinomial2(\lambda, \tau)$
- Relationship type: Special case
- Relationship definition: $p0 = 0$

- Relationship pair: $ZeroInflatedNegativeBinomial1(\lambda, \tau, p0) \rightarrow ZeroInflatedPoisson1(\lambda, \pi)$
- Relationship type: Limiting
- Relationship definition: $\tau \rightarrow 0$

- Relationship pair: $ZeroInflatedNegativeBinomial1(\lambda, \tau, p0) \rightarrow Poisson1(\lambda)$
- Relationship type: Limiting
- Relationship definition: $p0 = 0, \tau \rightarrow 0$

References

[Famoye and Singh, 2006], [Trocóniz et al., 2009]

ZeroInflatedPoisson1

**name**	Zero-inflated Poisson 1 (ID: 0000197)
**type**	discrete
**variate**	k, scalar
**support**	$k \in \{0, 1, 2, 3, \dots\}$

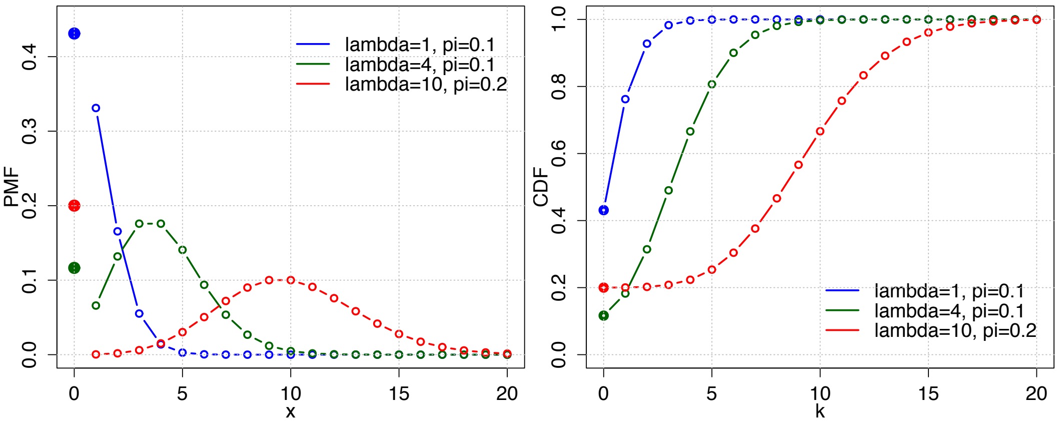

Figure 1.74: ZeroInflatedPoisson1 distribution plotted using the provided R code.

Parameter: rate

**name**	Poisson intensity
**type**	scalar
**symbol**	λ
**definition**	$\lambda \in R, \lambda > 0$

Parameter: probabilityOfZero

**name**	probability of extra zeros
**type**	scalar
**symbol**	π
**definition**	$0 < \pi < 1, \pi \in R$

Functions

PMF

$$\begin{cases} \pi + (1-\pi)e^{-\lambda} & \text{for } k = 0 \\ (1-\pi)e^{-\lambda}\frac{\lambda^k}{k!} & \text{for } k > 0 \end{cases}$$

PMF in R

```
PMF1=pi + (1-pi)*exp(-lambda) # if k=0
PMF2=(1-pi)*exp(-lambda) * lambda^k/factorial(k) # if k>0
```

CDF

$$\Sigma_{i=1}^{x} f(i), x \in \{0, 1, 2, ...\} \text{ with } f \text{ the PMF}$$

CDF in R

```
c(PMF1,cumsum(PMF2)+PMF1)
```

Characteristics

Mean

$$(1-\pi)\lambda$$

Variance

$$\lambda(1-\pi)(1+\lambda\pi)$$

Relationships

- Relationship pair: $ZeroInflatedPoisson1(\lambda, \pi) \rightarrow Poisson1(\lambda)$
- Relationship type: Special case
- Relationship definition: $\pi = 0$

- Relationship pair: $ZeroInflatedNegativeBinomial1(\lambda, \tau, p0) \rightarrow ZeroInflatedPoisson1(\lambda, \pi)$
- Relationship type: Limiting
- Relationship definition: $\tau \rightarrow 0$

- Relationship pair: $ZeroInflatedGeneralizedPoisson1(\mu, \alpha, p0) \rightarrow ZeroInflatedPoisson1(\lambda, \pi)$
- Relationship type: Special case & Reparameterisation
- Relationship definition: $\alpha = 0, \lambda = \mu$

References

[Famoye and Singh, 2006], [Trocóniz et al., 2009], [Plan, 2014]
http://en.wikipedia.org/wiki/Zero-inflated_model#Zero-inflated_Poisson

Bibliography

[Cameron and Trivedi, 2013] Cameron, A. C. and Trivedi, P. K. (2013). *Regression analysis of count data*, volume 53. Cambridge university press.

[Consul and Famoye, 2006] Consul, P. C. and Famoye, F. (2006). *Lagrangian probability distributions.* Birkhäuser, Boston.

[Famoye and Singh, 2006] Famoye, F. and Singh, K. P. (2006). Zero-inflated generalized poisson regression model with an application to domestic violence data. *Journal of Data Science*, 4(1):117–130.

[Forbes et al., 2011] Forbes, C., Evans, M., Hastings, N., and Peacock, B. (2011). *Statistical distributions.* John Wiley & Sons.

[Gelman et al., 2014] Gelman, A., Carlin, J. B., Stern, H. S., Dunson, D. B., Vehtari, A., and Rubin, D. B. (2014). Bayesian data analysis.

[Hilbe, 2011] Hilbe, J. M. (2011). *Negative binomial regression.* Cambridge University Press.

[Ismail and Zamani, 2013] Ismail, N. and Zamani, H. (2013). Estimation of claim count data using negative binomial, generalized poisson, zero-inflated negative binomial and zero-inflated generalized poisson regression models. In *Casualty Actuarial Society E-Forum, Spring 2013.*

[Leemis and Mcqueston, 2008] Leemis, L. M. and Mcqueston, J. T. (2008). Univariate distribution relationships. *The American Statistician*, 62(1):45–53.

[Limpert et al., 2001] Limpert, E., Stahel, W. A., and Abbt, M. (2001). Log-normal distributions across the sciences: Keys and clues. *BioScience*, 51(5):341–352.

[Lunn, 2012] Lunn, D. (2012). *The BUGS book: a practical introduction to Bayesian analysis.* Texts in statistical science. CRC Press.

[Marichev and Trott, 2013] Marichev, O. and Trott, M. (2013). The Ultimate Univariate Probability Distribution Explorer.

[MathWorks, 2015] MathWorks (2015). *MATLAB and Statistics Toolbox.* The MathWorks Inc., Natick, Massachusetts.

[Plan, 2014] Plan, E. L. (2014). Modeling and simulation of count data. *CPT Pharmacometrics Syst Pharmacol*, 3:e129.

[Plummer, 2003] Plummer, M. (2003). JAGS: A Program for Analysis of Bayesian Graphical Models Using Gibbs Sampling.

[Shmueli et al., 2005] Shmueli, G., Minka, T. P., Kadane, J. B., Borle, S., and Boatwright, P. (2005). A useful distribution for fitting discrete data: revival of the conway–maxwell–poisson distribution. *Journal of the Royal Statistical Society: Series C (Applied Statistics)*, 54(1):127–142.

[Spiegelhalter et al., 2003] Spiegelhalter, D., Thomas, A., Best, N., and Lunn, D. (2003). WinBUGS User Manual, Version 1.4.

[Stacy, 1962] Stacy, E. W. (1962). A generalization of the gamma distribution. *The Annals of Mathematical Statistics*, pages 1187–1192.

[STAN Development Team, 2015] STAN Development Team (2015). Stan Modeling Language Users Guide and Reference Manual, Version 2.9.0.

[Trocóniz et al., 2009] Trocóniz, I. F., Plan, E. L., Miller, R., and Karlsson, M. O. (2009). Modelling overdispersion and markovian features in count data. *J Pharmacokinet Pharmacodyn*, 36(5):461–77.

[Yanagimoto, 1989] Yanagimoto, T. (1989). The inverse binomial distribution as a statistical model. *Communications in Statistics-Theory and Methods*, 18(10):3625–3633.

[Yang et al., 2007] Yang, Z., Hardin, J. W., Addy, C. L., and Vuong, Q. H. (2007). Testing approaches for overdispersion in poisson regression versus the generalized poisson model. *Biom J*, 49(4):565–84.
