# Supplementary material for: ProbOnto: ontology and knowledge base of probability distributions
Source: Bioinformatics. 2016 Apr 3;32(17):2719–21. doi: 10.1093/bioinformatics/btw170 (PMC5013898; doi:10.1093/bioinformatics/btw170)
Supplement: Supplementary Data [file supp_btw170_Swat_et_al_Supplementary2_ProbOnto_useInPharmML.pdf]

SUPPLEMENTARY MATERIALS – PART 2/2

M.J. Swat et al.

ProbOnto – Ontology and Knowledge Base of Probability Distributions

ProbOnto usage in PharmML

VERSION 1.0 (MARCH 2016)

FOR UPDATES VISIT HTTP://PROBONTO.ORG

Contents

1 Introduction **3**
- 1.1 Ontology versus Knowledge Base . . . 4
- 1.2 Limitations in UncertML application in PharmML . . . 4
- 1.3 ProbOnto Features . . . 5
- 1.4 Working with ProbOnto . . . 6
  - 1.4.1 Example 1 . . . 6
  - 1.4.2 PharmML elements supporting ProbOnto . . . 6
- 1.5 Annotation of models with ProbOnto ontology . . . 7
  - 1.5.1 Implementation in PharmML . . . 7
  - 1.5.2 Annotation of PharmML . . . 7
  - 1.5.3 Background Information is Contained in ProbOnto . . . 7
- 1.6 Relationships . . . 8
- 1.7 Independency of ProbOnto . . . 9

2 Alternative parameterisations **11**
- 2.1 Negative binomial distribution . . . 11
  - 2.1.1 Re-parameterisation formulas . . . 12
- 2.2 Normal distribution . . . 12
  - 2.2.1 Re-parameterisation formulas . . . 12
- 2.3 Log-normal distribution . . . 13
  - 2.3.1 Re-parameterisation formulas . . . 14

3 Use Examples **16**
- 3.1 Count data models . . . 16
  - 3.1.1 Zero-inflated Poisson – ZIP . . . 16
  - 3.1.2 Poisson with mixtures – PMIX . . . 17
  - 3.1.3 Other count data models . . . 17
- 3.2 Categorical data models . . . 19
  - 3.2.1 Nominal categorical model . . . 19
- 3.3 Truncated distributions . . . 20
- 3.4 Complete PharmML model . . . 21

A Distributions in ProbOnto **22**
- A.1 Univariate distributions . . . 23
  - A.1.1 Tool coverage . . . 23
  - A.1.2 Code names . . . 24
- A.2 Mixture distribution . . . 26
- A.3 Multivariate distributions . . . 27
  - A.3.1 Tool coverage . . . 27
  - A.3.2 Code names . . . 27

A Parameterisations of the negative binomial (NB1 vs NB4) **28**
- A.1 Differences explained . . . 29
- A.2 Comparison of formulation support . . . 30

References **31**

List of Figures

1.1 Illustration of possible model misspecification when using incorrect parameterisation 3
1.2 PMF and CDF of the Poisson distribution plotted using the R-code stored in ProbOnto. . . . 8
1.3 Relationships of Normal1 distribution which are a subset of the network shown in Figure 1.4. 9
1.4 ProbOnto 1.0 – relationships and re-parameterisations diagram 10

2.1 Schematic representation of the lognormaly distributed data on the natural and logarithmic scale 13
2.2 Representation of the lognormaly distributed basal insulin data in diabetic patients 14
2.3 Re-parameterisation relationships implemented in ProbOnto 15

3.1 Count data models and the relationships between models stored in ProbOnto 18
3.2 Truncated normal distribution 21

A.1 Comparison of PMF and CDF plots for *Option 1* and *Option 2* 28

List of Tables

A.1 Univariate distributions supported in ProbOnto with overview of tool support. 24
A.2 Code names for distribution and parameter names of the univariate distributions 26
A.3 Mixture distribution with overview of tool support. 26
A.4 Code names for mixture distribution – defined for univariate distributions only. 26
A.5 Multivariate distributions with overview of tool support. 27
A.6 Code names for the multivariate distributions and their parameters. 27

A.1 UncertML versus R 29
A.2 Overview of different formulation types of NB 31

Chapter 1

Introduction

Background and motivation ProbOnto was first designed to facilitate the encoding of nonlinear mixed effect models and their annotation in the Pharmacometrics Markup Language (PharmML) [Swat et al., 2015] developed by the DDMoRe consortium [Harnisch et al., 2013].

When encoding probabilistic uncertainties using a parametric distribution, its name and parameters are usually sufficient to specify it unambiguously as in most cases such parameter set are unique. But, because for a number of cases two or more parameterisations exist, one needs to be precise which parameters are used when referring to a distribution, otherwise one might end up with a wrong model (see for an example Figure 1.1). For this purpose, an external standard reference is very useful as it requires less effort to declare the required distribution in an exchange format or programming language.

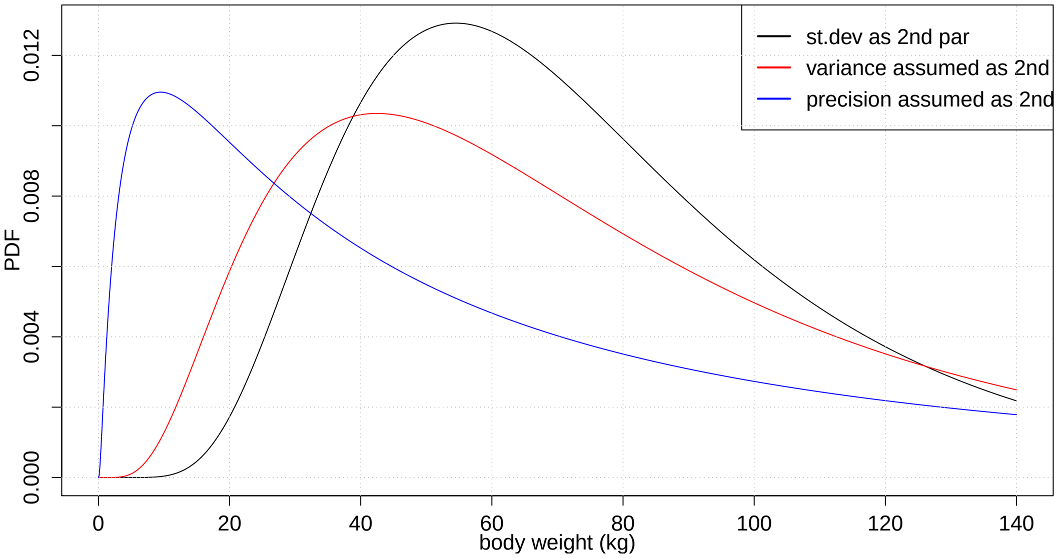

Figure 1.1: Illustration of possible model misspecification when using incorrect parameterisation. (1) The black curve corresponds to a log-normal distribution of body weight, $\mathcal{LN}(\mu = \log(70), \sigma = 0.5)$, the intended parameterisation. (2) Here it was mistakenly assumed that 0.5 corresponds to the variance and calculation of standard deviation as required by the R function *dlnorm* gives $\sigma = \sqrt{0.5} = 0.707$ (red). (3) Here the modeller assumed that the 2nd input value corresponds to the precision and calculated the standard deviation as $\sigma = 1/\sqrt{0.5} = 1.41$ (blue). Small numerical differences in σ on the log-scale, result in significant differences on the natural scale.

Until now, we have been relying on UncertML, a high quality reference resource for encapsulating probabilistic uncertainties, [UncertML Team, 2014], [Williams et al., 2008]. It facilitates the encoding of a range of continuous and discrete uni/multi-variate probability distributions. However, from the perspective of PharmML, it has some limitations as described in section 1.2.

Status Quo The initial motivation for *ProbOnto* was to create an ontology of probability distributions purely for annotation purposes. Many resources are available online and in printed format but no suitable

ontology exists so far[1]. Similarly, the databases of distributions available online come with analog issues. The largest and most comprehensive known to us collection of probability distributions, the UUPDE by [Marichev and Trott, 2013]. With up to 60 properties for each of its 500 probability distributions it is an invaluable resource for parametric distributions. Unfortunately, it comes with univariate cases only, still lacks a number of distributions and doesn't provide references or parameter names making its use in our context impracticable[2].

Idea It turns out that *ProbOnto* can be very helpful in designing a flexible alternative for UncertML with many additional features. It can be used in PharmML or other target tools/languages *both* as an ontological resource for annotation purposes and as a knowledge base, providing the means to specify a wide range of distributions, distribution related functions and quantities.

In fact, such a solution is indispensable in the face of requirements posed by the models we would like to encode, both currently and in the foreseeable future.

1.1 Ontology versus Knowledge Base

The following definitions are based on a post by Jeen Broekstra found on the *dataversity* discussion forum[3].

Ontology is a formal representation of a domain of knowledge. It is an abstract entity defining the vocabulary for a domain and the relations between concepts. However, an ontology doesn't specify how that knowledge is stored (as physical file, in a database, or in some other form), and how the knowledge can be accessed.

Knowledge base is a physical artifact. It is a database, a repository of information that can be accessed and manipulated in some predefined fashion.

The information stored in a knowledge base is modelled according to rules and relationships defined in an ontological model.

1.2 Limitations in UncertML application in PharmML

Although very useful to a certain extent, there are limitations in the design and scope of UncertML making the encoding of statistical models as used in pharmacometrics cumbersome (e.g. especially when a required distribution is not available). Here some known limitations:

- it doesn't support the assignment of expressions for distribution parameters or the specification of block references, which is required if the parameter in question is defined elsewhere in the model.
- it doesn't cover many distributions used in Pharmacometrics, e.g.
  - discrete ones such as Generalized Poisson or Zero-inflated Poisson, see section 3.1.3
  - alternative parameterisations for distributions such as Negative Binomial, Log-Normal etc.
  - multivariate continuous distributions such as Inverse-Wishart.
- UncertML is a reference resource for distributions but does not provide mechanisms to retrieve programmatically related functions and quantities.
- every extension requires changes in the already complex XML schema.

Other minor issues:

- Negative Binomial (NB1) comes in an unusual formulation (as opposed to just different parameterisation) which is rarely seen in the literature. It is different from the formulation supported by R, Matlab [MathWorks, 2015] and winBUGS and might go unnoticed to an unexperienced user, leading to false results. A detailed discussion can be found in the appendix A.

[1] For example, the Statistics Ontology, STATO, `http://stato-ontology.org/`, provides for most distributions merely a link to an external reference/definition. No parameters or related functions and quantities are defined in the ontology making their annotation impossible. Other ontologies, we have analysed number of them featured in the BioPortal, [Noy et al., 2009], suffer from equivalent limitations as they are designed in a similar way.

[2] Another example is Distributome, `http://www.distributome.org/`, which comes with an impressive and well-referenced collection of 90+ distributions but doesn't contain many of relevant for us types and/or parameterisations and is limited to univariate parametric ones.

[3] `http://answers.semanticweb.com/questions/21500/what-is-the-difference-between-knowledge-base-and-ontology`

- `<degreesOfFreedom>` parameter element of the Wishart distribution doesn't support referencing a variable (required for Bayesian inference)
- the implementation of `<MultivariateNormalDistribution>` requires the specification of the `dimension` attribute of the covariance matrix – although this can be estimated it requires unnecessary calculations when translating models to PharmML.
- version 3.0 which we currently use is not yet released publicly, the UncertML website is not updated and 3.0 documentation is not available.

UncertML extension A seemingly easy solution would be to extend UncertML, but to do so it would require the introduction of major extensions and changes to the XML schema. Only the support of the most important missing features would *de facto* require a rewrite of the entire standard, as UncertML doesn't possess the structure to encode algebraic expressions. This would most certainly result in a different implementation of the mathematical notation, compared to PharmML, resulting in inconsistent, layered and/or overlapping schemas, difficult to handle and to validate.

Suggested way forward ProbOnto offers an alternative solution avoiding all the limitations listed above, while providing a number of additional features and means to build in a very flexible probability distribution support in MDL, PharmML and other languages/tools within DDMoRe and beyond.

1.3 ProbOnto Features

The Knowledge Base had been build based on the UncertML standard collection of 28 parametric distributions. Following its implementation we extended it for models used in pharmacometrics. We have added a number of most essential discrete data models, all distributions and/or their parameterisations used in Monolix and winBUGS, the majority of distributions supported by the Matlab Statistical Toolbox and many relationships between distributions. New distributions and relationships are being added on regular basis, as necessary.

In the following list we summarise ProbOnto 1.0 features (January 2016)

- General
  - More than 80 distributions and alternative parameterisations.
  - Over 130 relationships and re-parameterisation formulas.
  - Supports encoding of univariate mixture distributions and truncation bounds (open/closed).
  - Allows for easy encoding of distributions and related functions in target tools/languages thanks to its generic format.
  - Doesn't enforce specific implementation in target tools.
  - For the use of ProbOnto in PharmML a small generic schema has been designed providing flexible encoding support for all distributions and their features, see section 1.4.
  - Collection of supported distributions, see appendix A for a list of available distributions and their essential features, is easily extendable.
  - All mathematical functions and quantities are available in Latex and additionally for functions R-code is provided.
- ProbOnto as Ontology
  - Can be used to annotate statistical models based on supported probability distributions, e.g. their name, parameters, truncation bounds, their defining functions and quantities.
- ProbOnto as Knowledge Base
  - Provides, for each distribution, either PDF or PMF and in many cases also other distribution related functions such as CDF, hazard and survival functions – the level of coverage depends on the particular distribution,
  - related quantities such as mean, median, mode, variance,
  - info about support/range, relationships to other distributions,
  - application examples and

– references.

The distribution collection and their features are based on the project by [UncertML Team, 2014], probability distribution pages of the English Wikipedia[4], [Forbes et al., 2011], [Leemis and Mcqueston, 2008], [Johnson et al., 2005], [Song and Chen, 2011] and [Weisstein, 2007] to name the main sources.

1.4 Working with ProbOnto

The subsequent chapters come with a number of examples of ProbOnto use but it is worth highlighting two basic implementation rules

- The name of a distribution, encoded in the <ProbOnto name="..."> tag, where instead of the dots one of the 'Code names' assigned to each distribution in ProbOnto must be used.
- The same holds for the parameters of a distribution, encoded in the <Parameter name="..."> tag. Note that the order of parameters doesn't matter.

To remain consistent with the nomenclature used so far in PharmML and MDL which was based on the UncertML vocabulary, the majority of parameter *code names* is identical to those used in UncertML. For new distributions and their parameters we have defined the most common names used in the literature. The *code names* are collected in the tables A.2, A.6 and A.4 of the appendix.

1.4.1 Example 1

The implementation of the negative binomial model illustrates how this works. There are many parameterisations for this distribution but the version with Poisson intensity, λ, and over-dispersion, τ, as parameters, code name *NegativeBinomial2*, is used here for demonstration

```
<Distribution>
    <ProbOnto name="NegativeBinomial1">
        <Parameter name="rate">
            <ct:Assign>
                <ct:SymbRef blkIdRef="pm1" symbIdRef="rabbit"/>
            </ct:Assign>
        </Parameter>
        <Parameter name="overdispersion">
            <ct:Assign>
                <ct:SymbRef blkIdRef="pm2" symbIdRef="piggy"/>
            </ct:Assign>
        </Parameter>
    </ProbOnto>
</Distribution>
```

According to the rules, the names of the distributions and their parameters must be the code names defined by ProbOnto, see table A.6. The user can then assign any symbols to the parameters, with *rabbit* for *rate*, defined in parameter model pm1 and *piggy* for *overdispersion*, defined in parameter model pm2.

1.4.2 PharmML elements supporting ProbOnto

ProbOnto has been designed to be applicable in any target tool and comes without any limitations or requirements with respect to its use in a given software. For use within PharmML we provide an XML schema which can be easily updated, e.g. when new distributions are added to the knowledge base. After adding a new distribution to the knowledge base, such extensions of the schema consist merely in adding new values to the name attributes.

The following elements support ProbOnto distribution encoding

- <ProbOnto> tag with the name attribute for the distribution code names with following child elements
  - <Parameter> with the name attribute for the parameter code names. It can be assigned any expression.
  - <LowerTruncationBound> and <UpperTruncationBound> to indicate the truncation bounds for univariate distributions with attribute type which can be either *closed* or *open*.
  - <MixtureComponent> with the name attribute for the code name of mixture component.

[4]See the list of distributions on Wikipedia at https://en.wikipedia.org/wiki/List_of_probability_distributions

1.5 Annotation of models with ProbOnto ontology

1.5.1 Implementation in PharmML

The following code shows the typical Poisson model implementation

```
<PMF>
   <Distribution>
      <ProbOnto id="X1" name="Poisson1">
         <Parameter id="X2" name="rate">
            <ct:Assign>
               <ct:SymbRef symbIdRef="lambda"/>
            </ct:Assign>
         </Parameter>
      </ProbOnto>
   </Distribution>
</PMF>
```

1.5.2 Annotation of PharmML

Notice that in the above code snippet the elements defining the distribution used, <ProbOnto>, and its parameter, <Parameter> are given identifiers, `id="X1"` and `id="X2"`, respectively. This allows us to annotate these elements so that we can make explicit their intended interpretation. Using the PharmML metadata annotation schema, we would record such interpretation using the property *has-interpreted-type*. In what follows, 'ps' abbreviates the namespace for this schema and 'probonto' abbreviates the namespace for the ProbOnto ontology, part of the stack of ontologies used in PharmML annotation.

> # The distribution element is interpreted as an instantiation of the Poisson distribution.
>
> *X1 ps:has-interpreted-type probonto:0000111.*
>
> # The parameter element is interpreted as an instantiation of the parameter element, λ, of the Poisson distribution.
>
> *X2 ps:has-interpreted-type probonto:0000114.*

In ProbOnto, *0000111* and *0000114* are the identifiers for the Poisson distribution and its (unique) parameter, respectively. The two statements above encode the interpretation of the PharmML code defining the distribution. Such statements can in principle be generated automatically after processing the PharmML code.

Annotating the actual PharmML model and the element to which the distribution applies would involve more or, a variation upon, the above to the effect that the ProbOnto distribution is identified.

1.5.3 Background Information is Contained in ProbOnto

Given the annotation of the PharmML code linking to ProbOnto, we can then use ProbOnto to make explicit all the information that is packed into these two very terse annotation statements.

Underlying accessible knowledge about Poisson distribution

We thus have access to the following regarding the distribution contained in the PharmML code (as much as is contained in the ProbOnto definition of the Poisson distribution):

**name**	Poisson (ID: 0000111)
**type**	discrete
**variate**	k, scalar
**support**	$k \in \{0, 1, 2, 3, \dots\}$

Additionally, we can obtain from ProbOnto the following type of information.

Underlying accessible knowledge about the related functions

PMF

$$\frac{\lambda^k}{k!} e^{-\lambda}$$

PMF in R

```
lambda^k/factorial(k) * exp(-lambda)
```

CDF

$$\frac{\Gamma(\lfloor k+1 \rfloor, \lambda)}{\lfloor k \rfloor !}$$

CDF in R

```
Igamma(floor(k+1), lambda, lower=F) / factorial(floor(k))
```

using *Igamma* function from the *zipfR* package, `http://cran.r-project.org/web/packages/zipfR/zipfR.pdf`.

Underlying accessible knowledge about the (rate) parameter

**name**	Poisson intensity (ID: 0000114)
**type**	scalar
**symbol**	λ
**definition**	$\lambda \in R, \lambda > 0$

The amount of information that may be encoded in ProbOnto is extensible. Thus, via a very simple and direct mechanism of annotation that amounts to linking a distribution and its parameter(s) in a piece of PharmML code, we can inherit and obtain all the background relevant information. This knowledge can be used either for our understanding and the validation of our PharmML encoding or, with adequate software support, for processing by tools.

Currently, such extensive software support is not available but is part of the development path for PharmML and its implementation of ProbOnto.

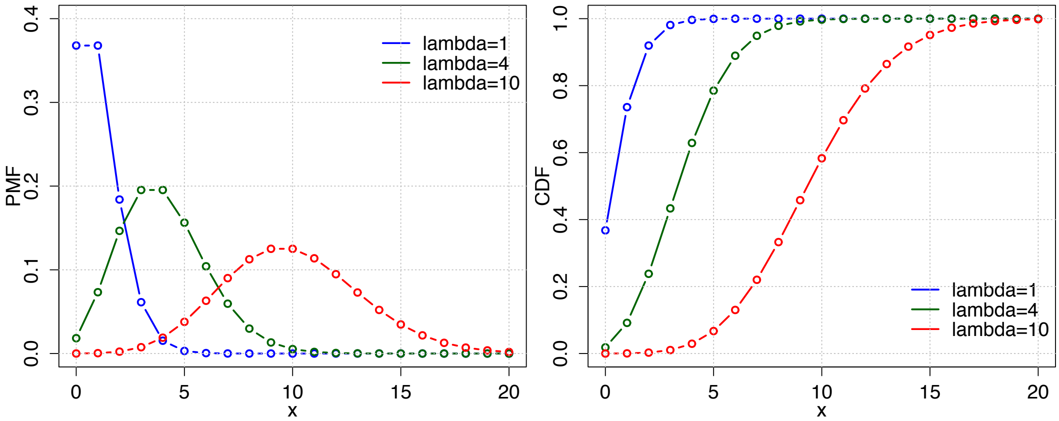

Figure 1.2: PMF and CDF of the Poisson distribution plotted using the R-code stored in ProbOnto.

1.6 Relationships

Relationships which connect different distributions is what makes an ontology and knowledge base so useful. Information of this type can provide a modeller with hints about alternative models applicable to a particular dataset/experiment. As an example, consider the normal distribution in its *Normal1* parameterisation connected to multiple distributions, both discrete and continuous ones, see Figure 1.3. Another interesting subset of these relationships are re-parameterisations within a given distribution family, described in detail in chapter 2. We distinguish the following relationship types and their combinations:

- Approximation/limiting – type of relationships following when a parameter approaches a certain value or infinity.
- Re-parameterisation – relationship between distributions of the same family.
- Special case – when one distribution is a special case of another with a broader parameter space.
- Transformation – when a variate with one distribution is a function of (an)other variate(s) with different distributions.

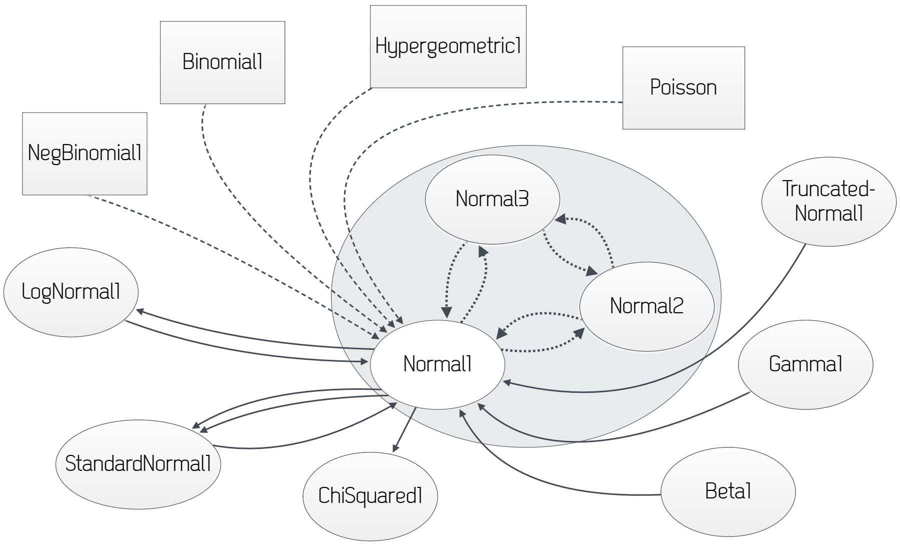

Figure 1.3: Relationships of Normal1 distribution which are a subset of the network shown in Figure 1.4.

The following list summarises the current coverage of relationships between various distributions, most of them being represented in figure 1.4.

- More then 70 relationships between various distributions.
- About 60 re-parameterisations between the members of distributions families, such as normal, log-normal, negative binomial, generalised Poisson and others.

1.7 Independency of ProbOnto

It is worth stressing that the ProbOnto ontology and knowledge base are fully independent from PharmML. ProbOnto also doesn't enforce specific implementation from tool designers – this allows it to be used across the DDMoRe target tools, languages and beyond.

For the purpose of the use in PharmML we have developed a schema which is tailored towards NLME models as used in pharmacometrics and is otherwise entirely optional. See chapter 3 for examples illustrating ProbOnto use in PharmML.

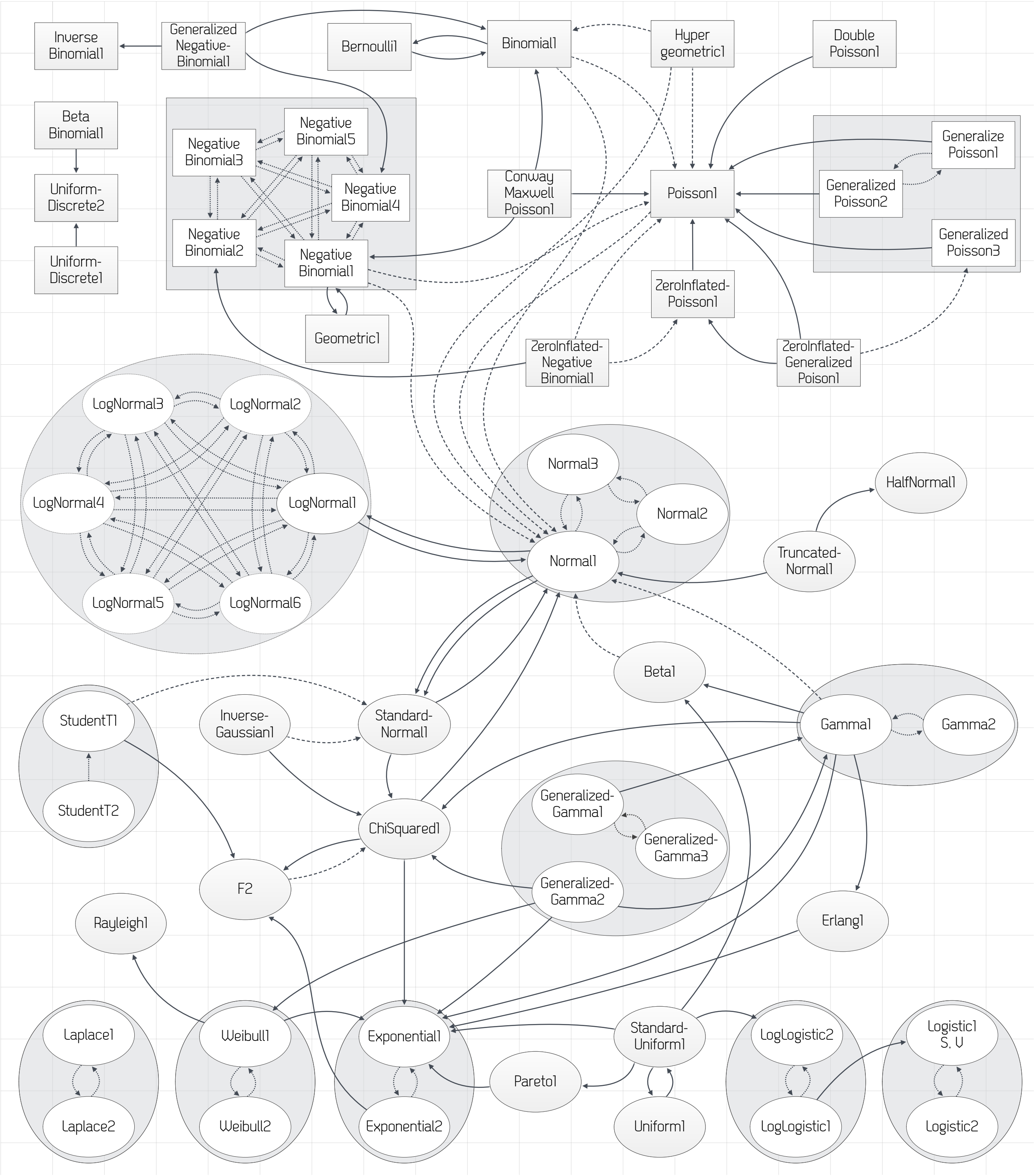

Figure 1.4: ProbOnto 1.0 – relationships and re-parameterisations diagram. Over 130 connections distributions are shown for those 60 distributions for which at least one connection exists.

Chapter 2

Alternative parameterisations

The existence of alternative parameterisations is apparent for number of reasons, such as model type, application area, available data and/or target tool – e.g. BUGS using precision, τ, rather then standard deviation or variance for a normal/log-normal and a number of other distributions. ProbOnto contains, wherever available, re-parameterisations formulas of distributions coming in different forms. This can be seen as an improvement of the inter-operability support between target tools supporting different parameter sets for a given probability density/mass function.

The needs for the support of re-parameterisation, e.g. between BUGS and R has been discussed before, [LeBauer et al., 2013]. The authors state that "[...] R and BUGS languages use different representations of common probability density functions, creating a potential for errors to occur in the implementation or interpretation of analyses that use both languages". While this paper addresses only two tools and five distributions, we aim to cover a much larger scope, with respect to both number of tools and parameterisations, see Figure 1.4.

Typical examples for alternative parameterisations are given in next sections. We provide for clarity the defining probability density/mass functions and the according formulas required to go from one form to another.

2.1 Negative binomial distribution

The available parameterisations are

- NegativeBinomial1(r, p) with r – *number of successes* and p – *success probability*

$$P(k;r,p) = \binom{k+r-1}{k} p^r (1-p)^k, \quad k \in \{0,1,2,\dots\} \text{ number of failures}$$

- NegativeBinomial4(r, p) with r – *number of failures* and p – *success probability*

$$P(k;r,p) = \binom{k+r-1}{k} (1-p)^r p^k, \quad k \in \{0,1,2,\dots\} \text{ number of successes}$$

- NegativeBinomial2(λ, τ), [Plan et al., 2009], with λ – *mean* and τ – *over-dispersion*,

$$P(k;\lambda,\tau) = \frac{\Gamma(k+\frac{1}{\tau})}{k!\,\Gamma(\frac{1}{\tau})} \Big(\frac{1}{1+\tau\lambda}\Big)^{\frac{1}{\tau}} \Big(\frac{\lambda}{\frac{1}{\tau}+\lambda}\Big)^k, \quad k \in \{0,1,2,\dots\}$$

- NegativeBinomial3(μ, ϕ), [STAN Development Team, 2015b],

$$P(k;\mu,\phi) = \binom{k+\phi-1}{k} \Big(\frac{\phi}{\mu+\phi}\Big)^{\phi} \Big(\frac{\mu}{\mu+\phi}\Big)^k, \quad k \in \{0,1,2,\dots\}$$

- NegativeBinomial5(α, β), [STAN Development Team, 2015b],

$$P(k;\alpha,\beta) = \binom{k+\alpha-1}{\alpha-1} \Big(\frac{\beta}{\beta+1}\Big)^{\alpha} \Big(\frac{1}{\beta+1}\Big)^k, \quad k \in \{0,1,2,\dots\}$$

with NB2 being used in typical pharmacometric discrete data models, [Plan et al., 2009, Trocóniz et al., 2009].

2.1.1 Re-parameterisation formulas

- $\mathbf{NB1}(r,\, p) \to \mathbf{NB2}(\lambda,\, \tau)\ :\ \tau = 1/r;\quad \lambda = \frac{r(1-p)}{p}$
  $\mathbf{NB2}(\lambda,\, \tau) \to \mathbf{NB1}(r,\, p)\ :\ r = 1/\tau;\quad p = \frac{1}{1+\tau\lambda}$
- $\mathbf{NB1}(r,\, p) \to \mathbf{NB3}(\mu,\, \phi)\ :\ \phi = r;\quad \mu = \frac{r(1-p)}{p}$
  $\mathbf{NB3}(\mu,\, \phi) \to \mathbf{NB1}(r,\, p)\ :\ r = \phi;\quad p = \frac{r}{\mu+r}$
- $\mathbf{NB1}(r,\, p) \leftrightarrow \mathbf{NB4}(r,\, p)\ :\ p \to 1-p$
- $\mathbf{NB1}(r,\, p) \to \mathbf{NB5}(\alpha,\, \beta)\ :\ \alpha = r;\quad \beta = \frac{p}{1-p}$
  $\mathbf{NB5}(\alpha,\, \beta) \to \mathbf{NB1}(r,\, p)\ :\ r = \alpha;\quad p = \frac{\beta}{1+\beta}$
- $\mathbf{NB2}(\lambda,\, \tau) \to \mathbf{NB3}(\mu,\, \phi)\ :\ \mu = \lambda;\quad \phi = 1/\tau$
  $\mathbf{NB3}(\mu,\, \phi) \to \mathbf{NB2}(\lambda,\, \tau)\ :\ \lambda = \mu;\quad \tau = 1/\phi$
- $\mathbf{NB2}(\lambda,\, \tau) \to \mathbf{NB4}(r,\, p)\ :\ r = 1/\tau\quad p = \frac{\tau\lambda}{1+\tau\lambda}$
  $\mathbf{NB4}(r,\, p) \to \mathbf{NB2}(\lambda,\, \tau)\ :\ \tau = 1/r;\quad \lambda = \frac{rp}{1-p}$
- $\mathbf{NB2}(\lambda,\, \tau) \to \mathbf{NB5}(\alpha,\, \beta)\ :\ \alpha = 1/\tau;\quad \beta = 1/(\tau\lambda)$
  $\mathbf{NB5}(\alpha,\, \beta) \to \mathbf{NB2}(\lambda,\, \tau)\ :\ \lambda = \alpha/\beta;\quad \tau = 1/\alpha$
- $\mathbf{NB3}(\mu,\, \phi) \to \mathbf{NB4}(r,\, p)\ :\ r = \phi;\quad p = \mu/(\phi+\mu)$
  $\mathbf{NB4}(r,\, p) \to \mathbf{NB3}(\mu,\, \phi)\ :\ \phi = r;\quad \mu = \frac{rp}{1-p}$
- $\mathbf{NB3}(\mu,\, \phi) \to \mathbf{NB5}(\alpha,\, \beta)\ :\ \alpha = \phi;\quad \beta = \phi/\mu$
  $\mathbf{NB5}(\alpha,\, \beta) \to \mathbf{NB3}(\mu,\, \phi)\ :\ \mu = \alpha/\beta;\quad \phi = \alpha$
- $\mathbf{NB4}(r,\, p) \to \mathbf{NB5}(\alpha,\, \beta)\ :\ \alpha = r;\quad \beta = (1-p)/p$
  $\mathbf{NB5}(\alpha,\, \beta) \to \mathbf{NB4}(r,\, p)\ :\ r = \alpha;\quad p = 1/(1+\beta)$

2.2 Normal distribution

Available parameterisations are

- Normal1(μ, σ) with μ – *mean* and σ – *standard deviation*,

$$P(x; \boldsymbol{\mu}, \boldsymbol{\sigma}) = \frac{1}{\sigma\sqrt{2\pi}} \exp\Big[-\frac{(x-\mu)^2}{2\sigma^2}\Big]$$

- Normal2(μ, v) with μ – *mean* and v – *variance*,

$$P(x; \boldsymbol{\mu}, \boldsymbol{v}) = \frac{1}{\sqrt{v}\sqrt{2\pi}} \exp\Big[-\frac{(x-\mu)^2}{2v}\Big]$$

- Normal3(μ, τ) with μ – *mean* and τ – *precision* ($\tau = 1/\sigma^2$)

$$P(x; \boldsymbol{\mu}, \boldsymbol{\tau}) = \sqrt{\frac{\tau}{2\pi}}\Big[-\frac{\tau}{2}(x-\mu)^2\Big].$$

2.2.1 Re-parameterisation formulas

In this case the formulas are very simple but are given here for the completeness

- $\mathbf{N1}(\mu,\, \sigma) \to \mathbf{N2}(\mu,\, v)\ :\ \mu \to \mu;\quad v = \sigma^2$
  $\mathbf{N2}(\mu,\, v) \to \mathbf{N1}(\mu,\, \sigma)\ :\ \mu \to \mu;\quad \sigma = \sqrt{v}$
- $\mathbf{N1}(\mu,\, \sigma) \to \mathbf{N3}(\mu,\, \tau)\ :\ \mu \to \mu;\quad \tau = 1/\sigma^2$
  $\mathbf{N3}(\mu,\, \tau) \to \mathbf{N1}(\mu,\, \sigma)\ :\ \mu \to \mu;\quad \sigma = 1/\sqrt{\tau}$
- $\mathbf{N2}(\mu,\, v) \to \mathbf{N3}(\mu,\, \tau)\ :\ \mu \to \mu;\quad \tau = 1/v$
  $\mathbf{N3}(\mu,\, \tau) \to \mathbf{N2}(\mu,\, v)\ :\ \mu \to \mu;\quad v = 1/\tau.$

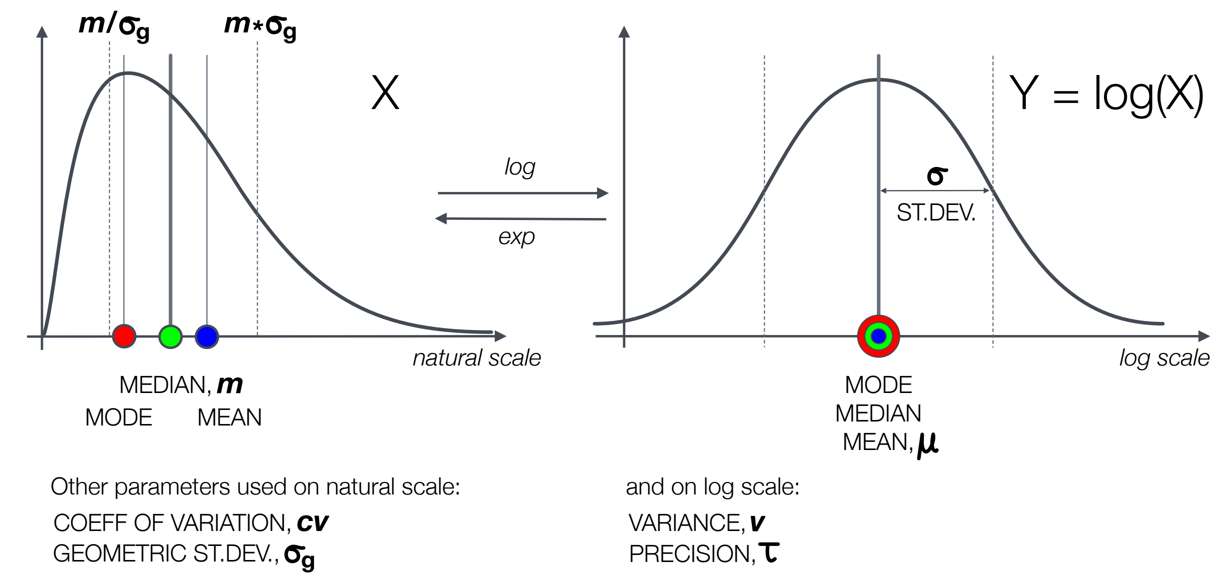

Figure 2.1: Schematic representation of the lognormaly distributed data on the natural (left) and logarithmic scale (right), see Figure 2.2 for real-life data example. **Bold** symbols stand for quantities commonly used to parameterise a log-normally distributed variable.

2.3 Log-normal distribution

The log-normal distribution is special in that not only different parameter sets exist but also because they are defined either on the natural or logarithmic scale. Interestingly, in one case the parameters are defined on two different scales, see Figure 2.1, for an overview.

Available parameterisations (also listed in Table A.1 with indication about their coverage in target tools) are

- LogNormal1(μ, σ) with *mean*, μ, and *standard deviation*, σ, both on the log-scale,

$$P(x; \boldsymbol{\mu}, \boldsymbol{\sigma}) = \frac{1}{x\sigma\sqrt{2\pi}} \exp\left[\frac{-(\log x - \mu)^2}{2\sigma^2}\right]$$

- LogNormal2(μ, v) with *mean*, μ, and *variance*, v, both on the log-scale,

$$P(x; \boldsymbol{\mu}, \boldsymbol{v}) = \frac{1}{x\sqrt{v}\sqrt{2\pi}} \exp\left[\frac{-(\log x - \mu)^2}{2v}\right]$$

- LogNormal3(m, σ) with *median*, m, on the natural scale and *standard deviation*, σ, on the log-scale,

$$P(x; \boldsymbol{m}, \boldsymbol{\sigma}) = \frac{1}{x\sigma\sqrt{2\pi}} \exp\left[\frac{-[\log(x/m)]^2}{2\sigma^2}\right]$$

- LogNormal4(m, cv) with *median*, m, and *coefficient of variation*, cv, both on the natural scale,

$$P(x; \boldsymbol{m}, \boldsymbol{cv}) = \frac{1}{x\sqrt{\log(cv^2+1)}\sqrt{2\pi}} \exp\left[\frac{-[\log(x/m)]^2}{2\log(cv^2+1)}\right]$$

- LogNormal5(μ, τ) with *mean*, μ, and *precision*, τ, both on the log-scale,

$$P(x; \boldsymbol{\mu}, \boldsymbol{\tau}) = \sqrt{\frac{\tau}{2\pi}}\frac{1}{x} \exp\left[-\frac{\tau}{2}(\log x - \mu)^2\right]$$

- LogNormal6(m, σ_g) with *median*, m, and *geometric st. deviation*, σ_g, both on the natural scale,

$$P(x; \boldsymbol{m}, \boldsymbol{\sigma_g}) = \frac{1}{x\log(\sigma_g)\sqrt{2\pi}} \exp\left[\frac{-[\log(x/m)]^2}{2\log^2(\sigma_g)}\right].$$

Figure 2.1 shows the schematic representation of lognormaly distributed data on the natural (left) and logarithmic scale (right), and indicates, on bold font, the available parameters characterising the distribution. On the other hand Figure 2.2 shows a real-life data example. We have used the basal insulin data in diabetic patients [Rudenski et al., 1991].

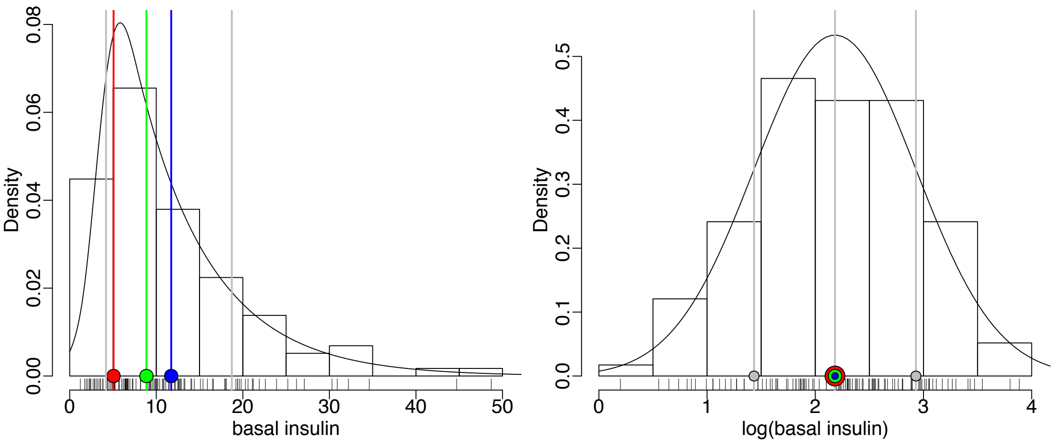

Figure 2.2: Representation of the lognormaly distributed basal insulin data in diabetic patients [Rudenski et al., 1991] on the natural scale (left) and on the logarithmic scale after *log*–transformation (right), colour code as in Figure 2.1. The density estimation for the data on the natural scale and its plotting was performed using the R package *logspline*, [Kooperberg, 2013]. The mode (red) on the natural scale doesn't tally with the maximum of the density curve as it should because it the latter is an approximation.

2.3.1 Re-parameterisation formulas

The recalculation between given parameterisations is error prone and should, whenever required, be taken over by the converters. The following equations might be useful when providing such translation support between target tools. For example when translating a model implemented for Monolix/NONMEM, which use either LN1 or LN2, with winBUGS as the target tool, which uses only LN5.

- LN1 relationships
  - $\mathbf{LN1}(\mu,\,\sigma) \to \mathbf{LN2}(\mu,\,v)\;:\;\mu \to \mu;\quad \sigma \to v = \sigma^2$
    $\mathbf{LN2}(\mu,\,v) \to \mathbf{LN1}(\mu,\,\sigma)\;:\;\mu \to \mu;\quad v \to \sigma = \sqrt{v}$
  - $\mathbf{LN1}(\mu,\,\sigma) \to \mathbf{LN3}(m,\,\sigma)\;:\;\mu \to m = \exp(\mu);\quad \sigma \to \sigma$
    $\mathbf{LN3}(m,\,\sigma) \to \mathbf{LN1}(\mu,\,\sigma)\;:\;m \to \mu = \log(m);\quad \sigma \to \sigma$
  - $\mathbf{LN1}(\mu,\,\sigma) \to \mathbf{LN4}(m,\,cv)\;:\;\mu \to m = \exp(\mu);\quad \sigma \to cv = \sqrt{\exp(\sigma^2)-1}$
    $\mathbf{LN4}(m,\,cv) \to \mathbf{LN1}(\mu,\,\sigma)\;:\;m \to \mu = \log(m);\quad cv \to \sigma = \sqrt{\log(cv^2+1)}$
  - $\mathbf{LN1}(\mu,\,\sigma) \to \mathbf{LN5}(\mu,\,\tau)\;:\;\mu \to \mu;\quad \sigma \to \tau = 1/\sigma^2$
    $\mathbf{LN5}(\mu,\,\tau) \to \mathbf{LN1}(\mu,\,\sigma)\;:\;\mu \to \mu;\quad \tau \to \sigma = 1/\sqrt{\tau}$
  - $\mathbf{LN1}(\mu,\,\sigma) \to \mathbf{LN6}(m,\,\sigma_g)\;:\;\mu \to m = \exp(\mu);\quad \sigma \to \sigma_g = \exp(\sigma)$
    $\mathbf{LN6}(m,\,\sigma_g) \to \mathbf{LN1}(\mu,\,\sigma)\;:\;m \to \mu = \log(m);\quad \sigma_g \to \sigma = \log(\sigma_g)$
- remaining LN2 relationships
  - $\mathbf{LN2}(\mu,\,v) \to \mathbf{LN3}(m,\,\sigma)\;:\;\mu \to m = \exp(\mu);\quad v \to \sigma = \sqrt{v}$
    $\mathbf{LN3}(m,\,\sigma) \to \mathbf{LN2}(\mu,\,v)\;:\;m \to \mu = \log(m);\quad \sigma \to v = \sigma^2$
  - $\mathbf{LN2}(\mu,\,v) \to \mathbf{LN4}(m,\,cv)\;:\;\mu \to m = \exp(\mu);\quad v \to cv = \sqrt{\exp(v)-1}$
    $\mathbf{LN4}(m,\,cv) \to \mathbf{LN2}(\mu,\,v)\;:\;m \to \mu = \log(m);\quad cv \to v = \log(cv^2+1)$
  - $\mathbf{LN2}(\mu,\,v) \to \mathbf{LN5}(\mu,\,\tau)\;:\;\mu \to \mu;\quad v \to \tau = 1/v$
    $\mathbf{LN5}(\mu,\,\tau) \to \mathbf{LN2}(\mu,\,v)\;:\;\mu \to \mu;\quad \tau \to v = 1/\tau$
  - $\mathbf{LN2}(\mu,\,v) \to \mathbf{LN6}(m,\,\sigma_g)\;:\;\mu \to m = \exp(\mu);\quad v \to \sigma_g = \exp(\sqrt{v})$
    $\mathbf{LN6}(m,\,\sigma_g) \to \mathbf{LN2}(\mu,\,v)\;:\;m \to \mu = \log(m);\quad \sigma_g \to v = \log(\sigma_g^2)$
- remaining LN3 relationships
  - $\mathbf{LN3}(m,\,\sigma) \to \mathbf{LN4}(m,\,cv)\;:\;m \to m;\quad \sigma \to cv = \sqrt{\exp(\sigma^2)-1}$
    $\mathbf{LN4}(m,\,cv) \to \mathbf{LN3}(m,\,\sigma)\;:\;m \to m;\quad cv \to \sigma = \sqrt{\log(cv^2+1)}$
  - $\mathbf{LN3}(m,\,\sigma) \to \mathbf{LN5}(\mu,\,\tau)\;:\;m \to \mu = \log(m);\quad \sigma \to \tau = 1/\sigma^2$
    $\mathbf{LN5}(\mu,\,\tau) \to \mathbf{LN3}(m,\,\sigma)\;:\;\mu \to m = \exp(\mu);\quad \tau \to \sigma = 1/\sqrt{\tau}$

– $\mathbf{LN3}(m,\,\sigma)\;\rightarrow\;\mathbf{LN6}(m,\,\sigma_g)\;:\;m \rightarrow m;\quad \sigma \rightarrow \sigma_g = \exp(\sigma)$
$\mathbf{LN6}(m,\,\sigma_g)\;\rightarrow\;\mathbf{LN3}(m,\,\sigma)\;:\;m \rightarrow m;\quad \sigma_g \rightarrow \sigma = \log(\sigma_g)$

- remaining LN4 relationships
  – $\mathbf{LN4}(m,\,cv)\;\rightarrow\;\mathbf{LN5}(\mu,\,\tau)\;:\;m \rightarrow \mu = \log(m);\quad cv \rightarrow \tau = 1/\log(cv^2+1)$
  $\mathbf{LN5}(\mu,\,\tau)\;\rightarrow\;\mathbf{LN4}(m,\,cv)\;:\;\mu \rightarrow m = \exp(\mu);\quad \tau \rightarrow cv = \sqrt{\exp(1/\tau)-1}$
  – $\mathbf{LN4}(m,\,cv)\;\rightarrow\;\mathbf{LN6}(m,\,\sigma_g)\;:\;m \rightarrow m;\quad cv \rightarrow \sigma_g = \exp\big(\sqrt{\log(cv^2+1)}\big)$
  $\mathbf{LN6}(m,\,\sigma_g)\;\rightarrow\;\mathbf{LN4}(m,\,cv)\;:\;m \rightarrow m;\quad \sigma_g \rightarrow cv = \sqrt{\exp\big(\log^2(\sigma_g)\big)-1}$
- remaining LN5 relationship
  – $\mathbf{LN5}(\mu,\,\tau)\;\rightarrow\;\mathbf{LN6}(m,\,\sigma_g)\;:\;\mu \rightarrow m = \exp(\mu);\quad \tau \rightarrow \sigma_g = \exp(1/\sqrt{\tau})$
  $\mathbf{LN6}(m,\,\sigma_g)\;\rightarrow\;\mathbf{LN5}(\mu,\,\tau)\;:\;m \rightarrow \mu = \log(m);\quad \sigma_g \rightarrow \tau = 1/\log^2(\sigma_g)$

The proof of the majority of the formulas is straightforward taking into account the definition of the parameters in question. The relationship between σ or τ (on the log scale) and cv (on the natural scale), essential for the re-calculation formulas involving LN4 parameterisation, is a bit more tricky to see. The proof starts with the known relationships for the mean, $mean$, and variance, var, on the natural scale. Then the square of the coefficient of variation, cv, on the natural scale reads

$$cv^2 = \frac{var}{mean^2} = \frac{(e^{\sigma^2}-1)\,e^{2\mu+\sigma^2}}{[e^{(\mu+1/2\sigma^2)}]^2} = (e^{\sigma^2}-1) \Leftrightarrow cv = \sqrt{e^{\sigma^2}-1} \quad \& \quad \sigma = \sqrt{\log(cv^2+1)}.$$

To see then the relationships between cv and σ_g on the natural scale one has to use the formula $\sigma_g = exp(\sigma)$ and we get the expected results

$$cv = \sqrt{\exp\big(\log^2(\sigma_g)\big)-1} \quad \& \quad \sigma_g = \exp\big(\sqrt{\log(cv^2+1)}\big).$$

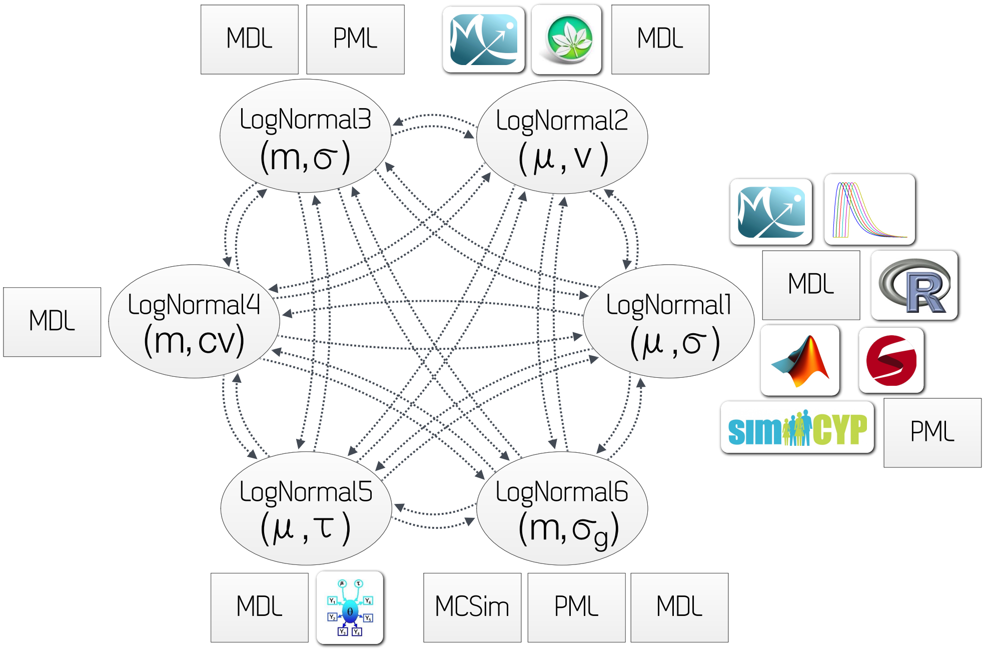

Figure 2.3: Re-parameterisation relationships implemented in ProbOnto and their support in selected target tools and other languages/tools. Note, that PopED features yet another parameterisation – to be supported soon in ProbOnto.

Chapter 3

Use Examples

3.1 Count data models

3.1.1 Zero-inflated Poisson – ZIP

ProbOnto simplifies the encoding of many discrete models significantly. In UncertML unavailable distributions such as Generalized Poisson (GP), Zero-inflated Poisson (ZIP) and other models frequently used in pharmacometrics, see for example [Plan et al., 2009] and [Trocóniz et al., 2009], are now available and very easy to encode. This example illustrates that.

The essential elements of the model is the PMF, here provided in the log-transformed form,

$$\begin{cases} \log(P(Y=k)) = \log(1-p0) - \lambda + k\log(\lambda) - \mathrm{factln}(k) & \text{if } k > 0 \\ \log(P(Y=k)) = \log(p0 + (1-p0)\exp(-\lambda)) & \text{otherwise} \end{cases}$$

and the definition of model parameters: the Poisson intensity, λ, and the probability of extra zeros, $p0$. In the case of explicitly encoded PMF the model becomes lengthly. This comes always with a risk of encoding bugs/typos.

Explicitly encoded PMF The complex conditional definition of this model within the <PMF> element reads

```
<mdef:ObservationModel blkId="om1">
    <mdef:Discrete>
        <mdef:CountData>
            <mdef:CountVariable symbId="y"/>
            <mdef:NumberCounts symbId="k"/>

            <mdef:PMF transform="log">
                <ct:Assign>
                    <math:Piecewise>
                        <!-- !!! 50 lines of code skipped here !!! -->
                        <!-- for encoding of the conditional PMF: -->
                        <!-- if (k > 0): log(1-p0)-lambda+k*log(lambda)-factln(k) -->
                        <!-- else: log(p0+(1-p0)*exp(-lambda)) -->
                    </math:Piecewise>
                </ct:Assign>
            </mdef:PMF>
        </mdef:CountData>
    </mdef:Discrete>
</mdef:ObservationModel>
```

Note also that the explicit encoding of PMF's can be used if the modeller wishes to encode a model not featured in ProbOnto. It therefore remains as an option in PharmML for any user-defined or other distribution.

Model encoded using ProbOnto In contrast to the first option, encoding of models supported by ProbOnto becomes very easy as only the code names for the distribution and its parameters need to be specified as the following code shows

```
<ObservationModel blkId="om1A">
    <Discrete>
        <CountData>
            <CountVariable symbId="y"/>
```

```
            <PMF transform="log">
                <Distribution>
                    <po:ProbOnto name="ZeroInflatedPoisson1">
                        <po:Parameter name="rate">
                            <ct:Assign>
                                <ct:SymbRef blkIdRef="pm1" symbIdRef="Lambda"/>
                            </ct:Assign>
                        </po:Parameter>
                        <po:Parameter name="probabilityOfZero">
                            <ct:Assign>
                                <ct:SymbRef blkIdRef="pm1" symbIdRef="P0"/>
                            </ct:Assign>
                        </po:Parameter>
                    </po:ProbOnto>
                </Distribution>
            </PMF>
        </CountData>
    </Discrete>
</ObservationModel>
```

3.1.2 Poisson with mixtures – PMIX

Rather then creating specific model types for e.g. mixture of Poisson models, we use a generic mixture model. For example for the mixture of two Poisson models

$$P(y_{ij} = k; \pi, \lambda_1, \lambda_2) \quad = \quad \pi \frac{e^{-\lambda_1}\lambda_1^k}{k!} + (1-\pi)\frac{e^{-\lambda_2}\lambda_2^k}{k!}$$

with $\lambda_1, \lambda_2 > 0$ and $\pi \in [0, 1]$, the *MixtureDistribution* can be used as the following code shows

```
<PMF>
    <Distribution>
        <po:ProbOnto name="MixtureDistribution">
            <!-- mixing weight -->
            <po:Parameter name="weight">
                <ct:Assign>
                    <ct:SymbRef symbIdRef="pi1"/>
                </ct:Assign>
            </po:Parameter>
            <!-- lambda1 - Poisson intensity -->
            <po:MixtureComponent name="Poisson1">
                <po:Parameter name="rate">
                    <ct:Assign>
                        <ct:SymbRef symbIdRef="lambda1"/>
                    </ct:Assign>
                </po:Parameter>
            </po:MixtureComponent>
            <!-- lambda2 - Poisson intensity -->
            <po:MixtureComponent name="Poisson1">
                <po:Parameter name="rate">
                    <ct:Assign>
                        <ct:SymbRef symbIdRef="lambda2"/>
                    </ct:Assign>
                </po:Parameter>
            </po:MixtureComponent>
        </po:ProbOnto>
    </Distribution>
</PMF>
```

The <MixtureComponent> elements hold the mixtures in question, here Poisson with λ_1 and Poisson λ_2, and <Parameter>, π, the mixture probability or *weight*. The solution has the advantage to be extendable to any number of mixing components, m, [Forbes et al., 2011]. The parameter π becomes a vector, π_i with $\pi_i \in [0, 1], i = 1, \ldots, m$, and can be encoded as such using the <Vector> element.

Discussion The use of a generic *MixtureDistribution* seems well justified in this case. However, because reference literature exists for general Mixed Poisson regression models, [Wang et al., 1996], the introduction of such specialised mixture distribution could be considered for ProbOnto in future as well.

3.1.3 Other count data models

Growing number of count data related distributions means better coverage of this important type of models, see for example [Paule et al., 2012] and [Plan, 2014]. The models supported so far include

- Conway-Maxwell-Poisson (λ, ν)
- Double Poisson (μ, ϕ)
- Generalized Negative Binomial (θ, β, m)
- Generalized Poisson
  - Generalized Poisson 1 (θ, δ)
  - Generalized Poisson 2 (μ, δ)
  - Generalized Poisson 3 (μ, α)
- Inverse Binomial (k, p)
- Negative Binomial
  - Negative Binomial 1$(r,\ p)$
  - Negative Binomial 2 (λ, τ)
  - Negative Binomial 3 (μ, k)
  - Negative Binomial 4 (r, p)
  - Negative Binomial 5 (α, β)
- Poisson (λ)
- Zero-Inflated Negative Binomial $(\lambda, \tau, p0)$
- Zero-Inflated Generalized Poisson $(\mu, \alpha, p0)$
- Zero-Inflated Poisson (λ, π)

As can be seen from the listing, two models come with multiple parameterisations. This aspect is described in detail in section 2. In short, their presence, with where available re-parameterisation formulas, allows for

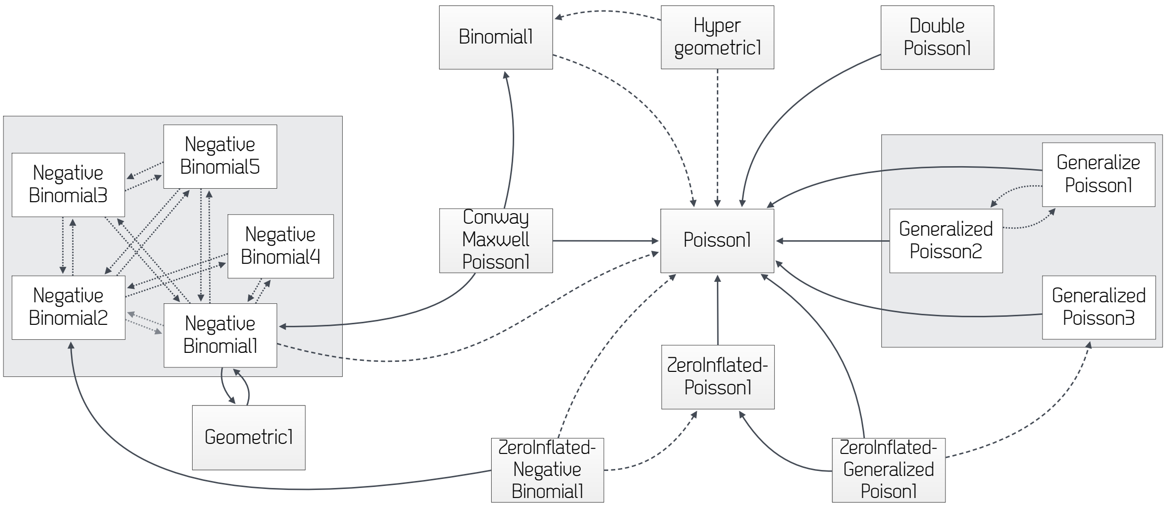

Figure 3.1: Count data models and the relationships between models stored in ProbOnto which are a subset of the network shown in Figure 1.4.

accounting for and dealing with varying support in target tools. The re-parameterisation formulas will be supported by the libPharmML making model translation to the target tool an easy task. Their relationships are visualised in Figure 3.1 which are a subset of the network shown in Figure 1.4.

3.2 Categorical data models

For categorical models we always first list in PharmML all categories, see below element `<ListOfCategories>`... `</ListOfCategories>` which informs the user/target tool about the number and identifiers of the categories in question. The probabilities vector $p_i, i = 1, \ldots, k$ is the only parameter and the user has two options. One can declare either

- explicitly the probabilities for all k categories or
- the $k-1$ probabilities, with the last probability, p_k, known assuming $\Sigma_i p_i = 1$.

Note that the order of the categories in `<ListOfCategories>` and the `<Parameter>` (where the probability vector, p_i, is encoded) elements, must be preserved.

3.2.1 Nominal categorical model

Consider the following *Observation model* for nominal categorical data, [Swat et al., 2014]:

- Type of observed variable – discrete/categorical
- Category variable: y
- Set of categories: $\{1, 2, 3\}$
- Probabilities for category '1' and '2'

$$p1 := P(y = 1) = a1/(a1 + a2 + a3)$$
$$p2 := P(y = 2) = a2/(a1 + a2 + a3)$$

The following code shows how to implement the model starting with the general information: parameters involved and equations for the probabilities

```
<ObservationModel blkId="om1">

    <Discrete>
        <CategoricalData ordered="no">
            <!-- can alternatively be defined as individual parameters with IIV etc.-->
            <PopulationParameter symbId="a1"/>
            <PopulationParameter symbId="a2"/>
            <PopulationParameter symbId="a3"/>

            <PopulationParameter symbId="p1">
                <ct:Assign>
                    <!-- omitted formula p1 = a1/(a1+a2+a3) -->
                </ct:Assign>
            </PopulationParameter>
            <PopulationParameter symbId="p2">
                <ct:Assign>
                    <!-- omitted formula p2 = a2/(a1+a2+a3) -->
                </ct:Assign>
            </PopulationParameter>
```

then listing the categories and specifying the category variable

```
            <ListOfCategories>
                <Category symbId="cat1"/>
                <Category symbId="cat2"/>
                <Category symbId="cat3"/>
            </ListOfCategories>
            <CategoryVariable symbId="y"/>
```

and eventually defining the unordered categorical distribution, *CategoricalNonordered* in ProbOnto, with the parameter

- `categoryProb` – event probabilities vector, $p_1, \ldots, p_k$

with the number of categories, here equal $k=3$, which can be inferred from the length of the `<ListOfCategories>`. The PMF reads then

```
            <PMF>
                <Distribution>
                    <po:ProbOnto name="CategoricalNonordered1">
                        <!-- category probabilities - a vector of length 2 (=k-1) -->
                        <po:Parameter name="categoryProb">
                            <ct:Assign>
                                <ct:Vector>
                                    <ct:VectorElements>
                                        <ct:SymbRef symbIdRef="p1"/>
                                        <ct:SymbRef symbIdRef="p2"/>
                                    </ct:VectorElements>
                                </ct:Vector>
                            </ct:Assign>
                        </po:Parameter>
                    </po:ProbOnto>
                </Distribution>
            </PMF>
        </CategoricalData>
    </Discrete>
</ObservationModel>
```

Given that there are k categories, by default the specification of $k-1$ probabilities is sufficient assuming $\Sigma p_i = 1$.

3.3 Truncated distributions

Truncation bounds can be set using <LowerTruncationBound> and <UpperTruncationBound> elements which accept any expression, such as $X \sim \mathcal{N}(\mu, \sigma, lower = \mu - 1.96\,\sigma, upper = \mu + 1.96\,\sigma)$ which in PharmML reads

```
<IndividualParameter symbId="pTruncated">
    <Distribution>
        <po:ProbOnto name="Normal1">
            <po:Parameter name="mean">
                <ct:Assign>
                    <ct:SymbRef symbIdRef="mu"/>
                </ct:Assign>
            </po:Parameter>
            <po:Parameter name="stdev">
                <ct:Assign>
                    <ct:SymbRef symbIdRef="sigma"/>
                </ct:Assign>
            </po:Parameter>
            <po:LowerTruncationBound>
                <ct:Assign>
                    <math:Binop op="minus">
                        <ct:SymbRef symbIdRef="mu"/>
                        <math:Binop op="times">
                            <ct:Real>1.96</ct:Real>
                            <ct:SymbRef symbIdRef="sigma"/>
                        </math:Binop>
                    </math:Binop>
                </ct:Assign>
            </po:LowerTruncationBound>
            <po:UpperTruncationBound>
                <ct:Assign>
                    <math:Binop op="plus">
                        <ct:SymbRef symbIdRef="mu"/>
                        <math:Binop op="times">
                            <ct:Real>1.96</ct:Real>
                            <ct:SymbRef symbIdRef="sigma"/>
                        </math:Binop>
                    </math:Binop>
                </ct:Assign>
            </po:UpperTruncationBound>
        </po:ProbOnto>
    </Distribution>
</IndividualParameter>
```

The following Figure 3.2 illustrates few examples of truncated normal distribution on the interval [-2.5,1]

Note, that the normal distribution has been selected for demonstration purposes how to use the truncation bounds. ProbOnto features in its collection the $TruncatedNormal1(\mu, \sigma, a, b)$ distribution, which could be used instead.

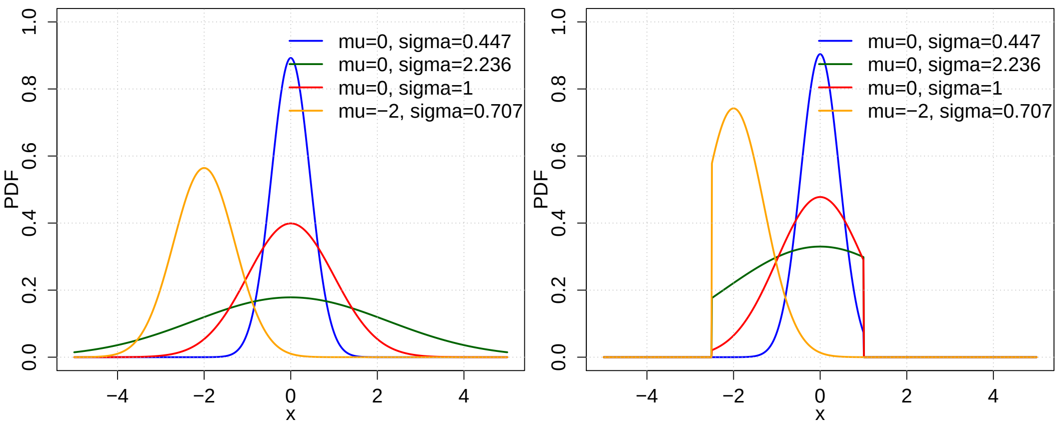

Figure 3.2: Truncated normal distribution, N1. The truncated density plots on the right hand side were performed using the *truncnorm* function of the *dtruncnorm* R-package.

3.4 Complete PharmML model

At last, we provide a complete PharmML coded model – the Poisson model for count data defined as follows

- Type of observed variable – discrete/count
- Category variable: Y
- PMF

$$P(y_{ij} = k; \lambda) \quad = \quad \frac{e^{-\lambda}\lambda^k}{k!}$$

Its implementation reads

```
<?xml version="1.0" encoding="UTF-8"?>
<PharmML xmlns:xsi="http://www.w3.org/2001/XMLSchema-instance"
   xmlns="http://www.pharmml.org/pharmml/0.8/PharmML"
   xsi:schemaLocation="http://www.pharmml.org/pharmml/0.8/PharmML
   http://www.pharmml.org/pharmml/0.8/PharmML"
   xmlns:ct="http://www.pharmml.org/pharmml/0.8/CommonTypes"
   xmlns:po="http://www.pharmml.org/probonto/ProbOnto"
   xmlns:mdef="http://www.pharmml.org/pharmml/0.8/ModelDefinition"
   implementedBy="MJS" writtenVersion="0.8" id="i1">

   <ct:Name>Poisson_model</ct:Name>

   <mdef:ModelDefinition>
      <mdef:ParameterModel blkId="pm1">
         <mdef:Parameter symbId="lambda"/>
      </mdef:ParameterModel>
      <mdef:ObservationModel blkId="om1">
         <mdef:Discrete>
            <mdef:CountData>
               <mdef:CountVariable symbId="Y"/>
               <mdef:PMF>
                  <mdef:Distribution>
                     <po:ProbOnto name="Poisson1">
                        <po:Parameter name="rate">
                           <ct:Assign>
                              <ct:SymbRef blkIdRef="pm1" symbIdRef="lambda"/>
                           </ct:Assign>
                        </po:Parameter>
                     </po:ProbOnto>
                  </mdef:Distribution>
               </mdef:PMF>
            </mdef:CountData>
         </mdef:Discrete>
      </mdef:ObservationModel>
   </mdef:ModelDefinition>
</PharmML>
```

In PharmML, such model can be extended by a declaration of an explicit or dataset sourced trail design and modelling steps, such as simulation, estimation or optimal trial design – for brevity these elements have been omitted. For full examples visit `ddmore.eu` or `pharmml.org`.

Appendix A

Distributions in ProbOnto

This document contains overview tables of the supported univariate and multivariate distributions, the tool coverage and the code names.

All DDMoRe target tools distributions are covered, i.e. those used in (Monolix [Lixoft Team, 2014], NONMEM [Beal et al., 2009] and winBUGS [Spiegelhalter et al., 2003]), a number of distributions featured in STAN, [STAN Development Team, 2015b], and many others useful in pharmacometrics in beyond (Matlab Statistical Toolbox and R).

A.1 Univariate distributions

A.1.1 Tool coverage

**ProbOnto** 1.1	Parame- -ters	**UncertML** 3.0	**BUGS** 1.4	**Monolix** 4.3	**NONMEM** 7.3	**STAN** 2.9.0
Discrete Univariate						
Bernoulli 1	p	y	y	–	–	y
Beta-Binomial	n, α, β	–	–	–	–	y
Binomial 1	n, p	y	y	–	–	y
Categorical ordered	$p_1, ..., p_k$	y	y	–	–	–
Categorical nonordered	$p_1, ..., p_k$	y	y	–	–	y
Conway-Maxwell-Poisson1	λ, ν	–	–	–	–	–
Double Poisson1	μ, ϕ	–	–	–	–	–
Generalized Negative Binomial	θ, β, m	–	–	–	–	–
Generalized Poisson 1	θ, δ	–	–	–	–	–
Generalized Poisson 2	μ, δ	–	–	–	–	–
Generalized Poisson 3	μ, α	–	–	–	–	–
Geometric	p	y	–	–	–	–
Hypergeometric	N, K, n	y	–	–	–	y
Inverse Binomial	k, p	–	–	–	–	–
Negative Binomial 1	r, p	–	y	–	–	–
Negative Binomial 2	λ, τ	–	–	–	–	–
Negative Binomial 3	μ, k	–	–	–	–	y
Negative Binomial 4	r, p	y	–	–	–	–
Negative Binomial 5	α, β	–	–	–	–	y
Poisson	λ	y	y	–	–	y
Uniform Discrete 1	a, b, n	–	–	–	–	–
Uniform Discrete 2	$a = 0, n$	–	–	–	–	–
Zero-Inflated Negative Binomial	$\lambda, \tau, p0$	–	–	–	–	–
Zero-Inflated Generalized Poisson	$\mu, \alpha, p0$	–	–	–	–	–
Zero-Inflated Poisson	λ, π	–	–	–	–	–
Continuous Univariate						
Beta	α, β	y	y	y	–	y
Birnbaum-Saunders	n, α, γ	–	–	–	–	–
Cauchy	x_0, γ	y	–	–	–	y
Chi-Squared	k	y	y	y	–	y
Erlang	b, c	–	–	–	–	–
Exponential 1	λ	y	y	y	–	y
Exponential 2	β	–	–	–	–	–
F (aka Fisher-Snedecor)	n_1, n_2	y	–	y	–	–
Gamma 1	k, θ	y	–	y	–	–
Gamma 2	r, μ	–	y	–	–	y
Generalized Gamma 1	$a,\ d,\ p$	–	–	–	–	–
Generalized Gamma 2	$a,\ b,\ c,\ k$	–	–	–	–	–
Generalized Gamma 3	$a,\ d,\ p$	–	y	–	–	–
Gompertz	η, b	–	–	y	–	–
Gumbel (aka Extreme Value)	μ, β	–	–	y	–	y
Half-Normal	θ	–	–	–	–	–
Inverse-Gamma	α, β	y	–	–	–	y
Inverse-Gaussian (aka Wald)	λ, μ	–	–	–	–	–
Laplace 1 (aka Double-exponential)	μ, b	y	–	–	–	y
Laplace 2	μ, τ	–	y	–	–	–
Logistic 1	μ, s	y	–	–	–	y

Logistic 2	μ, τ	–	y	–	–	–
Log-Logistic 1 (aka Fisk)	α, β	y	–	–	–	–
Log-Logistic 2	λ, κ	–	–	–	–	–
Log-Normal 1	μ, σ	y	–	y	–	y
Log-Normal 2	$\mu,\ v$	y	–	y	–	–
Log-Normal 3	$m,\ \sigma$	–	–	–	–	–
Log-Normal 4	$m,\ cv$	–	–	–	–	–
Log-Normal 5	$\mu,\ \tau$	–	y	–	–	–
Log-Normal 6	$m,\ \sigma_g$	–	–	–	–	–
Log-Uniform	$min,\ max$	–	–	–	–	–
Nakagami	m, Ω	–	–	–	–	–
Normal 1	μ, σ	y	–	y	y	y
Normal 2	μ, v	y	–	y	–	–
Normal 3	μ, τ	–	y	–	–	–
Normal-inverse-gamma	$\mu, \lambda, \alpha, \beta$	y	–	–	–	–
Pareto	x_m, α	y	y	–	–	y
Rayleigh	σ	–	–	y	–	y
Standard Normal	$\mu=0, \sigma=1$	y	–	y	y	–
Standard Uniform	$a=0, b=1$	–	–	y	y	–
Student's T 1	ν	y	–	y	–	–
Student's T 2	μ, τ, k	–	y	–	–	–
Triangular	a, b, c	–	–	–	–	–
Truncated Normal	μ, σ, a, b	–	–	–	–	–
Uniform	$a,\ b$	y	y	y	–	y
Weibull 1	λ, k	y	–	y	–	y
Weibull 2	λ, v	–	y	–	–	–

Table A.1: Univariate distributions supported in ProbOnto with overview of tool support.

A.1.2 Code names

**Distribution**	**Parameters**			
Code name	Symbol	Code name	Symbol	Code name
		Discrete Univariate		
`Bernoulli1`	p	`probability`	–	–
`BetaBinomial1`	n	`numberOfTrials`	α	`alpha`
			β	`beta`
`Binomial1`	n	`numberOfTrials`	p	`probability`
`CategoricalOrdered1`	$p_1, \ldots, p_k$	`categoryProb`	–	–
`CategoricalNonordered1`	$p_1, \ldots, p_k$	`categoryProb`	–	–
`ConwayMaxwellPoisson1`	λ	`rate`	ν	`rateOfDecay`
`DoublePoisson1`	μ	`rate`	ϕ	`dispersion`
`GeneralizedNegativeBinomial1`	θ	`theta`	β	`beta`
			m	`m`
`GeneralizedPoisson1`	θ	`rate`	δ	`dispersion`
`GeneralizedPoisson2`	μ	`mean`	δ	`dispersion`
`GeneralizedPoisson3`	μ	`mean`	α	`dispersion`
`Geometric1`	p	`probability`	–	–
`Hypergeometric1`	N	`populationSize`	K	`numberOfSuccesses`
			n	`numberOfTrials`
`InverseBinomial1`	k	`index`	p	`probability`
`NegativeBinomial1`	r	`numberOfSuccesses`	p	`probability`
`NegativeBinomial2`	λ	`rate`	τ	`overdispersion`
`NegativeBinomial3`	μ	`mean`	k	`dispersion`
`NegativeBinomial4`	r	`numberOfFailures`	p	`probability`

NegativeBinomial5	α	shape	β	inverseScale
Poisson1	λ	rate	–	–
UniformDiscrete1	a	minimum	b	maximum
			n	numberOfValues
UniformDiscrete2	$a = 0$	minimum	n	numberOfValues
ZeroInflatedNegativeBinomial1	λ	rate	τ	overdispersion
			$p0$	probabilityOfZero
ZeroInflatedGeneralizedPoisson1	μ	mean	α	dispersion
			$p0$	probabilityOfZero
ZeroInflatedPoisson1	λ	rate	π	probabilityOfZero
Continuous Univariate				
Beta1	α	alpha	β	beta
BirnbaumSaunders1	α	scale	γ	shape
Cauchy1	x_0	location	γ	scale
ChiSquared1	k	degreesOfFreedom	–	–
Erlang1	b	scale	c	shape
Exponential1	λ	rate	–	–
Exponential2	β	mean	–	–
F1	n_1	numerator	n_2	denominator
Gamma1	k	shape	θ	scale
Gamma2	r	shape	μ	rate
GeneralizedGamma1	a	scale	d	shape1
			k	shape2
GeneralizedGamma2	a	location	b	scale
	c	shape1	p	shape2
GeneralizedGamma3	r	scale	μ	shape1
			β	shape2
Gompertz1	η	shape	b	scale
Gumbel1	μ	location	β	scale
HalfNormal1	θ	mean	σ	stdev
InverseGamma1	α	shape	β	scale
InverseGaussian1	λ	shape	μ	mean
Laplace1	μ	location	b	scale
Laplace2	μ	location	τ	tau
Logistic1	μ	location	s	scale
Logistic2	μ	location	τ	inverseScale
LogLogistic1	α	scale	β	shape
LogLogistic2	λ	scale	κ	shape
LogNormal1	μ	meanLog	σ	stdevLog
LogNormal2	μ	meanLog	v	varLog
LogNormal3	m	median	σ	stdevLog
LogNormal4	m	median	cv	coefVar
LogNormal5	μ	meanLog	τ	precision
LogNormal6	m	median	σ_g	geomStdev
LogUniform1	min	minimum	max	maximum
Nakagami1	m	shape	Ω	spread
Normal1	μ	mean	σ	stdev
Normal2	μ	mean	v	var
Normal3	μ	mean	τ	precision
NormalInverseGamma1	μ	mean	λ	lambda
	α	alpha	β	beta
Pareto1	x_m	scale	α	shape
			α	tailIndex
Rayleigh1	σ	scale	–	–
StandardNormal1	$\mu{=}0$	mean	$\sigma{=}1$	stdev

StandardUniform1	$a=0$	minimum	$b=1$	maximum
StudentT1	ν	degreesOfFreedom	–	–
StudentT2	μ	mean	τ	scale
			ν	degreesOfFreedom
Triangular1	a	lowerLimit	b	upperLimit
			c	shape
TruncatedNormal1	μ	mean	σ	stdev
	a	lowerBound	b	upperBound
Uniform1	a	minimum	b	maximum
Weibull1	λ	scale	k	shape
Weibull2	λ	lambda	v	shape

Table A.2: Code names for distribution and parameter names of the univariate distributions. The use of the parameter code names for StandardNormal1 and StandardUniform1 is not mandatory.

A.2 Mixture distribution

**ProbOnto** 1.1	Parameters	**UncertML** 3.0	**WinBUGS** 1.4	**Monolix** 4.3	**NONMEM** 7.3	**STAN** 2.9.0
MixtureDistribution1	$\pi_1, \ldots, \pi_k$	y	–	–	–	–

Table A.3: Mixture distribution with overview of tool support.

**ProbOnto**	Parameters	
1.1	Symbol	Code name
MixtureDistribution1	$\pi_1, \ldots, \pi_k$	weight

Table A.4: Code names for mixture distribution – defined for univariate distributions only.

A.3 Multivariate distributions

A.3.1 Tool coverage

**ProbOnto**	Parameters	**UncertML**	**WinBUGS**	**Monolix**	**NONMEM**	**STAN**
1.1		3.0	1.4	4.3	7.3	2.9.0
Discrete Multivariate						
Multinomial	$n, p_1, \ldots, p_k$	y	y	–	–	y
Continuous Multivariate						
Dirichlet	$\alpha_1, \ldots, \alpha_K$	y	y	–	–	y
Inverse-Wishart	Ψ, ν	–	–	–	y	y
Multivariate Normal 1	μ, Σ	y	–	–	–	y
Multivariate Normal 2	μ, T	–	y	–	–	y
Multivariate (Student)-T1	μ, Σ, ν	y	–	–	–	y
Multivariate (Student)-T2	μ, T, k	–	y	–	–	–
Wishart 1	V, n	y	–	–	–	y
Wishart 2	R, k	–	y	–	–	–

Table A.5: Multivariate distributions with overview of tool support.

A.3.2 Code names

**Distribution**	**Parameters**			
Code name	Symbol	Code name	Symbol	Code name
Discrete Multivariate				
`Multinomial1`	n	`numberOfTrials`	$p_1, \ldots, p_k$	`probabilityOfSuccess`
Continuous Multivariate				
`Dirichlet1`	$\alpha_1, \ldots, \alpha_K$	`concentration`	–	–
`InverseWishart1`	Ψ	`scaleMatrix`	ν	`degreesOfFreedom`
`MultivariateNormal1`	μ	`mean`	Σ	`covarianceMatrix`
`MultivariateNormal2`	μ	`mean`	T	`precisionMatrix`
`MultivariateStudentT1`	μ	`mean`	Σ	`covarianceMatrix`
			ν	`degreesOfFreedom`
`MultivariateStudentT2`	μ	`mean`	T	`precisionMatrix`
			k	`degreesOfFreedom`
`Wishart1`	V	`scaleMatrix`	n	`degreesOfFreedom`
`Wishart2`	R	`inverseScaleMatrix`	k	`degreesOfFreedom`

Table A.6: Code names for the multivariate distributions and their parameters.

Appendix A

Parameterisations of the negative binomial (NB1 vs NB4)

There are at least three ways to formulate and interpret an experiment for the independent Bernoulli trials with a fixed number of outcomes (successes, failures or both) with the goal to estimate its probability distribution. The negative binomial (NB) model which formalises this experiment is formulated using two out of three following parameters dependent on the formulation:

- number of trials (i.e. total number successes or failures)
- number of successes
- number of failures

The possible formulations for such experiment are

Option 1 observing k failures before obtaining the rth success (most common)

Option 2 obtaining r successes until k failures have occurred

Option 3 number of trials (successes or failures), n, required before the rth success occurs.

The definition of the negative binomial in UncertML is based on the English Wikipedia, which features *Option 2*[1], a rather rare form of this distribution. This might lead to mistakes because tools such as Matlab, R and winBUGS, to mention here only few, use the most common formulation, *Option 1*, see Figure A.1 and Table A.1 capturing the differences.

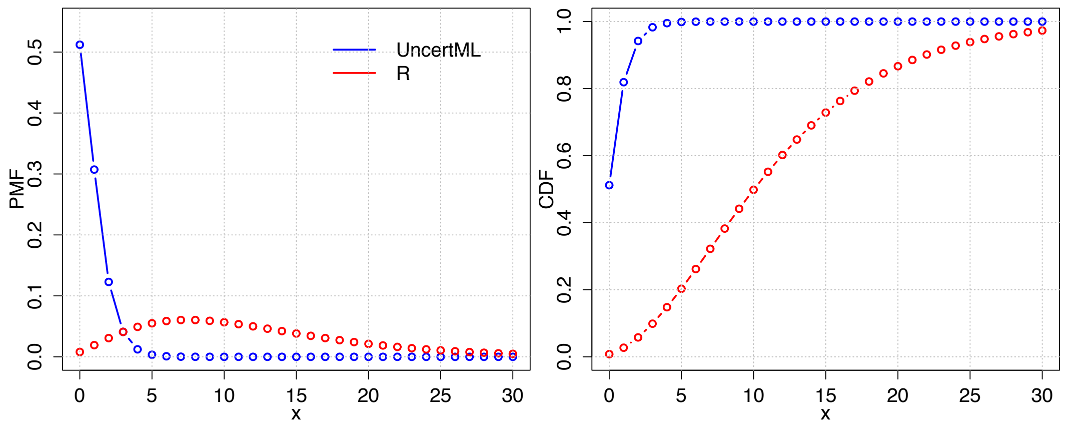

Figure A.1: Comparison of PMF and CDF plots for *Option 1* (supported in UncertML) and *Option 2* (supported in R). In blue the functions for the UncertML formulation, in red as implemented in R for parameters $r = n = 3$ and $p = 0.2$. Using the formulas given in Table A.1 one can calculate the means as 0.75 and 12, for *Option 1* and *Option 2*, respectively.

[1]This was not always the case as the change from the standard formulation to the current one happened around 2010. The majority of users who commented on this change are unhappy about it, see the English and French Wikipedia Talk/Discussion pages. Because there is no agreement to reverse these changes, which would require significant effort, the page remains as is.

	UncertML	R
PMF	$f(x;r,p)=\binom{x+r-1}{x}p^x(1-p)^r$	$f(x;n,p)=\binom{x+n-1}{x}p^n(1-p)^x$
Support	$x \in 0,1,2,...$ number of **successes**	$x \in 0,1,2,...$ number of **failures**
Parameters	$p \in [0,1]$ – probability of success<br>r – number of **failures**	$p \in (0,1]$ – probability of success<br>n – number of **successes**
Mean	$\frac{rp}{1-p}$	$\frac{n(1-p)}{p}$

Table A.1: UncertML versus R: comparison of the model formulation, parameter interpretation and the support variable. The PMF equations of these two models are very similar so that the differences can go unnoticed to most modellers which might in turn result in unwanted results, see figure A.1.

See Table A.2 for the overview of the occurrence of these options in the literature and software tools.

A.1 Differences explained

The above mentioned formulation options, see also table A.1, are better understood if one compares different language version for the articles on negative binomial distribution in [Wikipedia (English), 2015], [Wikipedia (French), 2015] and [Wikipedia (German), 2015].

English Wikipedia version (Option 1) – supported in UncertML

Interpretation: distribution of the number of successes, k, until r failures have occurred.

$$P_{NB}(k;r,p)=\binom{k+r-1}{k}p^k(1-p)^r, \quad \mathrm{E}(X=k)=\frac{rp}{(1-p)}$$

- Support
  - $k \in \{0,1,2,3,\dots\}$ – number of **successes**
- Parameters
  - $r > 0$ – number of **failures** until the experiment is stopped
  - $p \in (0,1)$ – success probability in each experiment

French Wikipedia version (Option 2)

Interpretation: distribution of the number of failures, k, before obtaining n successes

$$P_{NB}(k;n,p)=\binom{k+n-1}{k}p^n(1-p)^k, \quad \mathrm{E}(X=k)=\frac{n(1-p)}{p}$$

- Support
  - $k \in \{0,1,2,3,\dots\}$ – number of **failures**
- Parameters
  - $n > 0$ – number of **successes** until the experiment is stopped (fr: *le nombre de succès attendus*)
  - $p \in (0,1)$ – success probability in each experiment (fr: *la probabilitè d'un succès*)

German Wikipedia version (Option 2)

The German Wiki page describes two alternative representations and interpolations of this distribution. We present here that which is listed as the alternative representation. (The alternative is of type referred above as 'Option 3'.)
Interpretation: distribution of the number of failures, k, before obtaining r successes. (ger.: *NB Distribution beschreibt die Anzahl, k, der Misserfolge bis zum Eintreten des r-ten Erfolgs.*)

$$P_{NB}(k;r,p)=\binom{k+r-1}{k}p^r(1-p)^k, \quad \mathrm{E}(X=k)=\frac{r(1-p)}{p}$$

- Support
  - $k \in \{0, 1, 2, 3, \dots\}$ – number of **failures** (ger: *Anzahl Misserfolge*)
- Parameters
  - $r > 0$ – number of **successes** until the experiment is stopped (ger: *Anzahl Erfolge bis zum Abbruch*)
  - $p \in (0, 1)$ – success probability in each experiment, (ger: *Einzel-Erfolgs-Wahrscheinlichkeit*)

$$P_{NB}(k; r, p) = \binom{k+r-1}{k} p^r (1-p)^k.$$

A.2 Comparison of formulation support

The following table gives an overview of supported NB formulation options across over 30 literature sources and specialised software tools.

Source	PMF	Probability	Support	Variate
Option 1 *probability of observing a fixed number of failures before certain number of success*				
[Hilbe, 2011]	$\binom{y+r-1}{y} p^r (1-p)^{\mathbf{y}}$	$0 < p < 1$	$0 \le y < \infty$	#failures
[Forbes et al., 2011]	$\binom{x+r-1}{x} p^r (1-p)^{\mathbf{x}}$	$0 < p < 1$	$0 \le x < \infty$	#failures
[Leemis and Mcqueston, 2008]	$\binom{x+r-1}{x} p^r (1-p)^{\mathbf{x}}$	$0 < p < 1$	$0 \le x < \infty$	–
[Devroye, 1986]	$\binom{x+n-1}{x} p^n (1-p)^{\mathbf{x}}$	$p \in (0, 1)$	$x \ge 0$	#failures
[Dobson, 2002]	$\binom{y+r-1}{y} p^r (1-p)^{\mathbf{y}}$	–	y	–
[Hardin et al., 2007]	$\binom{y+r-1}{r-1} p^r (1-p)^{\mathbf{y}}$	$0 < p < 1$	$y = 0, 1, 2, ...$	#failures
[Bolker, 2008]	$\frac{(n+x-1)!}{(n-1)!\,x!} p^n (1-p)^{\mathbf{x}}$	$0 < p < 1$	$x \ge 0$	#failures
[Scheaffer and Young, 2009]	$\binom{x+r-1}{r-1} p^r (1-p)^{\mathbf{x}}$	–	$x = 0, 1, ...$	#failures
[Bonate, 2011]	$\frac{\Gamma(y+k)}{\Gamma(k) y!} p^k (1-p)^{\mathbf{y}}$	–	$y \ge 0$	–
[Yu et al., 2013]	$\binom{r+x-1}{x} p^r (1-p)^{\mathbf{x}}$	$p \in (0, 1)$	$x \ge 0$	#failures
[Dodge, 2008]	$C^x_{z+x-1} p^r (1-p)^{\mathbf{k}}$	–	k	#failures
[Lesaffre and Lawson, 2012]	$\binom{\theta+n-1}{\theta} \pi^n (1-\pi)^{\theta}$	$0 \le p \le 1$	$\theta \in \{0, 1, 2, ..., n\}$	#failures
[Law, 2007]	$\binom{x+s-1}{x} p^s (1-p)^{\mathbf{x}}$	$p \in (0, 1)$	$x \in \{0, 1, 2, ...\}$	#failures
[Vidakovic, 2011]	$\binom{r+x-1}{x} p^r (1-p)^{\mathbf{x}}$	–	$x = 0, 1, 2, ...$	#failures
[Cook, 2009]	$\binom{r+x-1}{x} p^r (1-p)^{\mathbf{x}}$	$0 < p < 1$	$x \ge 0$	#failures
Matlab, Stats Toolbox	$\binom{x+r-1}{x} p^r (1-p)^{\mathbf{x}}$	$0 < p < 1$	$0 \le x < \infty$	#failures
[R Core Team, 2013]	$\frac{\Gamma(x+n)}{\Gamma(n) x!} p^n (1-p)^{\mathbf{x}}$	$0 < p \le 1$	$x \in 0, 1, ...$	#failures
[Yee, 2008, Yee, 2015]	$\binom{y+k-1}{y} p^k (1-p)^{\mathbf{y}}$	$0 < p < 1$	$y \in 0, 1, 2, ...$	–
[SAS Institute Inc., 2014]	$\frac{(n+x-1)!}{x!\,(n-1)!} p^n (1-p)^{\mathbf{x}}$	$p \in (0, 1)$	$x \in \{0, 1, ...\}$	–
[Lunn et al., 2002]	$\binom{x+r-1}{x} p^r (1-p)^{\mathbf{x}}$	–	$0 \le x < \infty$	#failures[2]
[Plummer, 2003]	$\binom{x+r-1}{x} p^r (1-p)^{\mathbf{x}}$	$0 < p \le 1$	$x \ge 0$	–
[Marichev and Trott, 2013]	$\binom{m+n-1}{n-1} p^n (1-p)^{\mathbf{m}}$	$0 < p \le 1$	$m \in 0, 1, 2, ...$	–
VoseSoftware.com	$\binom{s+x-1}{x} p^s (1-p)^{\mathbf{x}}$	$0 < p \le 1$	$x \in 0, 1, ...$	#failures
boost.org	$\binom{x+r-1}{x} p^r (1-p)^{\mathbf{x}}$	$0 \le p \le 1$	x	#failures

[2] Interpretation based on the description of winBUGS in [Vidakovic, 2011].

Mathwave.com	$\binom{x+n-1}{x}p^n(1-p)^{\mathbf{x}}$	$0 < p < 1$	$x \in 0, 1, ...$	–
Wolfram MathWorld	$\binom{x+r-1}{r-1}p^r(1-p)^{\mathbf{x}}$	–	x	#failures
[Wikipedia (French), 2015]	$\binom{k+r-1}{k}p^r(1-p)^{\mathbf{k}}$	$p \in (0, 1)$	$k \in 0, 1, 2, 3, ...$	#failures
[Wikipedia (German), 2015]♣	$\binom{k+r-1}{k}p^r(1-p)^{\mathbf{k}}$	$p \in (0, 1)$	$k \in 0, 1, 2, 3, ...$	#failures
massmatics.de♣	$\binom{k+r-1}{k}p^r(1-p)^{\mathbf{k}}$	$0 < p < 1$	k	#failures
Option 2				
probability of observing a fixed number of successes before certain number of failures				
[Agresti, 2013]	$\binom{y+k-1}{y}(1-p)^k p^{\mathbf{y}}$	–	$y \in 0, 1, 2, ...$	#successes
[Cameron and Trivedi, 2013]	$\binom{y+r-1}{r-1}(1-p)^r p^{\mathbf{y}}$	$0 < p < 1$	$y \in 0, 1, 2, ...$	–
[UncertML Team, 2014]	$\binom{x+r-1}{x}(1-p)^r p^{\mathbf{x}}$	$p \in [0, 1]$	$x \in 0, 1, 2, 3, ...$	#successes
[Wikipedia (English), 2015]	$\binom{k+r-1}{k}(1-p)^r p^{\mathbf{k}}$	$p \in (0, 1)$	$k \in 0, 1, 2, 3, ...$	#successes
[Consul and Famoye, 2006]	$\binom{x+n-1}{x}(1-p)^{\mathbf{n}} p^x$	$0 < p < 1$	$x = 0, 1, 2, 3, ...$	–
Option 3				
probability of number of trials required to achieve certain number of successes				
[Chen and Peace, 2010]	$\binom{n-1}{k-1}p^k(1-p)^{\mathbf{n}-k}$	–	$n \in k, k+1, ...$	#trials
[Dinov et al., 2015]	$\binom{x-1}{k-1}p^k(1-p)^{\mathbf{x}-k}$	$p \in (0, 1]$	$x \in k, k+1, ...$	#trials
[Song and Chen, 2011]	$\binom{x-1}{k-1}p^k(1-p)^{\mathbf{x}-k}$	$0 \leq p \leq 1$	$x \in k, k+1, ...$	–
[Heckert and Filliben, 2003]	$\binom{x-1}{k-1}p^k(1-p)^{\mathbf{x}-k}$	$0 < p < 1$	–	#trials
massmatics.de	$\binom{n-1}{r-1}p^r(1-p)^{\mathbf{n}-r}$	$0 < p < 1$	$n \in N, n \geq r$	#trials
[Wikipedia (German), 2015]	$\binom{n-1}{r-1}p^r(1-p)^{\mathbf{n}-r}$	$0 < p < 1$	$n \in N, n \geq r$	#trials

Table A.2: Overview of different formulation types of NB described or supported in literature and specialised software tools. *Option 1* and *Option 2* are parameterised with the number of success or failures, respectively, while *Option 3* with the total number of successes and failures. All options use the probability of success, p, as second parameter. Some of the well known resources, [STAN Development Team, 2015a] or [Gelman et al., 2014], have not been listed as they feature another version(s) of the negative binomial, NB2 or NB5, see section 2.1. ♣ These sources list *Option 1* as an alternative to *Option 3*.

Note, the first term of the PMF, in Option 1 & 2, can be formulated in various ways and the following equations illustrate their equivalence

$$C^x_{z+x-1} = \binom{x+z-1}{x} = \binom{x+z-1}{z-1} = \frac{(x+z-1)!}{x!\,(z-1)!} = \frac{\Gamma(x+z)}{x!\,\Gamma(z)}.$$

The R and UncertML versions are stored in ProbOnto, as NB1 and NB4, respectively.

Bibliography

[Agresti, 2013] Agresti, A. (2013). *Categorical data analysis*, volume 792 of *Wiley series in probability and statistics*. Wiley, Hoboken, NJ, 3rd ed edition.

[Beal et al., 2009] Beal, S. L., Sheiner, L. B., Boeckmann, A. J., and Bauer, R. J. (2009). NONMEM User's Guides. (1989-2009). Technical report, Icon Development Solutions, Ellicott City, MD, USA.

[Bolker, 2008] Bolker, B. M. (2008). *Ecological models and data in R*. Princeton University Press.

[Bonate, 2011] Bonate, P. L. (2011). *Pharmacokinetic-pharmacodynamic modeling and simulation*. Springer, New York.

[Cameron and Trivedi, 2013] Cameron, A. C. and Trivedi, P. K. (2013). *Regression analysis of count data*, volume 53. Cambridge university press.

[Chen and Peace, 2010] Chen, D. and Peace, K. E. (2010). *Clinical trial data analysis using R*. CRC Press.

[Consul and Famoye, 2006] Consul, P. C. and Famoye, F. (2006). *Lagrangian probability distributions*. Birkhäuser, Boston.

[Cook, 2009] Cook, J. D. (2009). Notes on the Negative Binomial Distribution.

[Devroye, 1986] Devroye, L. (1986). *Non-uniform random variate generation*. Springer-Verlag, New York.

[Dinov et al., 2015] Dinov, I. D., Siegrist, K., Pearl, D. K., Kalinin, A., and Christou, N. (2015). Probability Distributome: a web computational infrastructure for exploring the properties, interrelations, and applications of probability distributions. *Computational Statistics*, pages 1–19.

[Dobson, 2002] Dobson, A. J. (2002). *An introduction to generalized linear models*. Chapman & Hall/CRC texts in statistical science series. Chapman & Hall/CRC, Boca Raton, 2nd ed edition.

[Dodge, 2008] Dodge, Y. (2008). *The concise encyclopedia of statistics*. Springer Science & Business Media.

[Forbes et al., 2011] Forbes, C., Evans, M., Hastings, N., and Peacock, B. (2011). *Statistical distributions*. John Wiley & Sons.

[Gelman et al., 2014] Gelman, A., Carlin, J. B., Stern, H. S., Dunson, D. B., Vehtari, A., and Rubin, D. B. (2014). Bayesian data analysis.

[Hardin et al., 2007] Hardin, J. W., Hilbe, J. M., and Hilbe, J. (2007). *Generalized linear models and extensions*. Stata Press.

[Harnisch et al., 2013] Harnisch, L., Matthews, I., Chard, J., and Karlsson, M. O. (2013). Drug and disease model resources: a consortium to create standards and tools to enhance model-based drug development. *CPT Pharmacometrics Syst Pharmacol*, 2:e34.

[Heckert and Filliben, 2003] Heckert, N. and Filliben, J. J. (2003). NIST Handbook 148: DATAPLOT Reference Manual. *National Institute of Standards and Technology Handbook Series*, 1.

[Hilbe, 2011] Hilbe, J. M. (2011). *Negative binomial regression*. Cambridge University Press.

[Johnson et al., 2005] Johnson, N. L., Kemp, A. W., and Kotz, S. (2005). *Univariate discrete distributions*, volume 444. John Wiley & Sons.

[Kooperberg, 2013] Kooperberg, C. (2013). Logspline: Logspline density estimation routines. R package version 2.1.5.

[Law, 2007] Law, A. (2007). Simulation modeling and analysis. *McGraw-Hill series in industrial engineering and management science Show all parts in this series.*

[LeBauer et al., 2013] LeBauer, D. S., Dietze, M. C., and Bolker, B. M. (2013). Translating probability density functions: From R to BUGS and back again. *R Journal*, 5(1).

[Leemis and Mcqueston, 2008] Leemis, L. M. and Mcqueston, J. T. (2008). Univariate distribution relationships. *The American Statistician*, 62(1):45–53.

[Lesaffre and Lawson, 2012] Lesaffre, E. and Lawson, A. B. (2012). *Bayesian biostatistics.* John Wiley & Sons.

[Lixoft Team, 2014] Lixoft Team (2014). Lixoft, Monolix 4.3.

[Lunn et al., 2002] Lunn, D. J., Best, N., Thomas, A., Wakefield, J., and Spiegelhalter, D. (2002). Bayesian analysis of population pk/pd models: general concepts and software. *J Pharmacokinet Pharmacodyn*, 29(3):271–307.

[Marichev and Trott, 2013] Marichev, O. and Trott, M. (2013). The Ultimate Univariate Probability Distribution Explorer.

[MathWorks, 2015] MathWorks (2015). *MATLAB and Statistics Toolbox.* The MathWorks Inc., Natick, Massachusetts.

[Noy et al., 2009] Noy, N. F., Shah, N. H., Whetzel, P. L., Dai, B., Dorf, M., Griffith, N., Jonquet, C., Rubin, D. L., Storey, M.-A., Chute, C. G., et al. (2009). Bioportal: ontologies and integrated data resources at the click of a mouse. *Nucleic acids research*, page gkp440.

[Paule et al., 2012] Paule, I., Girard, P., Freyer, G., and Tod, M. (2012). Pharmacodynamic models for discrete data. *Clin Pharmacokinet*, 51(12):767–86.

[Plan, 2014] Plan, E. L. (2014). Modeling and simulation of count data. *CPT Pharmacometrics Syst Pharmacol*, 3:e129.

[Plan et al., 2009] Plan, E. L., Maloney, A., Trocóniz, I. F., and Karlsson, M. O. (2009). Performance in population models for count data, part i: maximum likelihood approximations. *J Pharmacokinet Pharmacodyn*, 36(4):353–66.

[Plummer, 2003] Plummer, M. (2003). JAGS: A Program for Analysis of Bayesian Graphical Models Using Gibbs Sampling.

[R Core Team, 2013] R Core Team (2013). *R: A Language and Environment for Statistical Computing.* R Foundation for Statistical Computing, Vienna, Austria. ISBN 3-900051-07-0.

[Rudenski et al., 1991] Rudenski, A. S., Matthews, D. R., Levy, J. C., and Turner, R. C. (1991). Understanding "insulin resistance": both glucose resistance and insulin resistance are required to model human diabetes. *Metabolism*, 40(9):908–17.

[SAS Institute Inc., 2014] SAS Institute Inc. (2014). SAS, Simulation Studio 13.2, User's Guide.

[Scheaffer and Young, 2009] Scheaffer, R. and Young, L. (2009). *Introduction to Probability and its Applications.* Cengage Learning.

[Song and Chen, 2011] Song, W. T. and Chen, Y.-C. (2011). Eighty univariate distributions and their relationships displayed in a matrix format. *Automatic Control, IEEE Transactions on*, 56(8):1979–1984.

[Spiegelhalter et al., 2003] Spiegelhalter, D., Thomas, A., Best, N., and Lunn, D. (2003). WinBUGS User Manual, Version 1.4.

[STAN Development Team, 2015a] STAN Development Team (2015a). Stan Modeling Language Users Guide and Reference Manual, Version 2.7.0. Avaiable at `http://mc-stan.org`.

[STAN Development Team, 2015b] STAN Development Team (2015b). Stan Modeling Language Users Guide and Reference Manual, Version 2.9.0.

[Swat et al., 2015] Swat, M. J., Moodie, S., Wimalaratne, S. M., Kristensen, N. R., Lavielle, M., Mari, A., Magni, P., Smith, M. K., Bizzotto, R., Pasotti, L., Mezzalana, E., Comets, E., Sarr, C., Terranova, N., Blaudez, E., Chan, P., Chard, J., Chatel, K., Chenel, M., Edwards, D., Franklin, C., Giorgino, T., Glont, M., Girard, P., Grenon, P., Harling, K., Hooker, A. C., Kaye, R., Keizer, R., Kloft, C., Kok, J. N., Kokash, N., Laibe, C., Laveille, C., Lestini, G., Mentré, F., Munafo, A., Nordgren, R., Nyberg, H. B., Parra-Guillen, Z. P., Plan, E., Ribba, B., Smith, G., Trocóniz, I. F., Yvon, F., Milligan, P. A., Harnisch, L., Karlsson, M., Hermjakob, H., and Le Novère, N. (2015). Pharmacometrics Markup Language (PharmML): Opening New Perspectives for Model Exchange in Drug Development. *CPT Pharmacometrics Syst Pharmacol*, 4(6):316–9.

[Swat et al., 2014] Swat, M. J., Wimalaratne, S., and Kristensen, N. R. (2014). Changes in PharmML 0.4 and 0.4.1. Technical report, EMBL-EBI.

[Trocóniz et al., 2009] Trocóniz, I. F., Plan, E. L., Miller, R., and Karlsson, M. O. (2009). Modelling overdispersion and markovian features in count data. *J Pharmacokinet Pharmacodyn*, 36(5):461–77.

[UncertML Team, 2014] UncertML Team (2014). Uncertainty Markup Language (UncertML) Version 3.0. URL: http://www.uncertml.org.

[Vidakovic, 2011] Vidakovic, B. (2011). *Statistics for bioengineering sciences: with MATLAB and WinBUGS Support*. Springer Science & Business Media.

[Wang et al., 1996] Wang, P., Puterman, M. L., Cockburn, I., and Le, N. (1996). Mixed poisson regression models with covariate dependent rates. *Biometrics*, 52(2):381–400.

[Weisstein, 2007] Weisstein, E. (2007). Wolfram mathworld.

[Wikipedia (English), 2015] Wikipedia (English) (Retrieved 4th August 2015). Negative binomial distribution — Wikipedia, the free encyclopedia.

[Wikipedia (French), 2015] Wikipedia (French) (Retrieved 4th August 2015). Negative binomial distribution — Wikipedia, the free encyclopedia.

[Wikipedia (German), 2015] Wikipedia (German) (Retrieved 4th August 2015). Negative binomialverteilung — Wikipedia, the free encyclopedia.

[Williams et al., 2008] Williams, M., Cornford, D., Bastin, L., and Ingram, B. (2008). UncertML: An XML Schema for Exchanging Uncertainty. In *Proceedings of the GIS Research UK 16th Annual Conference GISRUK 2008*, pages 275–279.

[Yee, 2008] Yee, T. W. (2008). The VGAM Package. *R News*, 8(2):28–39.

[Yee, 2015] Yee, T. W. (2015). Package VGAM.

[Yu et al., 2013] Yu, D., Huber, W., and Vitek, O. (2013). Shrinkage estimation of dispersion in Negative Binomial models for RNA-seq experiments with small sample size. *Bioinformatics*, 29(10):1275–82.
